# Common and rare variant association analyses in amyotrophic lateral sclerosis identify 15 risk loci with distinct genetic architectures and neuron-specific biology

**DOI:** 10.1038/s41588-021-00973-1

**Published:** 2021-12-06

**Authors:** Wouter van Rheenen, Rick A. A. van der Spek, Mark K. Bakker, Joke J. F. A. van Vugt, Paul J. Hop, Ramona A. J. Zwamborn, Niek de Klein, Harm-Jan Westra, Olivier B. Bakker, Patrick Deelen, Gemma Shireby, Eilis Hannon, Matthieu Moisse, Denis Baird, Restuadi Restuadi, Egor Dolzhenko, Annelot M. Dekker, Klara Gawor, Henk-Jan Westeneng, Gijs H. P. Tazelaar, Kristel R. van Eijk, Maarten Kooyman, Ross P. Byrne, Mark Doherty, Mark Heverin, Ahmad Al Khleifat, Alfredo Iacoangeli, Aleksey Shatunov, Nicola Ticozzi, Johnathan Cooper-Knock, Bradley N. Smith, Marta Gromicho, Siddharthan Chandran, Suvankar Pal, Karen E. Morrison, Pamela J. Shaw, John Hardy, Richard W. Orrell, Michael Sendtner, Thomas Meyer, Nazli Başak, Anneke J. van der Kooi, Antonia Ratti, Isabella Fogh, Cinzia Gellera, Giuseppe Lauria, Stefania Corti, Cristina Cereda, Daisy Sproviero, Sandra D’Alfonso, Gianni Sorarù, Gabriele Siciliano, Massimiliano Filosto, Alessandro Padovani, Adriano Chiò, Andrea Calvo, Cristina Moglia, Maura Brunetti, Antonio Canosa, Maurizio Grassano, Ettore Beghi, Elisabetta Pupillo, Giancarlo Logroscino, Beatrice Nefussy, Alma Osmanovic, Angelica Nordin, Yossef Lerner, Michal Zabari, Marc Gotkine, Robert H. Baloh, Shaughn Bell, Patrick Vourc’h, Philippe Corcia, Philippe Couratier, Stéphanie Millecamps, Vincent Meininger, François Salachas, Jesus S. Mora Pardina, Abdelilah Assialioui, Ricardo Rojas-García, Patrick A. Dion, Jay P. Ross, Albert C. Ludolph, Jochen H. Weishaupt, David Brenner, Axel Freischmidt, Gilbert Bensimon, Alexis Brice, Alexandra Durr, Christine A. M. Payan, Safa Saker-Delye, Nicholas W. Wood, Simon Topp, Rosa Rademakers, Lukas Tittmann, Wolfgang Lieb, Andre Franke, Stephan Ripke, Alice Braun, Julia Kraft, David C. Whiteman, Catherine M. Olsen, Andre G. Uitterlinden, Albert Hofman, Marcella Rietschel, Sven Cichon, Markus M. Nöthen, Philippe Amouyel, SLALOM Consortium, Giancarlo Comi, Nilo Riva, Christian Lunetta, Francesca Gerardi, Maria Sofia Cotelli, Fabrizio Rinaldi, Luca Chiveri, Maria Cristina Guaita, Patrizia Perrone, Mauro Ceroni, Luca Diamanti, Carlo Ferrarese, Lucio Tremolizzo, Maria Luisa Delodovici, Giorgio Bono, PARALS Consortium, Antonio Canosa, Umberto Manera, Rosario Vasta, Alessandro Bombaci, Federico Casale, Giuseppe Fuda, Paolina Salamone, Barbara Iazzolino, Laura Peotta, Paolo Cugnasco, Giovanni De Marco, Maria Claudia Torrieri, Francesca Palumbo, Salvatore Gallone, Marco Barberis, Luca Sbaiz, Salvatore Gentile, Alessandro Mauro, Letizia Mazzini, Fabiola De Marchi, Lucia Corrado, Sandra D’Alfonso, Antonio Bertolotto, Maurizio Gionco, Daniela Leotta, Enrico Odddenino, Daniele Imperiale, Roberto Cavallo, Pietro Pignatta, Marco De Mattei, Claudio Geda, Diego Maria Papurello, Graziano Gusmaroli, Cristoforo Comi, Carmelo Labate, Luigi Ruiz, Delfina Ferrandi, Eugenia Rota, Marco Aguggia, Nicoletta Di Vito, Piero Meineri, Paolo Ghiglione, Nicola Launaro, Michele Dotta, Alessia Di Sapio, Guido Giardini, SLAGEN Consortium, Cinzia Tiloca, Silvia Peverelli, Franco Taroni, Viviana Pensato, Barbara Castellotti, Giacomo P. Comi, Roberto Del Bo, Mauro Ceroni, Stella Gagliardi, Lucia Corrado, Letizia Mazzini, Flavia Raggi, Costanza Simoncini, Annalisa Lo Gerfo, Maurizio Inghilleri, Alessandra Ferlini, SLAP Consortium, Isabella L. Simone, Bruno Passarella, Vito Guerra, Stefano Zoccolella, Cecilia Nozzoli, Ciro Mundi, Maurizio Leone, Michele Zarrelli, Filippo Tamma, Francesco Valluzzi, Gianluigi Calabrese, Giovanni Boero, Augusto Rini, Bryan J. Traynor, Andrew B. Singleton, Miguel Mitne Neto, Ruben J. Cauchi, Roel A. Ophoff, Martina Wiedau-Pazos, Catherine Lomen-Hoerth, Vivianna M. van Deerlin, Julian Grosskreutz, Annekathrin Roediger, Nayana Gaur, Alexander Jörk, Tabea Barthel, Erik Theele, Benjamin Ilse, Beatrice Stubendorff, Otto W. Witte, Robert Steinbach, Christian A. Hübner, Caroline Graff, Lev Brylev, Vera Fominykh, Vera Demeshonok, Anastasia Ataulina, Boris Rogelj, Blaž Koritnik, Janez Zidar, Metka Ravnik-Glavač, Damjan Glavač, Zorica Stević, Vivian Drory, Monica Povedano, Ian P. Blair, Matthew C. Kiernan, Beben Benyamin, Robert D. Henderson, Sarah Furlong, Susan Mathers, Pamela A. McCombe, Merrilee Needham, Shyuan T. Ngo, Garth A. Nicholson, Roger Pamphlett, Dominic B. Rowe, Frederik J. Steyn, Kelly L. Williams, Karen A. Mather, Perminder S. Sachdev, Anjali K. Henders, Leanne Wallace, Mamede de Carvalho, Susana Pinto, Susanne Petri, Markus Weber, Guy A. Rouleau, Vincenzo Silani, Charles J. Curtis, Gerome Breen, Jonathan D. Glass, Robert H. Brown, John E. Landers, Christopher E. Shaw, Peter M. Andersen, Ewout J. N. Groen, Michael A. van Es, R. Jeroen Pasterkamp, Dongsheng Fan, Fleur C. Garton, Allan F. McRae, George Davey Smith, Tom R. Gaunt, Michael A. Eberle, Jonathan Mill, Russell L. McLaughlin, Orla Hardiman, Kevin P. Kenna, Naomi R. Wray, Ellen Tsai, Heiko Runz, Lude Franke, Ammar Al-Chalabi, Philip Van Damme, Leonard H. van den Berg, Jan H. Veldink

**Affiliations:** 1 Department of Neurology, UMC Utrecht Brain Center, University Medical Center Utrecht, Utrecht University, Utrecht, the Netherlands; 2 Department of Genetics, University of Groningen, University Medical Centre Groningen, Groningen, the Netherlands; 3 Department of Genetics, University Medical Center Utrecht, Utrecht University, Utrecht, the Netherlands; 4 University of Exeter Medical School, College of Medicine and Health, University of Exeter, Exeter, UK; 5 Department of Neurosciences, Experimental Neurology and Leuven Brain Institute (LBI), KU Leuven—University of Leuven, Leuven, Belgium; 6 Laboratory of Neurobiology, VIB, Center for Brain & Disease Research, Leuven, Belgium; 7 Department of Neurology, University Hospitals Leuven, Leuven, Belgium; 8 Translational Biology, Biogen, Boston, MA USA; 9 MRC Integrative Epidemiology Unit (IEU), Population Health Sciences, University of Bristol, Bristol, UK; 10 Institute for Molecular Bioscience, University of Queensland, Brisbane, Queensland Australia; 11 Illumina, San Diego, CA USA; 12 Complex Trait Genomics Laboratory, Smurfit Institute of Genetics, Trinity College Dublin, Dublin, Ireland; 13 Academic Unit of Neurology, Trinity Biomedical Sciences Institute, Trinity College Dublin, Dublin, Ireland; 14 Maurice Wohl Clinical Neuroscience Institute, Department of Basic and Clinical Neuroscience, Institute of Psychiatry, Psychology and Neuroscience, King’s College London, London, UK; 15 Department of Biostatistics and Health Informatics, Institute of Psychiatry, Psychology and Neuroscience, King’s College London, London, UK; 16 National Institute for Health Research Biomedical Research Centre and Dementia Unit, South London and Maudsley NHS Foundation Trust and King’s College London, London, UK; 17 Department of Neurology, Stroke Unit and Laboratory of Neuroscience, Istituto Auxologico Italiano IRCCS, Milan, Italy; 18 Department of Pathophysiology and Transplantation, ‘Dino Ferrari’ Center, Università degli Studi di Milano, Milan, Italy; 19 Sheffield Institute for Translational Neuroscience (SITraN), University of Sheffield, Sheffield, UK; 20 Instituto de Fisiologia, Instituto de Medicina Molecular João Lobo Antunes, Faculdade de Medicina, Universidade de Lisboa, Lisbon, Portugal; 21 Euan MacDonald Centre for Motor Neurone Disease Research, Edinburgh, UK; 22 UK Dementia Research Institute, University of Edinburgh, Edinburgh, UK; 23 School of Medicine, Dentistry and Biomedical Sciences, Queen’s University Belfast, Belfast, UK; 24 Department of Molecular Neuroscience, Institute of Neurology, University College London, London, UK; 25 Department of Clinical and Movement Neurosciences, UCL Queen Square Institute of Neurology, University College London, London, UK; 26 Institute of Clinical Neurobiology, University Hospital Würzburg, Würzburg, Germany; 27 Charité University Hospital, Humboldt University, Berlin, Germany; 28 Koç University, School of Medicine, KUTTAM-NDAL, Istanbul, Turkey; 29 Department of Neurology, Academic Medical Center, Amsterdam, the Netherlands; 30 Department of Medical Biotechnology and Translational Medicine, Università degli Studi di Milano, Milan, Italy; 31 Unit of Medical Genetics and Neurogenetics, Fondazione IRCCS Istituto Neurologico ‘Carlo Besta’, Milan, Italy; 32 3rd Neurology Unit, Motor Neuron Diseases Center, Fondazione IRCCS Istituto Neurologico ‘Carlo Besta’, MIlan, Italy; 33 Department of Medical Biotechnology and Translational Medicine, University of Milan, Milan, Italy; 34 Neurology Unit, IRCCS Foundation Ca’ Granda Ospedale Maggiore Policlinico, Milan, Italy; 35 Genomic and Post-Genomic Center, IRCCS Mondino Foundation, Pavia, Italy; 36 Department of Health Sciences, University of Eastern Piedmont, Novara, Italy; 37 Department of Neurosciences, University of Padova, Padova, Italy; 38 Department of Clinical and Experimental Medicine, University of Pisa, Pisa, Italy; 39 Department of Clinical and Experimental Sciences, University of Brescia, Brescia, Italy; 40 ’Rita Levi Montalcini’ Department of Neuroscience, ALS Centre, University of Torino, Turin, Italy; 41 Neurologia 1, Azienda Ospedaliero Universitaria Città della Salute e della Scienza, Turin, Italy; 42 Laboratory of Neurological Diseases, Department of Neuroscience, Istituto di Ricerche Farmacologiche Mario Negri IRCCS, Milan, Italy; 43 Department of Clinical Research in Neurology, University of Bari at ‘Pia Fondazione Card G. Panico’ Hospital, Bari, Italy; 44 Neuromuscular Diseases Unit, Department of Neurology, Tel Aviv Sourasky Medical Center, Tel Aviv, Israel; 45 Department of Neurology, Hannover Medical School, Hannover, Germany; 46 Essener Zentrum für Seltene Erkrankungen (EZSE), University Hospital Essen, Essen, Germany; 47 Department of Clinical Sciences, Neurosciences, Umeå University, Umeå, Sweden; 48 Faculty of Medicine, Hebrew University of Jerusalem, Jerusalem, Israel; 49 Department of Neurology, the Agnes Ginges Center for Human Neurogenetics, Hadassah Medical Center, Jerusalem, Israel; 50 Center for Neural Science and Medicine, Cedars-Sinai Medical Center, Los Angeles, CA USA; 51 Department of Neurology, Neuromuscular Division, Cedars-Sinai Medical Center, Los Angeles, CA USA; 52 Service de Biochimie et Biologie Moléculaire, CHU de Tours, Tours, France; 53 UMR 1253, Université de Tours, Inserm, Tours, France; 54 Centre de référence sur la SLA, CHU de Tours, Tours, France; 55 Centre de référence sur la SLA, CHRU de Limoges, Limoges, France; 56 UMR 1094, Université de Limoges, Inserm, Limoges, France; 57 ICM, Institut du Cerveau, Inserm, CNRS, Sorbonne Université, Hôpital Pitié-Salpêtrière, Paris, France; 58 Hôpital des Peupliers, Ramsay Générale de Santé, Paris, France; 59 Département de Neurologie, Centre de référence SLA Ile de France, Hôpital de la Pitié-Salpêtrière, AP-HP, Paris, France; 60 ALS Unit, Hospital San Rafael, Madrid, Spain; 61 Functional Unit of Amyotrophic Lateral Sclerosis (UFELA), Service of Neurology, Bellvitge University Hospital, L’Hospitalet de Llobregat, Barcelona, Spain; 62 MND Clinic, Neurology Department, Hospital de la Santa Creu i Sant Pau de Barcelona, Universitat Autonoma de Barcelona, Barcelona, Spain; 63 Montreal Neurological Institute and Hospital, McGill University, Montreal, Quebec Canada; 64 Department of Neurology and Neurosurgery, McGill University, Montreal, Quebec Canada; 65 Department of Human Genetics, McGill University, Montreal, Quebec Canada; 66 Department of Neurology, Ulm University, Ulm, Germany; 67 Division of Neurodegeneration, Department of Neurology, University Medicine Mannheim, Medical Faculty Mannheim, Heidelberg University, Mannheim, Germany; 68 German Center for Neurodegenerative Diseases (DZNE) Ulm, Ulm, Germany; 69 Département de Pharmacologie Clinique, Hôpital de la Pitié-Salpêtrière, UPMC Pharmacologie, AP-HP, Paris, France; 70 Pharmacologie Sorbonne Université, Paris, France; 71 Institut du Cerveau, Paris Brain Institute ICM, Paris, France; 72 Laboratoire de Biostatistique, Epidémiologie Clinique, Santé Publique Innovation et Méthodologie (BESPIM), CHU-Nîmes, Nîmes, France; 73 Sorbonne Université, Paris Brain Institute, APHP, INSERM, CNRS, Hôpital de la Pitié Salpêtrière, Paris, France; 74 Genethon, CNRS UMR, Evry, France; 75 Department of Clinical and Movement Neuroscience, UCL Institute of Neurology, Queen Square, London, UK; 76 Department of Neuroscience, Mayo Clinic College of Medicine, Jacksonville, FL USA; 77 Popgen Biobank and Institute of Epidemiology, Christian Albrechts-University Kiel, Kiel, Germany; 78 Institute of Clinical Molecular Biology, Kiel University, Kiel, Germany; 79 Analytic and Translational Genetics Unit, Massachusetts General Hospital, Boston, MA USA; 80 Stanley Center for Psychiatric Research, Broad Institute of MIT and Harvard, Cambridge, MA USA; 81 Department of Psychiatry and Psychotherapy, Charité—Universitätsmedizin, Berlin, Germany; 82 Cancer Control Group, QIMR Berghofer Medical Research Institute, Herston, Queensland Australia; 83 Department of Internal Medicine, Genetics Laboratory, Erasmus Medical Center Rotterdam, Rotterdam, the Netherlands; 84 Department of Epidemiology, Erasmus Medical Center Rotterdam, Rotterdam, the Netherlands; 85 Medical Faculty Mannheim, University of Heidelberg, Heidelberg, Germany; 86 Central Institute of Mental Health, Mannheim, Germany; 87 Institute of Human Genetics, University of Bonn, Bonn, Germany; 88 Department of Genomics, Life and Brain Center, Bonn, Germany; 89 Division of Medical Genetics, University Hospital Basel and Department of Biomedicine, University of Basel, Basel, Switzerland; 90 Institute of Neuroscience and Medicine INM-1, Research Center Juelich, Juelich, Germany; 91 INSERM UMR1167—RID-AGE LabEx DISTALZ—Risk Factors and Molecular Determinants of Aging-Related Diseases, University of Lille, Centre Hospitalier of the University of Lille, Institut Pasteur de Lille, Lille, France; 92 Neuromuscular Diseases Research Section, Laboratory of Neurogenetics, National Institute on Aging, NIH, Porter Neuroscience Research Center, Bethesda, MD USA; 93 Department of Neurology, Johns Hopkins University, Baltimore, MD USA; 94 Molecular Genetics Section, Laboratory of Neurogenetics, National Institute on Aging, NIH, Porter Neuroscience Research Center, Bethesda, MD USA; 95 Universidade de São Paulo, São Paulo, Brazil; 96 Centre for Molecular Medicine and Biobanking and Department of Physiology and Biochemistry, Faculty of Medicine and Surgery, University of Malta, Msida, Malta; 97 University Medical Center Utrecht, Department of Psychiatry, Rudolf Magnus Institute of Neuroscience, Utrecht, the Netherlands; 98 Department of Human Genetics, David Geffen School of Medicine, University of California, Los Angeles, CA USA; 99 Center for Neurobehavioral Genetics, Semel Institute for Neuroscience and Human Behavior, University of California, Los Angeles, CA USA; 100 Department of Neurology, David Geffen School of Medicine, University of California, Los Angeles, CA USA; 101 Department of Neurology, University of California, San Francisco, CA USA; 102 Center for Neurodegenerative Disease Research, Perelman School of Medicine at the University of Pennsylvania, Philadelphia, PA USA; 103 Hans Berger Department of Neurology, Jena University Hospital, Jena, Germany; 104 Precision Neurology Unit, Department of Neurology, University Hospital Schleswig-Holstein, University of Luebeck, Luebeck, Germany; 105 Institute of Human Genetics, Jena University Hospital, Jena, Germany; 106 Department of Geriatric Medicine, Karolinska University Hospital Huddinge, Stockholm, Sweden; 107 Department of Neurology, Bujanov Moscow Clinical Hospital, Moscow, Russia; 108 Moscow Research and Clinical Center for Neuropsychiatry of the Healthcare Department, Moscow, Russia; 109 Department of Functional Biochemistry of the Nervous System, Institute of Higher Nervous Activity and Neurophysiology Russian Academy of Sciences, Moscow, Russia; 110 ALS-Care Center, ‘GAOORDI’, Medical Clinic of the St. Petersburg, St. Petersburg, Russia; 111 Department of Biotechnology, Jožef Stefan Institute, Ljubljana, Slovenia; 112 Biomedical Research Institute BRIS, Ljubljana, Slovenia; 113 Faculty of Chemistry and Chemical Technology, University of Ljubljana, Ljubljana, Slovenia; 114 Ljubljana ALS Centre, Institute of Clinical Neurophysiology, University Medical Centre Ljubljana, Ljubljana, Slovenia; 115 Institute of Biochemistry and Molecular Genetics, Faculty of Medicine, University of Ljubljana, Ljubljana, Slovenia; 116 Department of Molecular Genetics, Institute of Pathology, Faculty of Medicine, University of Ljubljana, Ljubljana, Slovenia; 117 Clinic of Neurology, Clinical Center of Serbia, School of Medicine, University of Belgrade, Belgrade, Serbia; 118 Sackler Faculty of Medicine, Tel Aviv University, Tel Aviv, Israel; 119 Centre for Motor Neuron Disease Research, Faculty of Medicine, Health and Human Sciences, Macquarie University, Sydney, New South Wales Australia; 120 Brain and Mind Centre, University of Sydney, Sydney, New South Wales Australia; 121 Australian Centre for Precision Health and Allied Health and Human Performance, University of South Australia, Adelaide, South Australia Australia; 122 Centre for Clinical Research, Australian Institute for Bioengineering and Nanotechnology, University of Queensland, Brisbane, Queensland Australia; 123 Department of Neurology, Royal Brisbane and Women’s Hospital, Brisbane, Queensland Australia; 124 Calvary Health Care Bethlehem, Parkdale, Victoria Australia; 125 Queensland Brain Institute, University of Queensland, Brisbane, Queensland Australia; 126 Fiona Stanley Hospital, Perth, Western Australia Australia; 127 Notre Dame University, Fremantle, Western Australia Australia; 128 Centre for Molecular Medicine and Innovative Therapeutics, Health Futures Institute, Murdoch University, Perth, Western Australia Australia; 129 Northcott Neuroscience Laboratory, ANZAC Research Institute, Concord, New South Wales Australia; 130 Molecular Medicine Laboratory, Concord Repatriation General Hospital, Concord, New South Wales Australia; 131 Discipline of Pathology and Department of Neuropathology, Brain and Mind Centre, University of Sydney, Sydney, New South Wales Australia; 132 The School of Biomedical Sciences, Faculty of Medicine, University of Queensland, Brisbane, Queensland Australia; 133 Centre for Healthy Brain Ageing, School of Psychiatry, University of New South Wales, Sydney, New South Wales Australia; 134 Neuroscience Research Australia Institute, Randwick, New South Wales Australia; 135 Neuropsychiatric Institute, the Prince of Wales Hospital, UNSW, Randwick, New South Wales Australia; 136 Neuromuscular Diseases Unit/ALS Clinic, Kantonsspital St. Gallen, St. Gallen, Switzerland; 137 Social Genetic & Developmental Psychiatry Centre, Institute of Psychiatry, Psychology and Neuroscience (IoPPN), King’s College London, London, UK; 138 NIHR BioResource Centre Maudsley, NIHR Maudsley Biomedical Research Centre (BRC) at South London and Maudsley NHS Foundation Trust (SLaM) & Institute of Psychiatry, Psychology and Neuroscience (IoPPN), King’s College London, London, UK; 139 Department Neurology, Emory University School of Medicine, Atlanta, GA USA; 140 Department of Neurology, University of Massachusetts Medical School, Worcester, MA USA; 141 Department of Translational Neuroscience, UMC Utrecht Brain Center, University Medical Center Utrecht, Utrecht University, Utrecht, the Netherlands; 142 Department of Neurology, Third Hospital, Peking University, Beijing, China; 143 Population Health Science, Bristol Medical School, Bristol, UK; 144 King’s College Hospital, London, UK; 145 IRCCS San Raffaele Hospital, Milan, Italy; 146 Vita Salute San Raffaele University, Milan, Italy; 147 Casa di Cura del Policlinico, Milan, Italy; 148 NEMO Clinical Center, Serena Onlus Foundation, Niguarda Ca’ Granda Hospital, Milan, Italy; 149 Civil Hospital of Brescia, Brescia, Italy; 150 Neurology Unit, ASST Valcamonica, Esine, Brescia Italy; 151 Ospedale Valduce, Como, Italy; 152 AO Ospedale Civile di Legnano, Legnano, Italy; 153 IRCCS Istituto Neurologico Nazionale ‘C. Mondino’, Pavia, Italy; 154 AO ‘San Gerardo’ di Monza and University of Milano-Bicocca, Milano-Bicocca, Italy; 155 AO ‘Ospedale di Circolo Fondazione Macchi’ di Varese, Varese, Italy; 156 Neurology Unit 1U, Azienda Ospedaliero Universitaria Città della Salute e della Scienza di Torino, Turin, Italy; 157 Department of Medical Genetics, Azienda Ospedaliero Universitaria Città della Salute e della Scienza, Turin, Italy; 158 Neurologia 3, Azienda Ospedaliero Universitaria Città della Salute e della Scienza di Torino, Turin, Italy; 159 Istituto Auxologico Italiano, IRCCS, Piancavallo, Italy; 160 Department of Neurology, ‘Amedeo Avogadro’ University of Piemonte Orientale, Novara, Italy; 161 Azienda Ospedaliero Universitaria ‘Maggiore della Carità’, Novara, Italy; 162 Department of Health Sciences, ‘Amedeo Avogadro’ University of Piemonte Orientale, Novara, Italy; 163 Department of Neurology and Multiple Sclerosis Center, Azienda Ospedaliero Universitaria San Luigi, Orbassano, Italy; 164 Department of Neurology, Azienda Ospedaliera ‘Ordine Mauriziano’ di Torino, Turin, Italy; 165 Department of Neurology, Ospedale Martini, ASL Città di Torino, Turin, Italy; 166 Department of Neurology, Ospedale Maria Vittoria, ASL Città di Torino, Turin, Italy; 167 Department of Neurology, Ospedale San Giovanni Bosco, ASL Città di Torino, Turin, Italy; 168 Ospedale Humanitas Gradenigo, Turin, Italy; 169 Department of Neurology, Ospedale ‘Santa Croce’ di Moncalieri, ASL Torino 5, Moncaliari, Italy; 170 Department of Neurology, Ospedale Civile di Ivrea, ASL Torino 4, Ivrea, Italy; 171 Department of Neurology, Presidio Ospedaliero di Ciriè, ASL Torino 4, Ciriè, Italy; 172 Department of Neurology, Ospedale ‘Degli Infermi’ di Biella, ASL Biella, Ponderano, Italy; 173 Department of Neurology, Ospedale ‘Sant’Andrea’ di Vercelli, ASL Vercelli, Vercelli, Italy; 174 Department of Clinical and Experimental Medicine, ‘Amedeo Avogadro’ University of Piemonte Orientale, Novara, Italy; 175 Department of Neurology, Ospedale Civile ‘Edoardo Agnelli’ di Pinerolo, ALS Torino 2, Pinerolo, Italy; 176 Department of Neurology, Azienda Ospedaliera ‘Santi Antonio e Biagio’ di Alesssandria, Alessandria, Italy; 177 Department of Neurology, Ospedale ‘Santo Spirito’ di Casale Monferrato, ASL Alessandria, Casale Monferrato, Italy; 178 Department of Neurology, Ospedale ‘San Giacomo’ di Novi Ligure, ASL Alesssandria, Novi Ligure, Italy; 179 Department of Neurology, Ospedale ‘Cardinal Massia’ di Asti, ASL Asti, Asti, Italy; 180 Department of Neurology, Azienda Ospedaliera ‘Santa Croce e Carle’ di Cuneo, Cuneo, Italy; 181 Department of Neurology, Ospedale ‘Maggiore Santissima Annuziata’ di Savigliano, ASL Cuneo 1, Savigliano, Italy; 182 Department of Anesthesiology, Ospedale ‘Maggiore Santissima Annuziata’ di Savigliano, ASL Cuneo 1, Savigliano, Italy; 183 Department of Neurology, Ospedale ‘Michele e Pietro Ferrero’ di Verduno, ASL Cuneo 2, Verduno, Italy; 184 Department of Neurology, Ospedale ‘Regina Montis Regalis’ di Mondovì, ASL Cuneo 1, Aosta, Italy; 185 Department of Neurology, Ospedale Regionale ‘Umberto Parini’ di Aosta, Aosta, Italy; 186 Unit of General Neurology, IRCCS Mondino Foundation, Pavia, Italy; 187 Department of Brain and Behavioural Sciences, University of Pavia, Pavia, Italy; 188 ALS Center, Department of Neurology, Azienda Ospedaliero Universitaria Maggiore della Carità, Novara, Italy; 189 Rare Neuromuscular Diseases Centre, Department of Human Neuroscience, Sapienza University, Rome, Italy; 190 Unit of Medical Genetics, Department of Medical Science, University of Ferrara, Ferrara, Italy; 191 Department of Basic Medical Sciences, Neurosciences and Sense Organs, University of Bari, Bari, Italy; 192 Neurological Department, Antonio Perrino’s Hospital, Brindisi, Italy; 193 National Institute of Digestive Diseases, IRCCS S. de Bellis Research Hospital, Castellana Grotte, Italy; 194 ASL Bari, San Paolo Hospital, Bari, Italy; 195 Department of Neuroscience, United Hospital of Foggia, Foggia, Italy; 196 Unit of Neurology, Department of Emergency and Critical Care, Fondazione IRCCS Casa Sollievo della Sofferenza, San Giovanni Rotondo, Italy; 197 Neurology Unit, Miulli Hospital, Acquaviva delle Fonti, Italy; 198 Unit of Neurology, ‘S. Giacomo’ Hospital, Bari, Italy; 199 Department of Neurology, ASL (Local Health Authority) at the ‘V Fazzi’ Hospital, Lecce, Italy; 200 Department of Neurology, ASL (Local Health Authority) at the ‘SS Annunziata’ Hospital, Taranto, Italy

**Keywords:** Motor neuron disease, Genome-wide association studies, Neurodegenerative diseases

## Abstract

Amyotrophic lateral sclerosis (ALS) is a fatal neurodegenerative disease with a lifetime risk of one in 350 people and an unmet need for disease-modifying therapies. We conducted a cross-ancestry genome-wide association study (GWAS) including 29,612 patients with ALS and 122,656 controls, which identified 15 risk loci. When combined with 8,953 individuals with whole-genome sequencing (6,538 patients, 2,415 controls) and a large cortex-derived expression quantitative trait locus (eQTL) dataset (MetaBrain), analyses revealed locus-specific genetic architectures in which we prioritized genes either through rare variants, short tandem repeats or regulatory effects. ALS-associated risk loci were shared with multiple traits within the neurodegenerative spectrum but with distinct enrichment patterns across brain regions and cell types. Of the environmental and lifestyle risk factors obtained from the literature, Mendelian randomization analyses indicated a causal role for high cholesterol levels. The combination of all ALS-associated signals reveals a role for perturbations in vesicle-mediated transport and autophagy and provides evidence for cell-autonomous disease initiation in glutamatergic neurons.

## Abstract (web summary)

A cross-ancestry genome-wide association meta-analysis of amyotrophic lateral sclerosis (ALS) including 29,612 patients with ALS and 122,656 controls identifies 15 risk loci with distinct genetic architectures and neuron-specific biology.

## Main

ALS is a fatal neurodegenerative disease affecting one in 350 individuals. Due to degeneration of both upper and lower motor neurons, patients suffer from progressive paralysis, ultimately leading to respiratory failure within 3–5 years after disease onset^1^. In ~10% of patients with ALS, there is a clear family history for ALS, suggesting a strong genetic predisposition, and currently a pathogenic mutation can be found in more than half of these cases^2^. On the other hand, apparently sporadic ALS is considered a complex trait for which heritability is estimated at 40–50% (refs. ^3,4^). There is no widely accepted definition of familial or sporadic ALS^5^, and they are likely to represent the ends of a spectrum with overlapping genetic architectures for which the same genes have been implicated in both familial and sporadic disease^6–11^. To date, partially overlapping GWASs have identified up to six genome-wide significant loci, explaining a small proportion of the genetic susceptibility to ALS^11–16^. Indeed, some of these loci found in GWASs harbor rare variants with large effects also present in familial cases (for example, *C9orf72* and *TBK1*)^6,17,18^. For other loci, the role of rare variants remains unknown.

While ALS is referred to as a motor neuron disease, cognitive and behavioral changes are observed in up to 50% of patients, sometimes leading to frontotemporal dementia (FTD). The overlap with FTD is clearly illustrated by the pathogenic hexanucleotide repeat expansion in *C9orf72*, which causes familial ALS and/or FTD^17,18^ and the genome-wide genetic correlation between ALS and FTD^19^. Further expanding the ALS–FTD spectrum, a genetic correlation with progressive supranuclear palsy (PSP) has been described^20^. Shared pathogenic mechanisms between ALS and other neurodegenerative diseases, including common diseases such as Alzheimer’s disease (AD) and Parkinson’s disease (PD), can further reveal ALS pathophysiology and inform new therapeutic strategies.

Here, we combine new and existing individual-level genotype data in the largest GWAS of ALS to date. We present a comprehensive screen for pathogenic rare variants and short tandem repeat (STR) expansions as well as regulatory effects observed in brain cortex-derived RNA sequencing (RNA-seq) and methylation datasets to prioritize causal genes within ALS-risk loci. Furthermore, we reveal similarities and differences between ALS and other neurodegenerative diseases as well as the biological processes in disease-relevant tissues and cell types that affect ALS risk.

## Results

### Cross-ancestry meta-analysis reveals 15 risk loci for ALS

**Fig. 1:**
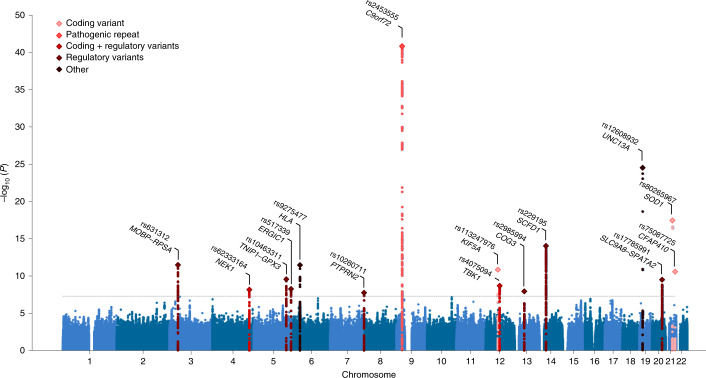
Manhattan plot of cross-ancestry meta-analysis. Genome-wide association statistics obtained by IVW meta-analysis of the stratified SAIGE logistic mixed model regression. The *y* axis corresponds to two-tailed −log_10_ (*P*values); the *x* axis corresponds to genomic coordinates (GRCh37). The horizontal dashed line reflects the threshold for calling genome-wide significant SNPs (*P* = 5 × 10^−8^). Color coding and gene labels reflect those prioritized by the gene-prioritization analysis. Labels in bold indicate genes with known highly pathogenic mutations for ALS. SAIGE = Scalable and Accurate Implementation of Generalized mixed model software package. Source data

**Extended Data Fig. 1:**
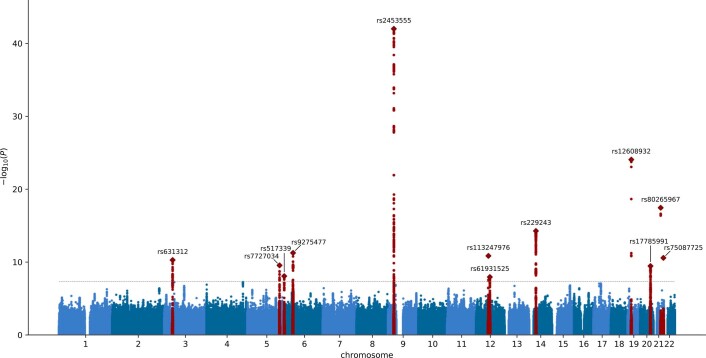
Manhattan plot in European ancestries GWAS. Genome-wide association statistics obtained by inverse-variance weighted meta-analysis of the stratified SAIGE logistic mixed model regression in European ancestry cohorts. Y-axis corresponds to the two-tailed -log_10_(*P*-value), x-axis corresponds to the genomic coordinates (GRCh37). Loci containing a genome-wide significant SNP are highlighted in red. SNP IDs are the top associated SNPs in each locus. The dotted horizontal line reflects the threshold for genome-wide significance (*P* = 5 × 10^−8^). Source data

To generate the largest GWAS of ALS to date, we merged individual-level genotype data from 117 cohorts into six strata matched by genotyping platform. A total of 27,205 patients with ALS and 110,881 control participants of European ancestries passed quality control (including 6,374 newly genotyped cases and 22,526 control participants; Methods and Supplementary Tables 1 and 2). Patients were not selected for a family history of ALS. Through meta-analysis of these six strata, we obtained association statistics for 10,461,755 variants down to a minor allele frequency (MAF) of 0.1% in the Haplotype Reference Consortium resource^21^. We observed moderate inflation of the test statistics (*λ*_GC_ = 1.12, *λ*_1000_ = 1.003), and linkage disequilibrium (LD) score regression yielded an intercept of 1.029 (s.e. = 0.0073), indicating that the majority of inflation was due to the polygenic signal in ALS (LD score regression (LDSC): $h_{\textrm{l}}^2$ = 0.028, s.e. = 0.003, *K* = 350^−1^, *P* = 5.5 × 10^−21^). The European ancestry analysis identified 12 loci reaching genome-wide significance (*P* < 5.0 × 10^−8^; Extended Data Fig. 1). For nine loci, the top SNP or a strong LD proxy (*r*^2^ = 0.996) was present in GWAS of ALS in Asian ancestries (2,407 patients with ALS and 11,775 control participants)^15,16^, and all showed a consistent direction of effects (*P*_binom_ = 2.0 × 10^−3^). The three SNPs that were not present in the Asian ancestry GWAS were low-frequency variants (MAF of 0.6–1.6% in European ancestries, Table 1). The genetic overlap between ALS risk in European and Asian ancestries resulted in a trans-ancestry genetic correlation of 0.57 (s.e. = 0.28) for genetic effect and 0.58 (s.e. = 0.30) for genetic impact, which were not statistically significantly different from unity (*P* = 0.13 and *P* = 0.16, respectively). We therefore performed a cross-ancestry meta-analysis totaling 29,612 cases and 122,656 controls, which revealed three additional loci, totaling 15 genome-wide significant risk loci for ALS risk (Fig. 1, Table 1 and Supplementary Tables 4–18). Conditional and joint analysis did not identify secondary signals within these loci.

**Table 1:** Genome-wide significant loci

							European ancestries		Asian ancestries		Cross-ancestry	
Chr	Position (bp)	ID	Prioritized gene	A_1_	A_2_	Freq	Effect (s.e.)	*P*	Effect (s.e.)	*P*	Effect (s.e.)	*P*
9	27,563,868	rs2453555	*C9orf72*	A	G	0.248	0.174 (0.013)	1.0 × 10^−43^	0.017 (0.066)	0.80	0.168 (0.012)	1.5 × 10^−41^
19	17,752,689	rs12608932	*UNC13A*	C	A	0.347	0.125 (0.012)	8.8 × 10^−25^	0.074 (0.038)	0.053	0.120 (0.012)	3.0 × 10^−25^
21	33,039,603	rs80265967	*SOD1*	C	A	0.006	1.078 (0.124)	3.5 × 10^−18^	–	–	–	–
14	31,045,596	rs229195	*SCFD1*	A	G	0.337	0.091 (0.012)	9.2 × 10^−15^	–	–	–	–
14	31,045,181	rs229194^a^	*SCFD1*	A	G	0.337	0.091 (0.012)	9.2 × 10^−15^	0.002 (0.036)	0.97	0.083 (0.011)	1.5 × 10^−13^
3	39,508,968	rs631312	*MOBP*, *RPSA*	G	A	0.291	0.079 (0.012)	5.2 × 10^−11^	0.084 (0.036)	0.020	0.080 (0.011)	3.3 × 10^−12^
6	32,672,641	rs9275477	*HLA*	C	A	0.096	−0.143 (0.021)	5.5 × 10^−12^	−0.110 (0.111)	0.32	−0.142 (0.02)	3.5 × 10^−12^
12	57,975,700	rs113247976	*KIF5A*	T	A	0.016	0.332 (0.049)	1.4 × 10^−11^	–	–	–	–
21	45,753,117	rs75087725	*CFAP410*	A	C	0.012	0.418 (0.063)	2.7 × 10^−11^	–	–	–	–
5	150,410,835	rs10463311	*GPX3*, *TNIP1*	C	T	0.253	0.079 (0.013)	3.5 × 10^−10^	0.042 (0.036)	0.24	0.075 (0.012)	2.7 × 10^−10^
20	48,438,761	rs17785991	*SLC9A8*, *SPATA2*	A	T	0.353	0.074 (0.012)	3.5 × 10^−10^	0.045 (0.076)	0.55	0.073 (0.012)	3.2 × 10^−10^
12	64,877,053	rs4075094	*TBK1*	A	T	0.112	−0.098 (0.018)	1.7 × 10^−8^	−0.216 (0.090)	0.017	−0.103 (0.017)	2.1 × 10^−9^
5	172,354,731	rs517339	*ERGIC1*	C	T	0.397	−0.065 (0.011)	8.5 × 10^−9^	−0.067 (0.074)	0.37	−0.065 (0.011)	5.6 × 10^−9^
4	170,583,157	rs62333164	*NEK1*	A	G	0.335	0.063 (0.012)	7.0 × 10^−8^	0.203 (0.070)	3.8 × 10^−3^	0.067 (0.012)	6.9 × 10^−9^
13	46,113,984	rs2985994	*COG3*	C	T	0.259	0.066 (0.013)	1.9 × 10^−7^	0.100 (0.041)	0.014	0.069 (0.012)	1.2 × 10^−8^
7	157,481,780	rs10280711	*PTPRN2*	G	C	0.124	0.076 (0.017)	5.8 × 10^−6^	0.132 (0.037)	2.9 × 10^−4^	0.086 (0.015)	1.8 × 10^−8^

Details of two-sided SAIGE logistic mixed model regression for the top associated SNPs within each genome-wide significant locus (*P* < 5 × 10^−8^). ^a^For the strongest associated SNP in the *SCFD1* locus, rs229195 (MAF = 0.337), details of the LD proxy rs229194 are described (MAF = 0.337, *r*^2^ = 0.996 in Asian ancestries), as only the LD proxy was present in the Asian ancestry GWAS. The low-frequency SNPs rs80265967, rs113247976 and rs75087725 were not present in the Asian ancestry GWAS, and no LD proxies (*r*^2^ > 0.8) were found. Chr, chromosome; Position, basepair position in the reference genome GRCh37; A_1_, effect allele; A_2_, non-effect allele; Freq, frequency of the effect allele in the European ancestry GWAS; s.e., standard error of the effect estimate.

Of these findings, eight loci have been reported in previous GWASs (*C9orf72*, *UNC13A*, *SCFD1*, *MOBP*–*RPSA*, *KIF5A*, *CFAP410*, *GPX3*–*TNIP1* and *TBK1*)^11,14,15^. The rs80265967 variant corresponds to the p.D90A mutation in *SOD1* previously identified in a Finnish ALS cohort enriched for familial ALS^13^. Interestingly, we observed a genome-wide significant common variant association signal within the *NEK1* locus, which was previously shown to harbor rare variants associated with ALS^8^. The recently reported association at the *ACSL5*–*ZDHHC6* locus^16,22^ did not reach the threshold for genome-wide significance (rs58854276, *P*_EUR_ = 5.4 × 10^−5^, *P*_ASN_ = 4.9 × 10^−7^, *P*_comb_ = 6.5 × 10^−8^; Supplementary Table 19), despite the fact that our analysis includes all data from the original discovery studies.

### Rare variant gene-based association analyses in ALS

**Extended Data Fig. 2:**
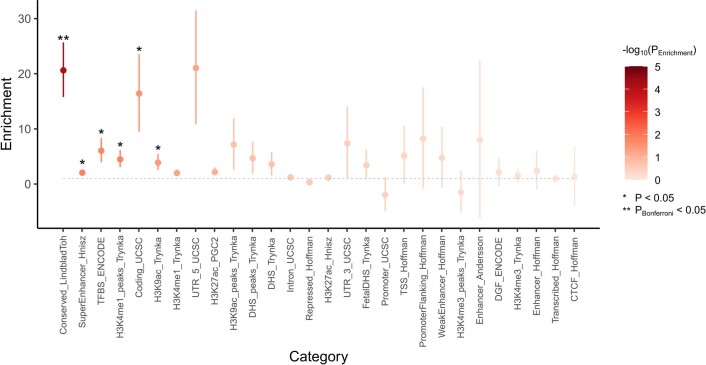
Annotation specific heritability enrichment. Enrichment of SNP-based heritability was calculated with LD-score regression. Grey dashed line represents no enrichment (enrichment = 1). Error bars denote standard error of enrichment estimate. Nominal statistically significant enrichment estimates (two-sided P < 0.05) are marked with an asterisk (*Conserved_LindbladToh* P = 6.5 × 10^−5^, *SuperEnhancer_Hnisz* P = 0.014, *TFBS_ENCODE* P = 0.017, *H3K4me1_peaks_Trynka* P = 0.018, *Coding_UCSC* P = 0.028, *H3K9ac_Trynka* P = 0.037). The category *Conserved_LindbladToh* was significant after Bonferroni correction for multiple testing across all categories (N = 28). Due to the regression framework in LDSC, enrichment estimates < 0 are possible (with large standard errors). Source data

**Extended Data Fig. 3:**
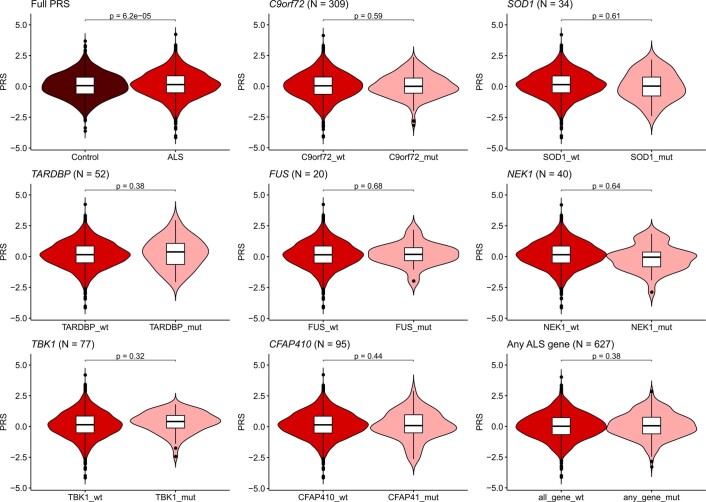
PRS stratified by rare variant carrier status. Distribution of PRS in controls and ALS patients with or without one or more rare variants in ALS risk genes. There was no statistically significant difference in PRS between ALS patients with and without rare variants in ALS risk genes (labeled as *gene_mut* or *gene_wt* respectively). In total, 5,112 ALS patients and 2,132 controls from stratum 6 with whole-genome sequencing data available were included. For *SOD1*, *TARDBP*, *FUS*, *NEK1*, *TBK1*, and *CFAP410*, rare variants were included according to the model that yielded the strongest association in the rare variant burden association analyses. For *C9orf72*, patients with the pathogenic hexanucleotide repeat expansion were compared to those without the expansion. The ‘any ALS gene’ groups all patients together with a rare variant in any of the ALS risk genes. *P*-values for difference in PRS were derived by two-tailed logistic regression. The number of ALS patients carrying a rare variant per gene is denoted in the corresponding panel. Intervals for boxplots: center = median, box = lower and upper quartile, hinges = median ± 2 * IQR, IQR = interquartile range. Source data

To assess a general pattern of underlying architectures that link associated SNPs to causal genes, we first tested for annotation-specific enrichment using stratified LDSC. This revealed that 5′ UTR regions as well as coding regions in the genome and those annotated as conserved were most enriched for ALS-associated SNPs (Extended Data Fig. 2). Subsequently, we investigated how rare, coding variants contributed to ALS risk by generating a whole-genome sequencing (WGS) dataset of patients with ALS (*n* = 6,538) and control participants (*n* = 2,415), which is a subset of the common variant GWAS cohort. The exome-wide association analysis included transcript-level rare variant burden testing for different models of allele-frequency thresholds and variant annotations (Methods). This identified *NEK1* as the strongest associated gene (minimal *P* = 4.9 × 10^−8^ for disruptive and damaging variants at MAF < 0.005), which was the only gene to pass the exome-wide significance thresholds (0.05 ÷ 17,994 = 2.8 × 10^−6^ and 0.05 ÷ 58,058 = 8.6 × 10^−7^ for number of genes and protein-coding transcripts, respectively; Supplementary Table 20). This association was independent from the previously reported increased rare variant burden in selected patients with ‘familial ALS’ (ref. ^8^) who were not included in this study. Polygenic risk score (PRS) analyses did not illustrate a difference in PRSs in patients carrying rare variants in ALS-risk genes (*SOD1*, *C9orf72* repeat expansion, *TARDBP*, *FUS*, *NEK1*, *TBK1* and *CFAP410*) compared to all patients with ALS (Extended Data Fig. 3). Although power was limited, this is compatible with a scenario in which the genetic risk of ALS in these patients is a sum of rare variants in ALS genes and other (common) genetic variation.

### Gene prioritization shows locus-specific underlying architectures

To assess whether rare variant associations could drive the common variant signals at the 15 genome-wide significant loci, we combined the common and rare variant analyses to prioritize genes within these loci. The SNP effects on gene expression were assessed by summary-based Mendelian randomization (MR) (SMR) in blood (eQTLGen^23^, *n* = 31,648) and a new brain cortex-derived eQTL dataset (MetaBrain^24^, *n* = 2,970). Finally, we analyzed methylation quantitative trait loci (mQTL) by SMR in blood-derived (*n* = 2,082) and brain-derived (*n* = 522) mQTL datasets^25–27^. Through these multi-layered gene-prioritization strategies, we classified each locus into one of four classes of most likely underlying genetic architecture to prioritize the causal gene (Supplementary Figs. 1–15).

First, in three GWAS loci, the strongest associated SNP was a low-frequency coding variant that was nominated as the causal variant. This was the case for rs80265967 (*SOD1*, p.D90A; Supplementary Fig. 14) and rs113247976 (*KIF5A*, p.P986L; Supplementary Fig. 8), which are coding variants in known ALS-risk genes. This was also the most likely causal mechanism for rs75087725 (*CFAP410*, formerly *C21orf2*, p.V58L; Supplementary Fig. 15), as the GWAS variant is a missense variant; no evidence for other mechanisms including repeat expansions or eQTL or mQTL effects was observed within this locus, and *CFAP410* itself is known to directly interact with *NEK1*, another ALS gene^6,28^. These three loci illustrate the power of large-scale GWASs combined with large imputation panels to directly identify low-frequency causal variants that confer disease risk.

Second, SNPs can tag a highly pathogenic repeat expansion, as was observed for rs2453555 (*C9orf72*) and the known GGGGCC hexanucleotide repeat in this locus (Supplementary Fig. 7). Conditional analysis revealed no residual signal after conditioning on the repeat expansion, which was in LD with the top SNP (*r*^2^ = 0.14, |*D*′| = 0.99, MAF_SNP_ = 0.25, MAF_STR_ = 0.047). Besides the repeat expansion, both eQTL and mQTL analyses point to *C9orf72* (Supplementary Fig. 7). The HEIDI (heterogeneity in dependent instruments) outlier test, however, rejected the null hypothesis that gene expression or methylation mediated the causal effect of the associated SNP (*P*_HEIDI,eQTL_ = 3.7 × 10^−23^ and *P*_HEIDI,mQTL_ = 4.1 × 10^−7^). This is in line with the idea that pathogenic repeat expansion is the causal variant in this locus and that eQTL and mQTL effects do not mediate a causal effect. We found no similar pathogenic repeat expansions that fully explained the SNP association signal in the other genome-wide significant loci.

**Extended Data Fig. 4:**
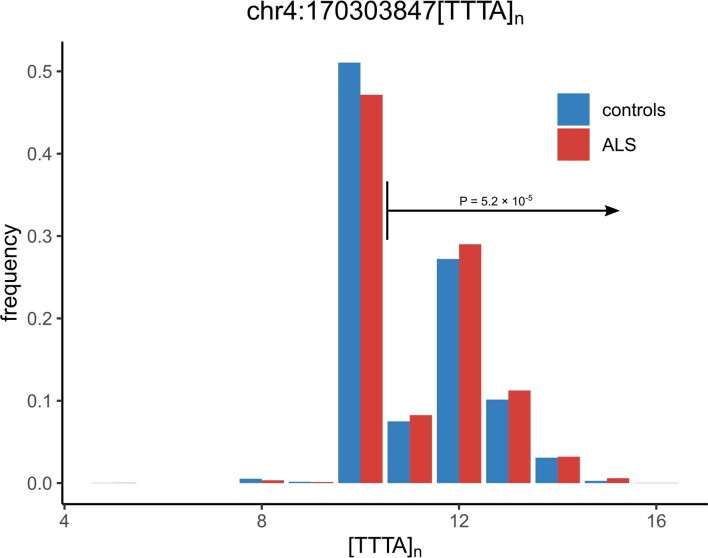
NEK1 repeat distribution. The frequency of STR alleles in ALS cases and controls are shown. A repeat length of 11 and longer was used as the optimal threshold for disease-associated genotype. The *P*-value was calculated by Firth logistic regression and FDR correction over all possible thresholds. Y-axis shows the allele frequency of repeat lengths. Repeat position on GRCh37, and repeat motif are shown. Source data

Third, in two loci (rs62333164 in *NEK1* and rs4075094 in *TBK1*), common and rare variants converged to the same gene, which are known ALS-risk genes^6,8^. For both loci, the rare variant burden association was conditionally independent from the top SNP that was included in the GWAS (Supplementary Figs. 2 and 9). Here, eQTL and mQTL analyses indicated that the risk-increasing effects of the common variants were mediated through both eQTL and mQTL effects on *NEK1* and *TBK1*. Furthermore, a polymorphic STR downstream of *NEK1* was associated with increased ALS risk (motif, TTTA; threshold = 10 repeat units, expanded allele frequency = 0.51, *P* = 5.2 × 10^−5^, false discovery rate (FDR) = 4.7 × 10^−4^; Extended Data Fig. 4). This polymorphic repeat was in LD with the top associated SNP within this locus (*r*^2^ = 0.24, |*D*′| = 0.70). There was no statistically significant association for the top SNP in the WGS data to reliably determine its independent contribution to ALS risk.

Lastly, the fourth group contains seven remaining loci for which there was no direct link to a causal gene through coding variants or repeat expansions. Here, we investigated regulatory effects of the associated SNPs on target genes acting as either eQTL or mQTL. Single genes were prioritized by SMR using both mQTL and eQTL for rs2985994 (*COG3*; Supplementary Fig. 10), rs229243 (*SCFD1*; Supplementary Fig. 11) and rs517339 (*ERGIC1*; Supplementary Fig. 4). In other loci, both methods prioritized multiple genes, such as rs631312 (*MOBP* and *RPSA*; Supplementary Fig. 1) and rs10463311 (*GPX3* and *TNIP1*; Supplementary Fig. 3). Aside from the prioritized genes, each of these loci harbored multiple genes that were not prioritized by any method and are therefore less likely to contribute to ALS risk.

For two loci, no gene was prioritized with these approaches. Within the *UNC13A* locus (rs12608932; Supplementary Fig. 12), recent studies illustrate that the genome-wide significant SNPs act as splicing quantitative trait loci conditional on dysfunction of TAR DNA-binding protein (TDP)-43, resulting in inclusion of a cryptic exon in *UNC13A*^29,30^. Furthermore, we could not prioritize a specific gene in the *HLA* locus (rs9275477; Supplementary Fig. 5).

### Genetic modifiers of ALS disease progression

**Fig. 2:**
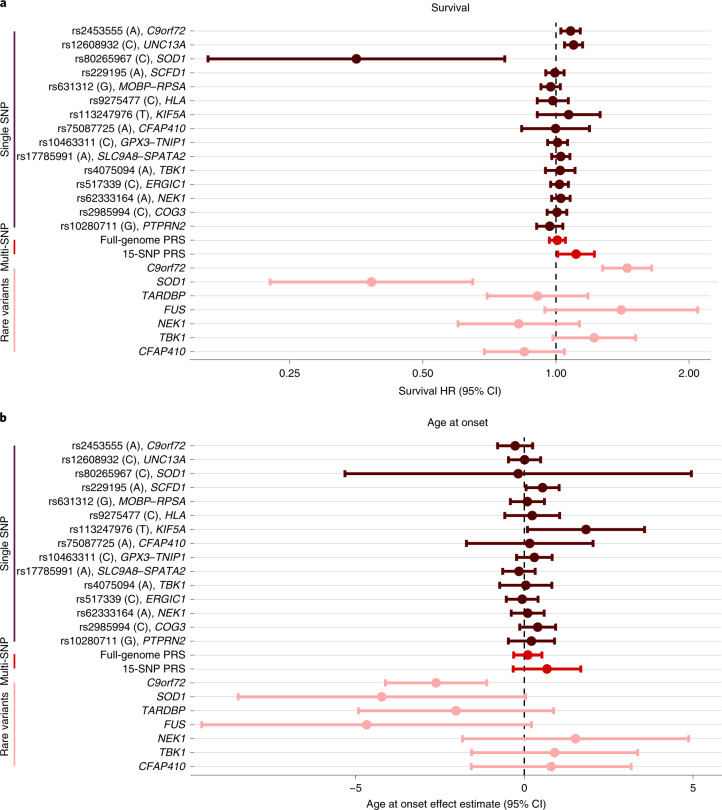
Genetic modifier analyses. **a**, Cox proportional HRs for genome-wide significant SNPs (brown, *n* = 15), PRSs (red, *n* = 2) and rare variant burden in ALS-risk genes (pink, *n* = 7) on survival (months) tested in 6,095 patients with ALS. Estimated HRs are displayed with error bars corresponding to 95% CIs. Higher HRs correspond to shorter survival times. **b**, Effect estimates from a linear regression model of age at onset (years) in 6,095 patients with ALS. Lower effect estimates correspond to a younger age at onset. Effect estimates from linear regression are displayed with error bars corresponding to 95% CIs. The risk-increasing allele for ALS corresponds to the effect allele for both survival and age-at-onset analyses. Source data

We investigated whether genetic risk factors for ALS also act as disease modifiers that affect disease onset and progression. Genotypes for the 15 genome-wide significant SNPs, PRSs and the rare variant burden for *SOD1*, *C9orf72* (repeat expansion status), *TARDBP*, *FUS*, *NEK1*, *TBK1* and *CFAP410* were obtained for all individuals with WGS for whom the complete core clinical data (sex, age at onset, site of onset, survival, time to censoring) were available (*n* = 6,095). Association analyses with survival and age at onset showed that common variants had a limited effect on survival (Fig. 2a) and age at onset (Fig. 2b) but confirmed the association between faster disease progression for the *UNC13A* risk allele (rs12608932, hazard ratio (HR) = 1.10, 95% confidence interval (CI) = 1.05–1.15, *P* = 1.2 × 10^−4^) and slower disease progression in patients with the *SOD1* p.D90A mutation (rs80265967, HR = 0.35, 95% CI = 0.16–0.77, *P* = 8.4 × 10^−4^). This limited effect of common genetic risk factors for ALS susceptibility on disease progression was reflected in the PRS analyses in which we found no effect of the full-genome PRS on survival (HR = 1.02, 95% CI = 0.98–1.06, *P* = 0.28) or age at onset (*b* = 0.10, s.e. = 0.21, *P* = 0.64). Analyses of rare variants confirmed faster disease progression in patients with the *C9orf72* repeat expansion (HR = 1.45, 95% CI = 1.28–1.65, *P* = 1.2 × 10^−8^) with an earlier age at onset (*b* = −2.62, s.e. = 0.77, *P* = 6.4 × 10^−4^).

### Locus-specific sharing of risk loci between ALS and neurodegenerative diseases

**Fig. 3:**
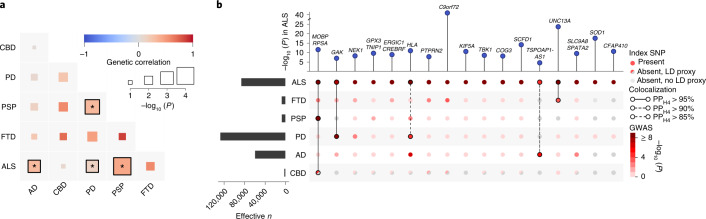
Shared genetic risk between ALS and neurodegenerative diseases. **a**, Genetic correlation analysis. Genetic correlation was estimated with LDSC between each pair of neurodegenerative diseases (ALS, AD, CBD, PD, PSP and FTD). Correlations marked with an asterisk reached nominal statistical significance (*P*_ALS,AD_ = 0.01, *P*_ALS,PD_ = 0.01, *P*_ALS,PSP_ = 0.0001, *P*_PSP,PD_ = 0.002). **b**, SNP associations of ALS lead SNPs or LD proxies in neurodegenerative diseases. The association with ALS is shown at the top. Effective sample size is shown on the left. Posterior probabilities of the same causal SNP affecting two diseases were estimated through colocalization analysis and are highlighted as connections. Source data

**Extended Data Fig. 5:**
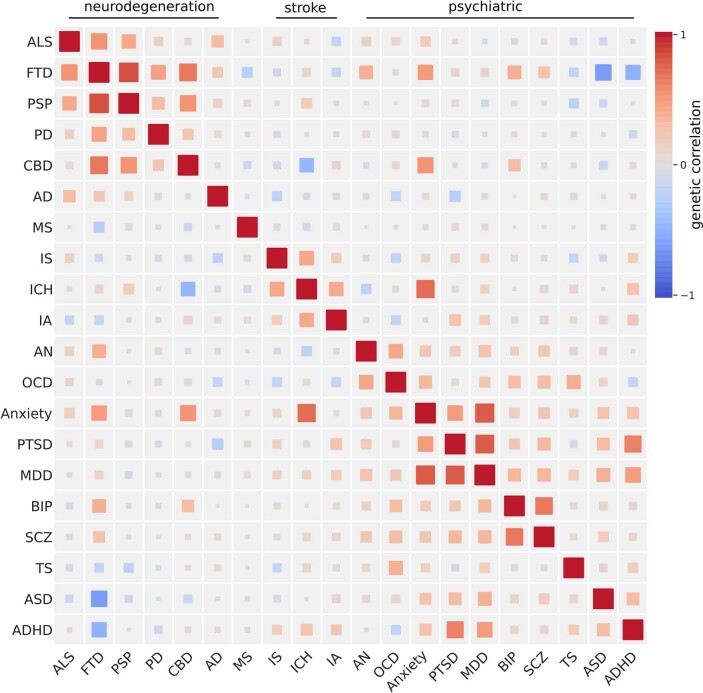
Genetic correlations between brain diseases. Correlation matrix for genetic correlation estimates obtained from bivariate LD score regression. Colors correspond to genetic correlation estimates. Strongest clusters appear between neurodegenerative diseases and within the psychiatric traits. ALS = amyotrophic lateral sclerosis, FTD = frontotemporal dementia, PSP progressive supranuclear palsy, PD = Parkinson’s disease, CBD = corticobasal degeneration, AD = (clinically diagnosed) Alzheimer’s disease, MS = multiple sclerosis, IS = ischemic stroke (any), ICH = intracerebral hemorrhage, IA = intracranial aneurysm (any), AN = anorexia nervosa, OCD = obsessive compulsive disorder, Anxiety = anxiety disorder (score), PTSD = post-traumatic stress disorder, MDD = major depressive disorder, BIP = bipolar disorder, SCZ = schizophrenia, TS = Tourette’s syndrome, ASD = autism spectrum disorder, ADHD = attention-deficit hyperactivity disorder. Source data

To investigate the pleiotropic properties of ALS-associated variants and shared genetic risk with other brain diseases, we estimated genetic correlations between neurodegenerative diseases, psychiatric traits, cerebrovascular diseases and multiple sclerosis (Extended Data Fig. 5). This showed strong genetic correlations among neurodegenerative diseases. Bivariate LDSC confirmed a statistically significant genetic correlation between ALS and PSP (*r*_g_ = 0.44, s.e. = 0.11, *P* = 1.0 × 10^−4^) as previously reported^20^ and also revealed a significant genetic correlation between ALS and AD (*r*_g_ = 0.31, s.e. = 0.12, *P* = 9.6 × 10^−3^) as well as between ALS and PD (*r*_g_ = 0.16, s.e. = 0.061, *P* = 0.011; Fig. 3a). The point estimate for the genetic correlation between ALS and FTD was high (*r*_g_ = 0.59, s.e. = 0.41, *P* = 0.15) but not statistically significant due to the limited size of the FTD GWAS (3,526 cases and 9,402 controls). Thus, power to detect a genetic correlation between ALS and FTD using LDSC was limited.

**Extended Data Fig. 6:**
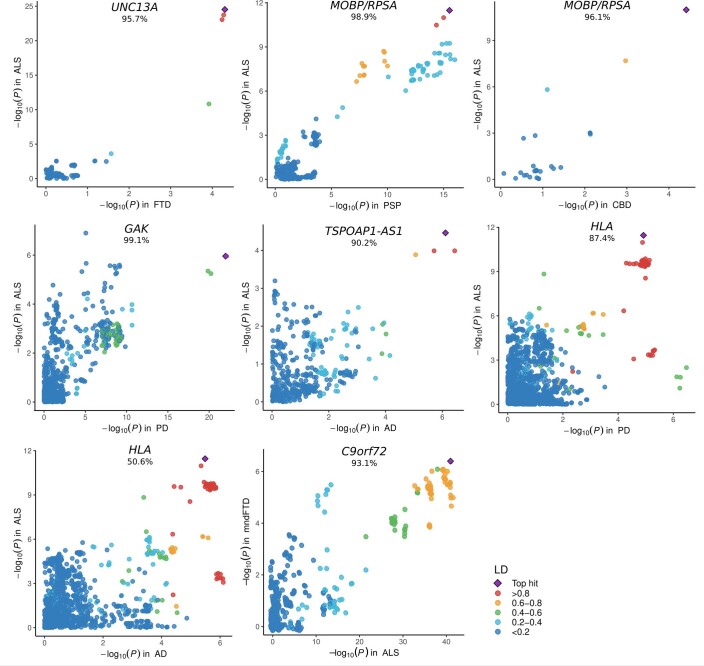
Colocalization signals. Loci were selected for colocalization analysis when the top associated SNP was associated with any neurodegenerative disease at 5 × 10^−5^. For ALS, the European ancestries meta-analysis was used. Bayesian posterior probabilities for a shared variant driving risk of both traits (PP_H4_) are reported below locus names. Colors reflect LD between the variant and top associated SNP. Source data

Patterns of sharing disease-associated genetic variants appeared to be locus specific (Fig. 3b and Supplementary Table 21). To assess whether two traits shared a common signal, indicating shared causal variants, we performed colocalization analyses for all loci meeting *P* < 5 × 10^−5^ in any of the GWASs of neurodegenerative diseases (*n* = 161 loci). This revealed a shared signal in the *MOBP*–*RPSA* locus between ALS, PSP and corticobasal degeneration (CBD) as well as a shared signal in the *UNC13A* locus between ALS and FTD (posterior probability, PP_H4_ > 95%; Extended Data Fig. 6). For the *HLA* locus, there was evidence for a shared causal variant between ALS and PD (PP_H4_ = 88%) but no conclusive evidence for ALS and AD (PP_H4_ = 51% for a shared causal variant and PP_H3_ = 49% for independent signals in both traits).

**Extended Data Fig. 7:**
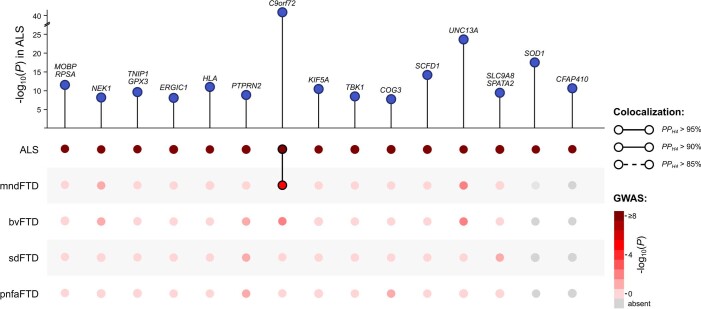
Colocalization analysis with FTD subtypes. Top associated SNPs in the ALS GWAS were selected for colocalization analysis between ALS and FTD subtypes using COLOC. In the top panel, point height is the two-sided -log_10_(*P*-value) of the top-associated SNP in the ALS GWAS. In the bottom panel, association *P*-values of these SNPs with FTD subtypes are shown by color. The Bayesian posterior probability for a shared causal variant between traits (PP_H4_) is depicted by a connection between points. Source data

Furthermore, colocalization analyses identified two additional shared loci that were not genome-wide significant in the ALS GWAS: between ALS and PD at the *GAK* locus (rs34311866, PP_H4_ = 99%) and between ALS and AD at the *TSPOAP1-AS1*locus (rs2632516, PP_H4_ = 90%). Of note, the association at *TSPOAP1-AS1* was not genome-wide significant in the GWAS of clinically diagnosed AD (*P* = 3.7 × 10^−7^) either but was identified in the larger AD-by-proxy GWAS^31^. For FTD subtypes, *C9orf72* showed a colocalization signal for a shared causal variant between ALS and the motor neuron disease subtype of FTD (mndFTD, PP_H4_ = 93%; Extended Data Figs. 6 and 7).

### Enrichment of glutamatergic neurons indicates cell-autonomous processes in ALS susceptibility

**Fig. 4:**
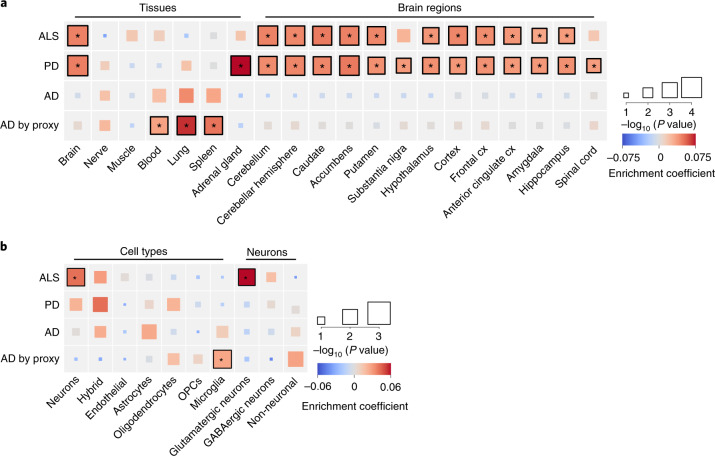
Tissue and cell type enrichment analysis. **a**, Enrichment of tissues and brain regions included in GTEx version 8 illustrates a brain-specific enrichment pattern in ALS, similar to that in PD but contrasting with that in AD. Tissues and brain regions displayed are those significantly enriched in ALS or PD, tissues previously reported in AD and tissues of specific interest for ALS (spinal cord, tibial nerve and muscle). Color represents the enrichment coefficient, and size indicates two-sided −log_10_ (*P*-values) of enrichment obtained by the linear regression model in the MAGMA gene property analysis. **b**, Cell type enrichment analyses indicate neuron-specific enrichment for glutamatergic neurons. In ALS, no enrichment was found for microglia or other non-neuronal cell types, contrasting with the pattern observed in AD. Color represents the enrichment coefficient, and size indicates two-sided −log_10_ (*P*-values) of enrichment obtained by the linear regression model in the MAGMA gene property analysis. Statistically significant enrichments after correction for multiple testing over all tissues (*n* = 54), cell types (*n* = 7) and neurons (*n* = 3) with FDR < 0.05 are marked with an asterisk. Cx, cortex; GABA, γ-aminobutyric acid; OPCs, oligodendrocyte progenitor cells. Source data

**Extended Data Fig. 8:**
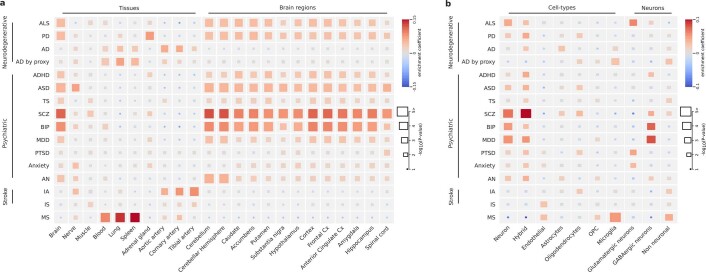
Tissue and cell-type enrichment analyses for all brain diseases. Tissue **(a)** and cell-type **(b)** enrichment for all included brain diseases obtained from two-sided MAGMA linear regression. Only brain diseases with exome-wide significant gene-based MAGMA associations (P < 2.7 × 10^−6^) were suitable for tissue and cell-type enrichment analyses. The color represents enrichment coefficient and size indicates two-sided -log_10_(*P*-value) of enrichment obtained by the linear regression model in the MAGMA gene-property analysis. Due to the large number of significant genes in the gene-based MAGMA analyses for schizophrenia, bipolar disorder and multiple sclerosis the enrichment *P*-values were truncated at P < 1.0 × 10^−5^. ALS = amyotrophic lateral sclerosis, PD = Parkinson’s disease, AD = Alzheimer’s disease, ADHD = attention-deficit hyperactivity disorder, ASD = autism spectrum disorder, TS = Tourette’s syndrome, SCZ = schizophrenia, BIP = bipolar disorder, MDD = major depressive disorder, PTSD = post-traumatic stress disorder, Anxiety = anxiety disorder (score), AN = anorexia nervosa, IA intracranial aneurysm (any), IS = ischemic stroke, MS = multiple sclerosis, Cx = cortex, OPC = oligodendrocyte progenitor cells. Source data

**Extended Data Fig. 9:**
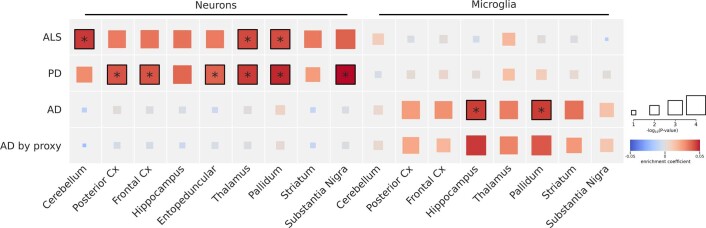
Cell-type enrichment analysis in mice. Cell-type enrichment analysis using the DropViz single-cell RNA sequencing dataset obtained from mice. Similar to the cell-type enrichment analyses there is neuron-specific enrichment in ALS and Parkinson’s disease. In Alzheimer’s disease microglia are the most enriched cell-types. The color represents enrichment coefficient and size indicates two-sided -log_10_(*P*-value) of enrichment obtained by the linear regression model in the MAGMA gene-property analysis. Statistically significant enrichments after correction for multiple testing with a false discovery rate (FDR) < 0.05 are marked with an asterisk. ALS = amyotrophic lateral sclerosis, PD = Parkinson’s disease, AD = Alzheimer’s disease, Cx = cortex. Source data

To find tissues and cell types for which gene expression profiles were enriched for genes within ALS-risk loci, we first combined gene-based association statistics calculated using MAGMA^32^ with gene expression patterns from the Genotype–Tissue Expression (GTEx) project (version 8) in a gene set enrichment analysis using FUMA^33^. We observed a significant enrichment in genes expressed in brain tissues across multiple brain regions but not in peripheral nervous tissue or muscle. Whereas this pattern roughly resembled the enrichments observed in PD and psychiatric traits, it was strikingly different from that reported^31^ and observed in AD in which blood, lung and spleen were mostly enriched, resembling the pattern observed in multiple sclerosis, which is a typical immune-mediated brain disease (Fig. 4a and full results in Supplementary Fig. 16 and Extended Data Fig. 8a). We subsequently queried single-cell RNA-seq datasets of human-derived brain samples to further specify brain-specific enriched cell types using the cell type analysis module in FUMA^34^. This showed significant enrichment for neurons but not for microglia or astrocytes (Fig. 4b). Further subtyping of these neurons illustrated that genes expressed in glutamatergic neurons were mostly enriched for genes within the ALS-associated risk loci. Again, this contrasted with AD, which showed specific enrichment of microglia, similar to multiple sclerosis (Extended Data Fig. 8b). In single-cell RNA-seq data obtained from brain tissues in mice, a similar pattern was observed showing neuron-specific enrichment in ALS and PD but microglia in AD (Extended Data Fig. 9). Together, this indicates that susceptibility to neurodegeneration in ALS is mainly driven by neuron-specific pathology and not by immune-related tissues and microglia.

### Brain-specific coexpression networks improve detection of ALS-relevant pathways

**Extended Data Fig. 10:**
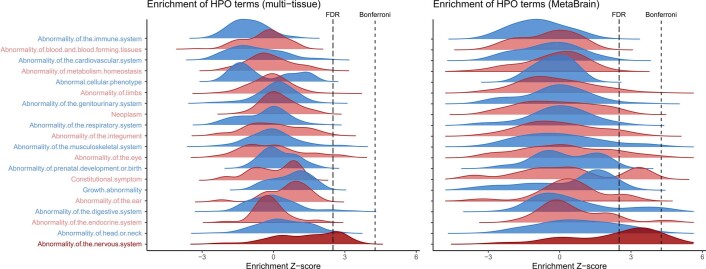
Human phenotype ontology term enrichment. Downstreamer enrichment analyses were performed using the multi-tissue and brain-specific co-expression matrix to identify co-regulated ALS-genes. The distribution of enrichment statistics (Z-scores) for all Human phenotype ontology (HPO) terms are plotted per HPO parent branch. The multi-tissue analysis indicates enrichment for the neurology parent branch ‘*abnormality of the nervous system*’ (dark-red), although no term passes the Bonferroni threshold for multiple testing. The brain-specific analysis illustrates stronger enrichment for the neurology parent branch. In total, 58 HPO terms pass the threshold for multiple testing of which 42 are defined within the ‘*abnormality of the nervous system*’ branch. Source data

To determine which processes were mostly enriched in ALS, we performed enrichment analyses that combined gene-based association statistics with gene coexpression patterns obtained from either multi-tissue transcriptome datasets^35^ or RNA-seq data from brain cortex samples (MetaBrain^24^). To validate this approach, we first tested for enrichment of human phenotype ontology (HPO) terms that are linked to well-established disease genes in the Online Mendelian Inheritance in Man (OMIM) and Orphanet catalogs. Using the multi-tissue coexpression matrix, we found no enriched HPO terms after Bonferroni correction for multiple testing. Using the brain-specific coexpression matrix, however, we found a strong enrichment of HPO terms that are related to ALS or neurodegenerative diseases in general, including ‘cerebral cortical atrophy’ (*P* = 1.8 × 10^−8^), ‘abnormal nervous system electrophysiology’ (*P* = 4.1 × 10^−7^) and ‘distal amyotrophy’ (*P* = 8.6 × 10^−7^; full list in Supplementary Table 22). In general, HPO terms in the neurological branch (‘abnormality of the nervous system’) showed an increase in enrichment statistics in ALS when using the brain-specific coexpression matrix compared to the multi-tissue dataset (Extended Data Fig. 10), which illustrates the benefit of the brain-specific coexpression matrix. Subsequently, we tested for enriched biological processes using reactome and gene ontology terms. Again, using the multi-tissue expression profiles, we found that no reactome annotations were enriched. Leveraging the brain-specific coexpression networks, we identified vesicle-mediated transport (‘membrane trafficking’, *P* = 4.2 × 10^−6^, ‘intra-Golgi and retrograde Golgi-to-endoplasmic reticulum (ER) trafficking’, *P* = 1.4 × 10^−5^) and autophagy (‘macroautophagy’, *P* = 3.2 × 10^−5^) as enriched processes after Bonferroni correction for multiple testing (Supplementary Table 23). The subsequently identified enriched gene ontology terms were all related to vesicle-mediated transport or autophagy (Supplementary Tables 24 and 25).

### MR analyses are in line with a causal relationship between cholesterol levels and ALS

**Fig. 5:**
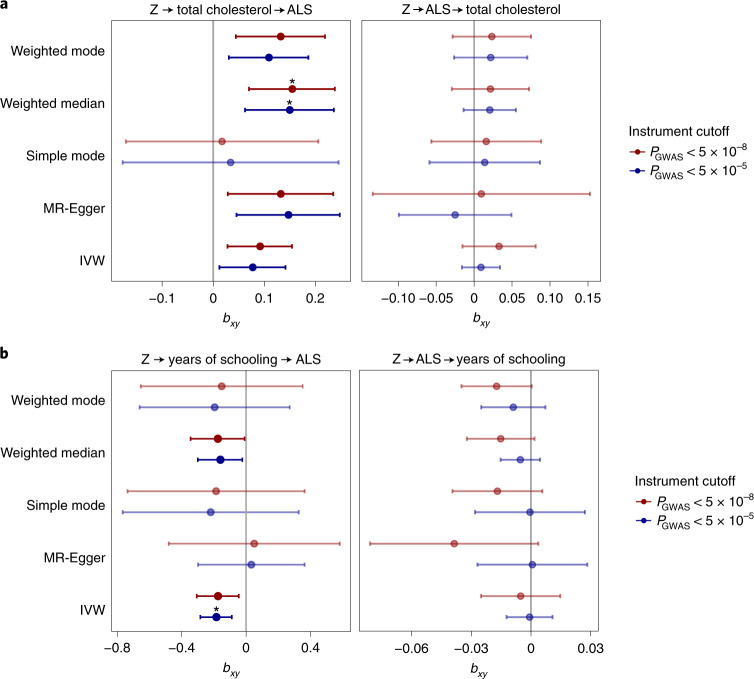
Causal inference of total cholesterol levels and years of schooling in ALS. **a**, MR results for ALS and total cholesterol levels. Results for the five different MR methods for two different *P*-value cutoffs for SNP instrument selection are presented. In total, 83 and 178 SNPs were used as instruments at cutoffs of *P* < 5 × 10^−8^ and *P* < 5 × 10^−5^, respectively. All methods show a consistent positive effect for an increased risk of ALS with higher total cholesterol levels. There is no evidence for reverse causality. Point estimates for MR are presented with error bars reflecting 95% CIs. **b**, MR results for ALS and years of schooling. In total, 306 and 681 SNPs were used as instruments at cutoffs of *P* < 5 × 10^−8^ and *P* < 5 × 10^−5^. Point estimates for MR are presented, with error bars reflecting 95% CIs. Statistically significant effects with a two-sided *P*-value passing Bonferroni correction for multiple testing over all tested traits (*n* = 22), instrument *P*-value cutoffs (*n* = 2) and MR methods (*n* = 5) are marked with an asterisk (total cholesterol, *P*_weighted median_ = 0.0003 and *P*_weighted median_ = 0.0007 for cutoffs at *P* < 5 × 10^−8^ and *P* < 5 × 10^−5^, respectively; years of schooling, *P*_IVW_ = 0.0002 at the cutoff of *P* < 5 × 10^−5^). Here, SNP outliers were not removed for instrument selection. Z, genetic instrument; *b*_*xy*_, estimated causal effect for an increase of 1 s.d. in genetically predicted exposure. Source data

From previous observational case–control studies and our blood-based methylome-wide study^36^, numerous non-genetic risk factors have been implicated in ALS. Here, we studied a selection of those putative risk factors through causal inference in an MR framework^37^. We selected 22 risk factors for which robust genetic predictors were available including body mass index, smoking, alcohol consumption, physical activity, cholesterol-related traits, cardiovascular diseases and inflammatory markers (Supplementary Table 26). These analyses provided the strongest evidence that cholesterol levels were causally related to ALS risk (*b*_weighted median_ = 0.15, s.e. = 0.04, *P* = 3.2 × 10^−4^; Fig. 5a and full results in Supplementary Table 27). These results were robust to removal of outliers through radial MR analysis^38^, and we observed no evidence for reverse causality (Supplementary Tables 28 and 29). Importantly, ascertainment bias can lead to the selection of more highly educated control participants^39^ compared to patients with ALS who are mostly ascertained through the clinic. In line with control participants having higher education, MR analyses indicated a negative effect for years of schooling on ALS risk (inverse-variance-weighted *P*_IVW_ = 2.0 × 10^−4^; Fig. 5b). As a result, years of schooling can act as a confounder for the observed risk-increasing effect of higher total cholesterol levels through ascertainment bias. To correct for this potential confounding, we applied multivariate MR analyses including both years of schooling and total cholesterol levels. The results for total cholesterol were robust in the multivariate analyses, suggesting a causal role for total cholesterol levels on ALS susceptibility (Supplementary Table 30).

## Discussion

In summary, in the largest GWAS on ALS to date including 29,612 patients with ALS and 122,656 control participants, we identified 15 risk loci contributing to ALS risk. Through in-depth analysis of these loci incorporating rare variant burden analyses and repeat expansion screens in WGS data and blood- and brain-specific eQTL and mQTL analyses, we prioritized genes in 13 of the loci. Across the spectrum of neurodegenerative diseases, we identified a genetic correlation between ALS and AD as well as PD and PSP with locus-specific patterns of shared genetic risk across all neurodegenerative diseases. Colocalization analysis identified two additional loci, *GAK* and *TSPOAP1-AS1*, with a high posterior probability of shared causal variants between ALS and PD and between ALS and AD, respectively. We found glutamatergic neurons as the most enriched cell type in the brain, and brain-specific coexpression network enrichment analyses indicated a role for vesicle-mediated transport and autophagy in ALS. Finally, causal inference of previously described risk factors provides evidence for high total cholesterol levels as a causal risk factor for ALS.

The cross-ancestry comparison illustrated similarities in the genetic risk factors for ALS in European and East Asian ancestries, providing an argument for cross-ancestry studies and to further expand ALS GWASs in non-European populations. It is important to note that three loci including those that harbor low-frequency variants (*KIF5A*, *SOD1* and *CFAP410*) were not included in the East Asian GWAS due to their low MAFs. Therefore, the shared genetic risk might not extend to rare genetic variation, for which population-specific frequencies have been observed even within Europe.

The multi-layered gene-prioritization analyses highlighted four different classes of genome-wide significant loci in ALS. First, the sample size of this GWAS combined with accurate imputation of low-frequency variants directly identified rare coding variants that increase ALS risk. These include the known p.D90A mutation in *SOD1* (MAF = 0.006) as well as rare variants in *KIF5A* (MAF = 0.016) and *CFAP410* (MAF = 0.012) for which, after their identification through GWAS, experimental work confirmed their direct role in ALS pathophysiology^11,28,40^. Second, we confirmed that the pathogenic *C9orf72* repeat expansion is tagged by genome-wide significant GWAS SNPs and that no residual signal is left by conditioning the SNP on the repeat expansion. Although more repeat expansions are known to affect ALS risk, we found no similar loci for which the SNPs tag a highly pathogenic repeat expansion. This suggests that highly pathogenic repeat expansions on a stable haplotype are merely the exception rather than the rule in ALS. Third, common and rare variant association signals can converge on the same gene as observed for *NEK1* and *TBK1*, consistent with observations for other traits and diseases^41–43^. We show that these signals are conditionally independent and that the common variants act on the same gene through regulatory effects as eQTL or mQTL. Fourth, we find evidence for regulatory effects of ALS-associated SNPs that act as eQTL or mQTL. These locus-specific architectures illustrate the complexity of ALS-associated GWAS loci for which not one solution fits all, but instead a multi-layered approach to prioritize genes is warranted.

In addition, we find locus-specific patterns of shared effects across neurodegenerative diseases. The *MOBP* locus has previously been identified in PSP and ALS, and here we show that indeed both diseases as well as CBD are likely to share the same causal variant in this locus. The same is true for *UNC13A* and *C9orf72* with FTD and mndFTD, respectively. The colocalization analysis with PD identified a shared causal variant in the *GAK* locus, which was not found in the ALS GWAS alone. Furthermore, the *TSPOAP1-AS1* locus harbors SNPs associated with ALS and AD risk. Although this locus was not significant in either of the GWASs, a larger GWAS including AD-by-proxy cases confirmed this as a risk locus for AD. This illustrates the power of cross-disorder analyses to leverage the shared genetic risk of neurodegenerative diseases.

We aimed to clarify the role of neuron-specific pathology in ALS susceptibility as opposed to non-cell-autonomous pathology through detailed cell type enrichment analyses. Previous experiments have illustrated multiple lines of evidence for non-cell-autonomous pathology in microglia, astrocytes and oligodendrocytes, which ultimately leads to neurodegeneration in ALS^44–46^. These experiments have shown that non-cell-autonomous processes, such as neuroinflammation, mainly act as modifiers of disease in *SOD1* models of ALS^45,46^. Here, we show that genes within loci associated with ALS susceptibility are specifically expressed in (glutamatergic) neurons. This provides evidence for neuron-specific pathology as a driver of ALS susceptibility, which is in stark contrast to the signal of inflammation-associated tissues and cell types in AD and multiple sclerosis. It also shows that disease susceptibility and disease modification can be distinct processes, which is supported by our finding that most genetic susceptibility factors do not have a strong effect on survival. This motivates future large-scale genetic studies on modifiers of ALS progression, as these can be targets for potential new treatments for ALS as well.

The subsequent functional enrichment analyses identified that membrane trafficking, Golgi-to-ER trafficking and autophagy were enriched for genes within ALS-associated loci. These terms and their related gene ontology terms of biological processes are all related to autophagy and degradation of (misfolded) proteins. This corroborates the central hypothesis of impaired protein degradation leading to aberrant protein aggregation in neurons, which is the pathological hallmark of ALS. Our results suggest that this is a central mechanism in ALS even in the absence of rare known mutations in genes directly involved in these biological processes such as *TARDBP*, *FUS*, *UBQLN2* and *OPTN*^47^.

Based on observational studies and MR analyses, conflicting evidence exists for lipid levels including cholesterol as a risk factor for ALS^48–50^. Potential selection bias, reverse causality and the subtype of cholesterol studied challenge the interpretation of these results. Here, we provided support for a causal relationship between high total cholesterol levels and ALS independent of educational attainment and ruling out reverse orientation of the MR effect. The total cholesterol effects were consistent across the different MR methods tested, indicating that this finding is robust to violation of the ‘no horizontal pleiotropy’ assumption. This is in line with our study showing methylation changes associated with increased cholesterol levels in ALS^36^. We do not find a clear pattern for either low-density lipoprotein (LDL) or high-density lipoprotein (HDL) cholesterol subtypes in relation to ALS risk. While cholesterol levels are closely related to cardiovascular risk, the association between cardiovascular risk and ALS risk remains controversial with conflicting reports^3,48,51^. Interestingly, recent work has shown that lipid metabolism and autophagy are closely related^52^, which brings the results of our pathway analyses and MR together. Both in vitro and in vivo experiments have shown that autophagy regulates lipid homeostasis through lipolysis and that impaired autophagy increases triglyceride and cholesterol levels. Conversely, high lipid levels were shown to impair autophagy^52^. Further studies on the effect of high cholesterol levels and protein degradation through autophagy illustrate that high cholesterol levels decrease the fusogenic ability of autophagic vesicles through decreased function of soluble *N*-ethylmaleimide-sensitive factor-attachment protein receptor (SNARE)^53,54^ and lead to increased protein aggregation due to impaired autophagy in mouse models of AD^55^. Therefore, the risk-increasing effect of cholesterol on ALS might be mediated through impaired autophagy.

In conclusion, our GWAS identifies 15 risk loci in ALS and illustrates locus-specific interplay between common and rare genetic variation that helps to prioritize genes for future follow-up studies. We show a causal role for cholesterol, which can be linked to impaired autophagy as common denominators of neuron-specific pathology that drive ALS susceptibility and serve as potential targets for therapeutic strategies.

## Methods

### Genome-wide association study

#### Data description

We obtained individual genotype-level data for all individuals in the previously published GWAS of ALS in European ancestries^11,14^ and publicly available control datasets including 120,971 controls genotyped on Illumina platforms. Additionally, 6,374 cases and 22,526 controls were genotyped on the Illumina OmniExpress and Illumina GSA arrays. Details for each cohort are provided in Supplementary Table 1. All patients with ALS were diagnosed and ascertained through specialized MND clinics where they were diagnosed with ALS according to the (revised) El Escorial Criteria^56^ by neurologists specialized in motor neuron diseases. Whole-blood samples were drawn for DNA isolation, which were specifically collected for ongoing case–control studies of ALS. Both cases with and without a family history for ALS and/or dementia were included. Cases were not pre-screened for specific ALS-related mutations. Given the late onset and relatively low lifetime risk of ALS, controls were not screened for (subclinical) signs of ALS. A detailed description of the ascertainment of newly genotyped cases and controls is provided in the Supplementary Note. All participants gave written informed consent, and the relevant local institutional review boards approved this study (Supplementary Note). Cases and controls formed cohorts when they were processed in the same laboratory and were genotyped in the same batch, resulting in 117 independent cohorts. Summary statistics were obtained for the Asian ancestry GWAS of ALS^15,16^ (Supplementary Note).

#### GWAS quality control and imputation

For each cohort, we first performed individual- and variant-level quality control, after which cohorts were merged into six strata based on genotyping platform. Subsequent stratum-wise quality control was performed, and strata were imputed up to the Haplotype Reference Consortium panel (r.1.1 2016) through the Michigan Imputation Server^21^. Full quality-control details are described in the Supplementary Note and Supplementary Fig. 17. Numbers of individuals and variants passing each quality-control step are described in Supplementary Table 2.

#### Association testing and meta-analysis

After quality control, a null logistic mixed model was fitted using SAIGE^57^ 0.29.1 for each stratum with principal component (PC)1–PC20 as covariates. The model was fit on a set of high-quality (INFO > 0.95) SNPs pruned with PLINK 1.9 (‘–indep-pairwise 50 25 0.1’) in a leave-one-chromosome-out scheme. Subsequently, a SNP-wise logistic mixed model including the saddlepoint approximation test was performed using genotype dosages with SAIGE. Association statistics for all strata were combined in an IVW fixed-effects meta-analysis using METAL^58^.

Genomic inflation factors were calculated per stratum and for the full meta-analysis. To assess any residual confounding due to population stratification and artificial structure in the data, we calculated the LDSC^59^ intercept using SNP LD scores calculated in the HapMap3 CEU population.

#### Cross-ancestry analyses

GWAS summary statistics from two Asian ancestry studies were obtained^15,16^. These summary statistics were meta-analyzed with all European ancestry data in strata as described above. To assess genetic correlation for ALS in European and Asian ancestries, we used Popcorn^60^ version 0.9.9. We used population-specific LD scores for genetic impact and genetic effect provided with the Popcorn software. The regression model (‘–use_regression’) was used to estimate genetic correlation. We calculated both the correlation of genetic effects (correlation of allelic effect sizes) and genetic impact (correlation of allelic effect size adjusted for difference in allele frequencies).

#### Conditional SNP analysis

Conditional and joint SNP analysis (COJO, GCTA version 1.91.1b)^61,62^ was performed to identify potential secondary GWAS signals within a single locus. SNPs with association *P* ≤ 5 × 10^−8^ were considered. Controls of European ancestry from the Health and Retirement Study (HRS, cohort 65, Supplementary Table 1), included in stratum 4 of this study, were used as the LD reference panel.

### Gene prioritization

#### Whole-genome sequencing

##### Sample selection, sequencing and data preparation

Patients with ALS and control participants from Project MinE^63^ were recruited for WGS. The participating cohorts were not pre-screened for ALS-associated mutations and are described in the Supplementary Note. In total, 228 patients were known to have at least one first- or second-degree relative with ALS. A full description of Project MinE and the sequencing and quality-control pipeline were described previously^64^. In summary, the first batch of 2,250 cases and control samples was sequenced on the Illumina HiSeq 2000 platform. All remaining 7,350 case and control samples were sequenced on the Illumina HiSeq X platform. All samples were sequenced to ~35× coverage with 100-bp reads and ~25× coverage with 150-bp reads for HiSeq 2000 and HiSeq X, respectively. Both sequencing sets used PCR-free library preparation. Samples were also genotyped on the Illumina 2.5M array. Sequencing data were then aligned to GRCh37 using the Isaac Aligner, and variants were called using the Isaac variant caller; both the aligner and caller are standard to Illumina’s aligning and calling pipeline. Full details of individual- and variant-level quality control are described in the Supplementary Note.

##### Genic burden association analyses

To aggregate rare variants in a genic burden test framework, we used a variety of variant filters to allow for different genetic architectures of ALS-associated variants per gene as we and others did previously^64,65^. In summary, variants were annotated according to allele-frequency threshold (MAF < 0.01 or MAF < 0.005) and predicted variant impact (‘missense’, ‘damaging’, ‘disruptive’). ‘Disruptive’ variants were those variants classified as frameshift, splice site, exon loss, stop gained, start loss and transcription ablation by SnpEff^66^. ‘Damaging’ variants were missense variants predicted to be damaging by seven prediction algorithms (SIFT^67^, PolyPhen-2 (ref. ^68^), LRT^69^, MutationTaster2 (ref. ^70^), Mutations Assessor^71^ and PROVEAN^72^). ‘Missense’ variants were those missense variants that did not meet the ‘damaging’ criteria. All combinations of allele-frequency threshold and variant annotations were used to test the genic burden on a transcript level in a Firth logistic regression framework in which burden was defined as the number of variants per individual. Sex and the first 20 PCs were included as covariates. All Ensembl protein-coding transcripts for which at least five individuals had a non-zero burden were included in the analysis.

##### Conditional genic burden analysis

We selected for each gene the protein-coding transcripts that were the most strongly associated with ALS across all different combinations of MAF and variant-impact thresholds. For these transcripts and variants, we applied Firth logistic regression on individuals included in both the GWAS and WGS datasets (5,158 cases and 2,167 controls). To assess whether the rare variant burden association and the signal from the GWAS were conditionally independent, we subsequently included the genotype of the top associated SNP within that locus as a covariate.

#### Short tandem repeat screen

For all individuals who had sequencing results in the HiSeq X dataset (5,392 cases, 1,795 controls), we screened all loci harboring SNPs associated with ALS meeting genome-wide significance for expansions of known and new STRs using ExpansionHunter^73^ and ExpansionHunter Denovo^74^.

First, we used ExpansionHunter (version 4.0) to screen for expansions of known STRs located within 1 Mb of the top ALS-associated SNP. For this, we used the STRs identified from indels in 18 high-quality genomes and the GangSTR STR catalog based on STR annotations in the reference genome^75^. We excluded all homopolymers from these catalogs. Repeat length was subsequently regressed on case–control status using Firth logistic regression including the first 20 PCs as covariates, recoding the STR size to a biallelic variant using a sliding window over all observed repeat lengths. To correct for multiple testing across all possible thresholds, we applied Benjamini–Hochberg correction per STR.

To screen for extremely long STR expansions (similar to the *C9orf72* repeat expansion) at loci that were not included in the predefined STR catalogs, we applied ExpansionHunter Denovo^74^. This method aims to only find STR expansions that exceed the sequencing read length (>150 bp) by identifying reads (mapped, mismapped and unmapped) that contain STR motifs, using their mate pairs for de novo mapping to the reference genome.

For all STRs, we calculated LD statistics (*r*^2^ and |*D*′|) between recoded repeat genotypes at the optimal threshold and the top associated GWAS SNP. Subsequently, we conditioned the SNP association on the repeat genotype in a Firth logistic regression.

#### Summary-based Mendelian randomization

We used multi-SNP SMR^76,77^ to infer the effect of gene expression variation on ALS using eQTL (the association of a SNP with expression of a gene) on ALS risk. We chose to apply SMR because this method yielded very similar results when compared to S-PrediXcan^78^ and TWAS^79^ (Supplementary Fig. 18) when applied using GTEx version 7 eQTL, and it can be applied to the large relevant eQTL datasets (MetaBrain and eQTLGen) without access to individual-level genotype and gene expression data. MetaBrain is a harmonized set of 8,727 RNA-seq samples from seven regions of the central nervous system from 15 datasets, and we selected eQTL derived from the cortex region of the brain in samples of European ancestry (MetaBrain Cortex-EUR eQTL, *n* = 2,970 individuals, *n* = 6,601 RNA-seq samples) as our instrument variable^24^. European-only ALS summary statistics were used as the outcome. To supplement this analysis, we also used eQTL in blood from the eQTLGen Consortium, as this is a large available eQTL resource. Samples of European ancestry in the HRS (cohort 65 of this GWAS) were used as the LD reference panel. SNPs with MAF ≥ 1% in the HRS were included. Further SMR settings were left as default, meaning probes with at least one eQTL with *P* ≤ 5 × 10^−8^ were included.

We subsequently performed SMR using DNA mQTL data and European-only ALS summary statistics. Human prefrontal cortex and whole-blood DNA mQTL were generated as part of ongoing analyses by the Complex Disease Epigenomics Group at the University of Exeter (https://www.epigenomicslab.com/) using the Illumina EPIC HumanMethylation array that quantifies DNAm at >850,000 sites across the genome^25^. The prefrontal cortex mQTL dataset was generated using DNA-methylation and SNP data from 522 individuals from the Brains for Dementia Research cohort^26^ and includes 4,623,966 *cis* mQTL (distance between quantitative trait locus SNP and DNAm site ≤500 kb) between 1,744,102 SNPs and 43,337 DNA-methylation sites. The whole-blood mQTL dataset was generated using DNAm and SNP data from 2,082 individuals^80^ and included 30,432,023 *cis* mQTL between 4,030,902 SNPs and 167,854 DNA-methylation sites. mQTL reaching the significance threshold *P* ≤ 1 × 10^−10^ were taken forward for SMR analysis as described by Hannon and colleagues^80^. To map CpG sites to their putative target genes, we used the expression quantitative trait methylation results from a paired methylation and gene expression (RNA-seq) study in blood^81^. For CpG sites where no expression quantitative trait methylation was present in this dataset, we used positional mapping based on the basal regulatory domains and extended regulatory domains as defined in the Genomic Regions Enrichment of Annotations Tool (GREAT)^82^, which is applied in the ‘cpg_to_gene‘ function in the CpGtools toolkit^83^.

### Polygenic risk score calculation

PRSs were constructed based on the 15 lead SNPs of genome-wide significant loci (15-SNP PRS) or a full-genome-wide model (full-genome PRS). For the 15-SNP PRS, the SNP weights were defined as the meta-analyzed effect estimates. We used the summary-BayesR framework from the Genome-wide Complex Trait Bayesian analysis (GCTB) toolkit^84,85^ to obtain SNP weights for the full-genome PRS based on the European ancestry meta-analysis excluding stratum 6. We used the default model parameters and the precalculated sparse LD matrix of imputed HapMap3 SNPs in 50,000 random individuals included in the UK Biobank of European ancestries. Summary-BayesR SNP effects were plotted against marginal SNP effects to rule out potential biased estimates due to non-convergence of the MCMC algorithm. Finally, the PRSs for all individuals in stratum 6 were calculated using the ‘–score’ function in PLINK and normalized to zero mean and unit variance.

### Modifier analyses

For 6,095 of the patients with WGS and ALS, core clinical data were obtained including sex, site of onset (spinal or bulbar), age at onset (years), country of origin and survival, defined as time from disease onset to death, 23 h of continuous non-invasive ventilation per day or tracheostomy. Patients who were still alive were censored at the last date of follow-up.

The genetic risk factors included SNP genotypes, PRSs, *C9orf72* repeat expansion status and the number of rare coding mutations in ALS-risk genes (*SOD1*, *TARDBP*, *FUS*, *NEK1*, *TBK1* and *CFAP410*) as obtained from WGS as described above.

For survival analyses, the Cox proportional hazards mixed model from the ‘coxme‘ package in R was used, modeling country of origin as a random effect. Fixed-effect covariates included sex, age at onset, site of onset, GWAS stratum and PC1–PC5. Violation of the proportional hazards assumption for genotype on survival was assessed by inspecting Schoenfeld residuals. For age-at-onset analyses, we applied linear regression of age at onset on genotype including sex, site of onset, country, GWAS stratum and PC1–PC5 as covariates.

### Cross-trait analyses

#### Datasets and data preparation

GWAS summary statistics for clinically diagnosed AD^86^, PD^87^, FTD^88^, CBD^89^ and PSP^20^ in individuals of European ancestry were obtained. For AD, we used the clinical diagnosis as the case definition to avoid spurious genetic correlations that could have been introduced through the by-proxy design^31^, in which by-proxy cases are defined as having a parent with AD. Although this is a powerful design for gene discovery and the genetic correlation with clinically diagnosed AD is high^90^, mislabeling by-proxy cases when parents suffer from other types of dementia (for example, Lewy body dementia, Parkinson’s dementia, FTD or vascular dementia) can lead to spurious genetic correlations with ALS and other neurodegenerative diseases. For FTD, we primarily used the results of the cross-subtype meta-analysis, which includes behavioral variant FTD, semantic dementia FTD, progressive non-fluent aphasia FTD and mndFTD. For CBD, allele coding was unavailable, and effect alleles were inferred by matching allele frequencies to those observed in the Haplotype Reference Consortium. SNPs with MAF > 0.4 were excluded. Because downstream methods rely on LD scores or population-specific LD patterns, the European ancestry summary statistics from the present study were used for ALS. For sample size parameters, effective sample size was calculated as described previously.

Multiple sclerosis summary statistics were obtained from the International Multiple Sclerosis Genetics Consortium^91^. For cerebrovascular diseases, GWAS summary statistics were obtained for ischemic stroke (any ischemic stroke)^92^, intracerebral hemorrhage^93^ and intracranial aneurysm^94^. For psychiatric traits, GWAS summary statistics were obtained from Psychiatric Genomics Consortium studies on anorexia nervosa^95^, obsessive–compulsive disorder^96^, anxiety disorders (anxiety score)^97^, post-traumatic stress disorder (all European ancestries)^98^, major depressive disorder^99^, bipolar disorder^100^, schizophrenia^101^, Tourette’s syndrome^102^, autism spectrum disorder^103^ and attention-deficit hyperactivity disorder (European ancestries)^104^.

#### Genetic correlation

Genome-wide genetic correlation between neurodegenerative traits was calculated using LDSC (version 1.0.0)^59^. Precomputed LD scores of European individuals in the 1000 Genomes project for high-quality HapMap3 SNPs were used (‘eur_w_ld_chr’). A free intercept was modeled to allow for potential sample overlap.

#### Colocalization

Before the colocalization analysis of neurodegenerative diseases, we first assessed residual confounding by estimating the LDSC intercept using LDSC (version 1.0.0) (ALS, 1.03 (s.e., 0.0073); AD, 1.03 (s.e., 0.013); PD, 0.98 (s.e., 0.0065); PSP, 1.05 (s.e., 0.0076); CBD, 0.98 (s.e., 0.0073); FTD, 1.00 (s.e., 0.0071)), showing limited inflation of test statistics due to confounding across these studies. For each locus (top SNP ±100 kb) harboring SNPs with an association with any of the neurodegenerative diseases (ALS, AD, PD, PSP, CBD, FTD) at *P* < 1 × 10^−5^, we performed colocalization analysis using the ‘coloc’ package in R^105^. We set the prior probabilities to *π*_1_ = 1 × 10^−4^, *π*_2_ = 1 × 10^−4^ and *π*_12_ = 1 × 10^−5^ for a causal variant in trait 1 or trait 2 and a shared causal variant between traits 1 and 2, respectively. Using the same parameters, we performed colocalization analysis for ALS and each of the FTD subtypes (behavioral variant FTD, semantic dementia FTD, progressive non-fluent aphasia FTD and mndFTD).

### Enrichment analyses

#### Linkage disequilibrium score regression annotation-specific enrichment analysis

We used LDSC (version 1.0.0)^59^ to calculate SNP-based heritability, the LDSC intercept and SNP-based heritability enrichment for partitions of the genome. In all LDSC analyses, summary statistics excluding the *HLA* region of only samples of European ancestry were included. LD scores and partitioned LD scores provided by LDSC were used for genome-wide and genic region-based heritability analyses. The option ‘–overlap-annot’ was used in the partitioned heritability analysis to allow for overlapping SNPs between MAF bins. SNPs with MAF > 5% were included.

#### Tissue and cell type enrichment analysis

Tissue and cell type enrichment analyses were performed using the GWAS summary statistics of the European ancestry meta-analysis and FUMA^33^ software version 1.3.6a. FUMA performs a genic aggregation analysis of GWAS association signals to calculate gene-wise association signals using MAGMA version 1.6 and subsequently tests whether tissues and cell types are enriched for expression of these genes. For tissue enrichment analysis, we used the GTEx version 8 reference set. FDR-corrected *P*-values <0.05 across all tissues (*n* = 54) were considered statistically significant. For cell type enrichment analyses^34^, we used human-derived single-cell RNA-seq data on major brain cell types (GSE67835 without fetal samples^106^), Allen Brain Atlas cell types^107^ for the human-derived major neuronal subtypes and the DropViz^108^ dataset for mouse-derived brain cell types across all brain regions. We applied FDR correction for multiple testing within each expression dataset, and FDR-corrected *P-*values <0.05 were considered statistically significant.

#### Pathway enrichment analysis

We used Downstreamer software^24^ to identify enriched biological pathways and processes. First, gene-based association statistics were obtained with the Pascal method^109^, which aggregates SNP association statistics including SNPs up to 10 kb upstream and downstream of a gene, accounting for LD using the non-Finnish European individuals from the 1000 Genomes Project phase 3 (ref. ^110^) as a reference. In the Downstreamer method, putative core genes are defined as those that are coexpressed with disease-associated genes and can therefore be implicated in disease. Coexpression networks are based on either a large, multi-tissue transcriptome dataset including 56,435 genes and 31,499 individuals or brain-specific RNA-seq data obtained from the MetaBrain resource. The gene-based association statistics, coexpression matrix and gene *Z* scores per pathway or HPO term are then combined in a generalized least-squares regression model to obtain enrichment statistics^24^. Enrichment analyses were performed for reactome, gene ontology and HPO terms using multi-tissue or brain-specific transcriptome datasets to calculate the coexpression matrix.

The distribution of enrichment *Z*-score statistics was compared between analyses using multi-tissue or brain-specific coexpression matrices. Using the ‘pyhpo’ module in Python, all HPO terms were assigned to their parent term(s) in the ‘phenotypic abnormality’ (HP:0000118) branch, which includes phenotypic abnormalities grouped per organ system.

### Mendelian randomization

Causal inference through MR analysis was performed for 22 exposures for which large-scale GWASs are available and for which there is prior evidence for an association with ALS. These include seven behavioral-related traits: body mass index (anthropometric)^111^, years of schooling (educational attainment)^112^, alcoholic drinks per week, age of smoking initiation and cigarettes per day from Liu et al.^113^, days per week of moderate physical activity and days per week of vigorous activity from the UK Biobank^114^; four blood pressure traits (coronary artery disease^115^, stroke^92^, diastolic blood pressure and systolic blood pressure^116^); seven immune system traits from Vuckovic et al.^117^ (basophil, eosinophil, lymphocyte, monocyte, neutrophil and white blood cell counts) and C-reactive protein^118^; and four lipid traits from Willer et al.^119^ (HDL cholesterol, LDL cholesterol, total cholesterol and triglyceride levels). A full description of the included studies is provided in Supplementary Table 26. From these GWASs, SNPs to serve as instruments for MR analyses were selected at two different *P*-value cutoffs (*P* < 5 × 10^−8^ and *P* < 5 × 10^−5^) and then LD clumped to obtain independent SNPs. SNP effect estimates on ALS risk were obtained from the European ancestry-only GWAS and, if needed, an LD proxy was selected (*r*^2^ > 0.8).

After harmonizing effect alleles and excluding palindromic SNPs, we performed a series of quality-control steps to avoid biased estimates of causal effects, checking for each exposure (1) instrument coverage (>85% overlapping SNPs; Supplementary Table 31), (2) instrument strength (*F*-statistic^37,120,121^ >10; Supplementary Table 32), (3) distribution and significance of the Wald ratios (visual inspection of volcano plots; Supplementary Table 33) and (4) heterogeneity across the instrument-exposure effects (*Q*-statistic at *P* < 0.05 indicated heterogeneity; Supplementary Table 34).

We applied five different MR methods: IVW using the random-effects model, MR-Egger and simple mode, weighted median and weighted mode methods. When only a single SNP was available, the Wald ratio test was conducted. MR analysis was conducted in R using the ‘mr()‘ function in the ‘TwoSampleMR‘ package^122^.

Subsequently, radial MR analysis was conducted to determine whether Wald ratio outliers needed to be removed from the IVW or MR-Egger MR estimates^38^. In addition, we conducted a Q-test to identify outlier SNPs (*P* < 0.05). These outliers were then removed from the original MR analyses (across all five MR methods). The radial MR analysis was conducted using the RadialMR R package (https://github.com/WSpiller/RadialMR). To determine whether MR effects were orientated in the correct direction (from exposure to ALS), we conducted both reverse MR^123^ and Steiger filtering^124^ on our top MR findings.

Finally, we explored whether the MR effects of our total and LDL cholesterol and systolic blood pressure exposures may be confounded by the effect we observed for years of schooling by conducting multivariate MR analysis^125^. Conditional *F*- and *Q*-statistics were calculated using the ‘MVMR‘ package^126^ in R.

### Statistical analyses

All presented *P*-values correspond to two-sided *P*-values uncorrected for multiple testing unless explicitly stated otherwise.

### Reporting Summary

Further information on research design is available in the Nature Research Reporting Summary linked to this article.

## Online content

Any methods, additional references, Nature Research reporting summaries, source data, extended data, supplementary information, acknowledgements, peer review information; details of author contributions and competing interests; and statements of data and code availability are available at 10.1038/s41588-021-00973-1.

## Supplementary information

Supplementary Information Supplementary Note, Figs. 1–18 and Tables 4–25 and 27–30

Reporting Summary

Peer Review Information

Supplementary Tables Supplementary Tables 1–3, 26 and 31–34.

## Data Availability

The GWAS summary statistics generated in this study are publicly available in the NHGRI-EBI GWAS Catalog at https://www.ebi.ac.uk/gwas/ (accession IDs GCST90027163 and GCST90027164 for cross-ancestry and European ancestry meta-analyses, respectively) and through the Project MinE website (https://www.projectmine.com/research/download-data/). Summary statistics of the rare variant burden analyses and eQTL and mQTL SMR analyses are available through the Project MinE website. The following publicly available datasets were used in this project: the Wellcome Trust Case Control Consortium (https://www.wtccc.org.uk/) and dbGaP datasets (phs000101.v3.p1, NIH Genome-Wide Association Studies of Amyotrophic Lateral Sclerosis; phs000126.v1.p1, CIDR: Genome Wide Association Study in Familial Parkinson Disease (PD); phs000196.v1.p1, Genome-Wide Association Study of Parkinson Disease: Genes and Environment; phs000344.v1.p1, Genome-Wide Association Study of Amyotrophic Lateral Sclerosis in Finland; phs000336, a Genome-Wide Association Study of Lung Cancer Risk; phs000346, Genome-Wide Association Study for Bladder Cancer Risk; phs000789, Collaborative Study of Genes, Nutrients and Metabolites (CSGNM); phs000206, Whole Genome Scan for Pancreatic Cancer Risk in the Pancreatic Cancer Cohort Consortium and Pancreatic Cancer Case–Control Consortium (PanScan); phs000297, eMERGE Network Study of the Genetic Determinants of Resistant Hypertension; phs000652, Cohort-Based Genome-Wide Association Study of Glioma (GliomaScan); phs000869, Barrett’s and Esophageal Adenocarcinoma Genetic Susceptibility Study (BEAGESS); phs000812, the Breast and Prostate Cancer Cohort Consortium (BPC3) GWAS of Aggressive Prostate Cancer and ER^−^ Breast Cancer; phs000428, Genetics Resource with the HRS; phs000360.v3, eMERGE Network Genome-Wide Association Study of Red Cell Indices, White Blood Count (WBC) Differential, Diabetic Retinopathy, Height, Serum Lipid Levels, Specifically Total Cholesterol, HDL (High Density Lipoprotein), LDL (Low Density Lipoprotein), and Triglycerides, and Autoimmune Hypothyroidism; phs000893.v1, Genome-Wide Association Study of Endometrial Cancer in the Epidemiology of Endometrial Cancer Consortium (E2C2); phs000168.v2, National Institute on Aging—Late Onset Alzheimer’s Disease Family Study: Genome-Wide Association Study for Susceptibility Loci; phs000092.v1, Study of Addiction: Genetics and Environment (SAGE); phs000864.v1, Genomic Predictors of Combat Stress Vulnerability and Resilience; phs000170.v2, a Genome-Wide Association Study on Cataract and HDL in the Personalized Medicine Research Project Cohort; phs000431.v2, IgA Nephropathy GWAS on Individuals of European Ancestry (IGANGWAS2); phs000237.v1, Northwestern NUgene Project: Type 2 Diabetes; phs000169.v1, Whole Genome Association Study of Visceral Adiposity in the Health Aging and Body Composition (Health ABC) Study; phs000982.v1, Genetic Analysis of Psoriasis and Psoriatic Arthritis: GWAS of Psoriatic Arthritis; phs000289.v2, National Human Genome Research Institute (NHGRI) GENEVA Genome-Wide Association Study of Venous Thrombosis (GWAS of VTE); phs000634.v1, National Cancer Institute (NCI) Genome Wide Association Study (GWAS) of Lung Cancer in Never Smokers; phs000274.v1, Genome-Wide Association Study of Celiac Disease; phs001172.v1, National Institute of Neurological Disorders and Stroke (NINDS) Parkinson’s Disease; phs000389.v1, GEnetics of Nephropathy—an International Effort (GENIE) GWAS of Diabetic Nephropathy in the UK GoKinD and All-Ireland Cohorts; phs000460.v1, Genetics of 24 Hour Urine Composition; phs000138.v2, GWAS for Genetic Determinants of Bone Fragility in European–American Premenopausal Women; phs000394.v1, Autopsy-Confirmed Parkinson Disease GWAS Consortium (APDGC); phs000948.v1, Genetic Discovery and Application in a Clinical Setting: Continuing a Partnership (eMERGE Phase II); phs000630.v1, Exome Chip Study of NIMH Controls; phs000678.v1, a Family-Based Study of Genes and Environment in Young-Onset Breast Cancer; phs000351.v1, National Cancer Institute Genome-Wide Association Study of Renal Cell Carcinoma; phs000314.v1, Genetic Associations in Idiopathic Talipes Equinovarus (Clubfoot)—GAIT; phs000147.v3, Cancer Genetic Markers of Susceptibility (CGEMS) Breast Cancer Genome-wide Association Study (GWAS)—Primary Scan: Nurses’ Health Study—Additional Cases: Nurses’ Health Study 2; phs000882.v1, National Cancer Institute (NCI) Prostate Cancer Genome-Wide Association Study for Uncommon Susceptibility Loci (PEGASUS); phs000238.v1, National Eye Institute Glaucoma Human Genetics Collaboration (NEIGHBOR) Consortium Glaucoma Genome-Wide Association Study; phs000397.v1, National Institute on Aging (NIA) Long Life Family Study (LLFS); phs000421.v1, a Genome-Wide Association Study of Fuchs’ Endothelial Corneal Dystrophy (FECD); phs000142.v1, a Whole Genome Association Scan for Myopia and Glaucoma Endophenotypes using Twin Studies; phs000303.v1, Genetic Epidemiology of Refractive Error in the KORA (Kooperative Gesundheitsforschung in der Region Augsburg) Study; phs000125.v1, CIDR: Collaborative Study on the Genetics of Alcoholism Case Control Study; phs001039.v1, International Age-Related Macular Degeneration Genomics Consortium—Exome Chip Experiment; phs000187.v1, High Density SNP Association Analysis of Melanoma: Case–Control and Outcomes Investigation; phs000101.v5, Genome-Wide Association Study of Amyotrophic Lateral Sclerosis; phs002068.v1.p1, Sporadic ALS Australia Systems Genomics Consortium (SALSA-SGC)). Source data are provided with this paper.

## Source data

Source Data Fig. 1 Link to publicly available GWAS summary statistics.
Source Data Fig. 2 Results of modifier analyses, effect estimates, 95% CIs and *P* values for the Cox proportional hazards mixed model (survival) and linear regression (age at onset).
Source Data Fig. 3 Bivariate LDSC results for genetic correlation estimates among neurodegenerative diseases and association statistics for genome-wide significant SNPs and two colocalization loci in neurodegenerative diseases.
Source Data Fig. 4 MAGMA enrichment statistics for GTEx tissues and cell types in ALS, PD, AD and AD by proxy.
Source Data Fig. 5 Pointer to Supplementary Table 27.
Source Data Extended Data Fig. 1 Link to publicly available GWAS summary statistics.
Source Data Extended Data Fig. 2 Results of the stratified LDSC enrichment analysis.
Source Data Extended Data Fig. 3 PRSs stratified by phenotype or rare variant burden status.
Source Data Extended Data Fig. 4 *NEK1* STR alleles for patients with ALS and controls.
Source Data Extended Data Fig. 5 LDSC genetic correlation estimates for brain diseases.
Source Data Extended Data Fig. 6 Association statistics for SNPs in loci with colocalization signals.
Source Data Extended Data Fig. 7 Association statistics for genome-wide significant SNPs in ALS and FTD subtypes.
Source Data Extended Data Fig. 8 MAGMA enrichment statistics for GTEx tissues and cell types across all brain diseases.
Source Data Extended Data Fig. 9 MAGMA enrichment statistics for DropViz cell types across ALS, PD, AD and AD by proxy.
Source Data Extended Data Fig. 10 Downstreamer HPO term enrichment statistics using multi-tissue and MetaBrain coexpression matrices.

## Code availability

The following software packages were used for data analyses: R version 3.6.3 with additional packages tidyverse version 1.3.0, data.table version 1.14.0, ggplot2 version 3.3.3, MASS version 7.3.53, SNPRelate version 1.26.0, logistf version 1.24, coloc version 5.1.0, twoSampleMR version 0.5.6, RadialMR version 1.0, MVMR version 0.3, survival version 3.1.8, coxme version 2.2.16 and survminer version 0.4.9 (https://www.r-project.org/), Python version 3.7 with additional modules pandas version 1.1.3, numpy version 1.18.1, scipy version 1.4.1, CpGtools version 1.0.9, matplotlib version 3.1.3, pyliftover version 0.4 and pyhpo version 2.5.0 (https://anaconda.org/), GenomeStudio version 2.0 (https://emea.illumina.com/techniques/microarrays/array-data-analysis-experimental-design/genomestudio.html), GCTA version 1.93.2beta (https://cnsgenomics.com/software/gcta/#Overview), EIGENSOFT version 6.1.4 (https://github.com/DreichLab/EIG), SNPTEST version 2.5.4-beta3 (https://www.well.ox.ac.uk/~gav/snptest/), PLINK version 1.9 (http://www.cog-genomics.org/plink2), the Michigan Imputation Server (https://imputationserver.sph.umich.edu), EAGLE version 2.3 through the Michigan Imputation Server (https://imputationserver.sph.umich.edu), SAIGE version 0.29.1 (https://github.com/weizhouUMICH/SAIGE), METAL 2011-03-25 (https://genome.sph.umich.edu/wiki/METAL), SnpSift 4.3p (https://pcingola.github.io/SnpEff), ANNOVAR version 2017-07-17 for LRT, Polyphen-2, MutationTaster2, Mutation Assessor, PROVEAN and SIFT (https://annovar.openbioinformatics.org/), Polyphen-2 (http://genetics.bwh.harvard.edu/pph2/), MutationTaster2 (http://www.mutationtaster.org/), Mutation Assessor release 3 (http://mutationassessor.org/r3/), PROVEAN version 1.1 (http://provean.jcvi.org/index.php), SIFT version 6.2.1 (https://sift.bii.a-star.edu.sg/), SnpEff 4.3p (https://pcingola.github.io/SnpEff), LDSC version 1.0.1 (https://github.com/bulik/ldsc), ExpansionHunter version 4 (https://github.com/Illumina/ExpansionHunter), ExpansionHunter Denovo (https://github.com/Illumina/ExpansionHunterDenovo), SMR (https://cnsgenomics.com/software/smr/), MAGMA version 1.6 (https://ctg.cncr.nl/software/magma), FUMA (https://fuma.ctglab.nl/), FUMA Cell-type (https://fuma.ctglab.nl/celltype), summary-BayesR (https://cnsgenomics.com/software/gctb/#SummaryBayesianAlphabet), S-PrediXcan (https://github.com/hakyimlab/MetaXcan) and TWAS (http://gusevlab.org/projects/fusion/).
