# Author Correction: Common and rare variant association analyses in amyotrophic lateral sclerosis identify 15 risk loci with distinct genetic architectures and neuron-specific biology

**DOI:** 10.1038/s41588-022-01020-3

**Published:** 2022-01-31

**Authors:** Wouter van Rheenen, Rick A. A. van der Spek, Mark K. Bakker, Joke J. F. A. van Vugt, Paul J. Hop, Ramona A. J. Zwamborn, Niek de Klein, Harm-Jan Westra, Olivier B. Bakker, Patrick Deelen, Gemma Shireby, Eilis Hannon, Matthieu Moisse, Denis Baird, Restuadi Restuadi, Egor Dolzhenko, Annelot M. Dekker, Klara Gawor, Henk-Jan Westeneng, Gijs H. P. Tazelaar, Kristel R. van Eijk, Maarten Kooyman, Ross P. Byrne, Mark Doherty, Mark Heverin, Ahmad Al Khleifat, Alfredo Iacoangeli, Aleksey Shatunov, Nicola Ticozzi, Johnathan Cooper-Knock, Bradley N. Smith, Marta Gromicho, Siddharthan Chandran, Suvankar Pal, Karen E. Morrison, Pamela J. Shaw, John Hardy, Richard W. Orrell, Michael Sendtner, Thomas Meyer, Nazli Başak, Anneke J. van der Kooi, Antonia Ratti, Isabella Fogh, Cinzia Gellera, Giuseppe Lauria, Stefania Corti, Cristina Cereda, Daisy Sproviero, Sandra D’Alfonso, Gianni Sorarù, Gabriele Siciliano, Massimiliano Filosto, Alessandro Padovani, Adriano Chiò, Andrea Calvo, Cristina Moglia, Maura Brunetti, Antonio Canosa, Maurizio Grassano, Ettore Beghi, Elisabetta Pupillo, Giancarlo Logroscino, Beatrice Nefussy, Alma Osmanovic, Angelica Nordin, Yossef Lerner, Michal Zabari, Marc Gotkine, Robert H. Baloh, Shaughn Bell, Patrick Vourc’h, Philippe Corcia, Philippe Couratier, Stéphanie Millecamps, Vincent Meininger, François Salachas, Jesus S. Mora Pardina, Abdelilah Assialioui, Ricardo Rojas-García, Patrick A. Dion, Jay P. Ross, Albert C. Ludolph, Jochen H. Weishaupt, David Brenner, Axel Freischmidt, Gilbert Bensimon, Alexis Brice, Alexandra Durr, Christine A. M. Payan, Safa Saker-Delye, Nicholas W. Wood, Simon Topp, Rosa Rademakers, Lukas Tittmann, Wolfgang Lieb, Andre Franke, Stephan Ripke, Alice Braun, Julia Kraft, David C. Whiteman, Catherine M. Olsen, Andre G. Uitterlinden, Albert Hofman, Marcella Rietschel, Sven Cichon, Markus M. Nöthen, Philippe Amouyel, SLALOM Consortium, Giancarlo Comi, Nilo Riva, Christian Lunetta, Francesca Gerardi, Maria Sofia Cotelli, Fabrizio Rinaldi, Luca Chiveri, Maria Cristina Guaita, Patrizia Perrone, Mauro Ceroni, Luca Diamanti, Carlo Ferrarese, Lucio Tremolizzo, Maria Luisa Delodovici, Giorgio Bono, PARALS Consortium, Antonio Canosa, Umberto Manera, Rosario Vasta, Alessandro Bombaci, Federico Casale, Giuseppe Fuda, Paolina Salamone, Barbara Iazzolino, Laura Peotta, Paolo Cugnasco, Giovanni De Marco, Maria Claudia Torrieri, Francesca Palumbo, Salvatore Gallone, Marco Barberis, Luca Sbaiz, Salvatore Gentile, Alessandro Mauro, Letizia Mazzini, Fabiola De Marchi, Lucia Corrado, Sandra D’Alfonso, Antonio Bertolotto, Maurizio Gionco, Daniela Leotta, Enrico Odddenino, Daniele Imperiale, Roberto Cavallo, Pietro Pignatta, Marco De Mattei, Claudio Geda, Diego Maria Papurello, Graziano Gusmaroli, Cristoforo Comi, Carmelo Labate, Luigi Ruiz, Delfina Ferrandi, Eugenia Rota, Marco Aguggia, Nicoletta Di Vito, Piero Meineri, Paolo Ghiglione, Nicola Launaro, Michele Dotta, Alessia Di Sapio, Guido Giardini, SLAGEN Consortium, Cinzia Tiloca, Silvia Peverelli, Franco Taroni, Viviana Pensato, Barbara Castellotti, Giacomo P. Comi, Roberto Del Bo, Mauro Ceroni, Stella Gagliardi, Lucia Corrado, Letizia Mazzini, Flavia Raggi, Costanza Simoncini, Annalisa Lo Gerfo, Maurizio Inghilleri, Alessandra Ferlini, SLAP Consortium, Isabella L. Simone, Bruno Passarella, Vito Guerra, Stefano Zoccolella, Cecilia Nozzoli, Ciro Mundi, Maurizio Leone, Michele Zarrelli, Filippo Tamma, Francesco Valluzzi, Gianluigi Calabrese, Giovanni Boero, Augusto Rini, Bryan J. Traynor, Andrew B. Singleton, Miguel Mitne Neto, Ruben J. Cauchi, Roel A. Ophoff, Martina Wiedau-Pazos, Catherine Lomen-Hoerth, Vivianna M. van Deerlin, Julian Grosskreutz, Annekathrin Roediger, Nayana Gaur, Alexander Jörk, Tabea Barthel, Erik Theele, Benjamin Ilse, Beatrice Stubendorff, Otto W. Witte, Robert Steinbach, Christian A. Hübner, Caroline Graff, Lev Brylev, Vera Fominykh, Vera Demeshonok, Anastasia Ataulina, Boris Rogelj, Blaž Koritnik, Janez Zidar, Metka Ravnik-Glavač, Damjan Glavač, Zorica Stević, Vivian Drory, Monica Povedano, Ian P. Blair, Matthew C. Kiernan, Beben Benyamin, Robert D. Henderson, Sarah Furlong, Susan Mathers, Pamela A. McCombe, Merrilee Needham, Shyuan T. Ngo, Garth A. Nicholson, Roger Pamphlett, Dominic B. Rowe, Frederik J. Steyn, Kelly L. Williams, Karen A. Mather, Perminder S. Sachdev, Anjali K. Henders, Leanne Wallace, Mamede de Carvalho, Susana Pinto, Susanne Petri, Markus Weber, Guy A. Rouleau, Vincenzo Silani, Charles J. Curtis, Gerome Breen, Jonathan D. Glass, Robert H. Brown, John E. Landers, Christopher E. Shaw, Peter M. Andersen, Ewout J. N. Groen, Michael A. van Es, R. Jeroen Pasterkamp, Dongsheng Fan, Fleur C. Garton, Allan F. McRae, George Davey Smith, Tom R. Gaunt, Michael A. Eberle, Jonathan Mill, Russell L. McLaughlin, Orla Hardiman, Kevin P. Kenna, Naomi R. Wray, Ellen Tsai, Heiko Runz, Lude Franke, Ammar Al-Chalabi, Philip Van Damme, Leonard H. van den Berg, Jan H. Veldink

**Affiliations:** 1 Department of Neurology, UMC Utrecht Brain Center, University Medical Center Utrecht, Utrecht University, Utrecht, the Netherlands; 2 Department of Genetics, University of Groningen, University Medical Centre Groningen, Groningen, the Netherlands; 3 Department of Genetics, University Medical Center Utrecht, Utrecht University, Utrecht, the Netherlands; 4 University of Exeter Medical School, College of Medicine and Health, University of Exeter, Exeter, UK; 5 Department of Neurosciences, Experimental Neurology and Leuven Brain Institute (LBI), KU Leuven—University of Leuven, Leuven, Belgium; 6 Laboratory of Neurobiology, VIB, Center for Brain & Disease Research, Leuven, Belgium; 7 Department of Neurology, University Hospitals Leuven, Leuven, Belgium; 8 Translational Biology, Biogen, Boston, MA USA; 9 MRC Integrative Epidemiology Unit (IEU), Population Health Sciences, University of Bristol, Bristol, UK; 10 Institute for Molecular Bioscience, University of Queensland, Brisbane, Queensland Australia; 11 Illumina, San Diego, CA USA; 12 Complex Trait Genomics Laboratory, Smurfit Institute of Genetics, Trinity College Dublin, Dublin, Ireland; 13 Academic Unit of Neurology, Trinity Biomedical Sciences Institute, Trinity College Dublin, Dublin, Ireland; 14 Maurice Wohl Clinical Neuroscience Institute, Department of Basic and Clinical Neuroscience, Institute of Psychiatry, Psychology and Neuroscience, King’s College London, London, UK; 15 Department of Biostatistics and Health Informatics, Institute of Psychiatry, Psychology and Neuroscience, King’s College London, London, UK; 16 National Institute for Health Research Biomedical Research Centre and Dementia Unit, South London and Maudsley NHS Foundation Trust and King’s College London, London, UK; 17 Department of Neurology, Stroke Unit and Laboratory of Neuroscience, Istituto Auxologico Italiano IRCCS, Milan, Italy; 18 Department of Pathophysiology and Transplantation, ‘Dino Ferrari’ Center, Università degli Studi di Milano, Milan, Italy; 19 Sheffield Institute for Translational Neuroscience (SITraN), University of Sheffield, Sheffield, UK; 20 Instituto de Fisiologia, Instituto de Medicina Molecular João Lobo Antunes, Faculdade de Medicina, Universidade de Lisboa, Lisbon, Portugal; 21 Euan MacDonald Centre for Motor Neurone Disease Research, Edinburgh, UK; 22 UK Dementia Research Institute, University of Edinburgh, Edinburgh, UK; 23 School of Medicine, Dentistry and Biomedical Sciences, Queen’s University Belfast, Belfast, UK; 24 Department of Molecular Neuroscience, Institute of Neurology, University College London, London, UK; 25 Department of Clinical and Movement Neurosciences, UCL Queen Square Institute of Neurology, University College London, London, UK; 26 Institute of Clinical Neurobiology, University Hospital Würzburg, Würzburg, Germany; 27 Charité University Hospital, Humboldt University, Berlin, Germany; 28 Koç University, School of Medicine, KUTTAM-NDAL, Istanbul, Turkey; 29 Department of Neurology, Academic Medical Center, Amsterdam, the Netherlands; 30 Department of Medical Biotechnology and Translational Medicine, Università degli Studi di Milano, Milan, Italy; 31 Unit of Medical Genetics and Neurogenetics, Fondazione IRCCS Istituto Neurologico ‘Carlo Besta’, Milan, Italy; 32 3rd Neurology Unit, Motor Neuron Diseases Center, Fondazione IRCCS Istituto Neurologico ‘Carlo Besta’, MIlan, Italy; 33 Department of Medical Biotechnology and Translational Medicine, University of Milan, Milan, Italy; 34 Neurology Unit, IRCCS Foundation Ca’ Granda Ospedale Maggiore Policlinico, Milan, Italy; 35 Genomic and Post-Genomic Center, IRCCS Mondino Foundation, Pavia, Italy; 36 Department of Health Sciences, University of Eastern Piedmont, Novara, Italy; 37 Department of Neurosciences, University of Padova, Padova, Italy; 38 Department of Clinical and Experimental Medicine, University of Pisa, Pisa, Italy; 39 Department of Clinical and Experimental Sciences, University of Brescia, Brescia, Italy; 40 ’Rita Levi Montalcini’ Department of Neuroscience, ALS Centre, University of Torino, Turin, Italy; 41 Neurologia 1, Azienda Ospedaliero Universitaria Città della Salute e della Scienza, Turin, Italy; 42 Laboratory of Neurological Diseases, Department of Neuroscience, Istituto di Ricerche Farmacologiche Mario Negri IRCCS, Milan, Italy; 43 Department of Clinical Research in Neurology, University of Bari at ‘Pia Fondazione Card G. Panico’ Hospital, Bari, Italy; 44 Neuromuscular Diseases Unit, Department of Neurology, Tel Aviv Sourasky Medical Center, Tel Aviv, Israel; 45 Department of Neurology, Hannover Medical School, Hannover, Germany; 46 Essener Zentrum für Seltene Erkrankungen (EZSE), University Hospital Essen, Essen, Germany; 47 Department of Clinical Sciences, Neurosciences, Umeå University, Umeå, Sweden; 48 Faculty of Medicine, Hebrew University of Jerusalem, Jerusalem, Israel; 49 Department of Neurology, the Agnes Ginges Center for Human Neurogenetics, Hadassah Medical Center, Jerusalem, Israel; 50 Center for Neural Science and Medicine, Cedars-Sinai Medical Center, Los Angeles, CA USA; 51 Department of Neurology, Neuromuscular Division, Cedars-Sinai Medical Center, Los Angeles, CA USA; 52 Service de Biochimie et Biologie Moléculaire, CHU de Tours, Tours, France; 53 UMR 1253, Université de Tours, Inserm, Tours, France; 54 Centre de référence sur la SLA, CHU de Tours, Tours, France; 55 Centre de référence sur la SLA, CHRU de Limoges, Limoges, France; 56 UMR 1094, Université de Limoges, Inserm, Limoges, France; 57 ICM, Institut du Cerveau, Inserm, CNRS, Sorbonne Université, Hôpital Pitié-Salpêtrière, Paris, France; 58 Hôpital des Peupliers, Ramsay Générale de Santé, Paris, France; 59 Département de Neurologie, Centre de référence SLA Ile de France, Hôpital de la Pitié-Salpêtrière, AP-HP, Paris, France; 60 ALS Unit, Hospital San Rafael, Madrid, Spain; 61 Functional Unit of Amyotrophic Lateral Sclerosis (UFELA), Service of Neurology, Bellvitge University Hospital, L’Hospitalet de Llobregat, Barcelona, Spain; 62 MND Clinic, Neurology Department, Hospital de la Santa Creu i Sant Pau de Barcelona, Universitat Autonoma de Barcelona, Barcelona, Spain; 63 Montreal Neurological Institute and Hospital, McGill University, Montreal, Quebec Canada; 64 Department of Neurology and Neurosurgery, McGill University, Montreal, Quebec Canada; 65 Department of Human Genetics, McGill University, Montreal, Quebec Canada; 66 Department of Neurology, Ulm University, Ulm, Germany; 67 Division of Neurodegeneration, Department of Neurology, University Medicine Mannheim, Medical Faculty Mannheim, Heidelberg University, Mannheim, Germany; 68 German Center for Neurodegenerative Diseases (DZNE) Ulm, Ulm, Germany; 69 Département de Pharmacologie Clinique, Hôpital de la Pitié-Salpêtrière, UPMC Pharmacologie, AP-HP, Paris, France; 70 Pharmacologie Sorbonne Université, Paris, France; 71 Institut du Cerveau, Paris Brain Institute ICM, Paris, France; 72 Laboratoire de Biostatistique, Epidémiologie Clinique, Santé Publique Innovation et Méthodologie (BESPIM), CHU-Nîmes, Nîmes, France; 73 Sorbonne Université, Paris Brain Institute, APHP, INSERM, CNRS, Hôpital de la Pitié Salpêtrière, Paris, France; 74 Genethon, CNRS UMR, Evry, France; 75 Department of Clinical and Movement Neuroscience, UCL Institute of Neurology, Queen Square, London, UK; 76 Department of Neuroscience, Mayo Clinic College of Medicine, Jacksonville, FL USA; 77 Popgen Biobank and Institute of Epidemiology, Christian Albrechts-University Kiel, Kiel, Germany; 78 Institute of Clinical Molecular Biology, Kiel University, Kiel, Germany; 79 Analytic and Translational Genetics Unit, Massachusetts General Hospital, Boston, MA USA; 80 Stanley Center for Psychiatric Research, Broad Institute of MIT and Harvard, Cambridge, MA USA; 81 Department of Psychiatry and Psychotherapy, Charité—Universitätsmedizin, Berlin, Germany; 82 Cancer Control Group, QIMR Berghofer Medical Research Institute, Herston, Queensland Australia; 83 Department of Internal Medicine, Genetics Laboratory, Erasmus Medical Center Rotterdam, Rotterdam, the Netherlands; 84 Department of Epidemiology, Erasmus Medical Center Rotterdam, Rotterdam, the Netherlands; 85 Medical Faculty Mannheim, University of Heidelberg, Heidelberg, Germany; 86 Central Institute of Mental Health, Mannheim, Germany; 87 Institute of Human Genetics, University of Bonn, Bonn, Germany; 88 Department of Genomics, Life and Brain Center, Bonn, Germany; 89 Division of Medical Genetics, University Hospital Basel and Department of Biomedicine, University of Basel, Basel, Switzerland; 90 Institute of Neuroscience and Medicine INM-1, Research Center Juelich, Juelich, Germany; 91 INSERM UMR1167—RID-AGE LabEx DISTALZ—Risk Factors and Molecular Determinants of Aging-Related Diseases, University of Lille, Centre Hospitalier of the University of Lille, Institut Pasteur de Lille, Lille, France; 92 Neuromuscular Diseases Research Section, Laboratory of Neurogenetics, National Institute on Aging, NIH, Porter Neuroscience Research Center, Bethesda, MD USA; 93 Department of Neurology, Johns Hopkins University, Baltimore, MD USA; 94 Molecular Genetics Section, Laboratory of Neurogenetics, National Institute on Aging, NIH, Porter Neuroscience Research Center, Bethesda, MD USA; 95 Universidade de São Paulo, São Paulo, Brazil; 96 Centre for Molecular Medicine and Biobanking and Department of Physiology and Biochemistry, Faculty of Medicine and Surgery, University of Malta, Msida, Malta; 97 University Medical Center Utrecht, Department of Psychiatry, Rudolf Magnus Institute of Neuroscience, Utrecht, the Netherlands; 98 Department of Human Genetics, David Geffen School of Medicine, University of California, Los Angeles, CA USA; 99 Center for Neurobehavioral Genetics, Semel Institute for Neuroscience and Human Behavior, University of California, Los Angeles, CA USA; 100 Department of Neurology, David Geffen School of Medicine, University of California, Los Angeles, CA USA; 101 Department of Neurology, University of California, San Francisco, CA USA; 102 Center for Neurodegenerative Disease Research, Perelman School of Medicine at the University of Pennsylvania, Philadelphia, PA USA; 103 Hans Berger Department of Neurology, Jena University Hospital, Jena, Germany; 104 Precision Neurology Unit, Department of Neurology, University Hospital Schleswig-Holstein, University of Luebeck, Luebeck, Germany; 105 Institute of Human Genetics, Jena University Hospital, Jena, Germany; 106 Department of Geriatric Medicine, Karolinska University Hospital Huddinge, Stockholm, Sweden; 107 Department of Neurology, Bujanov Moscow Clinical Hospital, Moscow, Russia; 108 Moscow Research and Clinical Center for Neuropsychiatry of the Healthcare Department, Moscow, Russia; 109 Department of Functional Biochemistry of the Nervous System, Institute of Higher Nervous Activity and Neurophysiology Russian Academy of Sciences, Moscow, Russia; 110 ALS-Care Center, ‘GAOORDI’, Medical Clinic of the St. Petersburg, St. Petersburg, Russia; 111 Department of Biotechnology, Jožef Stefan Institute, Ljubljana, Slovenia; 112 Biomedical Research Institute BRIS, Ljubljana, Slovenia; 113 Faculty of Chemistry and Chemical Technology, University of Ljubljana, Ljubljana, Slovenia; 114 Ljubljana ALS Centre, Institute of Clinical Neurophysiology, University Medical Centre Ljubljana, Ljubljana, Slovenia; 115 Institute of Biochemistry and Molecular Genetics, Faculty of Medicine, University of Ljubljana, Ljubljana, Slovenia; 116 Department of Molecular Genetics, Institute of Pathology, Faculty of Medicine, University of Ljubljana, Ljubljana, Slovenia; 117 Clinic of Neurology, Clinical Center of Serbia, School of Medicine, University of Belgrade, Belgrade, Serbia; 118 Sackler Faculty of Medicine, Tel Aviv University, Tel Aviv, Israel; 119 Centre for Motor Neuron Disease Research, Faculty of Medicine, Health and Human Sciences, Macquarie University, Sydney, New South Wales Australia; 120 Brain and Mind Centre, University of Sydney, Sydney, New South Wales Australia; 121 Australian Centre for Precision Health and Allied Health and Human Performance, University of South Australia, Adelaide, South Australia Australia; 122 Centre for Clinical Research, Australian Institute for Bioengineering and Nanotechnology, University of Queensland, Brisbane, Queensland Australia; 123 Department of Neurology, Royal Brisbane and Women’s Hospital, Brisbane, Queensland Australia; 124 Calvary Health Care Bethlehem, Parkdale, Victoria Australia; 125 Queensland Brain Institute, University of Queensland, Brisbane, Queensland Australia; 126 Fiona Stanley Hospital, Perth, Western Australia Australia; 127 Notre Dame University, Fremantle, Western Australia Australia; 128 Centre for Molecular Medicine and Innovative Therapeutics, Health Futures Institute, Murdoch University, Perth, Western Australia Australia; 129 Northcott Neuroscience Laboratory, ANZAC Research Institute, Concord, New South Wales Australia; 130 Molecular Medicine Laboratory, Concord Repatriation General Hospital, Concord, New South Wales Australia; 131 Discipline of Pathology and Department of Neuropathology, Brain and Mind Centre, University of Sydney, Sydney, New South Wales Australia; 132 The School of Biomedical Sciences, Faculty of Medicine, University of Queensland, Brisbane, Queensland Australia; 133 Centre for Healthy Brain Ageing, School of Psychiatry, University of New South Wales, Sydney, New South Wales Australia; 134 Neuroscience Research Australia Institute, Randwick, New South Wales Australia; 135 Neuropsychiatric Institute, the Prince of Wales Hospital, UNSW, Randwick, New South Wales Australia; 136 Neuromuscular Diseases Unit/ALS Clinic, Kantonsspital St. Gallen, St. Gallen, Switzerland; 137 Social Genetic & Developmental Psychiatry Centre, Institute of Psychiatry, Psychology and Neuroscience (IoPPN), King’s College London, London, UK; 138 NIHR BioResource Centre Maudsley, NIHR Maudsley Biomedical Research Centre (BRC) at South London and Maudsley NHS Foundation Trust (SLaM) & Institute of Psychiatry, Psychology and Neuroscience (IoPPN), King’s College London, London, UK; 139 Department Neurology, Emory University School of Medicine, Atlanta, GA USA; 140 Department of Neurology, University of Massachusetts Medical School, Worcester, MA USA; 141 Department of Translational Neuroscience, UMC Utrecht Brain Center, University Medical Center Utrecht, Utrecht University, Utrecht, the Netherlands; 142 Department of Neurology, Third Hospital, Peking University, Beijing, China; 143 Population Health Science, Bristol Medical School, Bristol, UK; 144 King’s College Hospital, London, UK; 145 IRCCS San Raffaele Hospital, Milan, Italy; 146 Vita Salute San Raffaele University, Milan, Italy; 147 Casa di Cura del Policlinico, Milan, Italy; 148 NEMO Clinical Center, Serena Onlus Foundation, Niguarda Ca’ Granda Hospital, Milan, Italy; 149 Civil Hospital of Brescia, Brescia, Italy; 150 Neurology Unit, ASST Valcamonica, Esine, Brescia Italy; 151 Ospedale Valduce, Como, Italy; 152 AO Ospedale Civile di Legnano, Legnano, Italy; 153 IRCCS Istituto Neurologico Nazionale ‘C. Mondino’, Pavia, Italy; 154 AO ‘San Gerardo’ di Monza and University of Milano-Bicocca, Milano-Bicocca, Italy; 155 AO ‘Ospedale di Circolo Fondazione Macchi’ di Varese, Varese, Italy; 156 Neurology Unit 1U, Azienda Ospedaliero Universitaria Città della Salute e della Scienza di Torino, Turin, Italy; 157 Department of Medical Genetics, Azienda Ospedaliero Universitaria Città della Salute e della Scienza, Turin, Italy; 158 Neurologia 3, Azienda Ospedaliero Universitaria Città della Salute e della Scienza di Torino, Turin, Italy; 159 Istituto Auxologico Italiano, IRCCS, Piancavallo, Italy; 160 Department of Neurology, ‘Amedeo Avogadro’ University of Piemonte Orientale, Novara, Italy; 161 Azienda Ospedaliero Universitaria ‘Maggiore della Carità’, Novara, Italy; 162 Department of Health Sciences, ‘Amedeo Avogadro’ University of Piemonte Orientale, Novara, Italy; 163 Department of Neurology and Multiple Sclerosis Center, Azienda Ospedaliero Universitaria San Luigi, Orbassano, Italy; 164 Department of Neurology, Azienda Ospedaliera ‘Ordine Mauriziano’ di Torino, Turin, Italy; 165 Department of Neurology, Ospedale Martini, ASL Città di Torino, Turin, Italy; 166 Department of Neurology, Ospedale Maria Vittoria, ASL Città di Torino, Turin, Italy; 167 Department of Neurology, Ospedale San Giovanni Bosco, ASL Città di Torino, Turin, Italy; 168 Ospedale Humanitas Gradenigo, Turin, Italy; 169 Department of Neurology, Ospedale ‘Santa Croce’ di Moncalieri, ASL Torino 5, Moncaliari, Italy; 170 Department of Neurology, Ospedale Civile di Ivrea, ASL Torino 4, Ivrea, Italy; 171 Department of Neurology, Presidio Ospedaliero di Ciriè, ASL Torino 4, Ciriè, Italy; 172 Department of Neurology, Ospedale ‘Degli Infermi’ di Biella, ASL Biella, Ponderano, Italy; 173 Department of Neurology, Ospedale ‘Sant’Andrea’ di Vercelli, ASL Vercelli, Vercelli, Italy; 174 Department of Clinical and Experimental Medicine, ‘Amedeo Avogadro’ University of Piemonte Orientale, Novara, Italy; 175 Department of Neurology, Ospedale Civile ‘Edoardo Agnelli’ di Pinerolo, ALS Torino 2, Pinerolo, Italy; 176 Department of Neurology, Azienda Ospedaliera ‘Santi Antonio e Biagio’ di Alesssandria, Alessandria, Italy; 177 Department of Neurology, Ospedale ‘Santo Spirito’ di Casale Monferrato, ASL Alessandria, Casale Monferrato, Italy; 178 Department of Neurology, Ospedale ‘San Giacomo’ di Novi Ligure, ASL Alesssandria, Novi Ligure, Italy; 179 Department of Neurology, Ospedale ‘Cardinal Massia’ di Asti, ASL Asti, Asti, Italy; 180 Department of Neurology, Azienda Ospedaliera ‘Santa Croce e Carle’ di Cuneo, Cuneo, Italy; 181 Department of Neurology, Ospedale ‘Maggiore Santissima Annuziata’ di Savigliano, ASL Cuneo 1, Savigliano, Italy; 182 Department of Anesthesiology, Ospedale ‘Maggiore Santissima Annuziata’ di Savigliano, ASL Cuneo 1, Savigliano, Italy; 183 Department of Neurology, Ospedale ‘Michele e Pietro Ferrero’ di Verduno, ASL Cuneo 2, Verduno, Italy; 184 Department of Neurology, Ospedale ‘Regina Montis Regalis’ di Mondovì, ASL Cuneo 1, Aosta, Italy; 185 Department of Neurology, Ospedale Regionale ‘Umberto Parini’ di Aosta, Aosta, Italy; 186 Unit of General Neurology, IRCCS Mondino Foundation, Pavia, Italy; 187 Department of Brain and Behavioural Sciences, University of Pavia, Pavia, Italy; 188 ALS Center, Department of Neurology, Azienda Ospedaliero Universitaria Maggiore della Carità, Novara, Italy; 189 Rare Neuromuscular Diseases Centre, Department of Human Neuroscience, Sapienza University, Rome, Italy; 190 Unit of Medical Genetics, Department of Medical Science, University of Ferrara, Ferrara, Italy; 191 Department of Basic Medical Sciences, Neurosciences and Sense Organs, University of Bari, Bari, Italy; 192 Neurological Department, Antonio Perrino’s Hospital, Brindisi, Italy; 193 National Institute of Digestive Diseases, IRCCS S. de Bellis Research Hospital, Castellana Grotte, Italy; 194 ASL Bari, San Paolo Hospital, Bari, Italy; 195 Department of Neuroscience, United Hospital of Foggia, Foggia, Italy; 196 Unit of Neurology, Department of Emergency and Critical Care, Fondazione IRCCS Casa Sollievo della Sofferenza, San Giovanni Rotondo, Italy; 197 Neurology Unit, Miulli Hospital, Acquaviva delle Fonti, Italy; 198 Unit of Neurology, ‘S. Giacomo’ Hospital, Bari, Italy; 199 Department of Neurology, ASL (Local Health Authority) at the ‘V Fazzi’ Hospital, Lecce, Italy; 200 Department of Neurology, ASL (Local Health Authority) at the ‘SS Annunziata’ Hospital, Taranto, Italy

**Keywords:** Motor neuron disease, Genome-wide association studies, Neurodegenerative diseases

Correction to: *Nature Genetics* 10.1038/s41588-021-00973-1, published online 6 December 2021.

In the version of this article initially published, the affiliation for Nazli Başak appeared incorrectly. Nazli Başak is at Koç University, School of Medicine, KUTTAM-NDAL, Istanbul, Turkey, and not Bogazici University. The error has been corrected in the HTML and PDF versions of the article.

