# Supplementary material for: Common and rare variant association analyses in amyotrophic lateral sclerosis identify 15 risk loci with distinct genetic architectures and neuron-specific biology
Source: Nat Genet. 2021 Dec 6;53(12):1636–48. doi: 10.1038/s41588-021-00973-1 (PMC8648564; doi:10.1038/s41588-021-00973-1)
Supplement: Supplementary file 1 — Supplementary Note, Figs. 1–18 and Tables 4–25 and 27–30 [file 41588_2021_973_MOESM1_ESM.pdf]

nature portfolio

https://doi.org/10.1038/s41588-021-00973-1

Supplementary information

Common and rare variant association analyses in amyotrophic lateral sclerosis identify 15 risk loci with distinct genetic architectures and neuron-specific biology

Common and rare variant association analyses in Amyotrophic Lateral Sclerosis identify 15 risk loci with distinct genetic architectures and neuron-specific biology

- Supplementary Information -

Supplementary Text ... 2
- Description of newly genotyped GWAS cohorts ... 2
- Description of Asian ancestries GWAS ... 7
- GWAS quality control ... 8
- Description whole-genome sequencing cohorts ... 10
- Whole-genome sequencing quality control ... 13
- Acknowledgments ... 15

Supplementary Figures ... 17
Supplementary Tables ... 51
References ... 67

Supplementary Text

Description of newly genotyped GWAS cohorts

Cohort 66 cKingsOE - Illumina OmniExpress

Germany (Grosskreutz). ALS patients and controls were recruited at the Thuringian Neuromuscular Center, Jena University Hospital, Germany. Written informed consent was obtained. ALS patients were diagnosed according to the El-Escorial criteria[1] of definite, probable or laboratory-supported probable ALS. Control subjects were population based controls matched for sex and age. All participants gave written informed consent and the Local ethics committee of the Medical Faculty of Friedrich Schiller University Jena approved this protocol.

Italy - Milan (Beghi). ALS patients enrolled were residents of the Lombardy Region, Northern Italy, and were primarily diagnosed in or referred to secondary and tertiary ALS centers included in the population based registry (SLALOM). Patients were not pre-screened for any mutations related to ALS. Control subjects were selected from the lists of the general practitioners of the companion cases and were matched for sex, age and geographic region. The structure of the SLALOM Registry has been described previously[2]. All participant gave written informed consent and the Ethical Committee of Città della Salute Hospital approved this protocol.

Italy - Turin (Chio). ALS patients were included in the Piemonte and Valle d'Aosta ALS Register (PARALS), north western Italy. Patients were not pre-screened for any mutations related to ALS. Control subjects were population based controls matched for sex, age and geographic region within Piemonte and Valle d'Aosta. The PARALS has been described in detail previously[3]. All participants gave written informed consent and the Ethical Committee of Città della Salute Hospital approved this protocol.

Italy - Bari (Logroscino). ALS patients were enrolled as part of the SLAP registry, a population-based registry including all ALS patients who were residents of Puglia region, SouthEast Italy. All ALS cases were diagnosed or referred to a network of diagnostic facilities including all secondary and tertiary ALS centers present in the geographic area. The base population was the population of Puglia in the same period according to the national census. Patients were not pre-screened for any mutations related to ALS. Control subjects were selected from the lists of the general practitioners (GPs) of the cases and were matched for sex, age, sex, and place of residence. The SLAP Registry has been described in detail previously[4]. All individuals gave written informed consent and the Institutional Review Board of the Azienda Sanitaria Locale, Lecce approved this protocol.

Sweden (Graff) This cohort included patients with ALS recruited at the Memory Clinic, Karolinska University Hospital, Huddinge, Sweden, after 1992. Patients were diagnosed with ALS according to the

revised El-Escorial criteria for ALS[1]. All individuals gave written informed consent and the Ethics Committee Stockholm approved this protocol.

The Netherlands (Veldink, van den Berg). ALS patients were diagnosed with ALS at the tertiary referral clinic for motor neuron disease at the University Medical Center Utrecht (Dutch ALS Center) or were included in the Prospective ALS Study in The Netherlands. Patients were not pre-screened for any mutations related to ALS. Control subjects were population based controls matched for sex, age and geographic region within the Netherlands. The Prospective ALS study in The Netherlands has been described in detail previously[5]. All individuals gave written informed consent and the University Medical Center Utrecht Medical Ethics Committee, Utrecht approved this protocol.

Cohort 104 cKingsGSA - Illumina GSA

Canada (Rouleau). ALS patients and unaffected controls were recruited from neurological clinics across Québec, Canada. Control subjects were recruited from various studies, either as the unaffected parents of psychiatric probands, unaffected relatives of probands of non-ALS neurological diseases, and unaffected individuals recruited specifically as controls. Samples were included without selection for variant carrier status, age, reported sex, or reported ethnicity. All individuals signed informed consent according to the relevant institutional ethics protocol. All individuals gave written informed consent and the Review Ethics Board at McGill University Health Center, Montreal approved this protocol.

France - Paris (Millecamps, Meininger, Salachas). ALS patients were diagnosed with probable or definite ALS according to the revised El Escorial criteria[1] between 1996 and 2004 at the national ALS reference center of the Pitié-Salpêtrière hospital (Paris). These patients were cases with sporadic occurrence of the disease (with no evident familial history). Control subjects were individuals of European ancestries with a French background (healthy spouses and husbands) matched for age and sex collected at the same period. All individuals gave written informed consent and the Medical Research Ethics Committee of "Assistance Publique-Hôpitaux de Paris" approved this protocol.

France - Tours (Vourc'h, Corcia, Couratier). ALS patients were diagnosed with probable or definite ALS according to the El Escorial Criteria by neurologists specialized in motor neuron diseases at the Reference centers for ALS of the University Hospitals of Limoges and Tours (LITORALS federation), members of the French FILSLAN networks. All individuals gave written informed consent and the ethics committee of Tours Hospital and Limoges University Hospital this protocol.

Slovenia (Koritnik, Rogelj, Zidar, Ravnik-Glavač, Glavač). ALS patients were diagnosed at the tertiary Ljubljana ALS Centre which takes care of the majority of Slovenian ALS patients. Control samples were healthy individuals matched for gender and age and were unrelated to ALS patients. More details were described previously[6]. All individuals gave written informed consent and the National Medical Ethics Committee of Republic of Slovenia approved this protocol.

The Netherlands (Veldink, van den Berg). ALS patients were diagnosed with ALS at the tertiary referral clinic for motor neuron disease at the University Medical Center Utrecht (Dutch ALS Center) or were included in the Prospective ALS Study in The Netherlands. Patients were not pre-screened for any mutations related to ALS. Control subjects were population based controls matched for sex, age and geographic region within the Netherlands. The Prospective ALS study in The Netherlands has been described in detail previously[5]. All individuals gave written informed consent and the University Medical Center Utrecht Medical Ethics Committee, Utrecht approved this protocol.

Cohort 107 cAUS - Illumina Exome

Australia (Wray, Blair, Kiernan). This cohort includes the University of Sydney's Australian Motor Neuron Disease DNA Bank (MND Bank) cohort recruited April 2000 to June 2011 (493 cases, 497 controls), with study protocol approved by the Sydney South West Area Health Service Human Research Ethics Committee (HREC). Cases were recruited from around Australia via state-based MND associations with diagnosis verified by a neurologist. The remainder of the cases (N=467) were recruited from clinics across Australia between 2015 and 2017 under HREC approvals from Royal Brisbane and Women's Hospital (RBWH; N=220), Macquarie University Multidisciplinary Motor Neurone Disease Clinic (N=205), Calvary Health Care Bethlehem in Melbourne (N=32), Fiona Stanley Hospital in Perth (N=10), and from 2016 under HREC approvals at each site for the sporadic ALS Australia Systems Genomics Consortium (SALSA-SGC). The ALS cases were diagnosed with definite or probable ALS according to the revised El Escorial criteria[1]. Some controls were recruited as either partners or friends of patients, healthy individuals free of neuromuscular diseases (N=166; N=82 from Macquarie, N=84 from RBWH). Additional controls were included from the Older Australian Twin Study (N=89, selected as single from a twin pair) from QIMR Berghofer Medical Research Institute, University of New South Wales and the University of Melbourne, and from the University of New South Wales Sydney Memory & Ageing Study (N=91), all approved by their respective HRECs. From this cohort N=846 cases and N=665 controls have data included in the dbGAP upload phs002068.v1.p1. All individuals gave written informed consent and the Sydney South West Area Health Service Human Research Ethics Committee (HREC) and HREC at the different sites: University of Sydney, Western Sydney Local Health District, Royal Brisbane and Women Hospital Metro North, South Metropolitan Health Service, Macquarie University, QIMR Berghofer Medical Research Institute, University of New South Wales and the University of Melbourne approved this protocol.

Cohort 117 Qskin - Illumina GSA

Australia (Whiteman, Olsen). The QSkin Sun and Health Study is a cohort of men and women aged 40–69 years randomly sampled from the population of Queensland, Australia in 2011 (ref. [7,8]). The cohort was established to study the development of skin cancer and melanoma; the baseline survey collected demographic and health information. The study sample for these analyses were a subset of the cohort who provided a saliva sample; participants were not screened for ALS. All individuals gave written informed consent and the Human Research Ethics Committee at the QIMR Berghofer Medical Research Institute approved this protocol.

Cohort 118 Sripke - Illumina GSA

Germany (Ripke). Controls were collected as part of The Berlin Psychosis Study (BePS). This is a case-control sample initiated in greater Berlin aiming to facilitate the discovery of novel genetic variants associated with schizophrenia. The current sample consists of control samples of European ancestries. Control subjects were recruited into the study via local advertisement and participant databases. Individuals were excluded if they had ever been diagnosed with schizophrenia, schizoaffective disorder or bipolar disorder. All individuals gave written informed consent and the Charité Universitätsmedizin, Berlin Medical Ethics Committee approved this protocol.

Cohort 119 and 120 cKingsGSA2 and cErasmusGSA - Illumina GSA

The following cases and controls were randomized over these two cohorts.

Canada (Rouleau). ALS patients and unaffected controls were recruited from neurological clinics across Québec, Canada. Control subjects were recruited from various studies, either as the unaffected parents of psychiatric probands (n = 334), unaffected relatives of probands of non-ALS neurological diseases (n = 245), and unaffected individuals recruited specifically as controls (n = 164). Samples were included without selection for variant carrier status, age, reported sex, or reported ethnicity. All individuals gave written informed consent and the Review Ethics Board Office at McGill University Health Center, Montreal approved this protocol.

France (Corcia, Vourc'h, Couratier). ALS patients were diagnosed with probable or definite ALS according to the El Escorial Criteria by neurologists specialized in motor neuron diseases at the Reference centers for ALS of the University Hospitals of Limoges and Tours (LITORALS federation), members of the French FILSLAN networks. All individuals gave written informed consent and the ethics committee of Tours Hospital and Limoges University Hospital this protocol.

Germany (Petri). Blood was collected from sporadic and familial ALS cases at the ALS/MND Clinic of the Department of Neurology of Hannover Medical School, Germany. Each patient provided informed consent for participation in the study. All patients were examined at least once by a neurologist specialized in ALS. Extensive clinical workup including magnetic resonance imaging (MRI), cerebral spinal fluid analysis, electromyography (EMG), and nerve conduction studies (NCS) was performed to exclude ALS-mimicking conditions. Longitudinal information over a number of years was available for most individuals as described in ref. [9,10]. All individuals gave written informed consent and the Medical Ethics Committee of Hannover Medical School approved this protocol.

Ireland (McLaughlin, Hardiman). Cases were diagnosed with probable or definite ALS according to the 1994 El-Escorial Criteria[11] by neurologists specialized in motor neurone diseases at Beaumont Hospital in Dublin. Patients were referred from all regions in Ireland and were part of an ongoing population-based prospective ALS registry. Control samples were healthy individuals matched for geography, sex and age. They were either spouses, those accompanying patients to the ALS clinic or community-derived volunteers. All individuals reported Irish ancestry for at least three generations. All individuals gave

written informed consent and the Beaumont Hospital Research & Ethics Committee, Dublin approved this protocol.

Italy - Bari (Logroscino). ALS patients were enrolled as part of the SLAP registry, a population-based registry including all ALS patients who were residents of Puglia region, SouthEast Italy. All ALS cases were diagnosed or referred to a network of diagnostic facilities including all secondary and tertiary ALS centers present in the geographic area. The base population was the population of Puglia in the same period according to the national census. Patients were not pre-screened for any mutations related to ALS. Control subjects were selected from the lists of the general practitioners (GPs) of the cases and were matched for sex, age, sex, and place of residence. The SLAP Registry has been described in detail previously[4]. All individuals gave written informed consent and the Institutional Review Board of the Azienda Sanitaria Locale, Lecce approved this protocol.

Italy - Milan (Silani, Ticozzi). Patients were diagnosed with ALS according to the El Escorial revised criteria by the SLAGEN Consortium, which includes Italian referral centers for motor neuron diseases. Patients were not pre-screened for any ALS-associated mutations. Control subjects were population-based controls matched for sex, age and geographic region within Italy. All individuals gave written informed consent and the Ethics Committee of the IRCCS Istituto Auxologico Italiano, Milan approved this protocol.

The Netherlands (Veldink, van den Berg). ALS patients were diagnosed with ALS at the tertiary referral clinic for motor neuron disease at the University Medical Center Utrecht (Dutch ALS Center) or were included in the Prospective ALS Study in The Netherlands. Patients were not pre-screened for any mutations related to ALS. Control subjects were population based controls matched for sex, age and geographic region within the Netherlands. The Prospective ALS study in The Netherlands has been described in detail previously[5]. All individuals gave written informed consent and the University Medical Center Utrecht Medical Ethics Committee, Utrecht approved this protocol.

Russia (Brylev). Patients were diagnosed with probable or definite ALS according to the 1994 El-Escorial Criteria[11] by neurologists specialized in motor neuron diseases in Moscow ALS center and St-Petersburg ALS-care service. Patients were referred from all regions of Russia, and were not pre-screened for any mutations related to ALS. Control subjects were healthy individuals matched for sex and age, some of them spouses or those accompanying patients to the ALS clinic. All individuals gave written informed consent and the Local ethical committee of Buyanov city hospital, Moscow approved this protocol.

Serbia (Stevic). All samples were taken from patients seen at the Clinic of Neurology, School of Medicine, University of Belgrade, Serbia. The Clinic of Neurology is a tertiary center caring for a large number of ALS patients in Serbia. Diagnoses were made by neurologists specialized in neuromuscular diseases and motor neuron diseases. After informed consent, full demographic and clinical information was entered into the clinic database. All individuals gave written informed consent and the Ethics Committee of the School of Medicine at the University of Belgrade approved this protocol.

UK (P. Shaw, Cooper-Knock). All patients were reviewed by a senior consultant Neurologist and diagnosed with definite or probable ALS, as defined by the El Escorial criteria[1]. A detailed family history was taken from each patient. Population, age and sex matched control subjects were recruited from partners or unrelated carers of patients with ALS. The South Sheffield Research Ethics Committee approved the study, and informed consent was obtained for all samples. All individuals gave written informed consent and the Yorkshire and the Humber - Sheffield Research Ethics Committee approved this protocol.

US - Cedar-Sinai (Baloh). Cases were identified at the Cedar-Sinai ALS Clinic in Los Angeles. All cases met El Escorial criteria for definite or probable ALS. All individuals gave written informed consent and the Institutional review board of Cedars-Sinai, Los Angeles approved this protocol.

US - UCLA (Ophoff). Cases were identified at the ALS clinical centers of University of California Los Angeles and University of California San Francisco. Patients fulfilled the El-Escorial Criteria for definite or probable ALS. Control participants were population-based individuals. All individuals gave written informed consent and the Institutional review board of the University of California at Los Angeles approved this protocol.

Description of Asian ancestries GWAS

Chinese ancestries GWAS (Benyamin et al.[12]). The ALS patients of Chinese ancestries were diagnosed with ALS according to the revised El-Escorial criteria[1] at Peking University Third Hospital (Beijing, China) by a neurologist specialized in ALS. Control subjects attended the Peking University Third Hospital, Peking University Sixth Hospital or Shanghai Changzheng Hospital (Shanghai) and had no medical or family history for neurological diseases. All patients and controls were of Chinese ancestry from Mainland China.

Japanese ancestries GWAS (Nakamura et al.[13]). The ALS patients of Japanese ancestries were ascertained by the Japanese Consortium for ALS research[14] throughout Japan. Patients were diagnosed with ALS according to the revised El-Escorial criteria[1] and had no family history for ALS. All patients were of Japanese ancestry. Control subjects were obtained from the Tohuku Medical Megabank Project[15].

GWAS quality control

Cohort-level quality control

For each cohort, SNPs were first annotated according to dbSNP150 and mapped to the hg19 reference genome. All multi-allelic and palindromic (A/T or C/G) SNPs were excluded. Subsequently, basic quality control was first performed by cohort, excluding extremely low-quality SNPs and genotyped individuals as well as excluding extreme population outliers. Low quality SNPs and genotyped individuals were excluded using PLINK 1.9 (--geno 0.1 and --mind 0.1)[16]. Population structure was assessed by projecting HapMap3 principal components (PCs) using EIGENSOFT[17] 6.1.4. Extreme outliers from the European ancestries population were removed (> 25 SD on PC1-4). Finally, cohorts were merged into strata based on genotyping platforms to preserve the maximum number of SNPs (Supplementary Table 2). Four out of 6 strata were formed by only a single platform. The remaining two strata included multiple platforms with 420,952 and 299,625 overlapping SNPs across platforms in these strata.

Stratum-level quality control

After excluding major SNP and sample outliers in cohort QC and merging cohorts into strata, stringent SNP QC was performed per stratum. The following filter criteria were applied: MAF > 0.01, SNP genotyping rate > 0.98, Deviation from Hardy-Weinberg disequilibrium in controls $P > 1 \times 10^{-5}$, and haplotype-biased missingness $P > 1 \times 10^{-8}$ (PLINK --maf 0.01, --geno 0.02, --hwe 1e-5 midp include-nonctrl, --test-mishap). Then, more stringent QC thresholds were applied to exclude individuals: individual missingness > 0.02, inbreeding coefficient |F| > 0.2, mismatches between genetic and reported gender, and missing phenotypes (PLINK --mind 0.02, --het, --check-sex). Subsequently, SNPs with a differential missingness (--test-missing midp) $P < 1 \times 10^{-4}$ were excluded. Duplicate individuals were removed (PI_HAT > 0.8). Finally, outliers from the European ancestries reference population (projected on HapMap 3: > 10 SD from CEU on PC1-4 and projected on 1000 Genomes: > 4 SD from CEU on PC1-4) and outliers within the stratum itself (> 4SD from stratum mean on PC1-4) were removed (Supplementary Figure 17).

After removing outliers, principal components were recalculated for each stratum. To assess the result of quality control prior to imputation, genomic inflation factors per stratum were calculated using SAIGE[18] to run a logistic mixed model regressing SNP genotype on ALS case-control status. SAIGE internally calculates an equivalent of a genetic relationship matrix to correct for relatedness and population structure. Additionally, PC1-20 and genotyping platform were included as covariates.
The number of individuals and SNPs passing quality control for each stratum prior to imputation is described in Supplementary Table 2.

Post-imputation quality control

Strata were then imputed using the HRC reference panel (r.1.1 2016) on the Michigan Imputation Server[19]. Data was phased using Eagle 2.3. After imputation, one individual of each pair of related

samples across strata (PI_HAT > 0.125) was removed whereas related pairs within a stratum were retained since the genetic relationship matrix corrects for relatedness. Post-imputation variant-level quality control included removing all monomorphic SNPs and multi-allelic SNPs from each stratum. SNPs with MAF < 0.1% in the HRC imputation panel were excluded. Subsequently, INFO scores were calculated for each stratum based on dosage information using SNPTEST[20] v2.5.4-beta3. Within each stratum, SNPs with an INFO-score < 0.6 and those deviating from Hardy-Weinberg equilibrium at $P < 1 \times 10^{-5}$ in control subjects were removed. Effective sample size was calculated for each stratum:

$$N_{effective} = \frac{4 \cdot N_{cases} \cdot N_{controls}}{N_{cases} + N_{controls}}$$

The difference in sample size and number of SNPs for each stratum prior to imputation, resulted in a different set of SNPs passing post-imputation quality control for each stratum. Therefore, only SNPs that were successfully imputed in an effective sample meeting > 50% of the maximum effective sample size were included.

The number of individuals and SNPs passing quality control for each stratum after imputation is described in Supplementary Table 2.

Description whole-genome sequencing cohorts.

The Netherlands (Veldink, van den Berg). ALS patients were diagnosed with ALS at the tertiary referral clinic for motor neuron disease at the University Medical Center Utrecht (Dutch ALS Center) or were included in the Prospective ALS Study in The Netherlands. Patients were not pre-screened for any mutations related to ALS. Control subjects were population based controls matched for sex, age and geographic region within the Netherlands. The Prospective ALS study in The Netherlands has been described in detail previously[5]. All individuals gave written informed consent and the University Medical Center Utrecht Medical Ethics Committee, Utrecht approved this protocol.

UK MNDA Biobank (C. Shaw, P. Shaw, Al-Chalabi, Morrison). Cases were diagnosed with ALS in one of 20 UK hospitals by neurologists specialized in motor neuron diseases. Patients had no family history for ALS. All participated in the UK National Biobank for Motor Neuron Disease Research. Patients had no family history for ALS and were of self-reported European descent. All individuals gave written informed consent and the Trent University Medical Ethics Committee approved this protocol.

Turkey (Basak). ALS patients were recruited from hospitals across Turkey between 2002 and 2019. DNA samples were collected at the Boğaziçi University. A full description is provided in reference [21]. All individuals gave written informed consent and the Ethics Committee on Research with Human Participants (INAREK) at Bogazici University, Istanbul approved this protocol.

Belgium (van Damme). Patients were diagnosed with ALS at the tertiary referral clinic for motor neuron diseases at the University Hospitals in Leuven. Patients were pre-screened for mutations in *C9orf72*, *SOD1*, *TARDBP* and *FUS*, but were not always excluded in case of pathogenic mutation[22]. Control subjects were often spouses of patients, supplemented with age- and sex-matched controls from other local studies[23]. All individuals gave written informed consent and the Ethical Committee of University Hospital Leuven approved this protocol.

Ireland (Hardiman, McLaughlin). Cases were diagnosed with probable or definite ALS according to the 1994 El-Escorial Criteria[11] by neurologists specialized in motor neurone diseases at Beaumont Hospital in Dublin. Patients were referred from all regions in Ireland and were part of an ongoing population-based prospective ALS registry. Patients were selected for sequencing such that all areas of Ireland were adequately represented. Control samples were neurologically healthy volunteers matched for geography, sex and age sampled from the community. All individuals reported Irish ancestry for at least three generations. All individuals gave written informed consent and the Beaumont Hospital Research & Ethics Committee, Dublin approved this protocol.

Spain (Mora Pardina, Povedano). ALS patients diagnosed with definite or probable ALS according to the El Escorial criteria[11]. Patients were seen by neurologists and neurophysiologists at the tertiary referral centers: the Bellvitge hospital and Carlos III hospital for Catalonia and Madrid respectively. Controls

were healthy individuals, without familial history of ALS, matched for age and sex. All individuals gave written informed consent and the Bellvitge University Hospital Ethics Committee, Barcelona and "Comité de Ética de la Investigación del Hospital Carlos III", Madrid approved this protocol.

United States (Landers, Glass). All samples were taken from patients seen at the Emory ALS Center in Atlanta, Georgia, USA. The Emory Center is a tertiary care ALS clinic caring for a large proportion of patients in Georgia and surrounding states[24]. Diagnoses were made by neurologists specialized in neuromuscular diseases and motor neuron diseases. After informed consent, full demographic and clinical information was stored into the clinic database. DNA was collected and stored. All individuals gave written informed consent and the Committee for the Protection of Human Subjects in Research of the University of Massachusetts Medical School, Worcester approved this protocol.

France (Corcia, Couratier, Vourc'h). ALS patients were diagnosed with probable or definite ALS according to the El Escorial Criteria[1] by neurologists specialized in motor neuron diseases at the Reference centers for ALS of the University Hospitals of Limoges and Tours (LITORALS federation), members of the French FILSLAN networks. All individuals gave written informed consent and the ethics committee of Tours Hospital and Limoges University Hospital this protocol.

Sweden (Andersen). Cases were diagnosed with probable or definite ALS according to the revised El-Escorial Criteria[1] by neurologists specialized in motor neuron diseases. Control individuals were free of any neuromuscular disease and matched for age, gender and ethnicity. Healthy controls were spouses of ALS patients or patients with other neurological diseases. All participants were from Swedish descent all reporting Northern Swedish citizenship for at least three generations. All individuals gave written informed consent and the Regional Ethical Review Board in Umeå approved this protocol.

Israel (Gotkine, Drory). ALS patients were diagnosed with probable or definite ALS according to the El Escorial Criteria[1] and in follow-up at the tertiary referral ALS clinic at the Hadassah University Hospital, Jerusalem or Tel-Aviv Sourasky Medical Center in Tel-Aviv. Patients were not pre-screened for any mutations related to ALS. Patients were referred from all regions in Israel and participated in a prospective ALS database and sample repository. All individuals gave written informed consent and the Hadassah University Hospital Institutional Review Board, Hadassah and The Institutional Review Board of Tel Aviv Sourasky Medical Center, Tel Aviv and approved this protocol.

Portugal (deCarvalho, Pinto). Patients were diagnosed with possible, probable or definite ALS according to the revised El-Escorial criteria by neurologists specialized in motor neuron diseases. Both cases with and without a family history (third degree relatives) were included. Control subjects were spouses or those accompanying patients to the clinic. All individuals gave written informed consent and the Local Research Ethics Committee at the Faculty of Medicine, University of Lisbon, Lisbon approved this protocol.

Italy (Chio, Ticozzi, Silani). Patients were diagnosed with ALS according to the El Escorial revised criteria at the ALS tertiary referral center of Istituto Auxologico Italiano IRCCS. All patients had probable or

definite familial ALS according to the Byrne criteria for FALS[25]. Patients were pre-screened for mutations in the *SOD1*, *TARDBP*, *FUS* and *C9orf72* genes. All individuals gave written informed consent and the Ethics Committee of the IRCCS Istituto Auxologico Italiano, Milan approved this protocol.

Switzerland (Weber). ALS patients were diagnosed at the Muskelzentrum/ALS clinic at the Kantonsspital St. Gallen, a tertiary referral center in Northern Switzerland. Patients fulfilled the El-Escorial Criteria for probable lab supported, probable or definite or ALS. Control subjects were healthy blood donors matched for age and gender. All individuals gave written informed consent and the Kantonale Ethikkomission des Kantons St. Gallen, St. Gallen approved this protocol.

Whole-genome sequencing quality control

For variant-level quality control, we set sites with a genotype quality (GQ) < 10 to missing and SNVs and indels with quality (QUAL) scores < 20 and < 30, respectively, were removed. We subsequently performed sample-level quality control. An overview of the number of samples that have been excluded at each of the following QC steps, stratified by country of origin, is included in Supplementary Table 3.

We estimated kinship coefficients (i.e., relatedness) using the KING method[26], as implemented in the SNPRelate package in R. In some instances, cohorts were intentionally enriched for related samples. We identified all pairs of related individuals (kinship > 0.0625).

We calculated the transition-transversion ratio in each sample using SnpSift[27] 4.3p. In WGS data, the expected transition-transversion ratio is ~2.0. Samples with a Ti/Tv ratio ± 6 SD from the full distribution of samples were removed.

Per sample, we calculated the total number of SNVs and total number of singletons. We removed samples with a total number of SNVs or Singletons > 6 SD from the mean. The transition in sequencing platforms from HiSeq 2000 to HiSeq X (which occurred in parallel with a change in the calling pipeline, to improve indel detection) caused an increase in observed indels per sample. Samples were thus filtered by platform (HiSeq 2000 or HiSeq X) and removed samples with number of indels ± 6 SD from the mean of their respective group.

We calculated average sample depth and again observed noticeable differences between those samples sequenced on the HiSeq 2000 and the HiSeq X, where average depth of coverage was somewhat higher (35X, on average) for samples sequenced on HiSeq 2000 compared to the samples sequenced on the HiSeqX (25X, on average). We removed no samples at this step.

Using the genetically inferred sex based on the number of X and Y chromosome, we tested to see if the inferred genetic sex was concordant with the sex as annotated in the available phenotype information. We excluded samples with mismatching information and samples for which phenotypic information is missing at this time.

We performed the remaining sample QC on high-quality variants: We removed all multi-allelic SNVs, PLINK 1.9 (--geno), variants with a missingness > 2% were excluded. We calculated Hardy-Weinberg equilibrium (HWE) in controls only, PLINK 1.9 (--hwe midp), and removed all variants with HWE $P < 1 \times 10^{-5}$. We calculated differential missingness, PLINK 1.9 (--test-mishap) between cases and controls and removed variants with $P < 1 \times 10^{-8}$. Samples with a missingness > 2%, in SNV and indels, were excluded. Final steps of sample QC was performed on a set of variants with a MAF > 10%, SNP missingness < 0.1%, variants residing outside four complex regions (the major histocompatibility complex (MHC) on chromosome 6; the lactase locus (LCT), on chromosome 2; and inversions on chromosomes 8 and 17); and we excluded the A/T and C/G variants. We used the SNVs to calculate observed and expected

autosomal homozygous genotype counts for each sample PLINK 1.9 (--het); samples with |F| > 0.1 were excluded. We excluded duplicate samples; PLINK 1.9 (--genome) with a PIHAT > 0.8, keeping the maximum number of non-duplicated individuals.

Principal component analysis (PCA) implemented in EIGENSOFT[17] was used to visualize potential structure in the data, induced by population stratification or other variables. Projections onto HapMap3 and the 1KG phase3 v5 populations indicated that the samples were primarily of European ancestry, though some were of African or East Asian ancestries, while other samples appeared to be admixed. Outliers from the European population (HapMap3: > 10 SD on PC1-4, 1KG: > 4 SD on PC1-4) were excluded from further analyses.

All samples were sent in batches to Illumina for sequencing. To prevent spurious association due to batch specific artifacts, we regressed all variants on a dummy coded variable indicating batch using PLINK 1.9 (--logistic). All variants with an association $P < 1 \times 10^{-10}$ in at least 1 batch were excluded.

Acknowledgments

W.v.R. is supported by funding provided by the Dutch Research Council (NWO) [VENI scheme grant 09150161810018] and Prinses Beatrix Spierfonds (neuromuscular fellowship grant W.F19-03). J.J.F.A.v.V. is funded by Projectnumber W.OR20-08 (The “Repeatome” as a basis for new treatments of ALS) of the Prinses Beatrix Spierfonds. K.P.K. is supported by funding provided by the Dutch Research Council (NWO) [VIDI grant 91719350]. G.S. was supported by a PhD studentship from the Alzheimer’s Society. E.H. and J.M. were supported by Medical Research Council (MRC) grant K013807 (awarded to J.M). mQTL SMR data analysis was undertaken using high-performance computing supported by a Medical Research Council (MRC) Clinical Infrastructure award M008924 (awarded to J.M.). French ALS patients of the Pitié-Salpêtrière hospital (Paris) have been collected with ARSla funding support. D.B. and T.R.G. received funding from Biogen and UK Medical Research Council (MRC Epidemiology Unit, MC_UU_00011/1 and MC_UU_00011/4) for this project. G.D.S. works in the Medical Research Council Integrative Epidemiology Unit at the University of Bristol MC_UU_00011/1. D.B., E. Tsai and H.R. are employees of Biogen. J.P.R. is funded by the Canadian Institutes of Health Research (FRN 159279). A.A.K is supported by The Motor Neurone Disease Association (MNDA) and NIHR Maudsley Biomedical Research Centre. R.J.P. is supported by the Gravitation program of the Dutch Ministry of Education, Culture, and Science and the Netherlands Organization for Scientific Research (NWO; BRAINSCAPES). Project MinE Belgium was supported by a grant from IWT (n° 140935), the ALS Liga België, the National Lottery of Belgium and the KU Leuven Opening the Future Fund (awarded to P.V.D.). P.V.D holds a senior clinical investigatorship of FWO-Vlaanderen and is supported by the E. von Behring Chair for Neuromuscular and Neurodegenerative Disorders, the ALS Liga België and the KU Leuven funds “Een Hart voor ALS”, “Laeversfonds voor ALS Onderzoek” and the “Valéry Perrier Race against ALS Fund”. Several authors of this publication are members of the European Reference Network for Rare Neuromuscular Diseases (ERN-NMD). The authors are pleased to acknowledge the contribution of “Live now” Charity Foundation and Moscow ALS palliative care service for supporting patients with ALS and their families. G.A.R is supported by the Canadian Institutes of Health. Research Australia including its Ice Bucket Challenge Grant. This work was supported in part by the Intramural Research Programs of the NIH, National Institute on Aging (Z01-AG000949-02). We acknowledge funding from the National Health and Medical Research Council (NHMRC) (1078901, 1083187, 1113400, 1095215, 1121962, 1173790, Enabling Grant #402703). S.T.N. is supported by funding through the FightMND Mid-Career Fellowship. The Older Australian Twins Study (OATS, used for controls) acknowledges funding from the NHMRC/Australian Research Council Strategic Award (401162) and NHMRC (1405325, 1024224, 1025243, 1045325, 1085606, 568969, 1093083). OATS was facilitated through access to Twins Research Australia, a national resource supported by a NHMRC Centre of Research Excellence Grant (1079102). The Sydney Memory and Ageing Study (Sydney MAS, used for controls) has been funded by three NHMRC Program Grants (350833, 568969, and 1093083). We also acknowledge the OATS and Sydney MAS research teams: https://cheba.unsw.edu.au/research-projects/sydney-memory-and-ageing-study; https://cheba.unsw.edu.au/project/older-australian-twins-study. D.C.W. is supported by a Research Fellowship [APP1155413] from the National Health and Medical Research Council of Australia (NHMRC). The QSkin Study is supported by Grants [APP1185416, APP1073898, APP1063061] from the National

Health and Medical Research Council of Australia (NHMRC). Several authors of this publication are members of the Netherlands Neuromuscular Center (NL-NMD) and the European Reference Network for rare neuromuscular diseases EURO-NMD. PJS is supported as an NIHR Senior Investigator and by the Sheffield NIHR Biomedical Research Centre. This is in part an EU Joint Programme - Neurodegenerative Disease Research (JPND) project. The project is supported through the following funding organisations under the aegis of JPND - www.jpnd.eu (United Kingdom, Medical Research Council (MR/L501529/1; MR/R024804/1) and Economic and Social Research Council (ES/L008238/1)) and through the Motor Neurone Disease Association. This study represents independent research part funded by the National Institute for Health Research (NIHR) Biomedical Research Centre at South London and Maudsley NHS Foundation Trust and King’s College London. A.A-C is supported by an NIHR Senior Investigator Award. Samples used in this research were in part obtained from the UK National DNA Bank for MND Research, funded by the MND Association and the Wellcome Trust. We would like to thank people with MND and their families for their participation in this project. We acknowledge sample management undertaken by Biobanking Solutions funded by the Medical Research Council at the Centre for Integrated Genomic Medical Research, University of Manchester. L.H.v.d.B. reports grants from The Netherlands Organization for Health Research and Development (Vici scheme), grants from The European Community's Health Seventh Framework Programme (grant agreement n° 259867 (EuroMOTOR)), grants from The Netherlands Organization for Health Research and Development) the STRENGTH project, funded through the EU Joint Programme – Neurodegenerative Disease Research, JPND). This project has received funding from the European Research Council (ERC) under the European Union's Horizon 2020 research and innovation programme (grant agreement n° 772376 – EScORIAL). The collaboration project is co-funded by the PPP Allowance made available by Health~Holland, Top Sector Life Sciences & Health, to stimulate public-private partnerships. This study was supported by the ALS Foundation Netherlands.

The authors thank the authors of the GWAS in AD, PD, PSP, FTD, CBD, the International Psychiatric Psychiatric Genetics Consortium, MEGASTROKE consortium, International Stroke Genetics Consortium intracranial aneurysm working group, and International Multiple Sclerosis Genetics Consortium for sharing their GWAS summary statistics. Summary statistics for ischemic stroke, intracerebral hemorrhage and intracranial aneurysm were accessed through the ISGC Cerebrovascular Disease Knowledge Portal.

Supplementary Figures

Supplementary Figures 1-15. Track plots for gene prioritization. Overview of genome-wide significant loci. The locus boundaries are defined as distance to the furthest SNPs with LD $r^2 \geq 0.2$ according to Central European samples from 1000 Genomes phase 3, or 250 Kb on both sides, whichever is greater. The track plots display the following data from top to bottom: 1) karyogram of the chromosome and chromosomal position of the locus. 2) ALS GWAS association statistics obtained from the two-tailed SAIGE logistic mixed model regression in the European + Asian ancestries meta-analysis. Colors illustrate LD with the lead SNP, as calculated in stratum 4. Dotted line is the genome-wide significance threshold of $P = 5 \times 10^{-8}$. 3) GWAS association statistics of other neurodegenerative diseases: Alzheimer's disease (AD)[28], corticobasal degeneration (CBD)[29], frontotemporal dementia (FTD)[30], Parkinson's disease (PD)[31] and progressive supranuclear palsy (PSP)[32]. Dotted line: $P = 5 \times 10^{-8}$. 4) Association statistics obtained by FDR-corrected two-tailed Firth logistic regression of repeat size called by ExpansionHunter v4 (ref [33]) or ExpansionHunter Denovo[22]. Dotted line is the Bonferroni multiple testing threshold for the number of repeats tested in the locus. 5) Summary-based Mendelian Randomization (SMR[34]) two-sided P-value of methylation quantitative trait loci (mQTL). mQTL effects were obtained from brain tissue and blood. Dotted line is the Bonferroni threshold of number of mQTL effects (brain and blood) tested in the locus. 5) Genetrack obtained from the UCSC browser. 6) Two-tailed Firth regression P-values for the rare variant gene-based burden analysis. Dotted line is the Bonferroni threshold for the number of genes tested within the locus. 7) eQTL-based SMR two-sided P-values. eQTLs obtained from blood (eQTLGen) and brain (metaBrain Cortex) were used. Dotted line is the Bonferroni threshold for the number of genes times the number of tissues these genes were tested in. Genes depicted in the shaded area were available in the eQTL dataset, but no eQTLs were strong enough to be included in the SMR analysis.

Supplementary figure 1. Trackplot rs631312, *MOBP/RPSA.* Within this locus no genes were enriched for rare variants that contributed to ALS risk and no repeat expansions associated with ALS risk were found. The ALS-associated SNPs within the rs631312 locus are predicted to mediate risk of ALS through decreased expression of *CCR8*, or increased expression of *RPSA* and/or *MOBP* in brain tissues. Additionally, three significant CpG sites identified through SMR are predicted to regulate expression of *RPSA*, *MOBP* and *SNORA6A2*, all in blood. Number of tests: SMR eQTL = 14 gene tissue pairs, SMR mQTL = 48 CpG tissue pairs, rare variant burden = 4 genes, repeat expansion = 69 repeat loci.
[figure on next page]

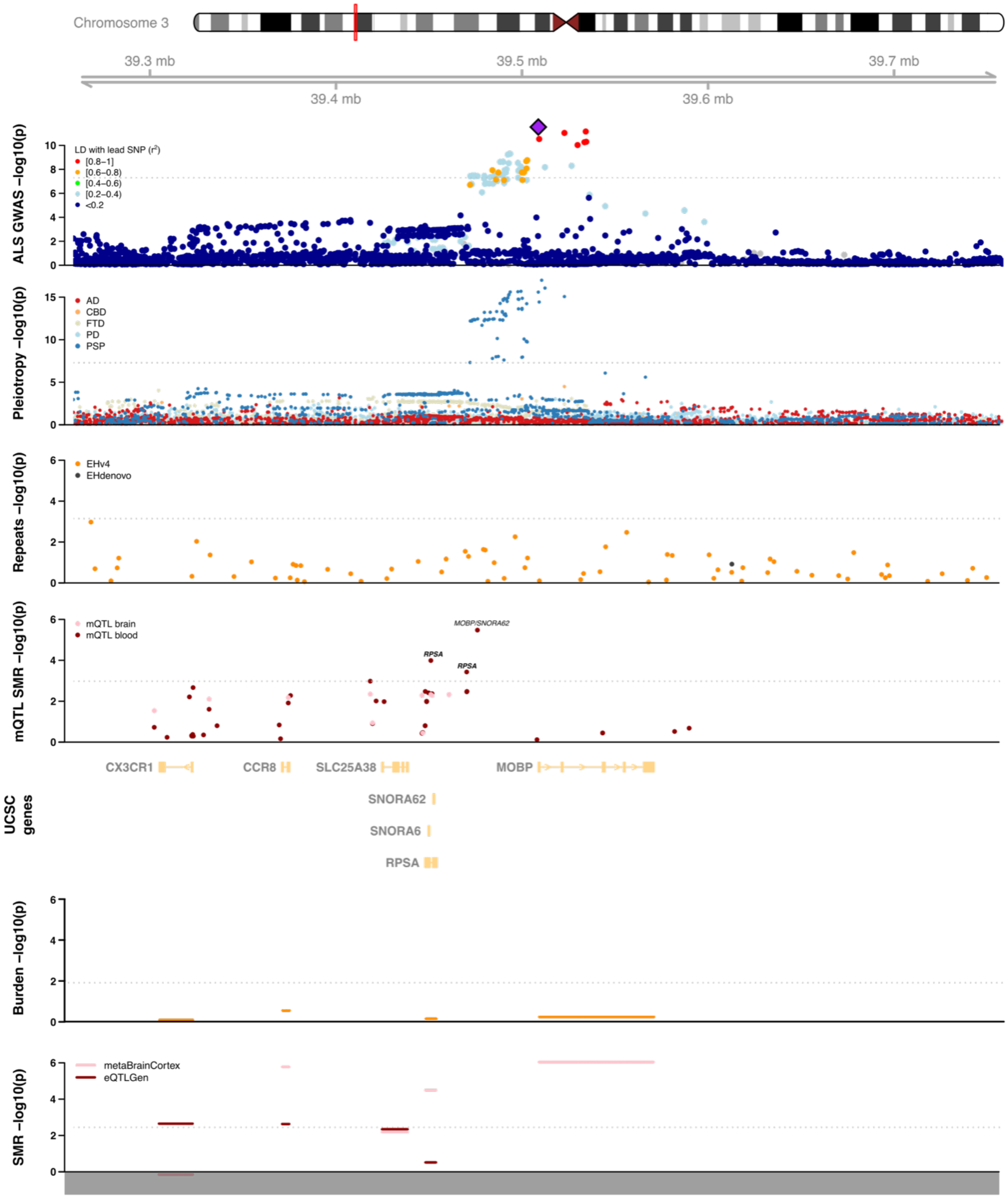
Chromosome 3
39.3 mb
39.4 mb
39.5 mb
39.6 mb
39.7 mb
ALS GWAS −log10(p)
LD with lead SNP (r2)
[0.8–1]
[0.6–0.8)
[0.4–0.6)
[0.2–0.4)
<0.2
Pleiotropy −log10(p)
AD
CBD
FTD
PD
PSP
Repeats −log10(p)
EHv4
EHdenovo
mQTL SMR −log10(p)
mQTL brain
mQTL blood
MOBP/SNORA62
RPSA
RPSA
CX3CR1
CCR8
SLC25A38
MOBP
SNORA62
SNORA6
RPSA
UCSC genes
Burden −log10(p)
SMR −log10(p)
metaBrainCortex
eQTLGen

Supplementary figure 2. Trackplot for rs62333164, *NEK1.* *NEK1* is a known ALS risk gene in this locus[24] and we find rare variants in *NEK1* to increase ALS risk. An exome-wide significant rare variant burden is found for the variant sets including disruptive, and disruptive + damaging variants, for both MAF thresholds. This rare variant burden signal is conditionally independent from the common variant signal (P_{RVB} = 2.53 × 10^{-7}, $P_{RVB|SNP}$ = 2.32 × 10^{-7}). Additionally, an expansion of the tetranucleotide repeat chr4:17030384$[TTTA]_n$ downstream of *NEK1* with more than 10 repeat units is associated with ALS risk (expanded allele-frequency = 0.51, P_{Repeat} = 5.2 × 10^{-5}, FDR = 4.7 × 10^{-4}). This repeat expansion is tagged by the top associated GWAS SNP within this locus and therefore does not represent a fully independent signal ($P_{Repeat|SNP}$ = 0.003, r^2 = 0.24, |D'| = 0.70). Besides the rare variant burden signal and repeat expansion, SMR prioritized *NEK1* as the causal gene through both eQTL and mQTL. SMR showed that reduced levels of NEK1 in cortex are associated with increased risk of ALS, while this effect is opposite and weaker in blood. For both the eQTL and mQTL signals the HEIDI pleiotropy test indicated nominal evidence for pleiotropy ($P < 0.05$, but $P > 0.05$/number of genes). Number of tests: SMR eQTL = 14 gene tissue pairs, SMR mQTL = 53 CpG tissue pairs, rare variant burden = 6 genes, repeat expansion = 125 repeat loci.
[figure on next page]

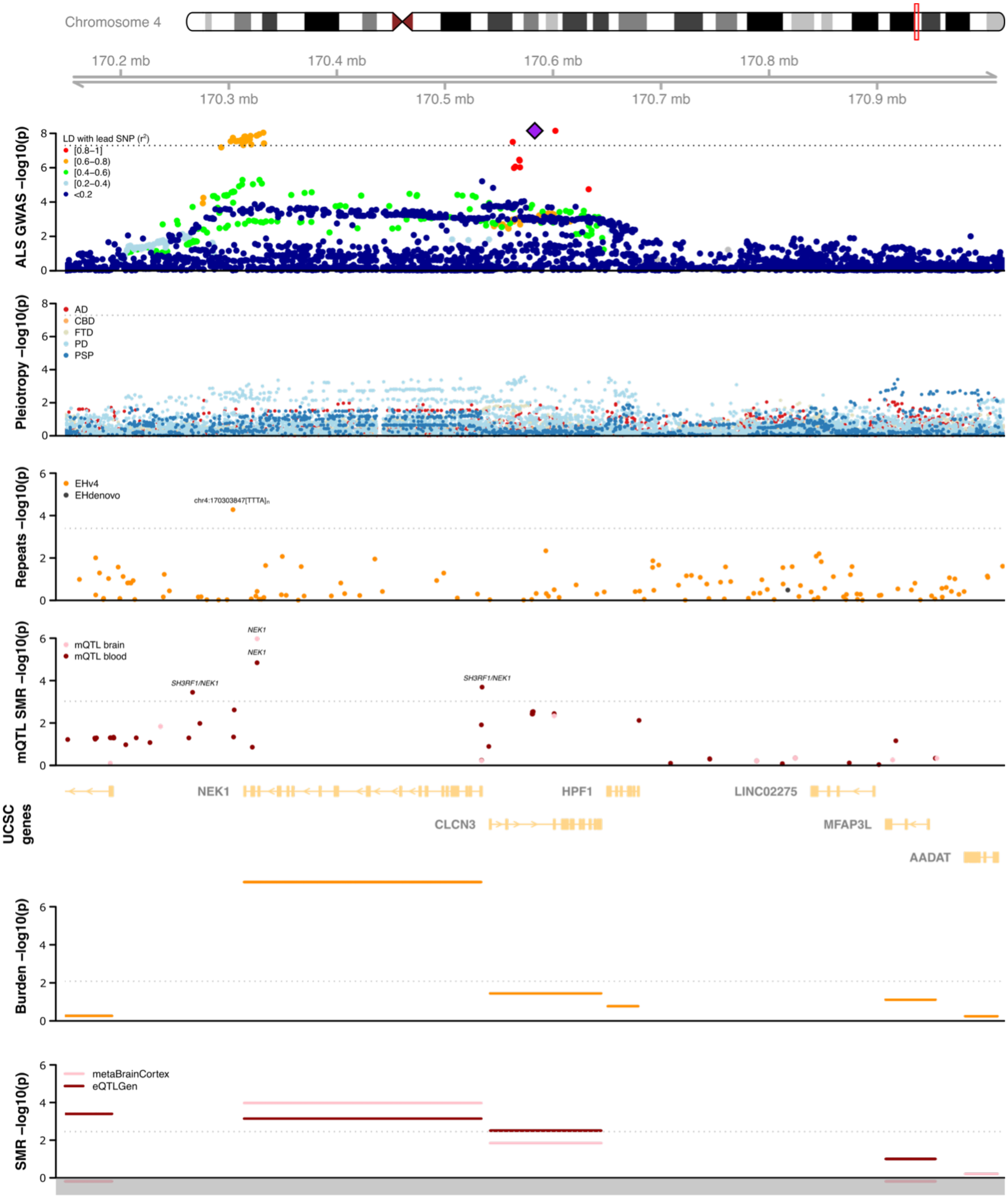

Chromosome 4
170.2 mb
170.4 mb
170.6 mb
170.8 mb
170.3 mb
170.5 mb
170.7 mb
170.9 mb
ALS GWAS −log10(p)
LD with lead SNP (r²)
[0.8–1]
[0.6–0.8)
[0.4–0.6)
[0.2–0.4)
<0.2
Pleiotropy −log10(p)
AD
CBD
FTD
PD
PSP
Repeats −log10(p)
EHv4
EHdenovo
chr4:170303847[TTTA]n
mQTL SMR −log10(p)
mQTL brain
mQTL blood
NEK1
NEK1
SH3RF1/NEK1
SH3RF1/NEK1
UCSC genes
NEK1
HPF1
LINC02275
CLCN3
MFAP3L
AADAT
Burden −log10(p)
SMR −log10(p)
metaBrainCortex
eQTLGen

Supplementary figure 3. Trackplot for rs7727034, *GPX3*/*TNIP1*. In the rs7727034 locus, several mQTL effects were found which, based on annotation, were predicted to affect both *TNIP1* and *GPX3*, blood prioritized both *TNIP1* and *GPX3* as well. Brain-specific eQTL, however, prioritized *GPX3* over *TNIP1*. Higher levels of *GPX3* were associated with an increased risk of ALS. None of the genes within this locus harbored rare variants that were associated with ALS risk, neither were any of the repeats within this locus. Number of tests: SMR eQTL = 22 gene tissue pairs, SMR mQTL = 76 CpG tissue pairs, rare variant burden = 9 genes, repeat expansion = 60 repeat loci.
[figure on next page]

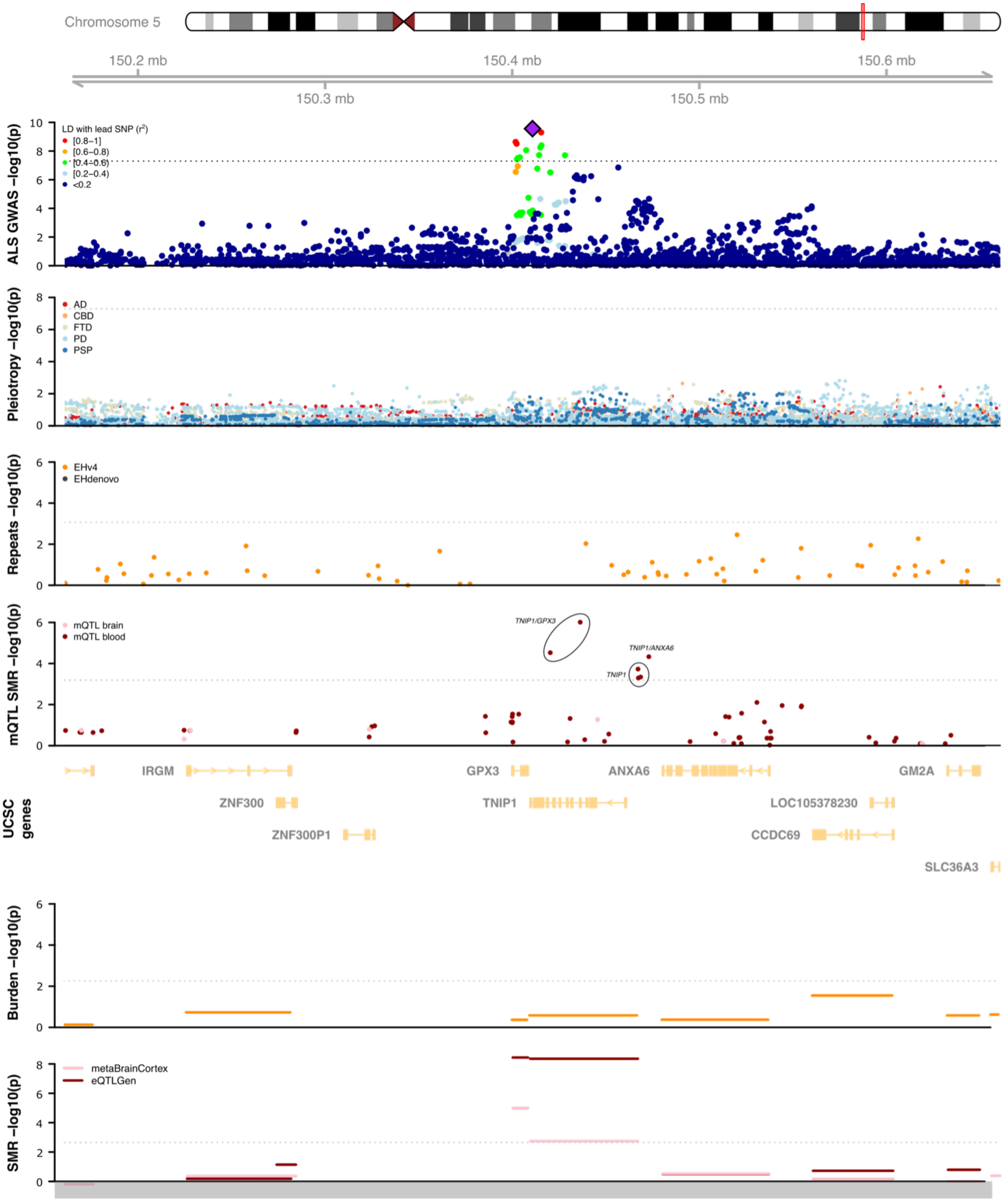
Chromosome 5
150.2 mb
150.3 mb
150.4 mb
150.5 mb
150.6 mb
ALS GWAS −log10(p)
LD with lead SNP (r²)
[0.8−1]
[0.6−0.8)
[0.4−0.6)
[0.2−0.4)
<0.2
Pleiotropy −log10(p)
AD
CBD
FTD
PD
PSP
Repeats −log10(p)
EHv4
EHdenovo
mQTL SMR −log10(p)
mQTL brain
mQTL blood
TNIP1/GPX3
TNIP1/ANXA6
TNIP1
UCSC genes
IRGM
ZNF300
ZNF300P1
GPX3
TNIP1
ANXA6
LOC105378230
CCDC69
GM2A
SLC36A3
Burden −log10(p)
SMR −log10(p)
metaBrainCortex
eQTLGen

Supplementary figure 4. Trackplot for rs517339, *ERGIC1.* Within the rs517339 locus we found no genes where rare variants were associated with an increased risk of ALS. The mQTL analysis, however, prioritized *ERGIC1* with signals in both blood and cortex, and eQTL analysis nominates *ERGIC1, CREBRF* and *BNIP1*, all from signals in blood. For all three genes, increased levels in blood were associated with an increased risk of ALS. No ALS-associated repeats were found. Number of tests: SMR eQTL = 22 gene tissue pairs, SMR mQTL = 102 CpG tissue pairs, rare variant burden = 6 genes, repeat expansion = 96 repeat loci.

[figure on next page]

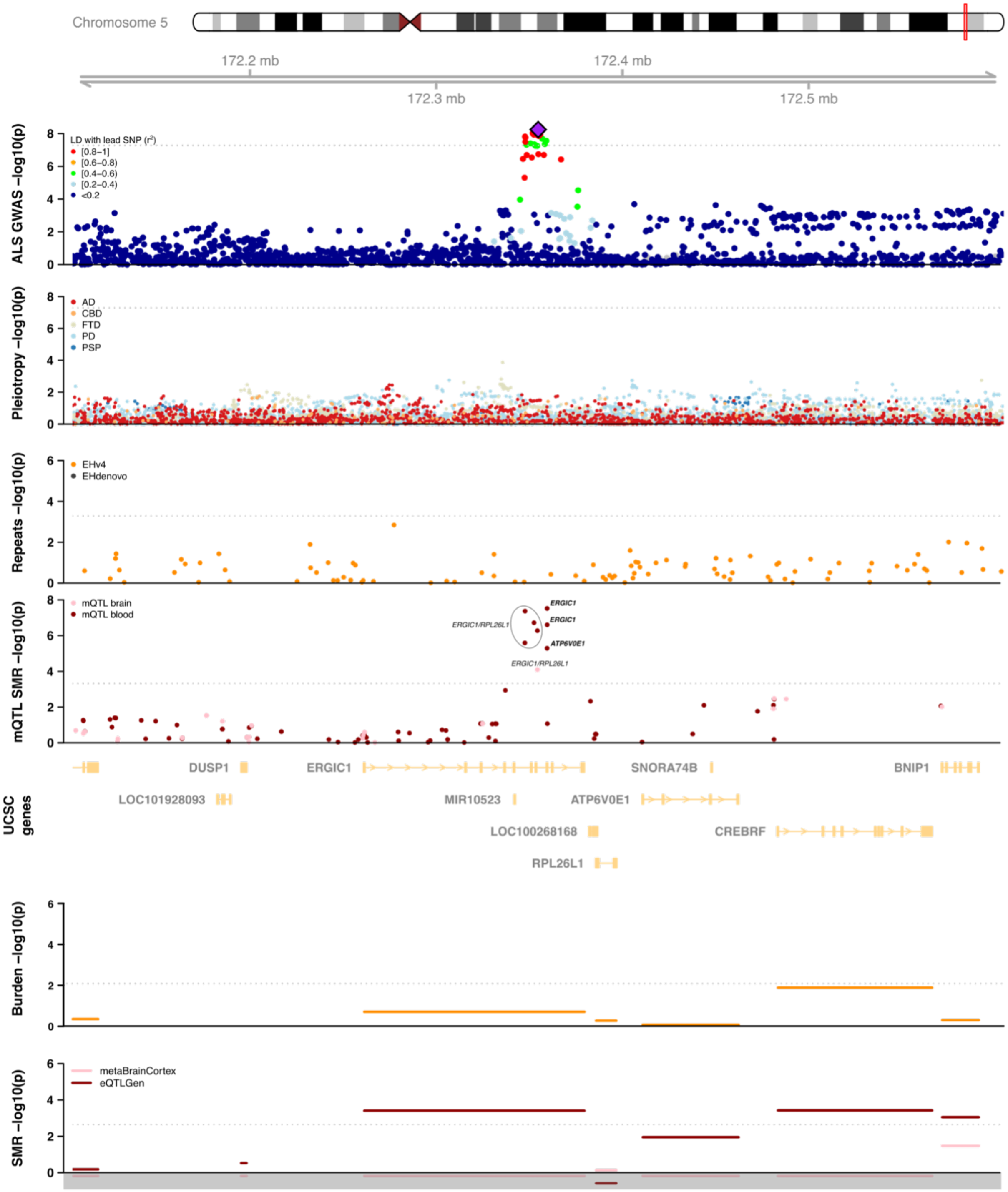

Chromosome 5
172.2 mb
172.4 mb
172.3 mb
172.5 mb
ALS GWAS −log10(p)
LD with lead SNP (r²)
[0.8–1]
[0.6–0.8)
[0.4–0.6)
[0.2–0.4)
<0.2
Pleiotropy −log10(p)
AD
CBD
FTD
PD
PSP
Repeats −log10(p)
EHv4
EHdenovo
mQTL SMR −log10(p)
mQTL brain
mQTL blood
ERGIC1
ERGIC1
ERGIC1/RPL26L1
ATP6V0E1
ERGIC1/RPL26L1
UCSC genes
DUSP1
ERGIC1
SNORA74B
BNIP1
LOC101928093
MIR10523
ATP6V0E1
LOC100268168
CREBRF
RPL26L1
Burden −log10(p)
SMR −log10(p)
metaBrainCortex
eQTLGen

Supplementary figure 5. Trackplot for rs9275477, *HLA.* The HLA locus, with rs9275477 as lead SNP contains many genes, and long-range LD. Neither rare variants, repeat expansions, nor regulatory effects through eQTL and mQTL prioritize a gene within this locus. Number of tests: SMR eQTL = 220 gene tissue pairs, SMR mQTL = 1,488 CpG tissue pairs, rare variant burden = 77 genes, repeat expansion = 403 repeat loci.
[figure on next page]

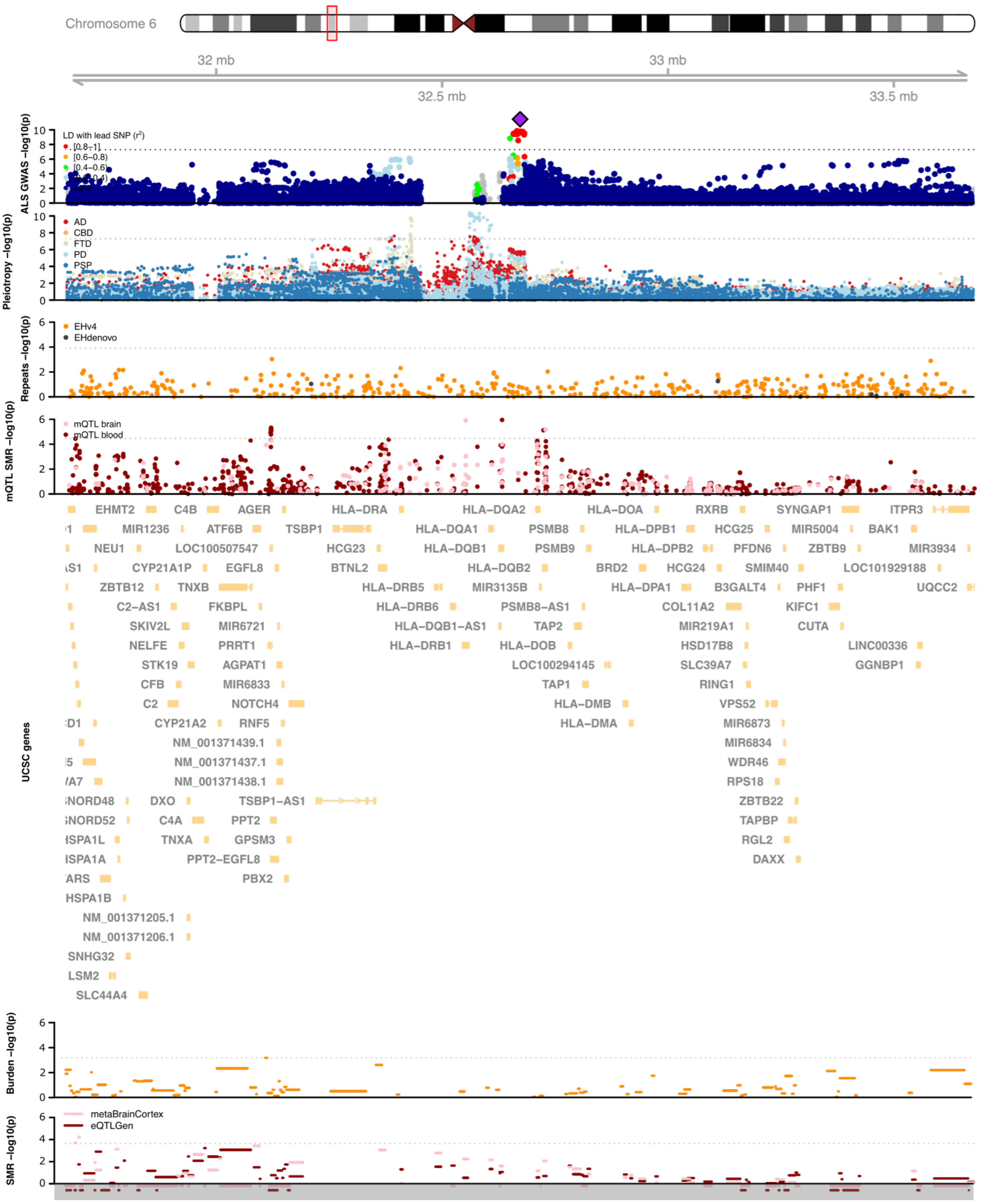
Chromosome 6
32 mb
33 mb
32.5 mb
33.5 mb
ALS GWAS −log10(p)
LD with lead SNP (r2)
Pleiotropy −log10(p)
AD
CBD
FTD
PD
PSP
Repeats −log10(p)
EHv4
EHdenovo
mQTL SMR −log10(p)
mQTL brain
mQTL blood
UCSC genes
Burden −log10(p)
SMR −log10(p)
metaBrainCortex
eQTLGen

Supplementary figure 6. Trackplot for rs10280711, *PTPRN2.* The rs10280711 locus only contains a single protein-coding gene, *PTPRN2*. For *PTPRN2* we observe a significant eQTL effect in blood, showing a reduced expression of *PTPRN2* associated with an increased risk of ALS. The rare variant burden for missense variants at MAF < 0.005 was nominally significant (P_{RVB} = 0.038). Previous biomarker screens have identified lower levels of PTPRN2 in cerebrospinal fluid of patients with FTD and *GRN* mutations[35] and patients with AD[36]. An epigenome-wide methylation study in Parkinson's disease identified hypomethylation of CpG sites in *PTPRN2* as a marker for motor decline[37]. Number of tests: SMR eQTL = 4 gene tissue pairs, SMR mQTL = 191 CpG tissue pairs, rare variant burden = 1 genes, repeat expansion = 81 repeat loci.
[figure on next page]

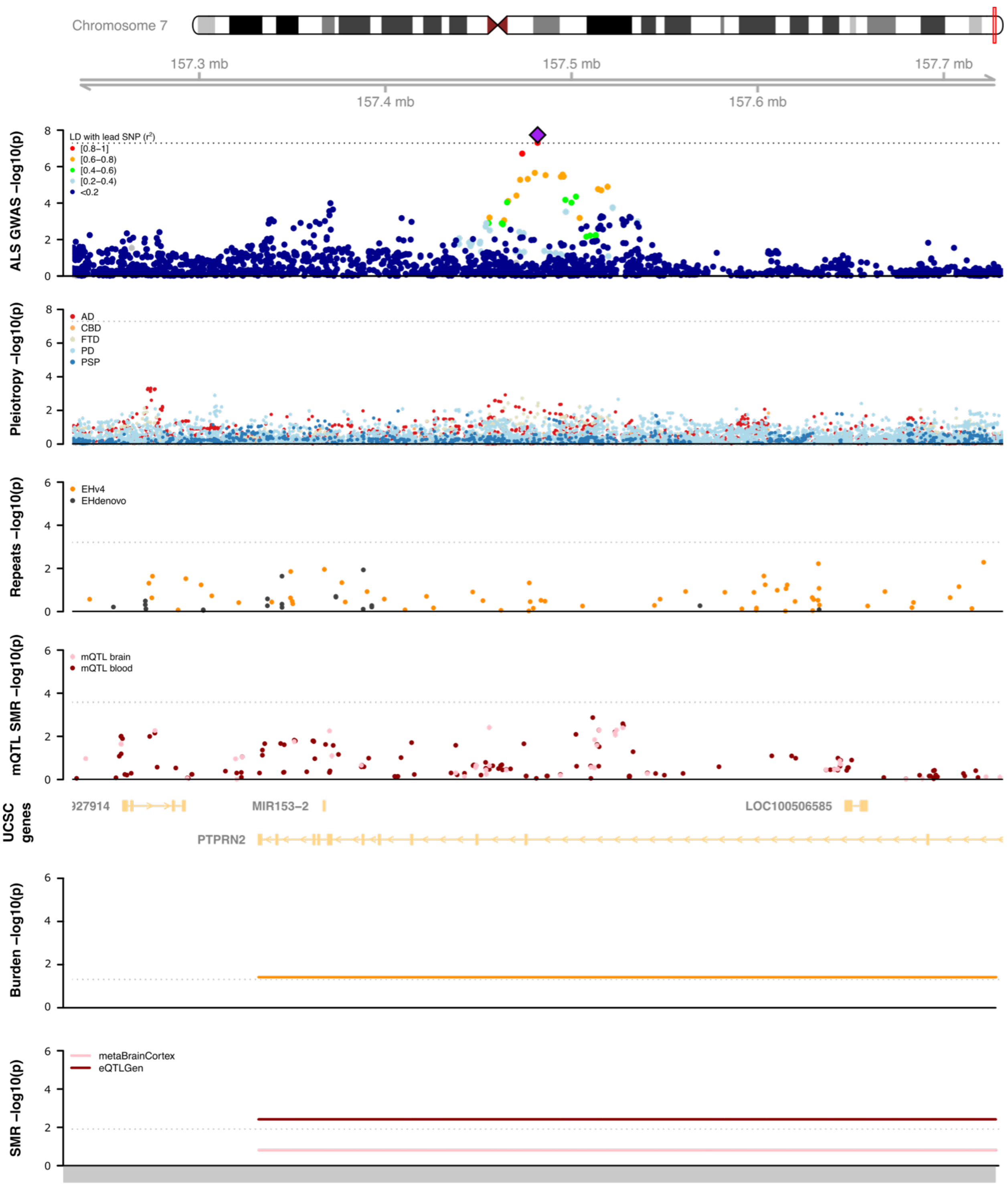
Chromosome 7
157.3 mb
157.4 mb
157.5 mb
157.6 mb
157.7 mb
ALS GWAS −log10(p)
LD with lead SNP (r²)
[0.8−1]
[0.6−0.8)
[0.4−0.6)
[0.2−0.4)
<0.2
Pleiotropy −log10(p)
AD
CBD
FTD
PD
PSP
Repeats −log10(p)
EHv4
EHdenovo
mQTL SMR −log10(p)
mQTL brain
mQTL blood
UCSC genes
MIR153−2
LOC100506585
PTPRN2
Burden −log10(p)
SMR −log10(p)
metaBrainCortex
eQTLGen

Supplementary figure 7. Trackplot for rs2453555, *C9orf72.* The rs2453555 SNP tags the pathogenic hexanucleotide repeat expansion in *C9orf72* which is the most common cause of ALS with or without frontotemporal dementia. This repeat expansion is detected by ExpansionHunter v4 since it is included in the catalogue, and also found through de-novo assembly of unmapped reads with ExpansionHunter De-novo. The repeat expansion is in high LD with the top SNP (r^2 = 0.14, |D'| = 0.99) and after conditioning on the repeat expansions, there is no residual association signal within the whole-genome sequencing cohort for the top SNP (P_{SNP} = 0.005, $P_{SNP|Repeat}$ = 0.86). Additionally, eQTL SMR analysis highlighted a potential effect of *C9orf72* gene expression, but the HEIDI test indicated strong evidence for heterogeneity ($P_{HEIDI} = 3.72 \times 10^{-23}$) suggesting horizontal pleiotropy and that the causal effect is not mediated through the SNP eQTL effect. An identical pattern was seen for mQTL SMR (minimal $P_{HEIDI} = 4.1 \times 10^{-7}$). Number of tests: SMR eQTL = 8 gene tissue pairs, SMR mQTL = 21 CpG tissue pairs, rare variant burden = 3 genes, repeat expansion = 63 repeat loci.
[figure on next page]

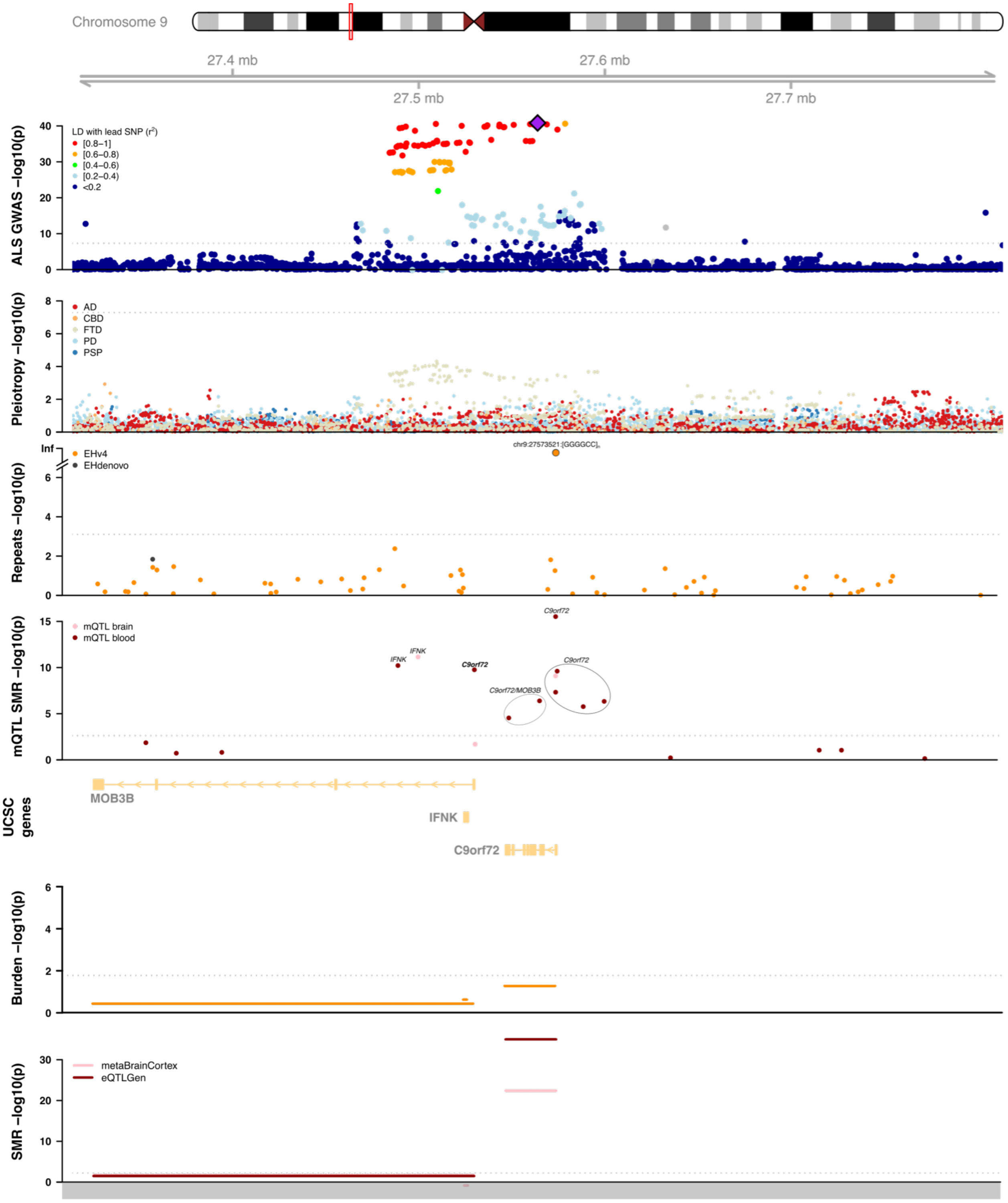

Chromosome 9
27.4 mb
27.6 mb
27.5 mb
27.7 mb
ALS GWAS −log10(p)
LD with lead SNP (r²)
[0.8−1]
[0.6−0.8)
[0.4−0.6)
[0.2−0.4)
<0.2
Pleiotropy −log10(p)
AD
CBD
FTD
PD
PSP
Repeats −log10(p)
Inf
EHv4
EHdenovo
chr9:27573521:[GGGGCC]n
mQTL SMR −log10(p)
mQTL brain
mQTL blood
C9orf72
IFNK
C9orf72/MOB3B
UCSC genes
MOB3B
Burden −log10(p)
SMR −log10(p)
metaBrainCortex
eQTLGen

Supplementary figure 8. Trackplot for rs113247976, *KIF5A.* The rs113247976 SNP is a coding low-frequency variant in *KIF5A* (P986L, frequency = 0.0128 in HRC). There are few associated low-frequency SNPs in LD. Loss-of-function mutations in the cargo-binding tail domain of *KIF5A* have previously been identified in familial ALS, which together with the associated SNP identified *KIF5A* as ALS risk gene[38]. We do not find an overall increased burden of rare variants in *KIF5A* in patients in our sequencing cohort, which consists of mostly apparently sporadic ALS patients. We find no evidence for repeat expansions or regulatory effects contributing to disease risk. Number of tests: SMR eQTL = 130 gene tissue pairs, SMR mQTL = 206 CpG tissue pairs, rare variant burden = 48 genes, repeat expansion = 344 repeat loci.
[figure on next page]

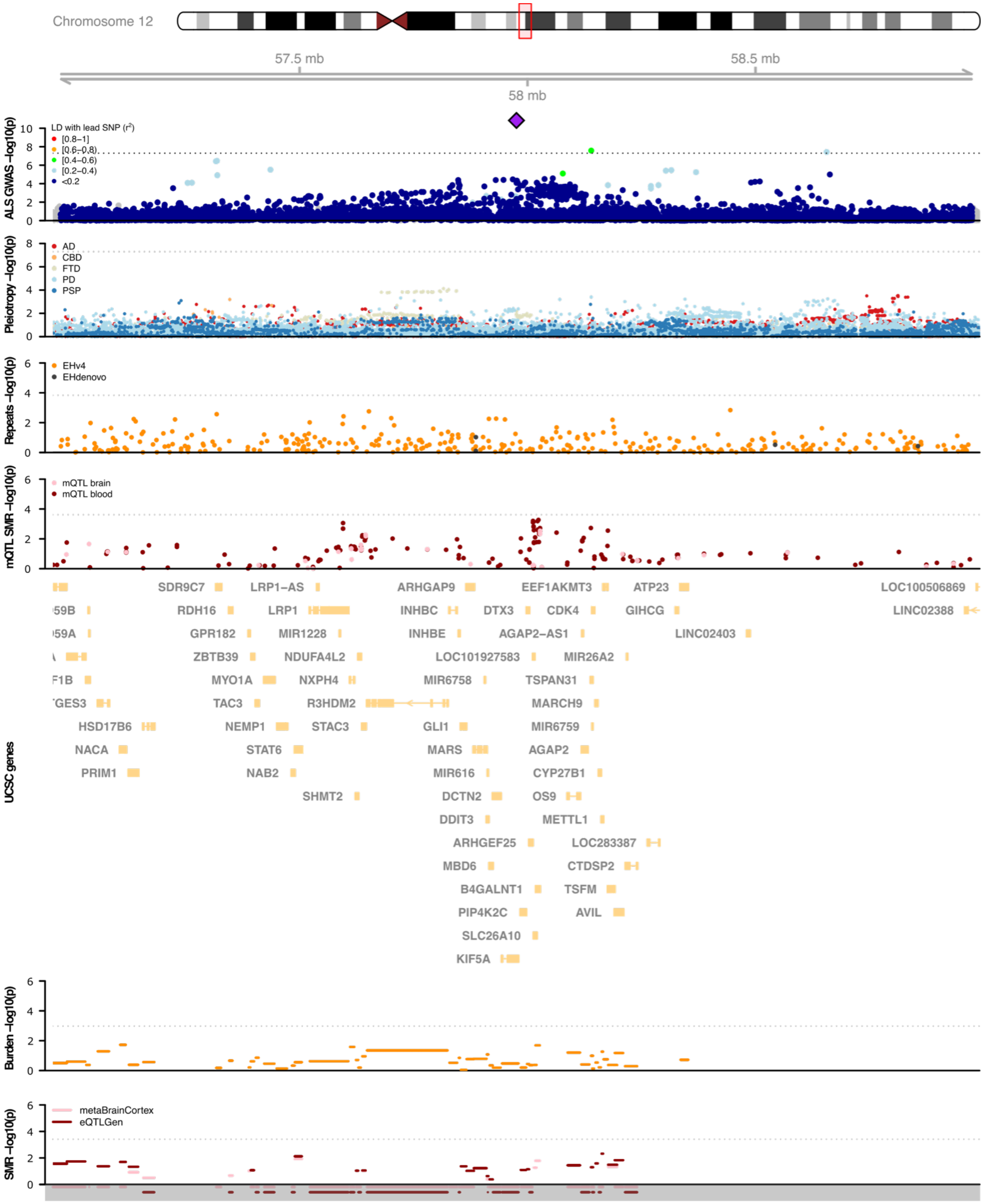
Chromosome 12
57.5 mb
58 mb
58.5 mb
ALS GWAS −log10(p)
LD with lead SNP (r²)
[0.8–1]
[0.6–0.8)
[0.4–0.6)
[0.2–0.4)
<0.2
Pleiotropy −log10(p)
AD
CBD
FTD
PD
PSP
Repeats −log10(p)
EHv4
EHdenovo
mQTL SMR −log10(p)
mQTL brain
mQTL blood
UCSC genes
SDR9C7
LRP1−AS
ARHGAP9
EEF1AKMT3
ATP23
LOC100506869
RDH16
LRP1
INHBC
DTX3
CDK4
GIHCG
LINC02388
GPR182
MIR1228
INHBE
AGAP2−AS1
LINC02403
ZBTB39
NDUFA4L2
LOC101927583
MIR26A2
MYO1A
NXPH4
MIR6758
TSPAN31
TAC3
R3HDM2
MARCH9
HSD17B6
NEMP1
STAC3
GLI1
MIR6759
NACA
STAT6
MARS
AGAP2
PRIM1
NAB2
MIR616
CYP27B1
SHMT2
DCTN2
OS9
DDIT3
METTL1
ARHGEF25
LOC283387
MBD6
CTDSP2
B4GALNT1
TSFM
PIP4K2C
AVIL
SLC26A10
KIF5A
Burden −log10(p)
SMR −log10(p)
metaBrainCortex
eQTLGen

Supplementary figure 9. Trackplot for rs4075094, *TBK1.* The *TBK1* gene is a known ALS gene within this locus. It was identified in a whole-exome sequencing case-control study in ALS[39] and loss-of-function mutations are associated with familial ALS[40]. Here, we find the rare variant burden for *TBK1* to be associated with ALS risk ($P_{RVB} = 6.1 \times 10^{-3}$) just passing the threshold for multiple testing across all genes within this locus ($0.05/8 = 6.25 \times 10^{-3}$). The lowest P-value is reached for rare variant burden models 13 and 14, meaning all non-synonymous (disruptive, damaging and missense) variants, for both MAF thresholds. The conditional analysis in all individuals with whole-genome sequencing and GWAS data indicated that the rare-variant burden is conditionally independent from the GWAS signal ($P_{RVB} = 0.021$, $P_{RVB|SNP} = 0.021$). SMR identified CpG sites that were associated with *TBK1* expression suggesting that the regulatory effects on *TBK1* contribute to ALS risk independent from the known ALS associated rare variants. The SMR eQTL analysis in blood predicted a risk increasing effect of higher *TBK1* expression which is in line with the mQTL analysis that indicated a risk increasing effect of hypomethylation. The HEIDI pleiotropy test showed nominal evidence for pleiotropy of the SMR signal ($P < 0.05$, but $P > 0.05$/number of genes). Number of tests: SMR eQTL = 22 gene tissue pairs, SMR mQTL = 47 CpG tissue pairs, rare variant burden = 8 genes, repeat expansion = 154 repeat loci.
[figure on next page]

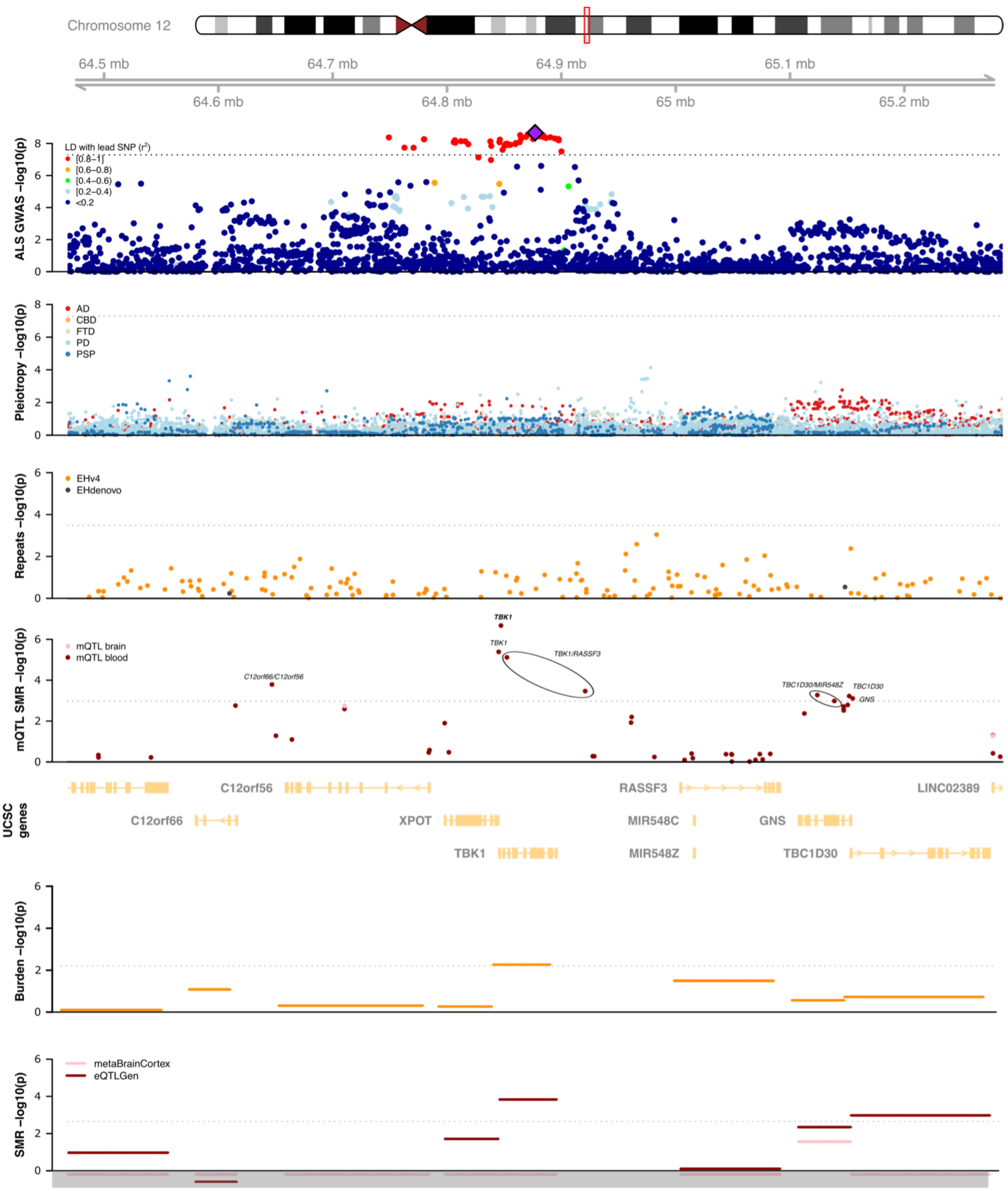
Chromosome 12
64.5 mb
64.7 mb
64.9 mb
65.1 mb
64.6 mb
64.8 mb
65 mb
65.2 mb
ALS GWAS −log10(p)
LD with lead SNP (r²)
[0.8−1]
[0.6−0.8)
[0.4−0.6)
[0.2−0.4)
<0.2
Pleiotropy −log10(p)
AD
CBD
FTD
PD
PSP
Repeats −log10(p)
EHv4
EHdenovo
mQTL SMR −log10(p)
mQTL brain
mQTL blood
TBK1
TBK1
TBK1/RASSF3
C12orf66/C12orf56
TBC1D30/MIR548Z
TBC1D30
GNS
UCSC genes
C12orf56
RASSF3
LINC02389
C12orf66
XPOT
MIR548C
GNS
TBK1
MIR548Z
TBC1D30
Burden −log10(p)
SMR −log10(p)
metaBrainCortex
eQTLGen

Supplementary figure 10. Trackplot for rs2985994, *COG3.* Within the rs2985994 locus, no genes exhibit a rare variant burden signal and we found no ALS-associated repeats. eQTL SMR in cortical tissues identified *COG3* as the most likely causal gene, with reduced expression leading to an increased risk of ALS (b_{xy} = -0.239, SE = 0.070, P = 5.15×10^{-4}). No evidence for heterogeneity was found by the HEIDI test (P = 0.28). The two CpG sites identified through SMR in blood methylation profiles were predicted to regulate *COG3* and/or *ERICH6B.* These analyses combined, prioritize *COG3* within the rs2985994 locus. Number of tests: SMR eQTL = 14 gene tissue pairs, SMR mQTL = 47 CpG tissue pairs, rare variant burden = 6 genes, repeat expansion = 84 repeat loci.
[figure on next page]

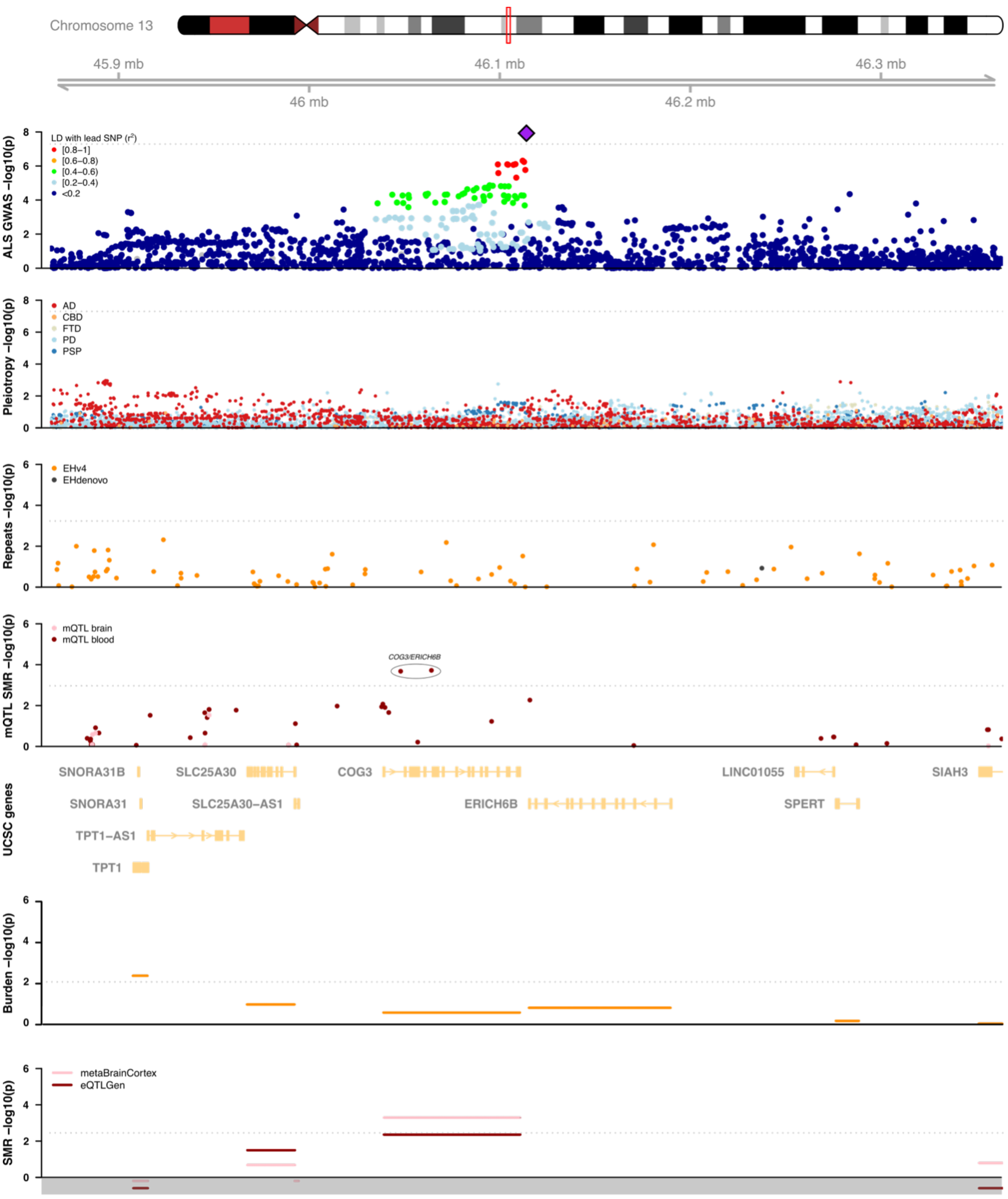
Chromosome 13
45.9 mb
46.1 mb
46.3 mb
46 mb
46.2 mb
ALS GWAS −log10(p)
LD with lead SNP (r²)
[0.8–1]
[0.6–0.8)
[0.4–0.6)
[0.2–0.4)
<0.2
Pleiotropy −log10(p)
AD
CBD
FTD
PD
PSP
Repeats −log10(p)
EHv4
EHdenovo
mQTL SMR −log10(p)
mQTL brain
mQTL blood
COG3/ERICH6B
UCSC genes
SNORA31B
SLC25A30
COG3
LINC01055
SIAH3
SNORA31
SLC25A30−AS1
ERICH6B
SPERT
TPT1−AS1
TPT1
Burden −log10(p)
SMR −log10(p)
metaBrainCortex
eQTLGen

Supplementary figure 11. Trackplot for rs229243, *SCFD1.* Within the rs229195 locus the associated SNPs span both *G2E3* and *SCFD1*. Rare variant burden analyses show no evidence for a rare variant signal in either two of these genes. There are no repeat expansions associated with ALS within this locus. mQTL SMR identified expression-altering CpG sites for both *G2E3* and *SCFD1* in blood and cortex, with the strongest (brain-specific) signal for *SCFD1*. Similarly, locus-wide significant eQTL SMR effects were found for both genes in both tissues, with a stronger signal for *SCFD1* than for *G2E3*. Increased levels of both genes were associated with an increased risk of ALS. Taken together, these analyses prioritize *SCFD1* over *G2E3.* Number of tests: SMR eQTL = 2 gene tissue pairs, SMR mQTL = 15 CpG tissue pairs, rare variant burden = 2 genes, repeat expansion = 64 repeat loci.
[figure on next page]

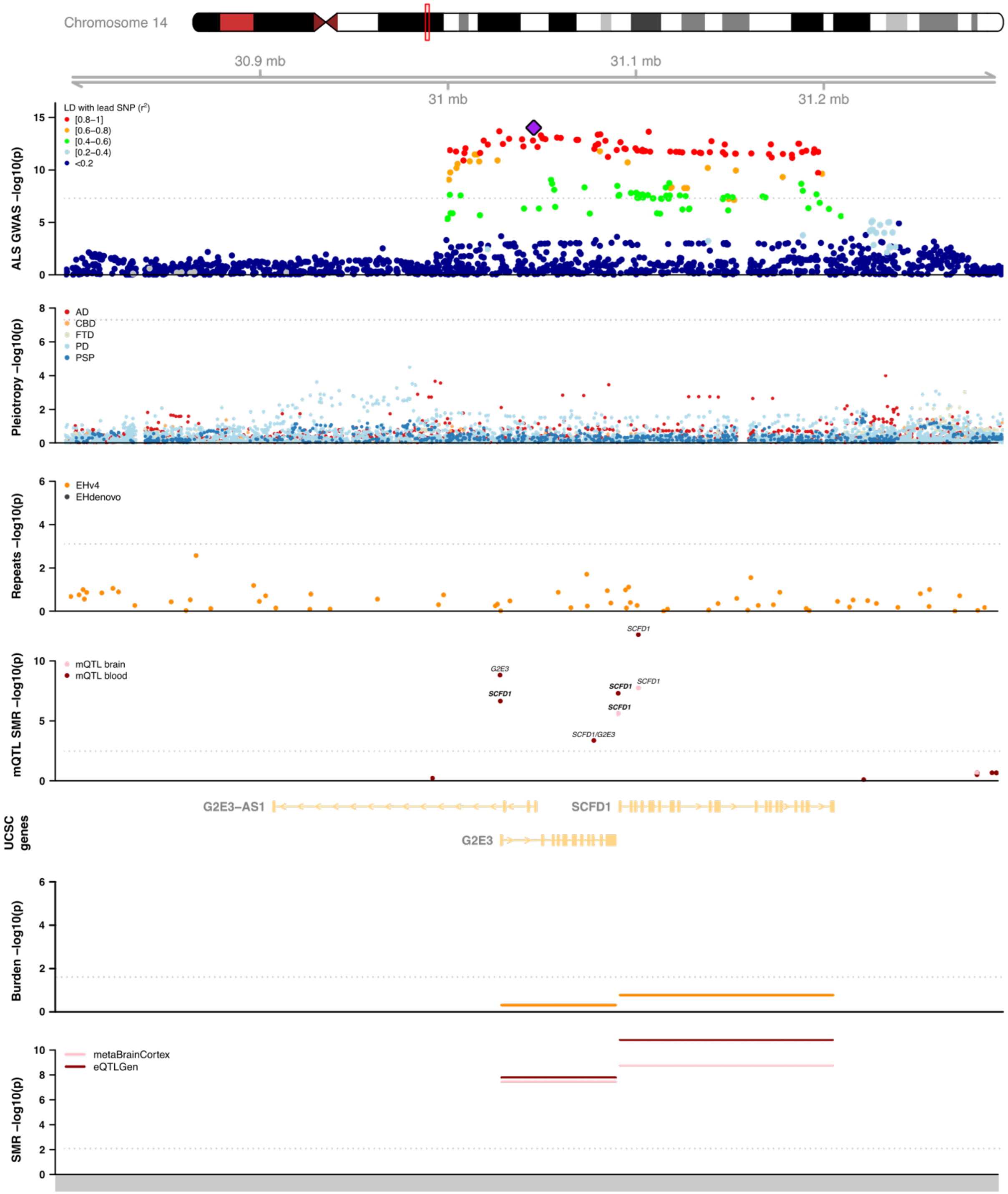

Chromosome 14
30.9 mb
31.1 mb
31 mb
31.2 mb
LD with lead SNP (r^2)
[0.8–1]
[0.6–0.8)
[0.4–0.6)
[0.2–0.4)
<0.2
ALS GWAS −log10(p)
AD
CBD
FTD
PD
PSP
Pleiotropy −log10(p)
EHv4
EHdenovo
Repeats −log10(p)
mQTL brain
mQTL blood
mQTL SMR −log10(p)
SCFD1
G2E3
SCFD1/G2E3
UCSC genes
G2E3−AS1
G2E3
Burden −log10(p)
metaBrainCortex
eQTLGen
SMR −log10(p)

Supplementary figure 12. Trackplot for rs12608932, *UNC13A.* The rs12608932 locus was one of the first robustly associated GWAS loci in ALS together with the *C9orf72* signal[41] and has been identified in FTD as well[42]. There is little LD with the top SNP in this region and the associated SNPs are all intronic variants within the *UNC13A* gene. We found no evidence of ALS-associated rare variants or repeat expansions within this region. The ALS-associated SNPs do not act as strong eQTL or mQTL for any of the genes or CgG sites in this region. Recent studies suggest that the ALS associated SNPs act as splice-QTL conditionally on TDP-43 dysfunction that lead to inclusion of a cryptic exon and potentially nonsense mediated decay of UNC13A[43,44]. Number of tests: SMR eQTL = 42 gene tissue pairs, SMR mQTL = 114 CpG tissue pairs, rare variant burden = 16 genes, repeat expansion = 188 repeat loci.
[figure on next page]

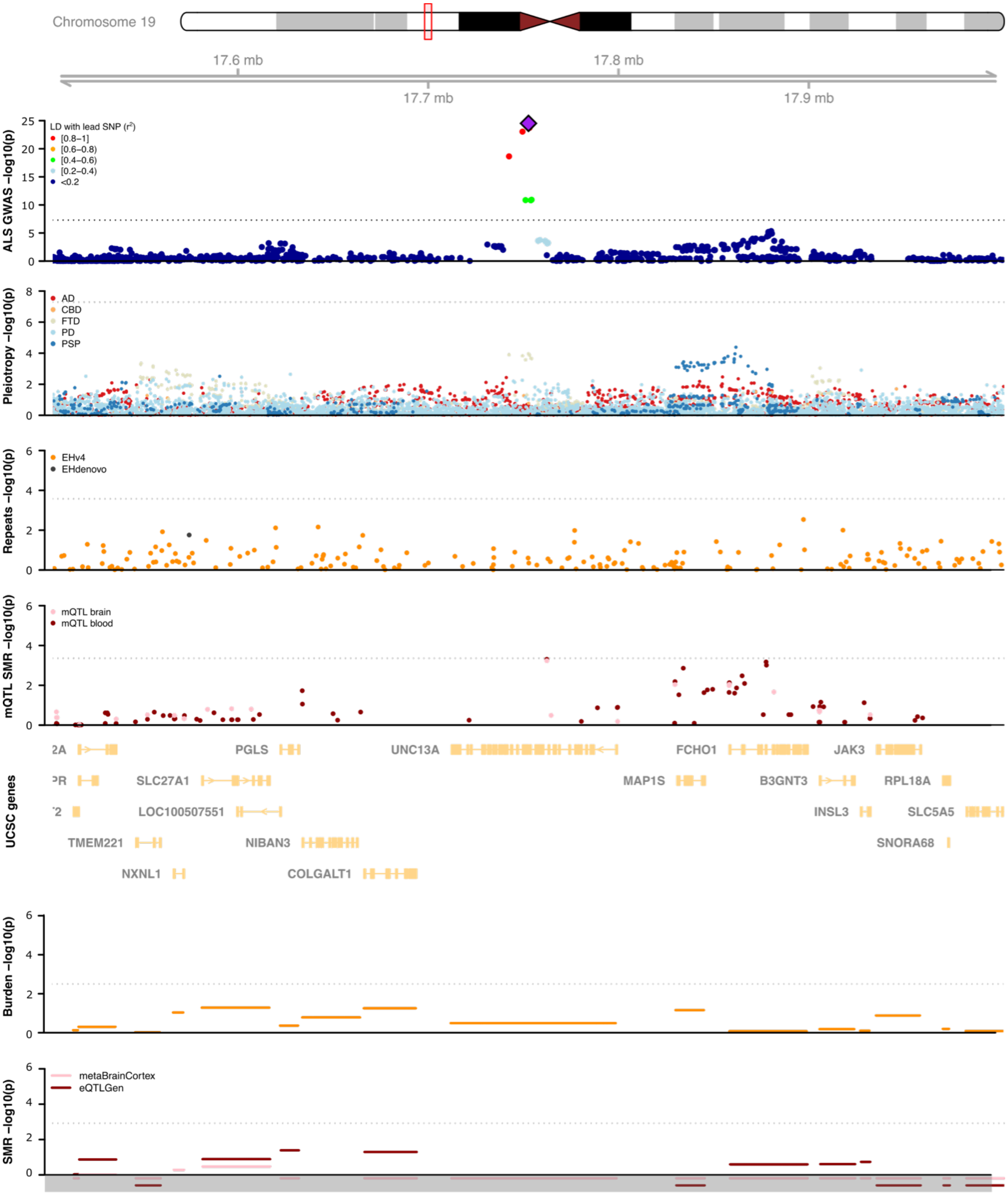

Chromosome 19
17.6 mb
17.8 mb
17.7 mb
17.9 mb
ALS GWAS −log10(p)
LD with lead SNP (r²)
[0.8–1]
[0.6–0.8)
[0.4–0.6)
[0.2–0.4)
<0.2
Pleiotropy −log10(p)
AD
CBD
FTD
PD
PSP
Repeats −log10(p)
EHv4
EHdenovo
mQTL SMR −log10(p)
mQTL brain
mQTL blood
UCSC genes
PGLS
UNC13A
FCHO1
JAK3
SLC27A1
MAP1S
B3GNT3
RPL18A
LOC100507551
INSL3
SLC5A5
TMEM221
NIBAN3
SNORA68
NXNL1
COLGALT1
Burden −log10(p)
SMR −log10(p)
metaBrainCortex
eQTLGen

Supplementary figure 13. Trackplot for rs17785991, *SLC9A8/SPATA2.* The rs17785991 locus consists of a long LD block spanning multiple genes. The strongest brain-mQTL identified CpG sites that were predicted to change expression of *SPATA2*, but SMR revealed multiple CpG sites spanning this region. eQTL SMR on the other hand highlighted *SLC9A8*, with a strong brain-specific signal with no evidence for heterogeneity (b_{xy} = 0.195 ± 0.042, P_{SMR} = 2.50 × 10^{-6}, P_{HEIDI} = 0.38). Number of tests: SMR eQTL = 21 gene tissue pairs, SMR mQTL = 96 CpG tissue pairs, rare variant burden = 8 genes, repeat expansion = 150 repeat loci.

[figure on next page]

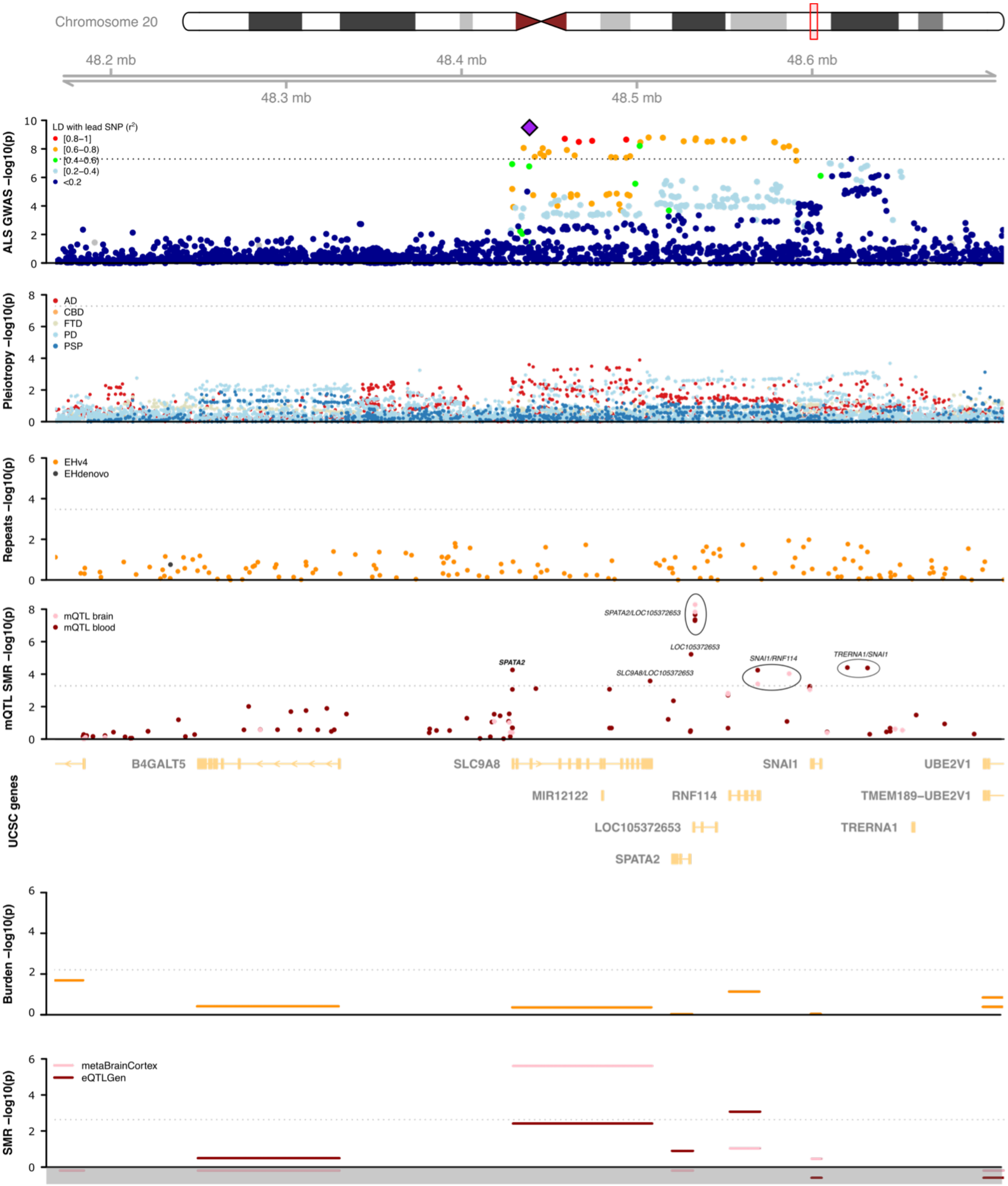

Chromosome 20
48.2 mb
48.3 mb
48.4 mb
48.5 mb
48.6 mb
ALS GWAS −log10(p)
LD with lead SNP (r²)
[0.8–1]
[0.6–0.8)
[0.4–0.6)
[0.2–0.4)
<0.2
Pleiotropy −log10(p)
AD
CBD
FTD
PD
PSP
Repeats −log10(p)
EHv4
EHdenovo
mQTL SMR −log10(p)
mQTL brain
mQTL blood
SPATA2/LOC105372653
LOC105372653
SPATA2
SLC9A8/LOC105372653
SNAI1/RNF114
TRERNA1/SNAI1
UCSC genes
B4GALT5
SLC9A8
SNAI1
UBE2V1
MIR12122
RNF114
TMEM189−UBE2V1
LOC105372653
TRERNA1
SPATA2
Burden −log10(p)
SMR −log10(p)
metaBrainCortex
eQTLGen

Supplementary figure 14. Trackplot for rs80265967, *SOD1.* The lead SNP rs80265967 is the coding (p.D90A) variant in *SOD1*. *SOD1* was the first gene described to cause ALS with a predominant autosomal dominant mode of inheritance. The p.D90A variant has been associated with ALS with an autosomal recessive mode of inheritance and is more common in the Finish population. The rare variant burden of *SOD1* was associated with ALS (P_{RVB} = 6.2 × 10^{-4} for all disruptive, damaging and non-synonymous variants with MAF < 0.01) which passed the threshold after correcting for multiple testing within this locus (0.05/16 = 3.1 × 10^{-3}). This association was not driven by the p.D90A variant due to the fact that no Finnish ALS cases were included in the WGS cohort. Conditioning on the p.D90A did not change the association statistics for rare variants within *SOD1* in the individuals overlapping the WGS and GWAS cohort (P_{RVB} = 4.5 × 10^{-4}, $P_{RVB|SNP}$ = 4.5 × 10^{-4}). A dinucleotide repeat expansion intronic of *TIAM1* was associated with ALS (threshold > 24 dinucleotides, P = 7.0 × 10^{-5}, FDR = 1.3 × 10^{-3}), which passed the threshold after correcting for multiple testing within this locus. This repeat is in LD with the p.D90A variant in *SOD1* (r^2 *= 2.7* × 10^{-4}, |D'| = 0.39). Number of tests: SMR eQTL = 33 gene tissue pairs, SMR mQTL = 124 CpG tissue pairs, rare variant burden = 16 genes, repeat expansion = 344 repeat loci.
[figure on next page]

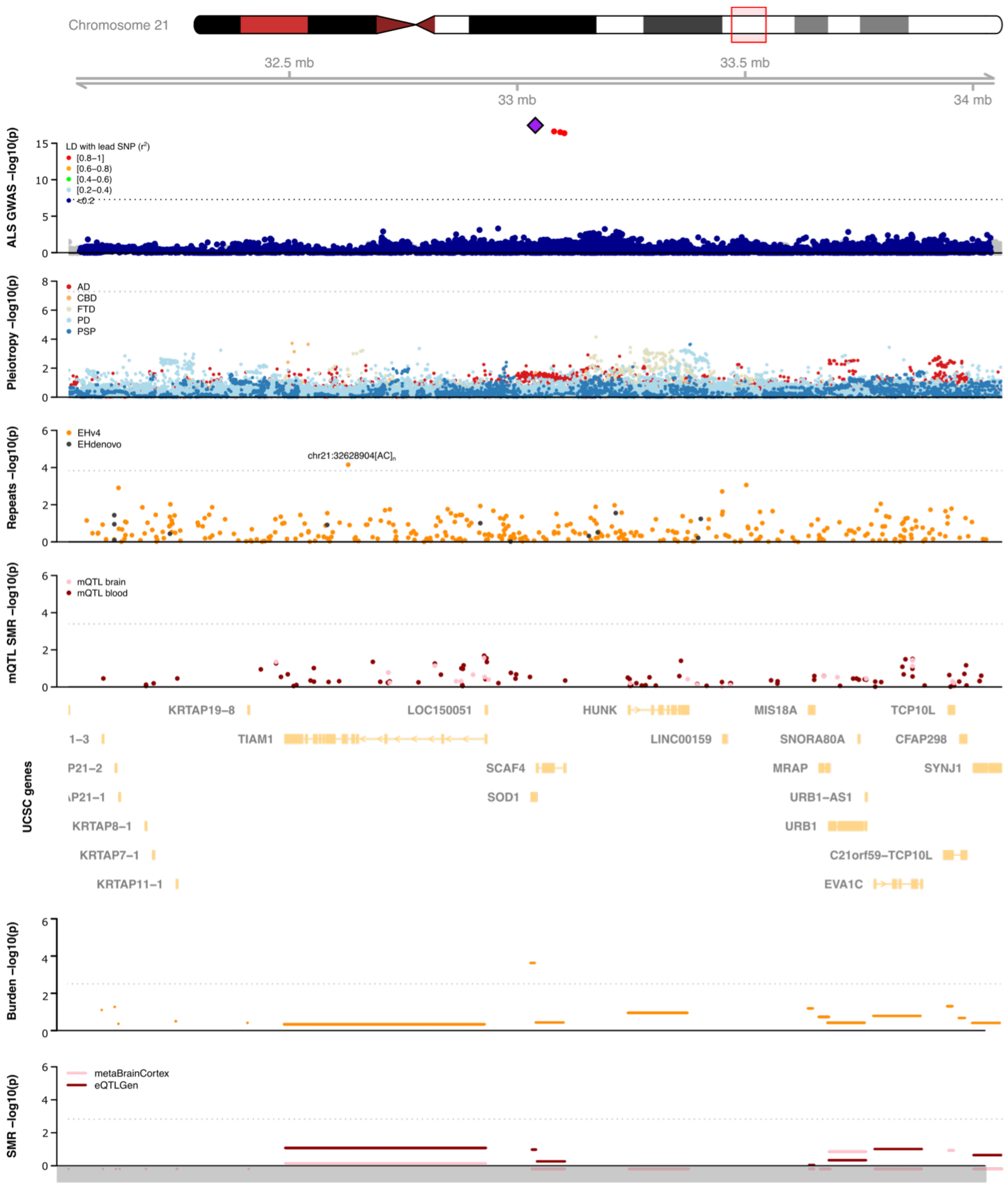
Chromosome 21
32.5 mb
33.5 mb
33 mb
34 mb
ALS GWAS −log10(p)
LD with lead SNP (r²)
[0.8−1]
[0.6−0.8)
[0.4−0.6)
[0.2−0.4)
≤0.2
Pleiotropy −log10(p)
AD
CBD
FTD
PD
PSP
Repeats −log10(p)
EHv4
EHdenovo
chr21:32628904[AC]n
mQTL SMR −log10(p)
mQTL brain
mQTL blood
UCSC genes
KRTAP19−8
LOC150051
HUNK
MIS18A
TCP10L
TIAM1
LINC00159
SNORA80A
CFAP298
SCAF4
MRAP
SYNJ1
SOD1
URB1−AS1
KRTAP8−1
URB1
KRTAP7−1
C21orf59−TCP10L
KRTAP11−1
EVA1C
Burden −log10(p)
SMR −log10(p)
metaBrainCortex
eQTLGen

Supplementary figure 15. Trackplot for rs75087725, *CFAP410*. The rs75087725 has previously been described in the discovery of the new ALS gene *C21orf2* (ref. [45]), later named *CFAP410*. This is a known coding variant in *CFAP410* (p.V58L). The rare variant burden association of *CFAP410* was nominally significant ($P_{RVB} = 5.7 \times 10^{-3}$ for all disruptive, damaging and non-synonymous variants with MAF < 0.01), which did not meet the threshold for multiple testing correction within this locus ($0.05/40 = 1.25 \times 10^{-3}$), but was conditionally independent from rs75087725. Number of tests: SMR eQTL = 51 gene tissue pairs, SMR mQTL = 294 CpG tissue pairs, rare variant burden = 29 genes, repeat expansion = 178 repeat loci. [figure on next page]

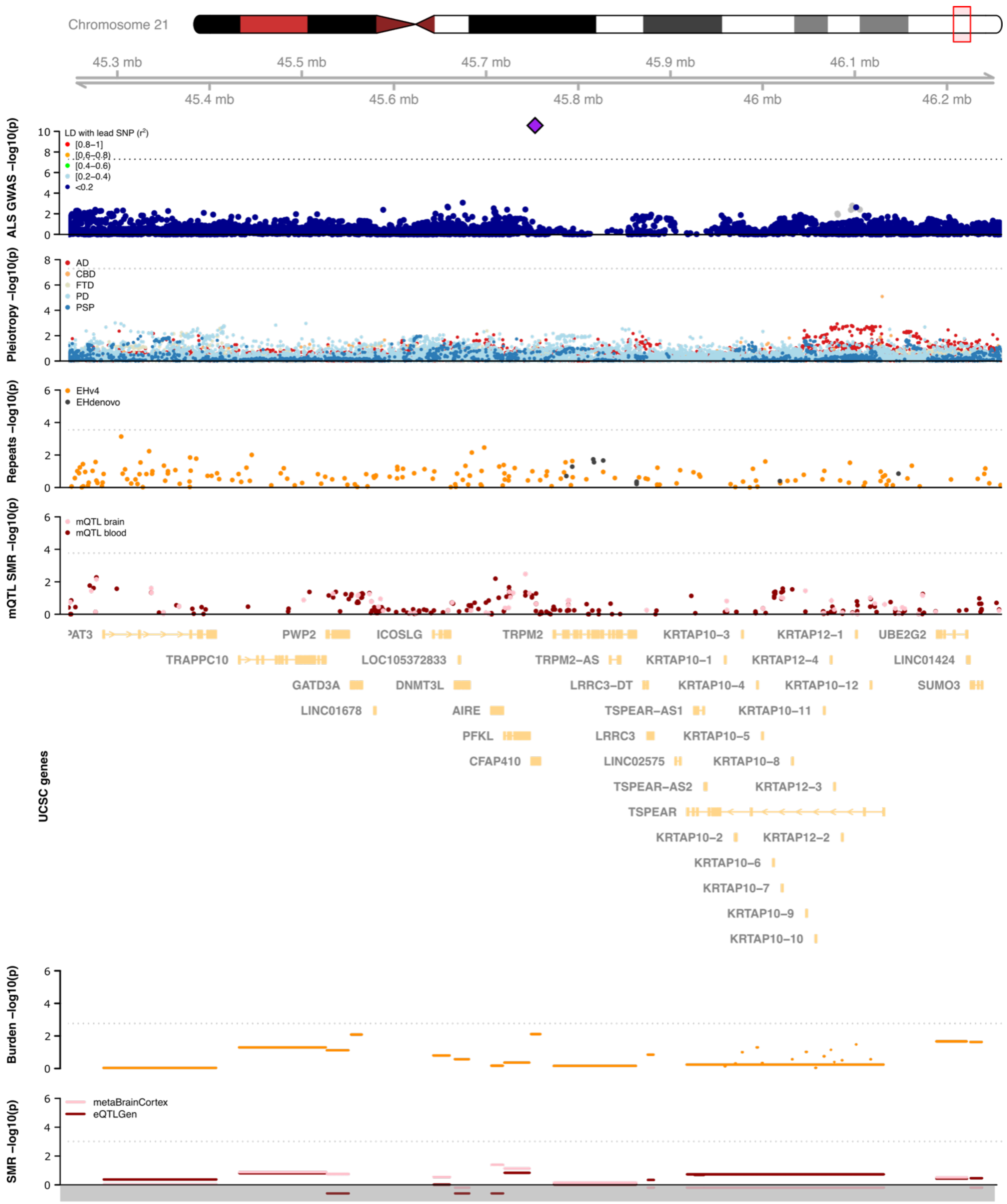

Chromosome 21
45.3 mb
45.5 mb
45.7 mb
45.9 mb
46.1 mb
45.4 mb
45.6 mb
45.8 mb
46 mb
46.2 mb
ALS GWAS −log10(p)
LD with lead SNP (r²)
[0.8–1]
[0.6–0.8)
[0.4–0.6)
[0.2–0.4)
<0.2
Pleiotropy −log10(p)
AD
CBD
FTD
PD
PSP
Repeats −log10(p)
EHv4
EHdenovo
mQTL SMR −log10(p)
mQTL brain
mQTL blood
UCSC genes
PAT3
TRAPPC10
PWP2
GATD3A
LINC01678
ICOSLG
LOC105372833
DNMT3L
AIRE
PFKL
CFAP410
TRPM2
TRPM2−AS
LRRC3−DT
TSPEAR−AS1
LRRC3
LINC02575
TSPEAR−AS2
TSPEAR
KRTAP10−3
KRTAP10−1
KRTAP10−4
KRTAP10−11
KRTAP10−5
KRTAP10−8
KRTAP12−3
KRTAP10−2
KRTAP10−6
KRTAP10−7
KRTAP10−9
KRTAP10−10
KRTAP12−1
KRTAP12−4
KRTAP10−12
KRTAP12−2
UBE2G2
LINC01424
SUMO3
Burden −log10(p)
SMR −log10(p)
metaBrainCortex
eQTLGen

Supplementary figure 16. Tissue enrichment analyses, full GTEx tissues. Enrichment analyses for all GTEx v8 tissues. Significant enrichment after FDR correction for multiple testing are marked with an asterisk. ALS = amyotrophic lateral sclerosis, PD = Parkinson's disease, AD = Alzheimer's disease.

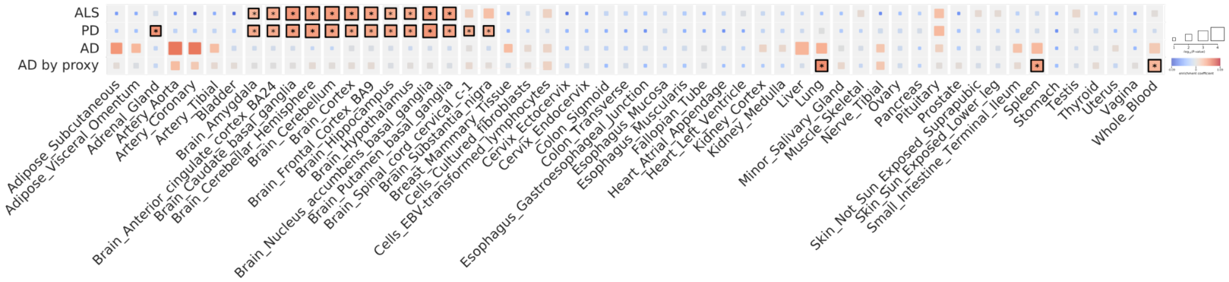

Supplementary figure 17. Principal components plots per stratum including HapMap3 reference populations. Individuals included in the GWAS, after removing outliers, plotted along their projected principal components based on HapMap3 reference populations for stratum 1 (**a**), 2 (**b**), 3 (**c**), 4 (**d**), 6 (**e**) and 7 (**f**). EUR = European ancestries reference population, MEX = Mexican ancestries reference populations, GIH = Gujarati Indians from Houston, AFR = African ancestries reference population and ASN = Asian ancestries reference population.

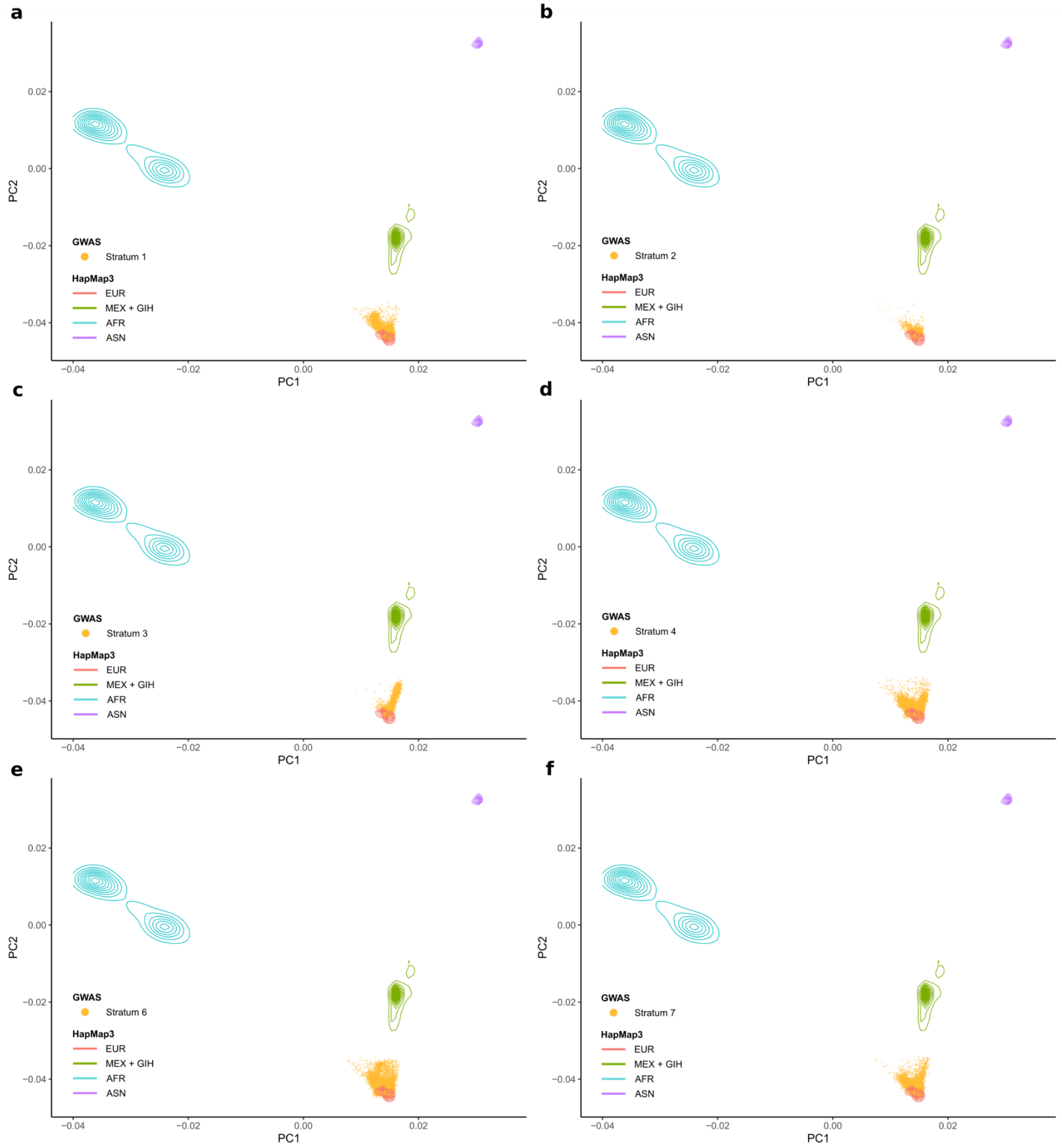

Supplementary figure 18. SMR, S-PrediXcan and TWAS comparison. Comparison between eQTL gene prioritization analyses using SMR, S-PrediXcan and TWAS with eQTL estimates from GTEx v7 whole blood and brain samples. eQTL-based gene statistics across all three methods were strongly correlated.

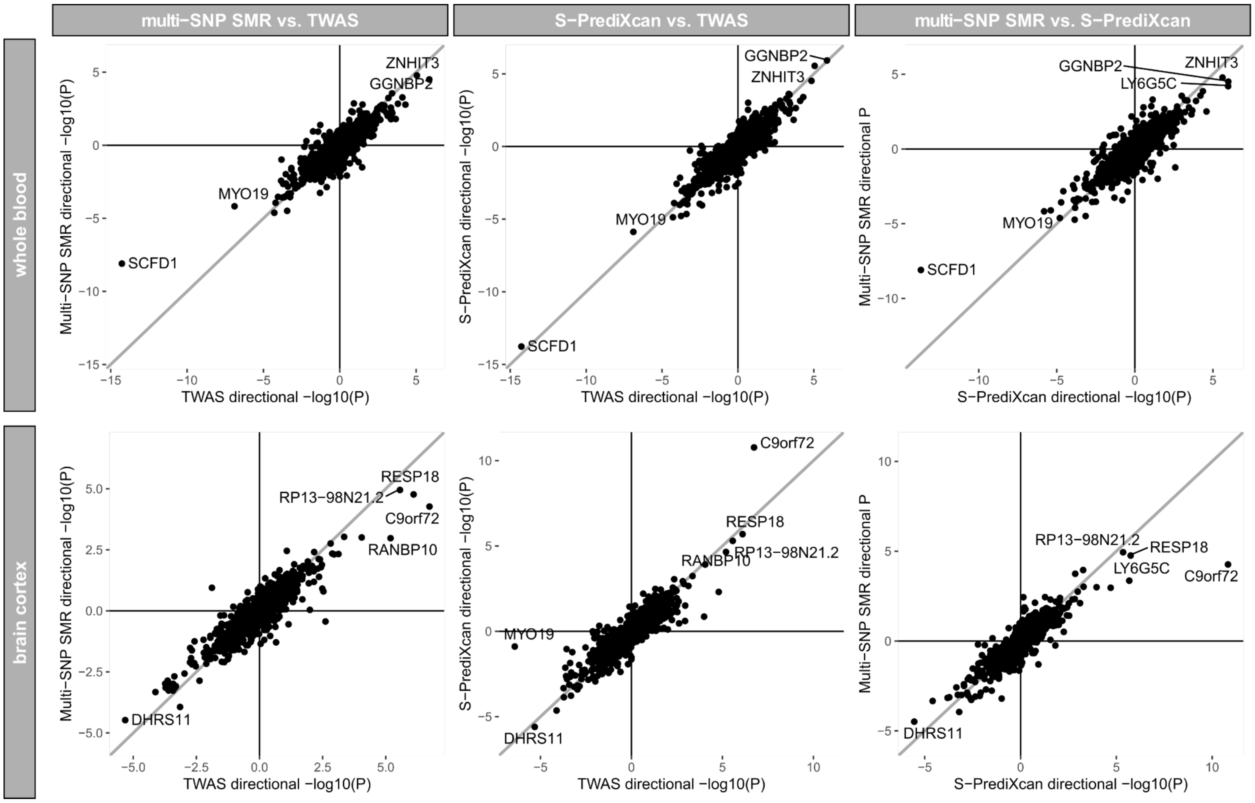

Supplementary Tables

Supplementary table 1. GWAS Cohort description
[table in Excel file]

Supplementary table 2. GWAS Quality control details
[table in Excel file]

Supplementary table 3. WGS Quality control details
[table in Excel file]

Supplementary tables 4-18. Details for genome-wide significant loci. SNP ID, effect allele, and annotated genes are reported above each Table. Per-stratum statistics are obtained from (two-sided) SAIGE logistic mixed model regression, meta-analysis results are obtained from inverse-variance weighted meta-analysis . European ancestries is the meta-analysis of strata s1, s2, s3, s4, s6, and s7. Cross-ancestry is the meta-analysis of European ancestries, China and Japan. Freq = effect allele frequency, N_{eff} = effective sample size.

Supplementary table 4. Details for rs631312 (G), *MOBP/RPSA*

Stratum	Freq	INFO	beta	SE	P	N_{eff}
s1	0.28	1.00	0.12	0.038	2.07×10^{-3}	7,500
s2	0.29	0.98	0.08	0.055	0.122	3,403
s3	0.30	0.98	0.12	0.052	0.024	4,085
s4	0.28	0.98	0.11	0.033	9.57×10^{-4}	12,570
s6	0.29	1.00	0.05	0.017	0.006	39,764
s7	0.30	0.95	0.12	0.031	6.29×10^{-5}	13,391
European ancestries	0.29	-	0.08	0.012	5.24×10^{-11}	80,713
China	-	-	0.04	0.049	0.405	3,445
Japan	-	-	0.13	0.053	0.012	4,147
Cross-ancestry	-	-	0.08	0.011	3.28×10^{-12}	88,305

Supplementary table 5. Details for rs62333164 (A), *NEK1*

Stratum	Freq	INFO	beta	SE	P	N_{eff}
s1	0.33	0.95	0.06	0.038	0.09	7,500
s2	0.34	0.99	0.01	0.054	0.82	3,403
s3	0.34	1.00	0.08	0.050	0.12	4,085
s4	0.33	1.00	0.03	0.032	0.27	12,570
s6	0.34	0.98	0.08	0.016	4.7×10^{-6}	39,764
s7	0.33	0.96	0.06	0.030	0.05	13,391
European ancestries	0.33	-	0.06	0.012	7.0×10^{-8}	80,713
China	-	-	0.19	0.094	0.04	3,445
Japan	-	-	0.22	0.106	0.04	4,147
Cross-ancestry	-	-	0.07	0.012	6.9×10^{-9}	88,305

Supplementary table 6. Details for rs10463311 (C), *GPX3/TNIP1*

Stratum	Freq	INFO	beta	SE	P	N_{eff}
s1	0.25	0.990	0.12	0.04	0.003	7,500
s2	0.24	0.987	0.10	0.06	0.083	3,403
s3	0.25	0.997	0.08	0.05	0.150	4,085
s4	0.25	0.996	0.11	0.03	6.30×10^{-4}	12,570
s6	0.26	0.993	0.07	0.02	8.30×10^{-5}	39,764
s7	0.25	0.950	0.05	0.03	0.156	13,391
European ancestries	0.25	-	0.08	0.01	3.46×10^{-10}	80,713
China	-	-	0.13	0.05	0.009	3,445
Japan	-	-	-0.06	0.05	0.284	4,147
Cross-ancestry	-	-	0.08	0.01	2.73×10^{-10}	88,305

Supplementary table 7. Details for rs517339 (C), *ERGIC1*

Stratum	Freq	INFO	beta	SE	P	N_{eff}
s1	0.40	1.000	-0.06	0.035	0.11	7,500
s2	0.40	0.998	-0.06	0.051	0.23	3,403
s3	0.38	0.999	-0.07	0.048	0.14	4,085
s4	0.40	1.000	-0.07	0.030	0.02	12,570
s6	0.40	0.998	-0.07	0.016	2.5×10^{-6}	39,764
s7	0.40	0.976	-0.03	0.029	0.29	13,391
European ancestries	0.40	-	-0.06	0.011	8.5×10^{-9}	80,713
China	-	-	-0.07	0.074	0.38	3,445
Japan	-	-	-	-	-	
Cross-ancestry	-	-	-0.06	0.011	5.6×10^{-9}	84,158

Supplementary table 8. Details for rs9275477 (C), *HLA*

Stratum	Freq	INFO	beta	SE	P	N_{eff}
s1	0.10	0.96	-0.230	0.060	1.1×10^{-4}	7,500
s2	0.09	0.95	-0.062	0.087	0.47	3,403
s3	0.11	0.97	0.005	0.076	0.95	4,085
s4	0.09	0.96	-0.091	0.057	0.11	12,570
s6	0.09	0.74	-0.195	0.031	2.6×10^{-10}	39,764
s7	0.09	0.99	-0.072	0.051	0.15	13,391
European ancestries	0.10	-	-0.143	0.021	5.5×10^{-12}	80,713
China	-	-	-0.110	0.111	0.34	3,445
Japan	-	-	-	-	-	-
Cross-ancestry	-	-	-0.142	0.020	3.5×10^{-12}	84,158

Supplementary table 9. Details for rs10280711 (G), *PTPRN2*

Stratum	Freq	INFO	beta	SE	P	N_{eff}
s1	0.13	0.80	0.082	0.058	0.156	7,500
s2	0.12	0.95	0.065	0.077	0.398	3,403
s3	0.13	0.99	0.048	0.072	0.507	4,085
s4	0.12	0.99	0.155	0.044	4.40×10^{-4}	12,570
s6	0.12	0.98	0.076	0.023	1.13×10^{-3}	39,764
s7	0.13	0.91	0.012	0.043	0.78	13,391
European ancestries	0.12	-	0.076	0.017	5.76×10^{-6}	80,713
China	-	-	0.139	0.048	0.005	3,445
Japan	-	-	0.122	0.056	0.029	4,147
Cross-ancestry	-	-	0.086	0.015	1.78×10^{-8}	88,305

Supplementary table 10. Details for rs2453555 (A), *C9orf72*

Stratum	Freq	INFO	beta	SE	P	N_{eff}
s1	0.24	0.93	0.16	0.04	1.84×10^{-4}	7,500
s2	0.24	0.99	0.17	0.06	0.004	3,403
s3	0.24	1.00	0.30	0.05	3.26×10^{-8}	4,085
s4	0.24	0.99	0.17	0.03	5.67×10^{-7}	12,570
s6	0.25	1.00	0.18	0.02	7.50×10^{-25}	39,764
s7	0.24	0.98	0.12	0.03	3.42×10^{-4}	13,391
European ancestries	0.25	-	0.17	0.01	9.98×10^{-43}	80,713
China	-	-	-0.07	0.10	0.514	3,445
Japan	-	-	0.08	0.09	0.350	4,147
Cross-ancestry	-	-	0.17	0.01	1.48×10^{-41}	88,305

Supplementary table 11. Details for rs113247976 (T), *KIF5A*

Stratum	Freq	INFO	beta	SE	P	N_{eff}
s1	0.014	0.82	0.26	0.16	0.105	7,500
s2	0.016	0.89	0.47	0.21	0.023	3,403
s3	0.025	0.93	0.27	0.15	0.081	4,085
s4	0.014	0.88	0.13	0.15	0.385	12,570
s6	0.015	0.83	0.38	0.07	3.48×10^{-8}	39,764
s7	0.012	0.85	0.36	0.14	0.008	13,391
European ancestries	0.016	-	0.33	0.05	1.42×10^{-11}	80,713
China	-	-	-	-	-	-
Japan	-	-	-	-	-	-
Cross-ancestry	-	-	0.33	0.05	1.42×10^{-11}	80,713

Supplementary table 12. Details for rs4075094 (A), *TBK1*

Stratum	Freq	INFO	beta	SE	P	N_{eff}
s1	0.11	0.97	-0.08	0.06	0.14	7,500
s2	0.11	0.98	-0.03	0.08	0.75	3,403
s3	0.11	0.99	-0.16	0.07	0.03	4,085
s4	0.11	0.98	-0.07	0.04	0.14	12,570
s6	0.11	0.94	-0.08	0.02	6.9×10^{-4}	39,764
s7	0.10	0.97	-0.19	0.04	2.7×10^{-5}	13,391
European ancestries	0.11	-	-0.10	0.02	1.7×10^{-8}	80,713
China	-	-	-0.28	0.11	0.02	3,445
Japan	-	-	-0.10	0.15	0.52	4,147
Cross-ancestry	-	-	-0.10	0.02	2.1×10^{-9}	88,305

Supplementary table 13. Details for rs2985994 (C), *COG3*

Stratum	Freq	INFO	beta	SE	P	N_{eff}
s1	0.26	0.97	0.11	0.040	0.004	7,500
s2	0.26	0.98	0.07	0.057	0.225	3,403
s3	0.27	0.99	0.05	0.053	0.344	4,085
s4	0.26	0.99	0.06	0.035	0.078	12,570
s6	0.26	0.98	0.06	0.018	0.0012	39,764
s7	0.26	0.95	0.07	0.033	0.029	13,391
European ancestries	0.26	-	0.07	0.013	1.87×10^{-7}	80,713
China	-	-	0.07	0.054	0.188	3,445
Japan	-	-	0.14	0.062	0.026	4,147
Cross-ancestry	-	-	0.07	0.012	1.16×10^{-8}	88,305

Supplementary table 14. Details for rs229195 (A), *SCFD1*

Stratum	Freq	INFO	beta	SE	P	N_{eff}
s1	0.33	0.981	0.14	0.037	1.70×10^{-4}	7,500
s2	0.34	0.997	0.02	0.053	0.673	3,403
s3	0.34	0.999	0.10	0.049	0.037	4,085
s4	0.34	0.999	0.10	0.031	0.0010	12,570
s6	0.34	0.950	0.11	0.017	2.70×10^{-10}	39,764
s7	0.33	0.939	0.02	0.031	0.522	13,391
European ancestries	0.34	-	0.09	0.012	9.20×10^{-15}	80,713
China	-	-	-	-	-	-
Japan	-	-	-	-	-	-
Cross-ancestry	-	-	0.09	0.012	9.20×10^{-15}	80,713

Supplementary table 15. Details for rs12608932 (C), *UNC13A*

Stratum	Freq	INFO	beta	SE	P	N_{eff}
s1	-	0.514	-	-	-	-
s2	0.35	0.997	0.12	0.05	0.02	3,403
s3	0.39	0.998	0.25	0.05	1.6×10^{-7}	4,085
s4	0.34	0.997	0.07	0.03	0.03	12,570
s6	0.34	0.997	0.12	0.02	1.3×10^{-13}	39,764
s7	0.35	0.988	0.14	0.03	9.1×10^{-7}	13,391
European ancestries	0.35	-	0.12	0.01	8.8×10^{-25}	73,213
China	-	-	0.05	0.05	0.29	3,445
Japan	-	-	0.11	0.06	0.08	4,147
Cross-ancestry	-	-	0.12	0.01	3.0×10^{-25}	80,805

Supplementary table 16. Details for rs17785991 (A), *SLC9A8/SPATA2*

Stratum	Freq	INFO	beta	SE	P	N_{eff}
s1	0.34	0.97	0.09	0.037	0.01	7,500
s2	0.36	0.90	0.09	0.055	0.10	3,403
s3	0.31	0.93	0.12	0.053	0.02	4,085
s4	0.35	0.95	0.05	0.032	0.11	12,570
s6	0.36	0.93	0.06	0.017	1.1×10^{-4}	39,764
s7	0.34	0.97	0.10	0.030	1.2×10^{-3}	13,391
European ancestries	0.35	-	0.07	0.012	3.5×10^{-10}	80,713
China	-	-	-	-	-	-
Japan	-	-	0.04	0.076	0.55	4,147
Cross-ancestry	-	-	0.07	0.012	3.2×10^{-10}	84,860

Supplementary table 17. Details for rs80265967 (C), *SOD1*

Stratum	Freq	INFO	beta	SE	P	N_{eff}
s1	-	0.61	-	-	-	-
s2	0.0006	0.79	0.08	1.21	0.95	3,403
s3	0.0105	0.96	0.91	0.17	1.0×10^{-7}	4,085
s4	0.0007	0.82	1.07	0.48	0.03	12,570
s6	0.0009	0.80	1.27	0.23	2.3×10^{-8}	39,764
s7	0.0008	0.98	1.56	0.40	1.2×10^{-4}	13,391
European ancestries	0.0060	-	1.08	0.12	3.5×10^{-18}	73,213
China	-	-	-	-	-	-
Japan	-	-	-	-	-	-
Cross-ancestry	-	-	1.08	0.12	3.5×10^{-18}	73,123

Supplementary table 18. Details for rs75087725 (A), *CFAP410*

Stratum	Freq	INFO	beta	SE	P	N_{eff}
s1	-	-	-	-	-	-
s2	-	-	-	-	-	-
s3	0.015	0.88	0.37	0.20	0.07	4,085
s4	0.012	0.83	0.34	0.16	0.03	12,570
s6	0.012	0.72	0.41	0.08	8.9×10^{-7}	39,764
s7	0.011	0.79	0.55	0.15	2.8×10^{-4}	13,391
European ancestries	0.012	-	0.42	0.06	2.7×10^{-11}	69,810
China	-	-	-	-	-	-
Japan	-	-	-	-	-	-
Cross-ancestry	-	-	0.42	0.06	2.7×10^{-11}	69,810

Supplementary table 19. Details for rs58854276 (A), *ACSL5/ZDHHC6*

Stratum	Freq	INFO	beta	SE	P	N_{eff}
s1	0.66	1.0	0.09	0.04	0.01	7,500
s2	0.66	0.94	0.14	0.05	0.01	3,403
s3	0.65	0.97	0.00	0.05	0.97	4,085
s4	0.65	1.0	0.10	0.03	1.7×10^{-3}	12,570
s6	0.66	0.99	0.02	0.02	0.24	39,764
s7	0.66	0.95	0.06	0.03	0.05	13,391
European ancestries	0.66	-	0.05	0.01	5.4×10^{-4}	80,713
China	-	-	0.19	0.05	1.8×10^{-4}	3,445
Japan	-	-	0.17	0.05	1.6×10^{-3}	4,147
Cross-ancestry	-	-	0.06	0.01	6.5×10^{-8}	88,305

Supplementary table 20. Rare variant genic burden analyses. In total, 14 different models with different minor allele frequency (MAF) thresholds and variant annotations were tests. The rare variant genic burden association signal for *NEK1* was the only exome-wide significant gene across all these models. No systematic inflation of the test statistics was observed ($\lambda_{GC} \leq 1.00$).

Model	MAF	Annotation	λ_{GC}	Exome-wide genes	P
1	< 0.01	disruptive	0.944	*NEK1*	2.63×10^{-7}
2	< 0.005	disruptive	0.940	*NEK1*	2.46×10^{-7}
3	< 0.01	damaging	0.942	-	-
4	< 0.005	damaging	0.934	-	-
5	< 0.01	missense	0.998	-	-
6	< 0.005	missense	0.992	-	-
7	< 0.01	synonymous	0.977	-	-
8	< 0.005	synonymous	0.989	-	-
9	< 0.01	non-classified	0.976	-	-
10	< 0.005	non-classified	0.976	-	-
11	< 0.01	disruptive + damaging	0.959	*NEK1*	8.63×10^{-8}
12	< 0.005	disruptive + damaging	0.945	*NEK1*	4.88×10^{-8}
13	< 0.01	all non-synonymous	0.993	-	-
14	< 0.005	all non-synonymous	1.00	-	-

Supplementary table 21. Association signals in neurodegenerative diseases. Association beta (b) and P-values (P) of ALS lead SNPs for Cross-ancestry ALS, Alzheimer's disease (AD), Parkinson's disease (PD), progressive supranuclear palsy (PSP), frontotemporal dementia (FTD), and corticobasal degeneration (CBD) are shown. EA: effect allele. Name: annotated gene names.

SNP	EA	Name	b_{ALS}	P_{ALS}	b_{AD}	P_{AD}	b_{PD}	P_{PD}	b_{PSP}	P_{PSP}	b_{FTD}	P_{FTD}	b_{CBD}	P_{CBD}
rs2453555	A	*C9orf72*	0.17	1.5E-41	-0.004	0.82	0.009	0.67	0.003	0.56	0.149	6.5E-04	*NA*	*NA*
rs12608932	C	*UNC13A*	0.12	3.0E-25	0	1.00	0.010	0.60	*NA*	*NA*	0.159	0.00011	-0.18	0.17
rs80265967	C	*SOD1*	1.08	3.5E-18	*NA*	*NA*	-1.014	0.13	*NA*	*NA*	*NA*	*NA*	*NA*	*NA*
rs229195	A	*SCFD1*	0.09	9.2E-15	0.002	0.91	0.003	0.89	0.003	0.47	-0.007	0.88	*NA*	*NA*
rs631312	G	*MOBP/RPSA*	0.08	3.3E-12	0.019	0.24	0.032	0.09	0.039	3.0E-16	0.129	0.02	*NA*	*NA*
rs9275477	C	*HLA*	-0.14	3.5E-12	-0.120	3.3E-06	-0.152	1.2E-05	-0.015	0.03	-0.099	0.21	*NA*	*NA*
rs113247976	T	*KIF5A*	0.33	1.4E-11	0.002	0.98	0.121	0.13	0.007	0.69	*NA*	*NA*	*NA*	*NA*
rs75087725	A	*CFAP410*	0.42	2.7E-11	*NA*	*NA*	-0.059	0.48	*NA*	*NA*	*NA*	*NA*	*NA*	*NA*
rs10463311	C	*GPX3/TNIP1*	0.08	2.7E-10	0.013	0.43	0.004	0.85	-0.010	0.05	-0.033	0.47	*NA*	*NA*
rs17785991	A	*SLC9A8/SPATA2*	0.07	3.2E-10	0.047	0.002	0.016	0.44	0.006	0.19	0.062	0.18	*NA*	*NA*
rs4075094	A	*TBK1*	-0.10	2.1E-09	-0.019	0.40	0.051	0.16	0.010	0.12	-0.004	0.95	*NA*	*NA*
rs517339	C	*ERGIC1/CREBRF*	-0.06	5.6E-09	0.007	0.62	0.002	0.92	-0.0004	0.93	-0.069	0.09	*NA*	*NA*
rs62333164	A	*NEK1*	0.07	6.9E-09	-0.025	0.11	0.058	0.0014	-0.004	0.38	-0.074	0.08	*NA*	*NA*
rs2985994	C	*COG3*	0.07	1.2E-08	-0.002	0.89	-0.002	0.91	-0.008	0.12	-0.003	0.94	*NA*	*NA*
rs10280711	G	*PTPRN2*	0.09	1.8E-08	0.003	0.89	-0.031	0.38	0.001	0.90	0.167	0.005	*NA*	*NA*
rs34311866	C	*GAK*	0.07	1.1E-06	0.015	0.46	0.227	8.0E-23	*NA*	*NA*	0.093	0.13	*NA*	*NA*
rs2632516	C	*BZRAP1-AS1*	-0.04	0.00010	-0.075	3.7E-07	0.001	0.95	-0.001	0.74	-0.011	0.79	*NA*	*NA*

Supplementary tables 22-25. Downstreamer enrichment analyses.

Supplementary table 22. Downstreamer HPO term enrichment.

HPO term	Description	Enrichment Z-score	Enrichment P-value
HP:0002120	Cerebral cortical atrophy	5.63	1.8×10^{-8}
HP:0007367	Atrophy/Degeneration affecting the central nervous system	5.47	4.6×10^{-8}
HP:0001288	Gait disturbance	5.38	7.6×10^{-8}
HP:0001311	Abnormal nervous system electrophysiology	5.06	4.1×10^{-7}
HP:0007369	Atrophy/Degeneration affecting the cerebrum	5.06	4.2×10^{-7}
HP:0031466	Impairment in personality functioning	5.00	5.7×10^{-7}
HP:0012444	Brain atrophy	4.96	6.9×10^{-7}
HP:0002059	Cerebral atrophy	4.96	7.1×10^{-7}
HP:0002465	Poor speech	4.94	$8.0\times10-^{7}$
HP:0003679	Pace of progression	4.93	8.3×10^{-7}
HP:0003693	Distal amyotrophy	4.92	8.6×10^{-7}
HP:0000750	Delayed speech and language development	4.80	1.6×10^{-6}
HP:0030178	Abnormality of central nervous system electrophysiology	4.79	1.7×10^{-6}
HP:0000713	Agitation	4.79	1.7×10^{-6}
HP:0100852	Abnormal fear/anxiety-related behavior	4.78	1.7×10^{-6}
HP:0012757	Abnormal neuron morphology	4.76	2.0×10^{-6}
HP:0002071	Abnormality of extrapyramidal motor function	4.74	2.1×10^{-6}
HP:0100022	Abnormality of movement	4.73	2.3×10^{-6}
HP:0003674	Onset	4.73	2.3×10^{-6}
HP:0002067	Bradykinesia	4.72	2.4×10^{-6}
HP:0002353	EEG abnormality	4.72	2.4×10^{-6}
HP:0025270	Abnormality of esophagus physiology	4.7	2.6×10^{-6}
HP:0000739	Anxiety	4.7	2.6×10^{-6}
HP:0002021	Pyloric stenosis	4.68	2.9×10^{-6}
HP:0003676	Progressive	4.68	2.9×10^{-6}
HP:0002015	Dysphagia	4.67	3.0×10^{-6}
HP:0000711	Restlessness	4.63	3.7×10^{-6}
HP:0000764	Peripheral axonal degeneration	4.62	3.9×10^{-6}
HP:0003593	Infantile onset	4.61	4.0×10^{-6}
HP:0031826	Abnormal reflex	4.58	4.6×10^{-6}
HP:0100704	Cortical visual impairment	4.57	4.8×10^{-6}
HP:0002450	Abnormal motor neuron morphology	4.55	5.3×10^{-6}

HP:0031797	Clinical course	4.52	6.1×10^{-6}
HP:0011799	Abnormality of facial soft tissue	4.52	6.2×10^{-6}
HP:0002167	Neurological speech impairment	4.49	7.1×10^{-6}
HP:0000716	Depressivity	4.48	7.6×10^{-6}
HP:0002311	Incoordination	4.47	8.0×10^{-6}
HP:0001344	Absent speech	4.46	8.3×10^{-6}
HP:0000194	Open mouth	4.45	8.7×10^{-6}
HP:0001257	Spasticity	4.44	9.2×10^{-6}
HP:0001276	Hypertonia	4.43	9.4×10^{-6}
HP:0004305	Involuntary movements	4.43	9.5×10^{-6}
HP:0002540	Inability to walk	4.42	9.7×10^{-6}
HP:0008207	Primary adrenal insufficiency	4.42	9.8×10^{-6}
HP:0000301	Abnormality of facial musculature	4.41	1.0×10^{-5}
HP:0007373	Motor neuron atrophy	4.40	1.1×10^{-5}
HP:0000738	Hallucinations	4.39	1.2×10^{-5}
HP:0010993	Abnormality of the cerebral subcortex	4.37	1.2×10^{-5}
HP:0031828	Abnormal superficial reflex	4.37	1.2×10^{-5}
HP:0003487	Babinski sign	4.37	1.2×10^{-5}
HP:0004400	Abnormality of the pylorus	4.34	1.4×10^{-5}
HP:0001336	Myoclonus	4.33	1.5×10^{-5}
HP:0002072	Chorea	4.32	1.6×10^{-5}
HP:0000708	Behavioral abnormality	4.31	1.6×10^{-5}
HP:0001761	Pes cavus	4.30	1.7×10^{-5}
HP:0001272	Cerebellar atrophy	4.30	1.7×10^{-5}
HP:0003477	Peripheral axonal neuropathy	4.28	1.9×10^{-0}
HP:0002577	Abnormality of the stomach	4.27	2.0×10^{-5}

Supplementary table 23. Downstreamer Reactome enrichment.

Annotation	Parent branch	Enrichment Z-score	Enrichment P-value	Enrichment Q-value
Membrane Trafficking	Vesicle mediated transport	4.60	4.2×10^{-6}	0.0020
Intra-Golgi and retrograde Golgi-to-ER traffic	Vesicle mediated transport	4.34	1.4×10^{-5}	0.0022
Macroautophagy	Autophagy	4.16	3.2×10^{-5}	0.0022

Supplementary table 24. Downstreamer GO Biological Processes enrichment.

Annotation	Enrichment Z-score	Enrichment P-value	Enrichment Q-value
exocytosis	5.12	3.1×10^{-7}	0.0000
cytoplasmic microtubule organization	4.96	7.0×10^{-7}	0.0000
regulation of phosphoprotein phosphatase activity	4.66	3.2×10^{-6}	0.0000
protein targeting to vacuole	4.59	4.5×10^{-6}	0.0000
endosomal transport	4.54	5.5×10^{-6}	0.0000
protein K48-linked deubiquitination	4.50	6.8×10^{-6}	0.0000
microtubule nucleation	4.42	1.0×10^{-5}	7.7×10^{-4}
ubiquitin-dependent protein catabolic process	4.38	1.2×10^{-5}	7.7×10^{-4}
intracellular protein transport	4.33	1.5×10^{-5}	7.7×10^{-4}
retrograde transport, endosome to Golgi	4.31	1.6×10^{-5}	7.7×10^{-4}
clathrin-dependent endocytosis	4.25	2.1×10^{-5}	7.7×10^{-4}
vesicle-mediated transport	4.19	2.8×10^{-5}	7.7×10^{-4}
protein localization to phagophore assembly site	4.13	3.6×10^{-5}	9.7×10^{-4}
macroautophagy	4.12	3.8×10^{-5}	9.7×10^{-4}

Supplementary table 25. Downstreamer GO Cellular Components enrichment.

Annotation	Enrichment Z-score	Enrichment P-value	Enrichment Q-value
microtubule associated complex	4.14	3.5×10^{-5}	0.003
phagophore assembly site	3.99	6.6×10^{-5}	0.006

Supplementary table 26-34. Mendelian randomization details.

Supplementary table 26. Included GWAS for instrument selection.
[Table in Excel file]

Supplementary table 27. Full MR analyses. Life-style traits.
MR statistics for 5 different methods (weighted median, weighted mode, simple mode, MR Egger and inverse-variance weighted MR) and two-sided p-values are presented. Presented p-values are not adjusted for multiple testing.

		Instrument cut-off: $P < 5×10^{-8}$				Instrument cut-off: $P < 5×10^{-5}$			
Exposure	Method	SNPs	beta	se	P	SNPs	beta	se	P
Age Of Smoking Initiation	Weighted median	6	0.15	0.35	0.67	103	-0.2	0.13	0.12
	Weighted mode	6	0.25	0.53	0.66	103	-0.37	0.34	0.27
	Simple mode	6	0.27	0.58	0.66	103	-0.4	0.38	0.29
	MR Egger	6	0.92	1.81	0.64	103	-0.41	0.38	0.29
	IVW	6	0.11	0.38	0.78	103	-0.07	0.1	0.50
Cigarettes per Day	Weighted median	22	-0.07	0.06	0.28	140	-0.07	0.06	0.21
	Weighted mode	22	-0.06	0.06	0.28	140	-0.05	0.06	0.41
	Simple mode	22	-0.02	0.11	0.83	140	0.02	0.12	0.84
	MR Egger	22	-0.06	0.09	0.52	140	-0.06	0.07	0.36
	IVW	22	-0.05	0.05	0.33	140	-0.03	0.03	0.44
Alcoholic drinks per week	Weighted median	35	-0.18	0.15	0.24	252	-0.15	0.15	0.3
	Weighted mode	35	-0.23	0.15	0.14	252	-0.21	0.13	0.12
	Simple mode	35	-0.45	0.36	0.22	252	-0.21	0.38	0.59
	MR Egger	35	-0.2	0.24	0.40	252	-0.12	0.17	0.47
	IVW	35	0	0.15	1	252	0.04	0.08	0.61
Body Mass Index	Weighted median	506	-0.06	0.07	0.35	526	-0.01	0.07	0.88
	Weighted mode	506	-0.04	0.12	0.74	526	0.01	0.15	0.96
	Simple mode	506	0.35	0.19	0.07	526	0.07	0.22	0.76
	MR Egger	506	-0.12	0.11	0.26	526	0.01	0.12	0.91
	IVW	506	-0.03	0.04	0.53	526	-0.02	0.05	0.61
Moderate activity	Weighted median	8	-0.11	0.17	0.50	96	0.03	0.07	0.71
	Weighted mode	8	-0.17	0.23	0.48	96	-0.18	0.18	0.32
	Simple mode	8	-0.17	0.23	0.50	96	-0.18	0.2	0.37
	MR Egger	8	0.08	0.89	0.93	96	0.16	0.23	0.48
	IVW	8	0.02	0.12	0.89	96	0.03	0.06	0.54
Vigorous activity	Weighted median	8	0.31	0.21	0.14	79	0.16	0.09	0.09
	Weighted mode	8	0.53	0.34	0.16	79	0.24	0.22	0.28
	Simple mode	8	0.59	0.34	0.12	79	0.24	0.25	0.33
	MR Egger	8	-1.64	2.4	0.52	79	-0.27	0.25	0.29
	IVW	8	0.24	0.18	0.19	79	0.07	0.07	0.31

Supplementary table 27. Full MR analyses (cont.) Cardiovascular traits and Educational attainment.

		Instrument cut-off: $P < 5{\times}10^{-8}$				Instrument cut-off: $P < 5{\times}10^{-5}$			
Exposure	Method	SNPs	beta	se	P	SNPs	beta	se	P
Diastolic blood pressure	Weighted median	448	0	0.01	0.48	463	0	0.01	0.86
	Weighted mode	448	0.01	0.01	0.53	463	0	0.01	0.99
	Simple mode	448	0	0.02	0.82	463	0	0.02	0.82
	MR Egger	448	0.01	0.01	0.22	463	0.01	0.01	0.36
	IVW	448	0	0	0.58	463	0	0	0.49
Systolic blood pressure	Weighted median	450	0.01	0.003	0.06	514	0.01	0.004	0.06
	Weighted mode	450	0.01	0.01	0.3	514	0.01	0.01	0.32
	Simple mode	450	0.01	0.01	0.54	514	0.01	0.01	0.6
	MR Egger	450	0.01	0.01	0.09	514	0.01	0.01	0.07
	IVW	450	0.01	0.002	0.06	514	0.01	0.002	0.003
Coronary artery disease	Weighted median	144	0.03	0.03	0.44	388	0.02	0.03	0.45
	Weighted mode	144	0.04	0.04	0.35	388	0.03	0.04	0.43
	Simple mode	144	-0.03	0.08	0.74	388	-0.03	0.08	0.69
	MR Egger	144	0.08	0.05	0.09	388	0.04	0.04	0.34
	IVW	144	0.03	0.02	0.15	388	0.03	0.02	0.11
Stroke	Weighted median	8	0.19	0.1	0.06	42	0.07	0.06	0.23
	Weighted mode	8	0.22	0.12	0.1	42	-0.03	0.13	0.81
	Simple mode	8	0.22	0.14	0.16	42	-0.03	0.14	0.85
	MR Egger	8	0.84	0.63	0.23	42	0.01	0.13	0.97
	IVW	8	0.08	0.1	0.44	42	0.07	0.04	0.11
Years of schooling	Weighted median	306	-0.18	0.09	0.04	681	-0.16	0.07	0.02
	Weighted mode	306	-0.15	0.26	0.56	681	-0.2	0.24	0.41
	Simple mode	306	-0.19	0.28	0.51	681	-0.22	0.28	0.43
	MR Egger	306	0.05	0.27	0.85	681	0.03	0.17	0.85
	IVW	306	-0.18	0.07	0.01	681	-0.19	0.05	0.0002

Supplementary table 27. Full MR analyses (cont.) Lipids.

		Instrument cut-off: $P < 5\times10^{-8}$				Instrument cut-off: $P < 5\times10^{-5}$			
Exposure	Method	SNPs	beta	se	P	SNPs	beta	se	P
Triglycerides	Weighted median	54	0.01	0.06	0.8	135	0.01	0.06	0.81
	Weighted mode	54	0.05	0.05	0.4	135	0.06	0.05	0.27
	Simple mode	54	0.18	0.11	0.1	135	0.13	0.1	0.2
	MR Egger	54	0.1	0.07	0.19	135	0.07	0.07	0.35
	IVW	54	0.03	0.04	0.43	135	0.03	0.04	0.53
Total cholesterol	Weighted median	83	0.15	0.04	0.0003	178	0.15	0.04	0.0007
	Weighted mode	83	0.13	0.04	0.004	178	0.11	0.04	0.007
	Simple mode	83	0.02	0.1	0.86	178	0.03	0.11	0.75
	MR Egger	83	0.13	0.05	0.01	178	0.15	0.05	0.005
	IVW	83	0.09	0.03	0.005	178	0.08	0.03	0.02
HDL cholesterol	Weighted median	87	0.01	0.05	0.86	158	0	0.05	0.93
	Weighted mode	87	0.01	0.05	0.9	158	-0.02	0.05	0.73
	Simple mode	87	0.01	0.09	0.94	158	0.03	0.1	0.79
	MR Egger	87	-0.06	0.07	0.34	158	-0.08	0.07	0.27
	IVW	87	0.04	0.04	0.32	158	0.03	0.04	0.49
LDL cholesterol	Weighted median	75	0.08	0.04	0.06	147	0.08	0.05	0.07
	Weighted mode	75	0.06	0.04	0.16	147	0.07	0.04	0.04
	Simple mode	75	0.2	0.09	0.03	147	0.18	0.09	0.05
	MR Egger	75	0.11	0.05	0.03	147	0.1	0.05	0.03
	IVW	75	0.07	0.03	0.03	147	0.06	0.03	0.08

Supplementary table 27. Full MR analyses (cont.) Inflammation markers.

		Instrument cut-off: $P < 5\times10^{-8}$				Instrument cut-off: $P < 5\times10^{-5}$			
Exposure	Method	SNPs	beta	se	P	SNPs	beta	se	P
C-Reactive protein level	Weighted median	55	-0.06	0.05	0.25	62	-0.08	0.06	0.19
	Weighted mode	55	-0.04	0.04	0.27	62	-0.08	0.06	0.17
	Simple mode	55	-0.02	0.09	0.83	62	0.16	0.2	0.43
	MR Egger	55	-0.05	0.05	0.28	62	-0.12	0.08	0.13
	IVW	55	-0.01	0.03	0.83	62	-0.06	0.06	0.27
white blood cell count	Weighted median	480	-0.06	0.05	0.23	744	-0.05	0.04	0.23
	Weighted mode	480	-0.02	0.07	0.76	744	-0.02	0.07	0.78
	Simple mode	480	-0.04	0.12	0.76	744	-0.04	0.12	0.75
	MR Egger	480	-0.13	0.06	0.04	744	-0.08	0.06	0.15
	IVW	480	-0.05	0.03	0.06	744	-0.05	0.03	0.07
lymphocyte cell count	Weighted median	486	-0.05	0.05	0.27	739	-0.09	0.04	0.04
	Weighted mode	486	-0.13	0.09	0.16	739	-0.19	0.08	0.01
	Simple mode	486	0.03	0.14	0.84	739	-0.01	0.13	0.92
	MR Egger	486	-0.05	0.06	0.39	739	-0.04	0.05	0.41
	IVW	486	-0.03	0.03	0.36	739	-0.03	0.03	0.26
neutrophil cell count	Weighted median	404	-0.05	0.05	0.3	656	-0.05	0.05	0.28
	Weighted mode	404	-0.03	0.06	0.65	656	-0.03	0.07	0.61
	Simple mode	404	-0.01	0.11	0.92	656	-0.01	0.12	0.9
	MR Egger	404	-0.13	0.07	0.06	656	-0.09	0.06	0.13
	IVW	404	-0.03	0.03	0.32	656	-0.03	0.03	0.27
monocyte cell count	Weighted median	472	0.06	0.04	0.16	716	0.05	0.04	0.17
	Weighted mode	472	0.05	0.05	0.25	716	0.04	0.04	0.41
	Simple mode	472	0	0.09	0.96	716	-0.03	0.1	0.78
	MR Egger	472	0.05	0.04	0.32	716	0.05	0.04	0.24
	IVW	472	-0.01	0.03	0.82	716	-0.01	0.02	0.76
eosinophil cell count	Weighted median	422	-0.02	0.04	0.62	645	-0.03	0.04	0.53
	Weighted mode	422	-0.04	0.06	0.48	645	-0.04	0.05	0.5
	Simple mode	422	-0.16	0.1	0.13	645	-0.06	0.1	0.6
	MR Egger	422	-0.03	0.05	0.58	645	-0.03	0.05	0.54
	IVW	422	-0.03	0.03	0.27	645	-0.03	0.03	0.28

Supplementary table 28. MR results after outlier removal for total cholesterol.
MR statistics for 5 different methods (weighted median, weighted mode, simple mode, MR Egger and inverse-variance weighted MR), two-sided p-values are presented. Presented p-values are not adjusted for multiple testing.

		Instrument cut-off: $P < 5\times10^{-8}$				Instrument cut-off: $P < 5\times10^{-5}$			
Method	Outliers removed	SNPs	beta	se	P	SNPs	beta	se	P
Weighted median	none	83	0.154	0.044	0.0004	178	0.149	0.044	0.0008
Weighted mode	none	83	0.132	0.041	0.002	178	0.109	0.04	0.008
Simple mode	none	83	0.017	0.091	0.85	178	0.034	0.105	0.75
MR Egger	none	83	0.132	0.053	0.01	178	0.147	0.052	0.005
IVW	none	83	0.091	0.032	0.005	178	0.077	0.033	0.02
Weighted median	IVW	75	0.155	0.042	0.0002	156	0.148	0.043	0.0006
Weighted mode	IVW	75	0.161	0.042	0.0003	156	0.106	0.043	0.01
Simple mode	IVW	75	-0.016	0.091	0.86	156	0.032	0.101	0.75
MR Egger	IVW	75	0.14	0.046	0.003	156	0.157	0.04	0.0001
IVW	IVW	75	0.092	0.029	0.001	156	0.071	0.026	0.007
Weighted median	Egger	76	0.166	0.044	0.0002	157	0.157	0.043	0.0003
Weighted mode	Egger	76	0.166	0.043	0.0003	157	0.145	0.043	0.001
Simple mode	Egger	76	-0.002	0.091	0.98	157	0.047	0.1	0.64
MR Egger	Egger	76	0.184	0.049	0.0004	157	0.2	0.042	5.5×10^{-6}
IVW	Egger	76	0.098	0.03	0.001	157	0.078	0.028	0.005

Supplementary table 29. MR results for reverse causality of genetic instruments for ALS on total cholesterol.
MR statistics for 5 different methods (weighted median, weighted mode, simple mode, MR Egger and inverse-variance weighted MR), two-sided p-values are presented. Presented p-values are not adjusted for multiple testing.

			Instrument cut-off: $P < 5\times10^{-8}$				Instrument cut-off: $P < 5\times10^{-5}$			
Exposure	Outcome	Method	SNPs	beta	se	P	SNPs	beta	se	P
ALS	Total cholesterol	Weighted median	9	0.02	0.03	0.4	75	0.02	0.02	0.2
ALS	Total cholesterol	Weighted mode	9	0.02	0.03	0.4	75	0.02	0.02	0.4
ALS	Total cholesterol	Simple mode	9	0.02	0.04	0.7	75	0.01	0.04	0.7
ALS	Total cholesterol	MR Egger	9	0.01	0.07	0.9	75	-0.03	0.04	0.5
ALS	Total cholesterol	IVW	9	0.03	0.02	0.2	75	0.01	0.01	0.5

Supplementary table 30. Multivariable MR results for total cholesterol, conditional on years of schooling.

MR statistics for two different methods (inverse-variance weighted MR and multivariable MR), two-sided p-values are presented. Presented p-values are not adjusted for multiple testing.

	Instrument cut-off: $P < 5\times10^{-8}$			Instrument cut-off: $P < 5\times10^{-5}$		
Method	beta	se	P	beta	se	P
IVW	0.09	0.03	0.005	0.08	0.03	0.02
Multivariable MR	0.15	0.04	2×10^{-4}	0.11	0.04	0.005

Supplementary table 31. MR instrument coverage

[table in Excel file]

Supplementary table 32. MR F-statistics

[table in Excel file]

Supplementary table 33. Wald ratios

[table in Excel file]

Supplementary table 34. Heterogeneity statistics

[table in Excel file]

References

1. Brooks, B. R., Miller, R. G., Swash, M. & Munsat, T. L. El Escorial revisited: Revised criteria for the diagnosis of amyotrophic lateral sclerosis. *Amyotroph. Lateral. Sc.* **1**, 293–299 (2000).
2. Beghi, E. et al. Incidence of ALS in Lombardy, Italy. *Neurology* **68**, 141–145 (2007).
3. Chiò, A. et al. Secular Trends of Amyotrophic Lateral Sclerosis: The Piemonte and Valle d'Aosta Register. *JAMA Neurol.* **74**, 1097–1104 (2017).
4. Logroscino, G. et al. Incidence of amyotrophic lateral sclerosis in southern Italy: a population based study. *J. Neurol. Neurosurg. Psychiatry* **76**, 1094–1098 (2005).
5. Huisman, M. H. B. et al. Population based epidemiology of amyotrophic lateral sclerosis using capture-recapture methodology. *J. Neurol. Neurosurg. Psychiatry* **82**, 1165–1170 (2011).
6. Dolinar, A., Koritnik, B., Glavač, D. & Ravnik-Glavač, M. Circular RNAs as Potential Blood Biomarkers in Amyotrophic Lateral Sclerosis. *Mol. Neurobiol.* **56**, 8052–8062 (2019).
7. Olsen, C. M. et al. Cohort profile: the QSkin Sun and Health Study. *Int. J. Epidemiol.* **41**, 929–929i (2012).
8. Olsen, C. M. et al. Does polygenic risk influence associations between sun exposure and melanoma? A prospective cohort analysis. *Br. J. Dermatol.* **183**, 303–310 (2020).
9. Osmanovic, A. et al. FIG4 variants in central European patients with amyotrophic lateral sclerosis: a whole-exome and targeted sequencing study. *Eur. J. Hum. Genet.* **25**, 324–331 (2017).
10. Osmanovic, A. et al. SPG7 mutations in amyotrophic lateral sclerosis: a genetic link to hereditary spastic paraplegia. *J. Neurol.* **267**, 2732–2743 (2020).
11. Brooks, B. R. El Escorial World Federation of Neurology criteria for the diagnosis of amyotrophic lateral sclerosis. Subcommittee on Motor Neuron Diseases/Amyotrophic Lateral Sclerosis of the World Federation of Neurology Research Group on Neuromuscular Diseases and the El Escorial 'Clinical limits of amyotrophic lateral sclerosis' workshop contributors. *J. Neurol. Sci.* **124 Suppl**, 96–107 (1994).

12. Benyamin, B. et al. Cross-ethnic meta-analysis identifies association of the GPX3-TNIP1 locus with amyotrophic lateral sclerosis. *Nat. Commun.* **8**, 611 (2017).
13. Nakamura, R. et al. A multi-ethnic meta-analysis identifies novel genes, including ACSL5, associated with amyotrophic lateral sclerosis. *Commun. Biol.* **3**, 526 (2020).
14. Hayashi, N. et al. Prognosis of amyotrophic lateral sclerosis patients undergoing tracheostomy invasive ventilation therapy in Japan. *J. Neurol. Neurosurg. Psychiatry* **91**, 285–290 (2020).
15. Kuriyama, S. et al. The Tohoku Medical Megabank Project: Design and Mission. *J. Epidemiol.* **26**, 493–511 (2016).
16. Chang, C. C. et al. Second-generation PLINK: rising to the challenge of larger and richer datasets. *Gigascience* **4**, 7 (2015).
17. Price, A. L. et al. Principal components analysis corrects for stratification in genome-wide association studies. *Nat. Genet.* **38**, 904–909 (2006).
18. Zhou, W. et al. Efficiently controlling for case-control imbalance and sample relatedness in large-scale genetic association studies. *Nat. Genet.* **50**, 1335–1341 (2018).
19. McCarthy, S. et al. A reference panel of 64,976 haplotypes for genotype imputation. *Nat. Genet.* **48**, 1279–1283 (2016).
20. Marchini, J., Howie, B., Myers, S., McVean, G. & Donnelly, P. A new multipoint method for genome-wide association studies by imputation of genotypes. *Nat. Genet.* **39**, 906–913 (2007).
21. Tunca, C. et al. Revisiting the complex architecture of ALS in Turkey: Expanding genotypes, shared phenotypes, molecular networks, and a public variant database. *Hum. Mutat.* **41**, e7–e45 (2020).
22. Debray, S. et al. Frequency of C9orf72 repeat expansions in amyotrophic lateral sclerosis: a Belgian cohort study. *Neurobiol. Aging* **34**, 2890.e7-2890.e12 (2013).
23. Goris, A. et al. No evidence for shared genetic basis of common variants in multiple sclerosis and amyotrophic lateral sclerosis. *Hum. Mol. Genet.* **23**, 1916–1922 (2014).
24. Traxinger, K., Kelly, C., Johnson, B. A., Lyles, R. H. & Glass, J. D. Prognosis and epidemiology of amyotrophic lateral sclerosis: Analysis of a clinic population, 1997-2011. *Neurol. Clin. Pract.* **3**, 313–320 (2013).

25. Byrne, S., Elamin, M., Bede, P. & Hardiman, O. Absence of consensus in diagnostic criteria for familial neurodegenerative diseases. *J. Neurol. Neurosurg. Psychiatry* **83**, 365–367 (2012).

26. Manichaikul, A. et al. Robust relationship inference in genome-wide association studies. *Bioinformatics* **26**, 2867–2873 (2010).

27. Cingolani, P. et al. Using Drosophila melanogaster as a Model for Genotoxic Chemical Mutational Studies with a New Program, SnpSift. *Front. Genet.* **3**, 35 (2012).

28. Kunkle, B. W. et al. Genetic meta-analysis of diagnosed Alzheimer's disease identifies new risk loci and implicates Aβ, tau, immunity and lipid processing. *Nat. Genet.* **51**, 414–430 (2019).

29. Kouri, N. et al. Genome-wide association study of corticobasal degeneration identifies risk variants shared with progressive supranuclear palsy. *Nat. Commun.* **6**, 7247 (2015).

30. Ferrari, R., Hernandez, D. G., Nalls, M. A. & Rohrer, J. D. Frontotemporal dementia and its subtypes: a genome-wide association study. *Lancet Neurol.* (2014).

31. Nalls, M. A. et al. Identification of novel risk loci, causal insights, and heritable risk for Parkinson's disease: a meta-analysis of genome-wide association studies. *Lancet Neurol.* **18**, 1091–1102 (2019).

32. Chen, J. A. et al. Joint genome-wide association study of progressive supranuclear palsy identifies novel susceptibility loci and genetic correlation to neurodegenerative diseases. *Mol. Neurodegener.* **13**, 41 (2018).

33. Dolzhenko, E. et al. Detection of long repeat expansions from PCR-free whole-genome sequence data. *Genome Res.* **27**, 1895–1903 (2017).

34. Zhu, Z. et al. Integration of summary data from GWAS and eQTL studies predicts complex trait gene targets. *Nat. Genet.* **48**, 481–487 (2016).

35. van der Ende, E. L. et al. Novel CSF biomarkers in genetic frontotemporal dementia identified by proteomics. *Ann. Clin. Transl. Neurol.* **6**, 698–707 (2019).

36. Llano, D. A., Bundela, S., Mudar, R. A., Devanarayan, V., & Alzheimer's Disease Neuroimaging Initiative (ADNI). A multivariate predictive modeling approach reveals a novel CSF peptide signature for both Alzheimer's Disease state classification and for predicting future disease progression. *PLoS One* **12**, e0182098 (2017).

37. Chuang, Y.-H. et al. Longitudinal Epigenome-Wide Methylation Study of Cognitive Decline and Motor Progression in Parkinson's Disease. *J. Parkinsons. Dis.* **9**, 389–400 (2019).

38. Nicolas, A. et al. Genome-wide Analyses Identify KIF5A as a Novel ALS Gene. *Neuron* **97**, 1268-1283.e6 (2018).

39. Cirulli, E. T. et al. Exome sequencing in amyotrophic lateral sclerosis identifies risk genes and pathways. *Science* **347**, 1436–1441 (2015).

40. Freischmidt, A. et al. Haploinsufficiency of TBK1 causes familial ALS and fronto-temporal dementia. *Nat. Neurosci.* **18**, 631–636 (2015).

41. van Es, M. A. et al. Genome-wide association study identifies 19p13.3 (UNC13A) and 9p21.2 as susceptibility loci for sporadic amyotrophic lateral sclerosis. *Nat. Genet.* **41**, 1083–1087 (2009).

42. Diekstra, F. P. et al. C9orf72and UNC13A are shared risk loci for amyotrophic lateral sclerosis and frontotemporal dementia: A genome-wide meta-analysis. *Ann. Neurol.* **76**, 120–133 (2014).

43. Brown, A.-L. et al. Common ALS/FTD risk variants in UNC13A exacerbate its cryptic splicing and loss upon TDP-43 mislocalization. *bioRxiv* (2021) doi:10.1101/2021.04.02.438170.

44. Ma, X. R. et al. TDP-43 represses cryptic exon inclusion in FTD/ALS gene UNC13A. *bioRxiv* (2021) doi:10.1101/2021.04.02.438213.

45. van Rheenen, W. et al. Genome-wide association analyses identify new risk variants and the genetic architecture of amyotrophic lateral sclerosis. *Nat. Genet.* **48**, 1043–1048 (2016).
