# Supplementary material for: Common and rare variant association analyses in amyotrophic lateral sclerosis identify 15 risk loci with distinct genetic architectures and neuron-specific biology
Source: Nat Genet. 2021 Dec 6;53(12):1636–48. doi: 10.1038/s41588-021-00973-1 (PMC8648564; doi:10.1038/s41588-021-00973-1)
Supplement: Supplementary file 2 — Reporting Summary [file 41588_2021_973_MOESM2_ESM.pdf]

nature research

Corresponding author(s): Wouter van Rheenen
Jan H. Veldink

Last updated by author(s): Sep 10, 2021

Reporting Summary

Nature Research wishes to improve the reproducibility of the work that we publish. This form provides structure for consistency and transparency in reporting. For further information on Nature Research policies, see our Editorial Policies and the Editorial Policy Checklist.

Statistics

For all statistical analyses, confirm that the following items are present in the figure legend, table legend, main text, or Methods section.

n/a	Confirmed	
☐	☒	The exact sample size (*n*) for each experimental group/condition, given as a discrete number and unit of measurement
☒	☐	A statement on whether measurements were taken from distinct samples or whether the same sample was measured repeatedly
☐	☒	The statistical test(s) used AND whether they are one- or two-sided<br>*Only common tests should be described solely by name; describe more complex techniques in the Methods section.*
☐	☒	A description of all covariates tested
☐	☒	A description of any assumptions or corrections, such as tests of normality and adjustment for multiple comparisons
☐	☒	A full description of the statistical parameters including central tendency (e.g. means) or other basic estimates (e.g. regression coefficient) AND variation (e.g. standard deviation) or associated estimates of uncertainty (e.g. confidence intervals)
☐	☒	For null hypothesis testing, the test statistic (e.g. *F*, *t*, *r*) with confidence intervals, effect sizes, degrees of freedom and *P* value noted<br>*Give P values as exact values whenever suitable.*
☐	☒	For Bayesian analysis, information on the choice of priors and Markov chain Monte Carlo settings
☒	☐	For hierarchical and complex designs, identification of the appropriate level for tests and full reporting of outcomes
☐	☒	Estimates of effect sizes (e.g. Cohen's *d*, Pearson's *r*), indicating how they were calculated

Our web collection on statistics for biologists contains articles on many of the points above.

Software and code

Policy information about availability of computer code

Data collection	No software was used to collect the data.
Data analysis	The following software packages have been used for data analyses: R v3.6.3 with additional packages tidyverse v1.3.0, data.table v1.14.0, ggplot2 v3.3.3, MASS v7.3.53, SNPRelate v1.26.0, logistf v1.24, coloc v5.1.0, twoSampleMR v0.5.6, RadialMR v1.0, MVMR v0.3, survival v3.1.8, coxme v2.2.16, survminer v0.4.9 (www.r-project.org), Python v3.7 with additional modules pandas v1.1.3, numpy v1.18.1, scipy v1.4.1, CpGtools v1.0.9, matplotlib v3.1.3, pyliftover v0.4, pyhpo v2.5.0 (www.anaconda.org), GenomeStudio v2.0 (https://emea.illumina.com/techniques/microarrays/array-data-analysis-experimental-design/genomestudio.html), GCTA v1.93.2beta (cnsgenomics.com/software/gcta), EIGENSOFT v6.1.4 (www.github.com/DreichLab/EIG), SNPTEST v2.5.4-beta3 (https://www.well.ox.ac.uk/~gav/snptest/), PLINK v1.9 (www.cog-genomics.org/plink2), Michigan Imputation Server (https://imputationserver.sph.umich.edu), EAGLE v2.3 through Michigan Imputation Server (https://imputationserver.sph.umich.edu), SAIGE v0.29.1 (www.github.com/weizhouUMICH/SAIGE), METAL 2011-03-25 (https://genome.sph.umich.edu/wiki/METAL), SnpSift 4.3p, (https://pcingola.github.io/SnpEff), ANNOVAR version 2017-07-17 for LRT, Polyphen-2, MutationTaster2, Mutation Assessor, PROVEAN and SIFT (https://annovar.openbioinformatics.org/), Polyphen-2 (http://genetics.bwh.harvard.edu/pph2/), MutationTaster2, (http://www.mutationtaster.org/), Mutation Assessor release 3 (http://mutationassessor.org/r3/), PROVEAN v1.1 (http://provean.jcvi.org/index.php), SIFT v6.2.1 (https://sift.bii.a-star.edu.sg/), SnpEff 4.3p (https://pcingola.github.io/SnpEff), LDSC v1.0.1 (www.github.com/bulik/ldsc), ExpansionHunter v4 (www.github.com/Illumina/ExpansionHunter), ExpansionHunter denovo, (www.github.com/Illumina/ExpansionHunterDenovo), SMR (www.cnsgenomics.com/software/smr), MAGMA v1.6 (www.ctg.cncr.nl/software/magma), FUMA (https://fuma.ctglab.nl/), FUMA Cell-type (https://fuma.ctglab.nl/celltype), summary-BayesR (https://cnsgenomics.com/software/gctb/#SummaryBayesianAlphabet), S-PrediXcan (https://github.com/hakyimlab/MetaXcan), TWAS (http://gusevlab.org/projects/fusion/)

For manuscripts utilizing custom algorithms or software that are central to the research but not yet described in published literature, software must be made available to editors and reviewers. We strongly encourage code deposition in a community repository (e.g. GitHub). See the Nature Research guidelines for submitting code & software for further information.

Data

Policy information about availability of data

All manuscripts must include a data availability statement. This statement should provide the following information, where applicable:

- Accession codes, unique identifiers, or web links for publicly available datasets
- A list of figures that have associated raw data
- A description of any restrictions on data availability

GWAS summary statistics generated in this study are publicly available in the NHGRI-EBI GWAS Catalog (accession IDs: GCST90027163 and GCST90027164 for cross-ancestry and European ancestries meta-analyses respectively) and through the Project MinE website (https://www.projectmine.com/research/download-data/). Summary statistics of the rare variant burden analyses and eQTL/mQTL summary-based Mendelian randomization analyses are available through the Project MinE website.
The following publicly available datasets were used in this project:
WellcomeTrust case-control consortium: www.wtccc.org.uk
dbGaP datasets:
phs000101.v3.p1: NIH Genome-Wide Association Studies of Amyotrophic Lateral Sclerosis
phs000126.v1.p1: CIDR: Genome Wide Association Study in Familial Parkinson Disease (PD)
phs000196.v1.p1: Genome-Wide Association Study of Parkinson Disease: Genes and Environment
phs000344.v1.p1: Genome-Wide Association Study of Amyotrophic Lateral Sclerosis in Finland
phs000336: A Genome-Wide Association Study of Lung Cancer Risk
phs000346: Genome-wide association study for Bladder Cancer Risk
phs000789: Collaborative Study of Genes, Nutrients and Metabolites (CSGNM)
phs000206: Whole Genome Scan for Pancreatic Cancer Risk in the Pancreatic Cancer Cohort Consortium and Pancreatic Cancer Case-Control Consortium (PanScan)
phs000297: eMERGE Network Study of the Genetic Determinants of Resistant Hypertension
phs000652: Cohort-Based Genome-Wide Association Study of Glioma (GliomaScan)
phs000869: Barrett's and Esophageal Adenocarcinoma Genetic Susceptibility Study (BEAGESS)
phs000812: The Breast and Prostate Cancer Cohort Consortium (BPC3) GWAS of Aggressive Prostate Cancer and ER- Breast Cancer
phs000428: Genetics Resource with the Health and Retirement Study
phs000360.v3: eMERGE Network Genome-Wide Association Study of Red Cell Indices, White Blood Count (WBC) Differential, Diabetic Retinopathy, Height, Serum Lipid Levels, Specifically Total Cholesterol, HDL (High Density Lipoprotein), LDL (Low Density Lipoprotein), and Triglycerides, and Autoimmune Hypothyroidism.
phs000893.v1: Genome-Wide Association Study of Endometrial Cancer in the Epidemiology of Endometrial Cancer Consortium (E2C2)
phs000168.v2: National Institute on Aging - Late Onset Alzheimer's Disease Family Study: Genome-Wide Association Study for Susceptibility Loci
phs000092.v1: Study of Addiction: Genetics and Environment (SAGE)
phs000864.v1: Genomic Predictors of Combat Stress Vulnerability and Resilience
phs000170.v2: A Genome-Wide Association Study on Cataract and HDL in the Personalized Medicine Research Project Cohort
phs000431.v2: IgA Nephropathy GWAS on Individuals of European Ancestry (IGANGWAS2)
phs000237.v1: Northwestern NUgene Project: Type 2 Diabetes
phs000169.v1: Whole Genome Association Study of Visceral Adiposity in the Health Aging and Body Composition (Health ABC) Study
phs000982.v1: Genetic Analysis of Psoriasis and Psoriatic Arthritis: GWAS of Psoriatic Arthritis
phs000289.v2: National Human Genome Research Institute (NHGRI) GENEVA Genome-Wide Association Study of Venous Thrombosis (GWAS of VTE)
phs000634.v1: National Cancer Institute (NCI) Genome Wide Association Study (GWAS) of Lung Cancer in Never Smokers
phs000274.v1: Genome-Wide Association Study of Celiac Disease
phs001172.v1: National Institute of Neurological Disorders and Stroke (NINDS) Parkinson's Disease
phs000389.v1: GEnetics of Nephropathy - an International Effort (GENIE) GWAS of Diabetic Nephropathy in the UK GoKinD and All-Ireland Cohorts
phs000460.v1: Genetics of 24 hour urine composition
phs000138.v2: GWAS for Genetic Determinants of Bone Fragility in European-American Premenopausal Women
phs000394.v1: Autopsy-Confirmed Parkinson Disease GWAS Consortium (APDGC)
phs000948.v1: Genetic Discovery and Application in a Clinical Setting: Continuing a Partnership (eMERGE Phase II)
phs000630.v1: Exome Chip Study of NIMH Controls
phs000678.v1: A Family-Based Study of Genes and Environment in Young-Onset Breast Cancer
phs000351.v1: National Cancer Institute Genome-Wide Association Study of Renal Cell Carcinoma
phs000314.v1: Genetic Associations in Idiopathic Talipes Equinovarus (Clubfoot) - GAIT
phs000147.v3: Cancer Genetic Markers of Susceptibility (CGEMS) Breast Cancer Genome-wide Association Study (GWAS) - Primary Scan: Nurses' Health Study - Additional Cases: Nurses' Health Study 2
phs000882.v1: National Cancer Institute (NCI) Prostate Cancer Genome-Wide Association Study for Uncommon Susceptibility Loci (PEGASUS)
phs000238.v1: National Eye Institute Glaucoma Human Genetics Collaboration (NEIGHBOR) Consortium Glaucoma Genome-Wide Association Study
phs000397.v1: National Institute on Aging (NIA) Long Life Family Study (LLFS)
phs000421.v1: A Genome-Wide Association Study of Fuchs' Endothelial Corneal Dystrophy (FECD)
phs000142.v1: A Whole Genome Association Scan for Myopia and Glaucoma Endophenotypes using Twin Studies
phs000303.v1: Genetic Epidemiology of Refractive Error in the KORA (Kooperative Gesundheitsforschung in der Region Augsburg) Study
phs000125.v1: CIDR: Collaborative Study on the Genetics of Alcoholism Case Control Study
phs001039.v1: International Age-Related Macular Degeneration Genomics Consortium - Exome Chip Experiment
phs000187.v1: High Density SNP Association Analysis of Melanoma: Case-Control and Outcomes Investigation
phs000101.v5: Genome-Wide Association Study of Amyotrophic Lateral Sclerosis
phs002068.v1.p1: Sporadic ALS Australia Systems Genomics Consortium (SALSA-SGC)

Field-specific reporting

Please select the one below that is the best fit for your research. If you are not sure, read the appropriate sections before making your selection.

☒ Life sciences ☐ Behavioural & social sciences ☐ Ecological, evolutionary & environmental sciences

Life sciences study design

All studies must disclose on these points even when the disclosure is negative.

Sample size	We did not pre-specify a sample size given the wide distribution of allele-frequencies and effect-sizes typically seen in GWAS. We have included all largest available control cohorts of European ancestries matched for genotyping platform that were available throught dbGaP to achieve a ~1:10 case:control ratio per stratum at maximum. This ratio was roughly determined based on power calculations (https://zzz.bwh.harvard.edu/gpc/cc2.html) that indicated that including even more controls would yield a limited increase in power and we expected increasing challenges introduced by batch effects when more smaller control cohorts were included.
Data exclusions	Individuals and genotypes were excluded from the analysis following rigorous quality control as described in the methods section.
Replication	We replicated our SNP associations in 2 independent GWAS in ALS patients and control subjects from Asian ancestries. All genome-wide significant SNPs showed an identical direction of effect. Given our effort to design a large-scale GWAS including all available individual level genotype data in ALS globally (including the newly genotyped ALS patients), there are no more independent datasets for replication in European ancestries.
Randomization	For newly genotyped case-control cohorts, samples were randomized by case-control status before hybridization on SNP genotyping arrays. For case-only and control-only cohorts (Supplementary table 1) samples could not randomized before hybridization. We therefore matched these cohorts based on genotyping platform and included Illumina genotyping arrays only. We subsequently corrected for genotyping platform as confounder by the stratified analyses creating 6 separate strata. Furthermore, principal components and a genetic relationship matrix were included as covariates in the statistical analyses to correct for structure in the data due to technical artifacts and population stratification. We assessed residual confounding of test- statistics by LD-Score Regression.
Blinding	Individuals involved in sample ascertainment were blinded for genotypes, individuals involved in genotyping individuals were blinded for phenotypes.

Reporting for specific materials, systems and methods

We require information from authors about some types of materials, experimental systems and methods used in many studies. Here, indicate whether each material, system or method listed is relevant to your study. If you are not sure if a list item applies to your research, read the appropriate section before selecting a response.

Materials & experimental systems

n/a	Involved in the study	
☒	☐	Antibodies
☒	☐	Eukaryotic cell lines
☒	☐	Palaeontology and archaeology
☒	☐	Animals and other organisms
☐	☒	Human research participants
☒	☐	Clinical data
☒	☐	Dual use research of concern

Methods

n/a	Involved in the study	
☒	☐	ChIP-seq
☒	☐	Flow cytometry
☒	☐	MRI-based neuroimaging

Human research participants

Policy information about studies involving human research participants

Population characteristics	Included were 8 strata of ALS patients and controls in the GWAS. The analyses were stratified for genotyping platform and reported ancestries:<br>stratum 1: 2,254 ALS patients, 11,155 controls, IlluminaCoreExome, European ancestries<br>stratum 2: 1,458 ALS patients, 2,043 controls, Illumina 317K, European ancestries<br>stratum 3: 1,701 ALS patients, 2,555 controls, Illumina 370K, European ancestries<br>stratum 4: 3,394 ALS patients, 42,402 controls, Illumina550K/Illumina610K/Illumina660K, European ancestries<br>stratum 6: 14,402 ALS patients, 32,094 controls, IlluminaOmniExpress/Illumina2M, European ancestries<br>stratum 7: 3,996 ALS patients, 20,632 controls, IlluminaGSA, European ancestries<br>stratum 8: 1,234 ALS patients, 2850 controls, IlluminaHumanOmniZhongHua, Chinese ancestries<br>stratum 9: 1,173 ALS patients, 8,925 controls, IlluminaHumanOmniExpressExome, Japanese ancestries<br>A more detailed description is provided in Supplementary Table 1 (numbers stratified by cohort) and the Supplementary Text (for newly genotyped individuals). The European ancestries for individuals in stratum 1-7 were inferred from PCA (Supplementary Figure 18).
Recruitment	The Supplementary Text describes recruitement for each newly genotyped or sequenced cohort.
Ethics oversight	Local ethics committee of the Medical Faculty of Friedrich Schiller University Jena, Jena, Germany.<br>Ethical Committee of Città della Salute Hospital, Torino, Italy.

Institutional Review Board of the Azienda Sanitaria Locale, Lecce, Italy.
Ethics Committee, Stockholm, Sweden
University Medical Center Utrecht Medical Ethics Committee, Utrecht, The Netherlands.
Review Ethics Board Office at McGill University Health Center, Montreal, Canada
Medical Research Ethics Committee of "Assistance Publique-Hôpitaux de Paris", Paris, France.
Ethics committee of Tours Hospital, Tours, France.
Ethics committee of Limoges University Hospital, Limoges, France.
National Medical Ethics Committee of Republic of Slovenia, Slovenia.
Sydney South West Area Health Service Human Research Ethics Committee, Australia.
Human Research Ethics Committee at the QIMR Berghofer Medical Research Institute, Autralia.
Charité Universitätsmedizin, Berlin Medical Ethics Committee, Berlin, Germany.
Medical Ethics Committee of Hannover Medical School, Hannover, Germany.
Beaumont Hospital Research & Ethics Committee, Dublin, Ireland.
Ethics Committee of the IRCCS Istituto Auxologico Italiano, Milan, Italy.
Local ethical committee of Buyanov city hospital, Moscow, Russia.
Ethics Committee of the School of Medicine, University of Belgrade.
Yorkshire and the Humber - Sheffield Research Ethics Committee, UK.
Instituional review board of Cedars-Sinai, Los Angeles, USA.
Institutional review board of the University of California at Los Angeles, USA.
Trent University Medical Ethics Committee, UK.
Ethics Committee on Research with Human Participants (INAREK) at Bogazici University, Istanbul, Turkey.
Ethical Committee of University Hospital Leuven, Leuven, Belgium.
"Comité de Ética de la Investigación del Hospital Carlos III", Madrid, Spain.
Bellvitge University Hospital Ethics Committee, Barcelona, Spain.
Committee for the Protection of Human Subjects in Research of the University of Massachusetts Medical School, Worcester, USA.
Regional Ethical Review Board in Umeå, Sweden.
Hadassah University Hospital IRB board, Hadssah, Israel.
The Institutional Review Board of Tel Aviv Sourasky Medical Center, Tel Aviv, Israel.
The Local Research Ethics Committee at the Faculty of Medicine, University of Lisbon, Lisbon, Portugal.
Kantonale Ethikkomission des Kantons St. Gallen, Switzerland

Note that full information on the approval of the study protocol must also be provided in the manuscript.
