# Supplementary material for: Common and rare variant association analyses in amyotrophic lateral sclerosis identify 15 risk loci with distinct genetic architectures and neuron-specific biology
Source: Nat Genet. 2021 Dec 6;53(12):1636–48. doi: 10.1038/s41588-021-00973-1 (PMC8648564; doi:10.1038/s41588-021-00973-1)
Supplement: Supplementary file 3 — Peer Review Information [file 41588_2021_973_MOESM3_ESM.pdf]

natureresearch

Peer Review Information

Journal: Nature Genetics
Manuscript Title: Common and rare variant association analyses in Amyotrophic Lateral Sclerosis identify 15 risk loci with distinct genetic architectures and neuron-specific biology
Corresponding author name(s): van Rheenen

Reviewer Comments & Decisions:

Decision Letter, initial version:

20th Apr 2021

Dear Dr van Rheenen,

Your Article, "Common and rare variant association analyses in Amyotrophic Lateral Sclerosis identify 15 risk loci with distinct genetic architectures and neuron-specific biology" has now been seen by 3 referees. You will see from their comments below that while they find your work of interest, some important points are raised. We are interested in the possibility of publishing your study in Nature Genetics, but would like to consider your response to these concerns in the form of a revised manuscript before we make a final decision on publication.

To guide the scope of the revisions, we discuss the referee reports in detail with a view to identifying key priorities that should be addressed in revision. As you will see from these comments, reviewer #1 thinks that the study represents an important advance; reviewer #2 has detailed comments which could improve the clarity of the results and methods; reviewer #3 provides a few suggestions to improve the genomic analyses and functional validation (point #4. Please note that editorially we don't require functional validation as a strict condition for publication). Please address all referee points as thoroughly as you can.

We therefore invite you to revise your manuscript taking into account all reviewer and editor comments. Please highlight all changes in the manuscript text file. At this stage we will need you to upload a copy of the manuscript in MS Word .docx or similar editable format.

We are committed to providing a fair and constructive peer-review process. Do not hesitate to contact us if there are specific requests from the reviewers that you believe are technically impossible or unlikely to yield a meaningful outcome.

When revising your manuscript:

*1) Include a “Response to referees” document detailing, point-by-point, how you addressed each referee comment. If no action was taken to address a point, you must provide a compelling argument. This response will be sent back to the referees along with the revised manuscript.

*2) If you have not done so already please begin to revise your manuscript so that it conforms to our Article format instructions, available
here.
Refer also to any guidelines provided in this letter.

*3) Include a revised version of any required Reporting Summary:
https://www.nature.com/documents/nr-reporting-summary.pdf
It will be available to referees (and, potentially, statisticians) to aid in their evaluation if the manuscript goes back for peer review.
A revised checklist is essential for re-review of the paper.

Please be aware of our guidelines on digital image standards.

Please use the link below to submit your revised manuscript and related files:

[REDACTED]

Note: This URL links to your confidential home page and associated information about manuscripts you may have submitted, or that you are reviewing for us. If you wish to forward this email to co-authors, please delete the link to your homepage.

We hope to receive your revised manuscript within eight to ten weeks. If you cannot send it within this time, please let us know.

Please do not hesitate to contact me if you have any questions or would like to discuss these revisions further.

Nature Genetics is committed to improving transparency in authorship. As part of our efforts in this direction, we are now requesting that all authors identified as ‘corresponding author’ on published papers create and link their Open Researcher and Contributor Identifier (ORCID) with their account on the Manuscript Tracking System (MTS), prior to acceptance. ORCID helps the scientific community achieve unambiguous attribution of all scholarly contributions. You can create and link your ORCID from the home page of the MTS by clicking on ‘Modify my Springer Nature account’. For more information please visit please visit <a
href="http://www.springernature.com/orcid">www.springernature.com/orcid.

We look forward to seeing the revised manuscript and thank you for the opportunity to review your work.

Sincerely,

Wei Li, PhD
Senior Editor
Nature Genetics
One New York Plaza, 47th Fl.
New York, NY 10004, USA
www.nature.com/ng

Reviewers' Comments:

Reviewer #1:
Remarks to the Author:
This is easily one of the most interesting and important genetic studies performed in recent years in ALS. The authors analyze a very large cohort for common variant associations and a smaller one for rare variant burden analyses. Importantly, they prioritize signals based on patterns of gene co expression and methylation data from cortical samples.

These novel approaches yield novel signals and some key insights. Notably, the data clearly implicate both vesicle mediated transport and autophagy, show evidence of independent common and rare variant associations for two ALS genes implicated in earlier rare variant studies (NEK1 and TBK1), and suggest strong cell autonomous effects in glutamatergic neurons of genetic variation on risk. The last observation is of particular interest in being in contrast with some experimental data and in apparently separating predisposition and disease modification.

Collectively these results represent an important advance in ALS genetics. The methodologies are appropriate and the results appear robust. My only suggestion is that the authors evaluate the risk associations they have detected for association with clinical outcomes (e.g. progression site of onset) to help explore the distinction between predisposition and disease course. While the the data will likely only be available for subset of samples such a targeted evaluation only of associated variants may be informative based only only a proportion of the samples and would add significantly to the interest of the study.

Reviewer #2:
Remarks to the Author:
Van Rheenen et al present a trans-ancestry GWAS of ALS, which identifies 15 genome-wide significant loci (7 novel). They prioritize causal genes at 14 loci, through integrating whole-genome sequence data from ALS patients, rare expansion data and QTL data. ALS risk alleles were enriched in genes expressed in brain tissues, glutamatergic neurons and brain co-expression networks annotated to vesicle mediated transport and autophagy. Mendelian randomization analyses suggested a causal role of high cholesterol on ALS.

I commend the authors for elegantly integrating layers of genomic data and examining convergence of evidence to prioritize likely causal genes from within these 15 loci. The comparisons drawn between

ALS and other neurodegenerative diseases are insightful. I don’t have any major concerns but I do have a lot of minor recommendations, which I believe would substantially improve the clarity of the manuscript.

Results

As I read through the results, there were several questions I had which only became clear after reading the methods section. It would be useful if these details could be added into the results:
-information on the expected proportion of sporadic and familial ALS cases in the study
-the set of sequenced individuals is not independent from the GWAS
-the sample sizes of the new European ALS cases and controls added in this study, the Asian ancestry cases and controls, and the total cross-ancestry meta-analysis
-some of the index SNPs were not present in the Asian samples due to low frequency (Table 1)
-sample sizes of the QTL datasets used would demonstrate their power

Fig 1 – this paper does a great job of dissecting the genetic architecture at the GWAS loci and it would be worth getting this information onto the plot through some sort of color legend/ symbols. Highlighting loci that also have known familial pathogenic mutations would also be a nice addition.

Table 1 – including the frequencies in Asian populations would demonstrate why some of the SNPs were not present. It seems that one SNP was just not present in the Asian samples; is there a proxy SNP in high LD that could be used to perform a meta-analysis? It would be more accurate to remove the “cross-ancestry” results for those that were not present in the Asian samples.

I did not see the total LDSC SNP heritability of ALS.

The “rare variant association analysis” would be better described as “rare variant gene/ transcript burden analysis” or something along those lines, since it does not examine rare variants in the 5’UTR or conserved regions.

The calculation of the PoPS score is mentioned in the results but is not explained and none of the scores are presented in the text, only on the trackplots. I suggest removing PoPS from the results, unless the scores will be highlighted for some genes. If PoPS is included in the results, a brief explanation of how it is calculated and interpreted is needed, otherwise this should be included in the methods.

For the 2 loci where the rare and common variant signals converge (NEK1 and TBK1), I think some elaboration is warranted in this section. What effect do the common risk increasing alleles have on gene expression and is that effect in brain, blood or both? What type of rare variant burden is found at these genes or what is the functional mechanism underlying the rare variant burden?

Similarly, for the genes in other loci implicated by eQTL or mQTLs, were the effects in blood, brain or a mix?

A note is warranted on the HLA, since no genes were prioritized here through any of the mechanisms tested and that is only clear from viewing the trackplot.

Fig 2a and 3 – it seems that both the colors and size of the squares correspond to the P value. I suggest using color only for simplicity, otherwise readers look for another layer of information that

isn't present.

For the MAGMA tissue and single-cell enrichment analysis, how many GTEx tissues were tested? The figure only shows a limited number but there are over 50.

I was surprised that there was no enrichment in peripheral nervous tissue or muscle. Is that expected based on disease biology? Could this be because the enrichment test is within genes expressed in brain tissue (>50% of genes) rather than genes differentially expressed in brain versus other tissues, and therefore the brain tissue enrichment analyses may have greater power? I wonder if the same pattern of enrichment would be observed for sets of genes differentially expressed between tissues?

Fig 4 – the number of instruments used at each cut-off would be informative here. Are the outliers removed for these results? Also, a quantification of the effect of unit increase in cholesterol on odds of ALS would in the text would aid interpretation of the magnitude of effect.

Caution is needed with the language used when reporting the Mendelian randomization results, because the method suggests causality but does not prove it.

Methods
The data description section needs an overview of how the cases were ascertained (collected specifically for case/ control studies of ALS, family studies, biobanks, postmortem studies) and how the diagnoses of ALS were made. Is it expected that 5% of these cases have familial ALS driven by a rare pathogenic mutation? This warrants a comment. It would also benefit from some brief details about the Asian ancestry studies (country of ascertainment, similarities or differences with the EUR studies).

I'm not sure why the Supplementary Tables are split between the pdf and excel file. It would be better to have them all together in the excel file. The tables in the excel file have no titles or legends. There's also a lot of Supplementary Figs and Tables which is a bit overwhelming. I don't think all of these are needed eg Supplementary Figs 2-17 and Supplementary Tables 4-18 present some of the same information. Could all the information be consolidated into figures? I think such figures and the Track plots would be better presented as Supplementary Data files and this would make the paper easier for the reader to navigate.

Was the WGS sample comprised of familial cases or a mixture of familial and sporadic? In the WGS QC section, I'm guessing non-Europeans were removed but this sentence seems unfinished "Outliers from the European population (HapMap3: > 10 SD on PC1-4, 1KG: > 4 SD on PC1-4)."

Where were the disruptive variant annotations obtained from?

Please add the number of tests and the multiple testing correction threshold for all analyses (burden tests, QTL analyses, MAGMA enrichments, MR).

Reviewer #3:
Remarks to the Author:
In their manuscript entitled "Common and rare variant association analyses in Amyotrophic Lateral

Sclerosis identify 15 risk loci with distinct genetic architectures and neuron-specific biology" by van Rheenen, van der Spek, Bakker et al, the authors describe a comprehensive cross-ancestry meta-analyses of GWAS data from 29,612 ALS patients and 122,656 controls, combined with 8,953 whole-genome sequenced individuals (6,538 ALS patients, 2,415 controls) and a large cortex-derived eQTL dataset. First, the authors identified 15 risk loci significantly associated with ALS; ALS associated signals indicate a critical role in vesicle mediated transport and autophagy. Second, the authors describe distinct enrichment patterns across brain regions and cell-types, with evidence supporting cell-autonomous disease initiation in glutamatergic neurons. Third, and perhaps most surprisingly, Mendelian randomization analyses indicated a causal role for high cholesterol levels. Revisions to independently test the predictions described in this manuscript, either through analysis of imputed and convergent expression patterns of ALS risk genes, validation in electronic health records, and/or functional validation of key molecular risk genes would improve the impact of this systematic and thorough analyses. With what I hope to be straightforward new analyses, I expect that this report will be suitable for Nature Genetics.

Major concerns:

1. Genomic analyses. The authors identify 15 common risk loci for ALS (8 previously reported), independently identify NEK as both a common and rare variant, and associate coding variants with 3 of the 15 common risk loci. They further conduct multi-omic analyses to integrate GWAS and eQTL datasets, and also explore enrichments across tissues, cell types, and biological pathways. This is a rigorous analysis and I have only minor suggestions:
a. Do the authors specifically include spinal cord tissue in the eQTL analyses and motor neuron signatures in the cell type analyses?
b. The analyses integrated eQTL information with GWAS by colocalization and SMR to estimate the association between intermediate gene expression levels and phenotypes. Are the results consistent with an alternative method (e.g. PrediXcan, TWAS)?
c. Are risk variants enriched for any regulatory motifs? To what extent are risk variants and genes in regions of evolutionary constraint?
d. Is polygenic risk burden altered in rare variant carriers? Do clinical characteristics (age of onset, rate of disease progression) differentiate rare variant carriers?

2. Disease-specificity: The authors did a great job exploring the extent of genetic overlap with co-morbid disorders (FTD, AD, PD), particularly in applying existing bulk and scRNAseq datasets to discuss the expression patterns of ALS-specific and shared disease genes between neural cell types. Could this be expanded to consider expression across human development?
a. The association between GWAS target gene expression and disease could be explored using post-mortem datasets (psychiatric, neurodegenerative and non-brain related) to further explore and validate the specificity of the results.)

3. Replication: If the authors used all available ALS GWAS datasets in their meta-analysis, can the issue of replication be addressed using PRS leave-one-out analyses to demonstrate a higher burden of common risk variants associated with ALS cases, compared with controls, across all the cohorts. Alternately, is it possible to conduct ALS-by-proxy in other existing datasets (eg. 23andme)?
a. It would be interesting, to evaluate the burden of ALS variant and mutations as a function of other clinical measures in HER.

4. Functional validation: The manuscript describes a wealth of analyses describing potential

mechanisms by which common and rare ALS variants impact disease risk. Can any of these hypotheses be specifically evaluated? For example:
a. The authors posit that missense mutations explain the GWAS effect at four genes (SOD1, KIF5A, CFAP410, NEK1). Can they conduct in vitro experiments to explore whether these missense mutations lead to decreased protein function, activity or mutant gain of function?
b. Do disease relevant perturbations in gene expression (in vitro or in xenopus/zebrafish/mouse models) impact vesicle mediated transport, autophagy, and/or survival? Particularly in glutamatergic neurons?
c. Can the causal role of top colocalized risk variants by demonstrated through CRISPR-editing or reporter assays (up to and including MPRA)?

Author Rebuttal to Initial comments

Dear dr. Li,

We thank you and the reviewers for the careful review of our manuscript entitled "*Common and rare variant association analyses in Amyotrophic Lateral Sclerosis identify 15 risk loci with distinct genetic architectures and neuron-specific biology*" (NG-A57139) and the opportunity to submit a revised version. The reviewers have raised important and interesting questions that we now address in the revised manuscript.

Both reviewer #1 and #3 suggested to investigate the disease modifying effects of the genome-wide significant loci and rare variants in ALS risk genes. Therefore, we have collected prospective survival data on 6,095 of the 6,538 whole-genome sequenced ALS patients, a cohort unique in its size for these phenotypic data. To describe the results of these analyses we have added a new paragraph and figure to the main text.

Below you will find the point-by-point response to the points raised by the reviewers where our answers are written in blue. The corresponding changes in the manuscript are highlighted in yellow. We have also corrected some remaining typos and made minor textual corrections.

We think the revisions have resulted in a much-improved manuscript and hope you will find the revised version of our manuscript suitable for publication in *Nature Genetics*.

Kind Regards,

Prof. Jan Veldink Dr. Wouter van Rheenen

Reviewer #1:
Remarks to the Author:
This is easily one of the most interesting and important genetic studies performed in recent years in ALS. The authors analyze a very large cohort for common variant associations and a smaller one for rare variant burden analyses. Importantly, they prioritize signals based on patterns of gene co expression and methylation data from cortical samples.

These novel approaches yield novel signals and some key insights. Notably, the data clearly implicate both vesicle mediated transport and autophagy, show evidence of independent common and rare variant associations for two ALS genes implicated in earlier rare variant studies (NEK1 and TBK1), and suggest strong cell autonomous effects in glutamatergic neurons of genetic variation on risk. The last observation is of particular interest in being in contrast with some experimental data and in apparently separating predisposition and disease modification.

Collectively these results represent an important advance in ALS genetics. The methodologies are appropriate and the results appear robust. My only suggestion is that the authors evaluate the risk associations they have detected for association with clinical outcomes (e.g. progression site of onset) to help explore the distinction between predisposition and disease course. While the data will likely only be available for a subset of samples such a targeted evaluation only of associated variants may be informative based only a proportion of the samples and would add significantly to the interest of the study.

We thank the reviewer for the revision of our manuscript, sharing our enthusiasm about our findings and the excellent suggestion to investigate the disease modifying effects of the identified genetic risk factors.

We collected detailed clinical data on age at onset, site of onset and longitudinal follow-up data on survival and respiratory status for ALS patients who were whole-genome sequenced (N = 6,095 out of a total of 6,538 sequenced cases). Within this cohort, we could study the effect of the 15 genome-wide significant SNPs, polygenic risk scores and rare variants on age at onset and disease progression (survival). We find disease modifying effects for C9orf72, UNC13A and SOD1 mutations that correspond to the expectation. Interestingly, the polygenic risk score for ALS did not have an effect on survival and most SNPs associated with ALS susceptibility did not affect age at onset or survival. This is in line with the notion that disease susceptibility and disease modification are (partly) different processes, which we agree with the reviewer is one of the interesting findings of our study.

We now discuss these findings in the revised manuscript.

Methods (page 44-45, line 967-980):

Modifier analyses

For 6,095 of the whole-genome sequenced ALS patients core clinical data was obtained including gender, site of onset (spinal or bulbar), age at onset (years), country of origin and survival defined as time from disease onset to death, 23-hour continuous non-invasive ventilation per day, or tracheostomy. Patients who were still alive were censored at the last date of follow-up.

The genetic risk factors included SNP genotypes, polygenic risk scores, C9orf72 repeat expansion status, and the number of rare coding mutations in ALS risk genes (SOD1, TARDBP, FUS, NEK1, TBK1, CFAP410) as obtained from whole-genome sequencing as described above.

For survival analyses the Cox proportional hazards mixed model from the `coxme` package in R was used modelling country of origin as a random effect. Fixed effect covariates included gender, age at onset, site of onset, GWAS stratum and PC1-5. Violation of the proportional hazards assumption for genotype on survival was assessed by inspecting Schoenfeld residuals. For the age at onset analyses, we applied linear regression of age at onset on genotype including gender, site of onset, country, GWAS stratum and PC1-5 as covariate.

Results (page 20-21, line 413-428):

Genetic modifiers of ALS disease progression. We investigated whether genetic risk factors for ALS also act as disease modifiers that affect disease onset and progression. Genotypes for the 15 genome-wide significant SNPs, polygenic risk scores and the rare variant burden for SOD1, C9orf72 (repeat expansion status), TARDBP, FUS, NEK1, TBK1 and CFAP410 were obtained for all whole-genome sequenced individuals for which the complete core clinical data (gender, age at onset, site of onset, survival, time to censoring) were available (N = 6,095). Association analyses with survival and age at onset showed that common variants had a limited effect on survival (Figure 2a) and age at onset (Figure 2b), but confirming the association between faster disease progression for the UNC13A risk allele (rs12608932, Hazard ratio [HR] = 1.10, 95% confidence interval [CI] = 1.05 - 1.15, P = 1.2 × 10^{-4}) and slower disease progression in patients with the SOD1 p.D90A mutation (rs80265967, HR = 0.35, 95%-CI = 0.16 - 0.77, P = 8.4 × 10^{-4}). This limited effect of common genetic risk factors for ALS susceptibility on disease progression is reflected in the polygenic risk score analyses where we found no effect of the full-genome PRS on survival (HR =

1.02, 95%-CI = 0.98 - 1.06, P = 0.28) or age at onset (b = 0.10, SE = 0.21, P = 0.64). The analyses of rare variants confirmed the faster disease progression in patients with the C9orf72 repeat expansion (HR = 1.45, 95%-CI = 1.28 - 1.65, $P = 1.2 \times 10^{-8}$) , with an earlier age at onset (b = -2.62, SE = 0.77, $P = 6.4 \times 10^{-4}$).

Figure

Figure 2. Genetic modifier analyses. (a) Cox proportional hazards ratios for SNPs (brown), polygenic risk scores (red) and rare variant burden in ALS risk genes (pink) on survival (months). Higher hazard ratios correspond to shorter survival. (b) Effect estimates from linear regression model on age at onset (years). Lower effect estimates correspond to a younger age at onset. The risk increasing allele for ALS corresponds to the effect allele for both survival and age at onset analyses. Error bars correspond to 95% confidence intervals.

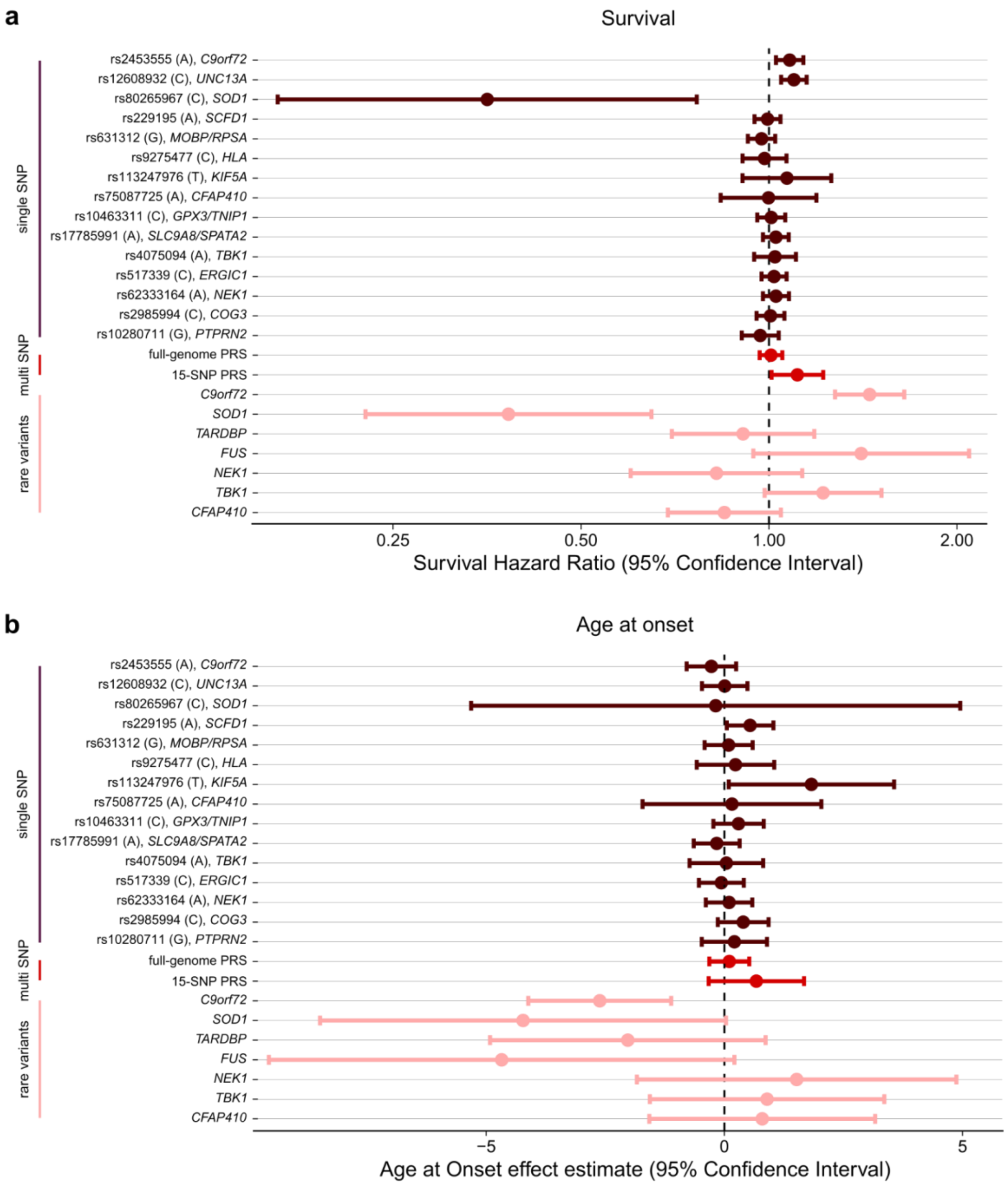
a
Survival
single SNP
multi SNP
rare variants
rs2453555 (A), C9orf72
rs12608932 (C), UNC13A
rs80265967 (C), SOD1
rs229195 (A), SCFD1
rs631312 (G), MOBP/RPSA
rs9275477 (C), HLA
rs113247976 (T), KIF5A
rs75087725 (A), CFAP410
rs10463311 (C), GPX3/TNIP1
rs17785991 (A), SLC9A8/SPATA2
rs4075094 (A), TBK1
rs517339 (C), ERGIC1
rs62333164 (A), NEK1
rs2985994 (C), COG3
rs10280711 (G), PTPRN2
full-genome PRS
15-SNP PRS
C9orf72
SOD1
TARDBP
FUS
NEK1
TBK1
CFAP410
0.25
0.50
1.00
2.00
Survival Hazard Ratio (95% Confidence Interval)
b
Age at onset
single SNP
multi SNP
rare variants
rs2453555 (A), C9orf72
rs12608932 (C), UNC13A
rs80265967 (C), SOD1
rs229195 (A), SCFD1
rs631312 (G), MOBP/RPSA
rs9275477 (C), HLA
rs113247976 (T), KIF5A
rs75087725 (A), CFAP410
rs10463311 (C), GPX3/TNIP1
rs17785991 (A), SLC9A8/SPATA2
rs4075094 (A), TBK1
rs517339 (C), ERGIC1
rs62333164 (A), NEK1
rs2985994 (C), COG3
rs10280711 (G), PTPRN2
full-genome PRS
15-SNP PRS
C9orf72
SOD1
TARDBP
FUS
NEK1
TBK1
CFAP410
−5
0
5
Age at Onset effect estimate (95% Confidence Interval)

Reviewer #2:

Remarks to the Author:

Van Rheenen et al present a trans-ancestry GWAS of ALS, which identifies 15 genome-wide significant loci (7 novel). They prioritize causal genes at 14 loci, through integrating whole-genome sequence data from ALS patients, rare expansion data and QTL data. ALS risk alleles were enriched in genes expressed in brain tissues, glutamatergic neurons and brain co-expression networks annotated to vesicle mediated transport and autophagy. Mendelian randomization analyses suggested a causal role of high cholesterol on ALS.

I commend the authors for elegantly integrating layers of genomic data and examining convergence of evidence to prioritize likely causal genes from within these 15 loci. The comparisons drawn between ALS and other neurodegenerative diseases are insightful. I don't have any major concerns but I do have a lot of minor recommendations, which I believe would substantially improve the clarity of the manuscript.

We thank the reviewer for carefully revising our manuscript, the appreciation of our work and the insightful suggestions to further improve our manuscript.

Results

As I read through the results, there were several questions I had which only became clear after reading the methods section. It would be useful if these details could be added into the results:

Thank you for this suggestion, we have moved some of the details in the Methods section to the result as suggested.

Information on the expected proportion of sporadic and familial ALS cases in the study

This is indeed an excellent point, and there is still an ongoing debate within the ALS community on the definition of "familial" and "sporadic". We would like to highlight some of the key points. Unfortunately, there is no widely adopted definition of familial (fALS) as was elegantly shown by Byrne et al. (JNNP 2012). Furthermore, a distinction between fALS and sporadic ALS (sALS) does not reflect distinct genetic architectures of fALS and sALS, rather fALS and sALS can be considered as the extremes of a spectrum. In fALS non-penetrance is rather the rule than the exception and even in patients with the pathogenic hexanucleotide repeat expansion in C9orf72 a second mutation is often found (van Blitterswijk, Human Molecular Genetics, 2012). This is in line with our finding that polygenic risk scores in ALS patients with the C9orf72 repeat expansion or variants in other ALS genes is higher compared to controls (even when the gene of interest and flanking regions are

excluded from PRS calculation). On the other hand, in apparently sporadic ALS still 6-7% of patients carry the pathogenic hexanucleotide repeat expansion (Majounie et al., Lancet Neurology, 2012 and van Rheenen et al., Neurology, 2012) again suggesting that ALS due to the C9orf72 repeat expansion is not purely monogenic. Furthermore, numerous ALS genes have been identified through both studies of familial as well as sporadic ALS (e.g. C9orf72, TBK1, NEK1, KIF5A). This illustrates that the distinction between familial and sporadic ALS is somewhat artificial and does not reflect distinct underlying genetic architectures.

For the GWAS samples, this cohort has been collected during a period of over 10 years and we unfortunately do not have a detailed family history for these individuals. We therefore prefer to describe that patients were not selected for a family history of ALS, and not make a distinction between fALS and sALS.

Main text (page 16, line 315-316):

"Patients were not selected for a family history of ALS."

Methods: (page 34, line 734-735):

"Both cases with and without a family-history for ALS and/or dementia were included. Cases were not pre-screened for specific ALS related mutations."

For the whole-genome sequencing cohort we have detailed information on the family history. As a result we now provide the numbers for patients with a known family history for ALS (page 38, line 825-828).

"The participating cohorts were not pre-screened for ALS-associated mutations and are described in the Supplementary Note. In total, 228 patients were known to have at least one first- or second-degree relative with ALS."

-the set of sequenced individuals is not independent from the GWAS

This is indeed important information and this is now described in the results section (page 17, line 346-348):

"Subsequently we investigated how rare coding variants contributed to ALS risk generating a whole-genome sequencing dataset of ALS patients (N = 6,538) and controls (N = 2,415) which is a subset of the common variant GWAS cohort".

-the sample sizes of the new European ALS cases and controls added in this study, the Asian ancestry cases and controls, and the total cross-ancestry meta-analysis

We thank the reviewer for this suggestion and we now mention the numbers of cases and controls in the first paragraph of the results section (page 16).

-some of the index SNPs were not present in the Asian samples due to low frequency (Table 1)

The reviewer is correct, 3 SNPs were absent in the Asian ancestries GWAS. This is now mentioned in the main text (page 16, line 324-325):

"The three SNPs that were not present in the Asian ancestries GWAS, were low-frequency variants (MAF 0.6 - 1.6% in European ancestries, Table 1)."

-sample sizes of the QTL datasets used would demonstrate their power

We now provide the total N for the eQTL as well as the mQTL datasets. These datasets are indeed much larger than the commonly used GTEx resource and as a result provided more power in the eQTL analyses. eQTLGen includes 31,684 individuals and MetaBrain includes cortex samples from 2,970 unique individuals. Similarly we provide these numbers for the mQTL datasets (2,082 and 522 for the blood and brain-derived datasets respectively).
Page 18, line 363-367:

"The SNP effects on gene expression were assessed through summary-based Mendelian Randomization (SMR) in blood (eQTLGen, N = 31,684) and a new brain cortex-derived expression quantitative trait locus (eQTL) dataset (MetaBrain, N = 2,970). Similarly, we analyzed methylation-QTL (mQTL) through SMR in blood (N = 2,082) and brain-derived (N = 522) mQTL datasets."

Fig 1 – this paper does a great job of dissecting the genetic architecture at the GWAS loci and it would be worth getting this information onto the plot through some sort of color legend/ symbols. Highlighting loci that also have known familial pathogenic mutations would also be a nice addition.

Thank you for appreciating the work that went into the analyses to dissect the genome-wide significant loci. Highlighting these architectures in the Manhattan plot is a great suggestion. Genes that are known to harbour highly pathogenic mutations (NEK1, C9orf72, TBK1, KIF5A, SOD1) are now highlighted in bold.

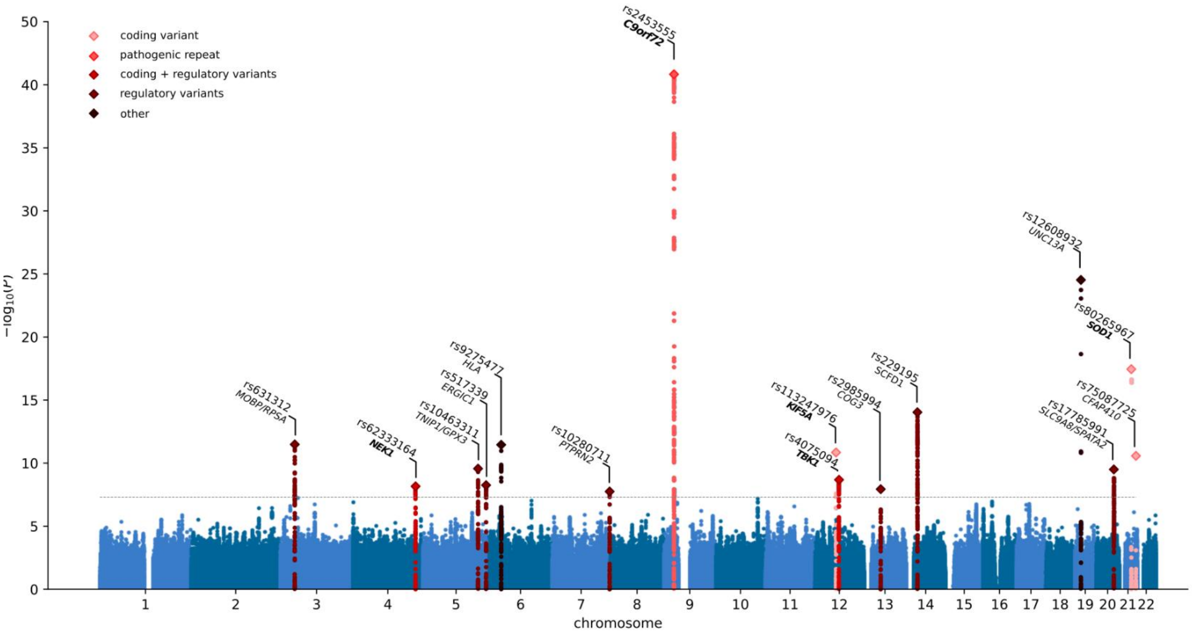

Figure 1. Manhattan plot of cross-ancestry meta-analysis. Horizontal dotted line reflects threshold for calling SNPs genome-wide significant ($P = 5 \times 10^{-8}$). Colour coding and gene labels reflect those prioritized by gene prioritization analysis. Labels in bold indicate genes with known highly pathogenic mutations for ALS.

Table 1 – including the frequencies in Asian populations would demonstrate why some of the SNPs were not present. It seems that one SNP was just not present in the Asian samples; is there a proxy SNP in high LD that could be used to perform a meta-analysis? It would be more accurate to remove the "cross-ancestry" results for those that were not present in the Asian samples.

Thank you for this useful suggestion. We have performed a proxy search in the Asian ancestries GWAS and identified rs229194 as a strong LD-proxy for rs229195. This variant does not have a

strong effect in the Asian ancestries GWAS (but has been consistently found in association studies in European ancestries). Due to the wide confidence interval, the association of rs229194 in the cross-ancestries GWAS is slightly weaker than in the European ancestries GWAS alone. We have changed the main text and Table 1 accordingly.

For the low-frequency SNPs we have left the cells for the "trans-ancestry meta-analysis" empty as suggested. Given the diversity of the Asian ancestries and the fact these are underrepresented in population databases such as gnomAD we prefer not to add a single estimate for the allele-frequency in Asian ancestries. Estimates from these reference panels might not reflect our study population (for which allele frequencies are unfortunately unavailable). The Freq column in Table 1 describes the observed frequency in our European ancestries GWAS and from this column it is already clear that the low-frequency SNPs were missing in Asian ancestries. We hope the reviewer agrees with this.

I did not see the total LDSC SNP heritability of ALS.

We now report the LDSC SNP h2 estimate of ALS to indicate that most of the inflation of test statistics was due to a polygenic signal in ALS. Page 16 line 318-320:

"We observed moderate inflation of the test statistics (λ_{GC} = 1.12, λ_{1000} = 1.003) and linkage-disequilibrium score regression yielded an intercept of 1.029 (SE = 0.0073), indicating that the majority of inflation is due to the polygenic signal in ALS (LD-score regression: = 0.028, SE = 0.003)."

The "rare variant association analysis" would be better described as "rare variant gene/ transcript burden analysis" or something along those lines, since it does not examine rare variants in the 5'UTR or conserved regions.

We agree with the reviewer on this and have changed this in the first line of this paragraph which now reads (page 17, line 342):

"Rare variant gene-based association analyses in ALS."

The calculation of the PoPS score is mentioned in the results but is not explained and none of the scores are presented in the text, only on the trackplots. I suggest removing PoPS from the results, unless the scores will be highlighted for some genes. If PoPS is included in the results, a brief explanation of how it is calculated and interpreted is needed, otherwise this should be included in the methods.

Thank you for pointing this out. Indeed, we did not want to focus too much on the PoPS prioritization analyses as this method is still under development and results are quite hard to benchmark in our study. As the reviewer kindly suggested, we have removed it from our gene prioritization analyses as it did not affect our findings.

For the 2 loci where the rare and common variant signals converge (NEK1 and TBK1), I think some elaboration is warranted in this section. What effect do the common risk increasing alleles have on gene expression and is that effect in brain, blood or both? What type of rare variant burden is found at these genes or what is the functional mechanism underlying the rare variant burden?

We agree with the reviewer that the converging evidence from common and rare variants in these loci is an interesting finding and the more details could further inform functional follow-up studies. We made the following adjustments to add clarity to the trackplots.

The legend of Supplementary Figure 5 (was 34) (NEK1) now reads:
"A genome-wide significant rare variant burden is found for the variant sets including disruptive, and disruptive + damaging variants, for both MAF thresholds."
and
"SMR showed that reduced levels of NEK1 in cortex are associated with increased risk of ALS, while this effect is opposite and weaker in blood."

The legend of supplementary Figure 12 (was 41) (TBK1) now reads:
"The lowest P-value is reached for rare variant burden models 13 and 14, meaning disruptive + damaging + missense variant, for both MAF thresholds."
and
"A risk increasing effect of TBK1 levels in blood was found both in the eQTL and mQTL analysis."
"The SMR eQTL analysis in blood predicted a risk increasing effect of higher TBK1 expression which is in line with the mQTL analysis that indicated a risk increasing effect of hypomethylation."

Similarly, for the genes in other loci implicated by eQTL or mQTLs, were the effects in blood, brain or a mix?

Following the suggestion by the reviewer, we added clarity on the tissue in which eQTL and mQTL associations were found, and the direction of eQTL effect to the Supplementary Figure legends by adding the sentences below:

Supplementary Figure 4 (was 33) (MOBP/RPSA):

"The ALS-associated SNPs within the rs631312 locus are predicted to mediate risk of ALS through expression of CCR8, RPSA, and/or MOBP in brain tissues. Additionally, the three significant CpG sites identified through SMR are predicted to regulate expression of RPSA, MOBP and SNORA6A2, all in blood."

Supplementary Figure 6 (was 35) (GPX3/TNIP1):

"Higher levels of GPX3 increased risk of ALS."

Supplementary Figure 7 (was 36) (ERGIC1):

"The mQTL analysis, however, prioritized ERGIC1 with signals in both blood and cortex, and eQTL analysis nominates ERGIC1, CREBRF and SNIP1, all from signals in blood. For all three genes, increased levels in blood were associated with higher risk of ALS."

Supplementary Figure 9 (was 38) (PTPRN2):

"Evidence is only available for PTPRN2, which has a significant eQTL SMR effect in blood, showing a protective effect of increased gene expression."

Supplementary figure 14 (was 43) (SCFD1):

"mQTL SMR identified expression-altering CpG sites for both G2E3 and SCFD1 in blood and cortex, with the strongest (brain-specific) signal for SCFD1. Increased expression of either gene was associated with decreased ALS risk. Similarly, locus-wide significant eQTL SMR effects were found for both genes in both tissues, with a stronger signal for SCFD1 than for G2E3."

A note is warranted on the HLA, since no genes were prioritized here through any of the mechanisms tested and that is only clear from viewing the trackplot.

Indeed, we did not discuss the HLA region and UNC13A since our analyses did not prioritize a single gene in these loci. Interestingly, during the revisions two new preprints came out that illustrate that the UNC13A SNPs act as splice-QTL and lead to cryptic exon inclusion only in the presence of TDP-43 dysfunction (pathological hallmark of ALS). We agree with the reviewer that these two loci should be mentioned. Page 20, line 408-412 :

For two loci no gene was prioritized through these approaches. Within the UNC13A locus (rs12608932, Supplementary Figure 15) recent studies illustrate that the genome-wide significant SNPs act as spliceQTL conditional on TDP-43 dysfunction resulting in inclusion of a cryptic exon in

UNC13A[29,30]***. Furthermore, we could not prioritize a specific gene in the HLA locus (rs9275477, Supplementary Figure 8).***

Fig 2a and 3 – it seems that both the colors and size of the squares correspond to the P value. I suggest using color only for simplicity, otherwise readers look for another layer of information that isn't present.

We thank the reviewer for pointing this out. We now use colors to present the direction of effect (genetic correlation in fig 2a and enrichment/depletion for fig 3 and supplementary figures) and size to present -log10(P-values):

Figure 3 (corresponding to figure 2 in original manuscript):

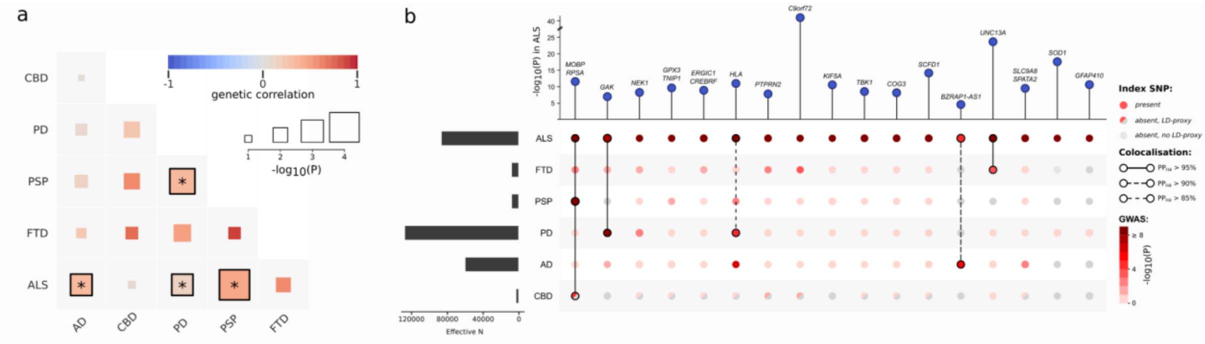

Figure 4 (corresponding to figure 3 in original manuscript):

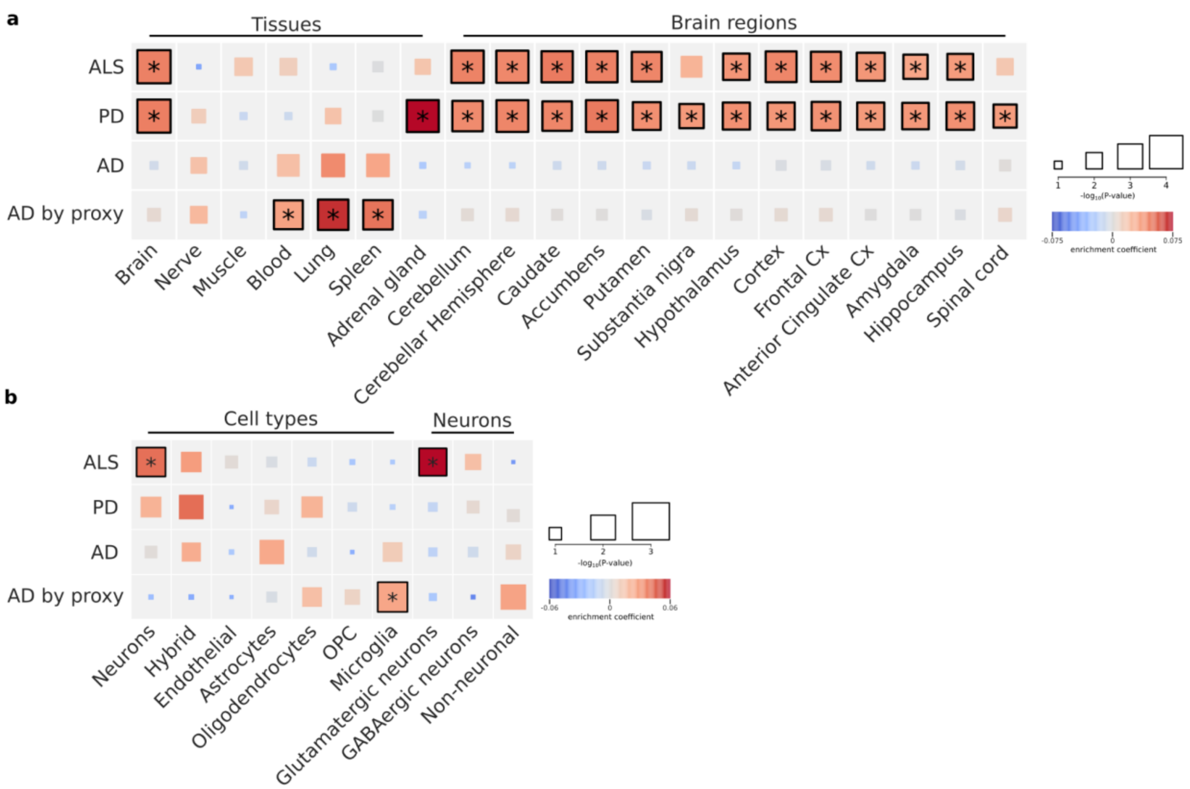

For the MAGMA tissue and single-cell enrichment analysis, how many GTEx tissues were tested? The figure only shows a limited number but there are over 50.

The reviewer is right that all tissues in GTEx v8 were tested and we thank the reviewer for pointing out that we did not clarify why we chose to visualize these tissues specifically. We decided to show only those with significant enrichment after correction for multiple testing (brain regions, adrenal gland, blood, spleen and lung) and those that are of specific interest for ALS: spinal cord, peripheral nerve, muscle. We included the non-significant tissues of interest because, exactly as the reviewer mentions in the next question, the absence of enrichment in these tissues is surprising or noteworthy. In order to provide the complete picture of our GTEx enrichment analyses, we provide a figure with all GTEx tissues in the supplement and refer to this in the main text.

Results (page 22, line 462-465):

"Whereas this pattern roughly resembles the enrichments observed in PD and psychiatric traits, it is strikingly different from that reported[31] and observed in AD where blood, lung and spleen

were mostly enriched resembling the pattern observed in multiple sclerosis which is a typical immune-mediated brain disease (Figure 4a, full results in Supplementary Figures 23 and 24)."

Supplementary figure 23

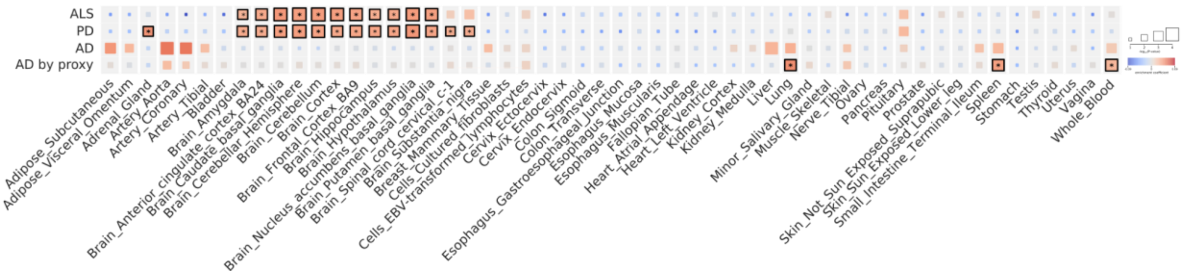

I was surprised that there was no enrichment in peripheral nervous tissue or muscle. Is that expected based on disease biology?

It is indeed interesting to see there was no enrichment in peripheral nervous tissue or muscle. The fact that we do not observe enrichment in muscle is in line with our current state of knowledge that ALS is a neurodegenerative (motor neuron) disease and indeed not a myopathy. The absence of enrichment in tibial nerve can be well explained by the fact that tibial nerve biopsies only contain axons and not the actual cell-bodies of (motor) neurons. Furthermore, tibial nerve biopsies contain sensory axons (apparently unaffected in ALS) and many non-neuronal cells including Schwann cells and a vascular tree. Therefore, transcriptomics from a peripheral nerve biopsy is probably not the optimal approach to sample the peripheral nervous system and the lower motor neuron in particular.

The absence of enrichment in muscle and peripheral nerve tissue could be considered an argument against the dying-back hypothesis (ALS originating in muscle or peripheral nerve and spreading "backwards" to the spinal cord and central nervous system).

Could this be because the enrichment test is within genes expressed in brain tissue (>50% of genes) rather than genes differentially expressed in brain versus other tissues, and therefore the brain tissue enrichment analyses may have greater power? I wonder if the same pattern of enrichment would be observed for sets of genes differentially expressed between tissues?

We thank the reviewer for this interesting thought and we performed the analyses as the reviewer suggested.

When we perform the enrichment test for differentially expressed genes in tissue of interest vs. other tissues we do observe a similar pattern of strongest enrichment in brain tissue. These however, do not pass the significance threshold after correction for multiple testing. And we can therefore not make strong claims based on this analysis.

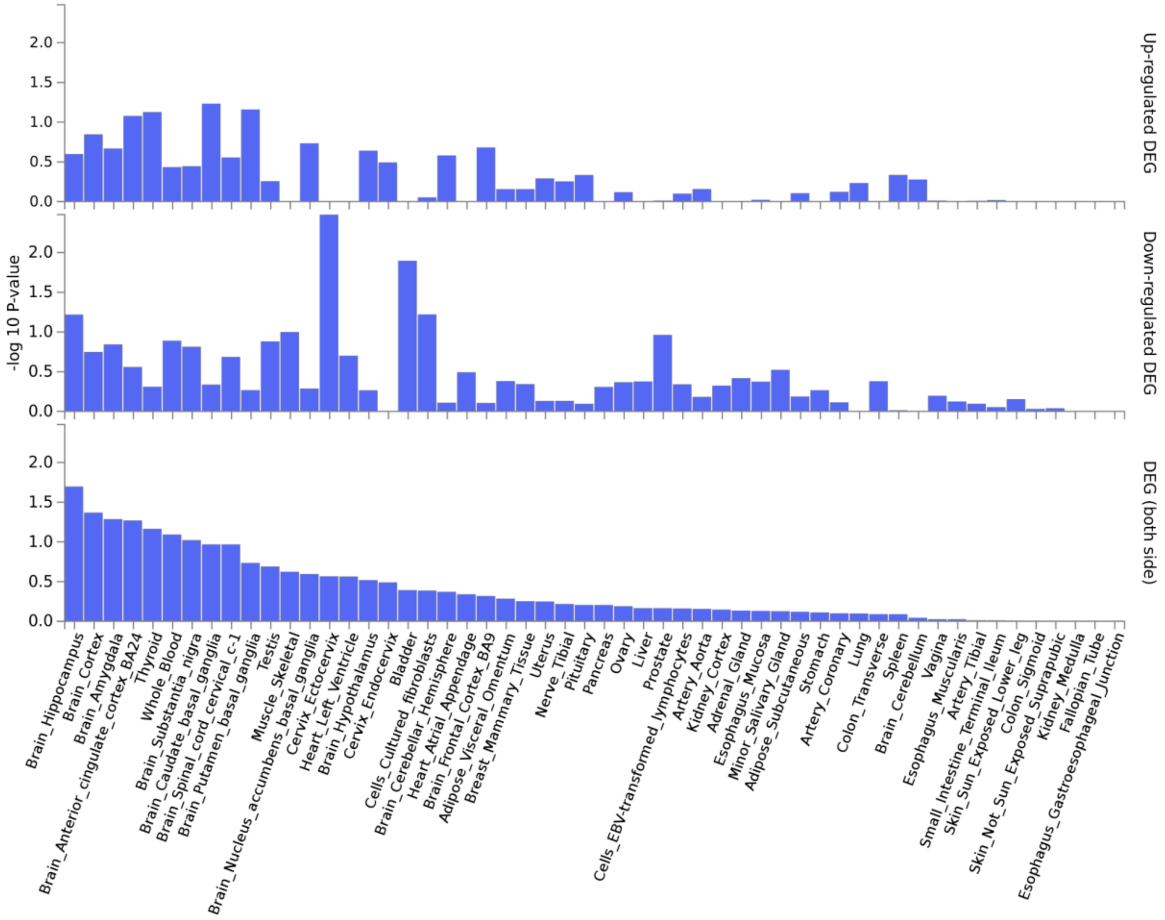

The difference between the MAGMA and DEG tissue enrichment analysis is not completely unexpected. Besides including differentially expressed genes only, the DEG enrichment analysis only includes genes that are prioritized in the MAGMA gene-based association analysis (P < 2.7e-6). Their overlap is assessed in a hypergeometrical test. This hard thresholding of differentially expressed genes and the MAGMA gene-based association analysis can result in a loss of information.

The MAGMA enrichment analysis includes expression-levels and gene-based association statistics of all genes, leveraging those that are below the exome-wide significance threshold. Their Z-scores

and expression levels are subsequently combined in a regression framework. This could explain the difference in statistical power for the hypergeometrical test in the DEG analysis compared to the MAGMA enrichment analysis.
Although we have not tested this extensively, we see a similar pattern for GWAS in MS and schizophrenia where test statistics of the MAGMA enrichment analyses are much higher than the DEG enrichment.

To further assess whether the enrichment of brain tissues in the MAGMA analysis is a function of the number of expressed genes, we ran this analysis for many more brain-related traits. This illustrates that brain enrichment is trait specific. For example AD and MS show enrichment for immune-related tissues (blood, spleen and lung), and analysis of the intracranial aneurysm (IA) GWAS shows enrichment in blood vessel tissue. Psychiatric traits show enrichment for brain tissue. These results are in line with our current state of knowledge of the biology of these traits.

Supplementary figure 24a:

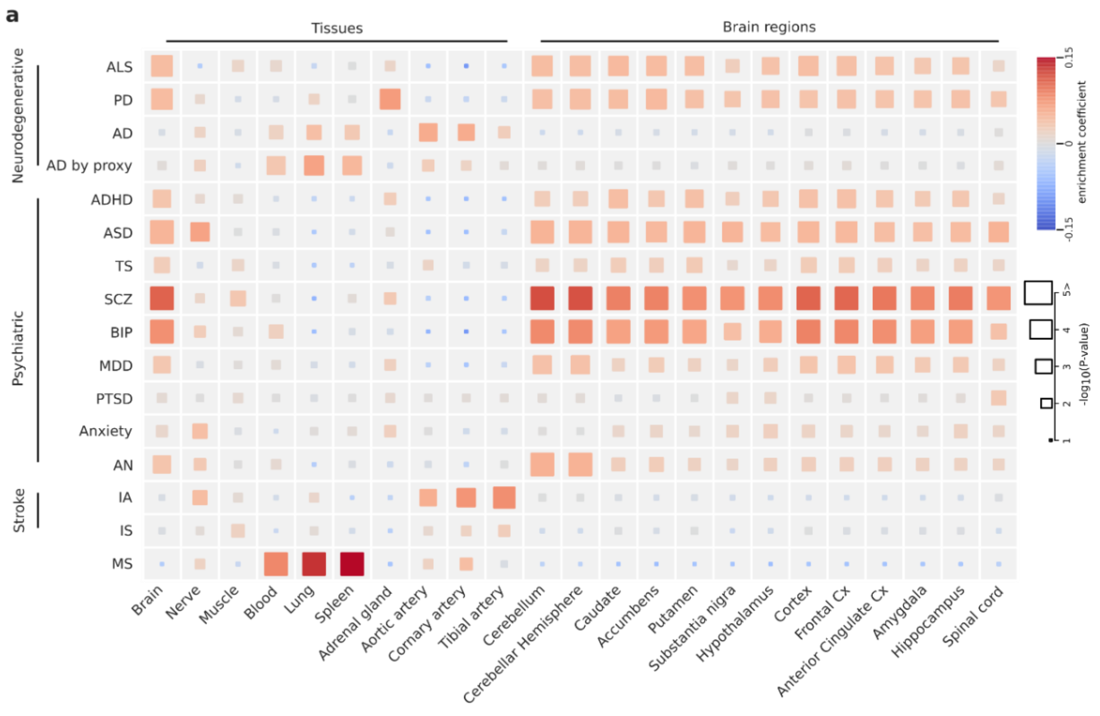

Fig 4 – the number of instruments used at each cut-off would be informative here. Are the outliers removed for these results?

We agree with the reviewer that this information can help the interpretation of the results and we have improved the legend according to the reviewer's suggestion. The legend of figure 5 (corresponds to figure 4 in original manuscript) now reads:

Figure 5. Causal inference of total cholesterol and years of schooling in ALS. (a) Mendelian randomization results for ALS and total cholesterol. Results for the five different Mendelian Randomization methods for two different P-value cut-offs for SNP instrument selection. In total 83 and 178 SNPs were used as instruments at the $P < 5 \times 10^{-8}$ and $P < 5 \times 10^{-5}$ cut-off. All methods show a consistent positive effect for an increased risk of ALS with higher total cholesterol levels. There is no evidence for reverse causality. (b) Mendelian randomization results for ALS and years of schooling. In total 306 and 681 SNPs were used as instruments at the $P < 5 \times 10^{-8}$ and $P < 5 \times 10^{-5}$ cut-off. Error-bars reflex 95% confidence intervals. Statistically significant effects that pass Bonferroni correction for multiple testing for all tested traits and MR methods are marked with an asterisk. Here, SNP outliers were not removed for instrument selection. Z = genetic instrument, MR = Mendelian Randomization, IVW = inverse-variance weighted, b_{xy} = estimated causal effect for one standard deviation increase in genetically predicted exposure.

The effect estimates did not change after removing outliers, but confidence intervals were slightly smaller after removing outliers. We prefer to present the result prior to outlier removal as we consider these outlier removal a sensitivity check that is better suited for the supplement (and it would otherwise double the number of plots in figure 5, providing nearly identical results).

Also, a quantification of the effect of unit increase in cholesterol on odds of ALS in the text would aid interpretation of the magnitude of effect.

According the the reviewer's suggestion we have now mentioned the effect estimate of the MR analysis.
Main text (page 22-23, line 505-506):

These analyses provided the strongest evidence for cholesterol levels to be causally related to ALS risk ($\beta_{WeightedMedian}$ = 0.15, SE = 0.04, $P_{WeightedMedian}$ = 3.2×10^{-4}, Figure 5a, full results in Supplementary Table 26).

But maybe we might misinterpret the reviewer's suggestion here if the suggestion is to present the predicted effect of actual cholesterol levels instead of unit increase in genetically predicted cholesterol (as estimated through MR).

In that case, we agree with the reviewer that it would be very interesting to know the odds ratio for ALS when reducing cholesterol levels by a certain mmol/L for scientific and epidemiological purposes. However, as the reviewer justly points out later, results from MR analyses should be interpreted with caution. Although MR results can be directionally concordant with clinical trial outcomes, much more methodological/empirical work is needed to investigate how accurate MR effect estimates predict generalizable effects from clinical trials (see Gill et al., Mendelian randomization for studying the effects of perturbing drug targets, Wellcome Open Res, 2021). We therefore believe presenting results as "an X reduction in total cholesterol levels leads to a Y reduction in ALS risk" does not reflect the nuances warranted in interpreting MR results.

For cholesterol and risk of ALS for example, MR analyses model a lifelong reduction in total cholesterol levels. Especially for a disease such as ALS where it is unknown whether this effect is cumulative over time (such as assumed in cardiovascular diseases) or not, MR estimates might not correspond to effects obtained in clinical trials (see also argument made by Ference et al, How to use Mendelian randomization to anticipate the results of randomized trials, Eur Heart J, 2018). Furthermore, country-specific differences in the distribution of cholesterol levels and even potential non-linear effects could further challenge the translation from the effects of genetically predicted cholesterol to actual cholesterol levels. Extrapolation of two-sample MR estimates could lead to oversimplification of results that only hold under strong assumptions.

The MR analyses as presented in our preprint have already led to patient organizations asking whether taking statins would be beneficial for ALS patients (even though effect on susceptibility can differ from effect on progression). We hope the reviewer supports our preference of not presenting the results this way.

Methods

The data description section needs an overview of how the cases were ascertained (collected specifically for case/ control studies of ALS, family studies, biobanks, postmortem studies) and how the diagnoses of ALS were made.

We agree with the reviewer on the importance of the ALS diagnosis. We now provide a summary of the diagnoses and ascertainment of ALS patients.

Methods (Page 34, line 731-739):

All ALS patients were diagnosed and ascertained through specialized MND clinics where they were diagnosed with ALS according to the (revised) El-Escorial Criteria[56] by neurologists specialized in motor neuron diseases. Whole-blood samples were drawn for DNA isolation which were specifically collected for ongoing case-control studies in ALS. Both cases with and without a family-history for ALS and/or dementia were included. Cases were not pre-screened for specific ALS related mutations. Given the late onset and relatively low life-time risk of ALS, controls were not screened for (subclinical) signs of ALS. A detailed description of the ascertainment of newly genotyped cases and controls is provided in the Supplementary Information. All participants gave written informed consent and the relevant local institutional review boards approved this study (Supplementary Information).

For a more detailed description of procedures per site we refer to the supplementary information.

Is it expected that 5% of these cases have familial ALS driven by a rare pathogenic mutation? This warrants a comment.

This is indeed an excellent point, and there is still an ongoing debate within the ALS community on the definition of "familial" and "sporadic". Unfortunately, there is no widely adopted definition of familial (fALS) as was elegantly shown by Byrne et al. (JNNP 2012). Furthermore, a distinction between fALS and sporadic ALS (sALS) does not reflect distinct genetic architectures of fALS and sALS, rather fALS and sALS can be considered as the extremes of a spectrum. In fALS non-penetrance is rather the rule than the exception and even in patients with the pathogenic hexanucleotide repeat expansion in C9orf72 a second mutation is often found (van Blitterswijk, Human Molecular Genetics, 2012). On the other hand, in apparently sporadic ALS still 6-7% of patients carry the pathogenic hexanucleotide repeat expansion (Majounie 2012 and van Rheenen, 2012) again suggesting that ALS due to the C9orf72 repeat expansion is not purely monogenic. This illustrates that the distinction between familial and sporadic ALS is somewhat artificial and does not reflect a distinct underlying genetic architectures. This is also illustrated by the fact that numerous ALS genes have been identified through both studies of familial as well as sporadic ALS (e.g. C9orf72, TBK1, NEK1, KIF5A).

We now explain this in the introduction (Page 15, line 289-291):

There is no widely accepted definition of familial or sporadic ALS(Byrne 2012) and they are likely to represent the ends of a spectrum with overlapping genetic architectures(Cirulli 2015,Freischmidt 2015,Brenner 2016,Kenna 2016,Majounie 2012,Nicolas 2018).

Furthermore, we have conducted polygenic risk score analyses comparing controls to ALS patients with rare coding variants in bonafide ALS genes and patients without variants in these genes. We found no difference in PRS between ALS patients with and without rare variants in these ALS genes, suggesting that not all variants in these genes are equally pathogenic and that the rare variants might act on a polygenic background.

Main text (page 18, line 356-360):

Polygenic risk score analyses did not illustrate a difference in polygenic risk scores in patients carrying rare variants in ALS risk genes (SOD1, C9orf72 repeat expansions, TARDBP, FUS, NEK1, TBK1, and CFAP410) compared to all ALS patients (Supplementary figure 3). Although power is limited, this is compatible with a scenario where the genetic risk of ALS in these patients is a sum of rare variants in ALS genes and other (common) genetic variation.

Supplementary figure 3.

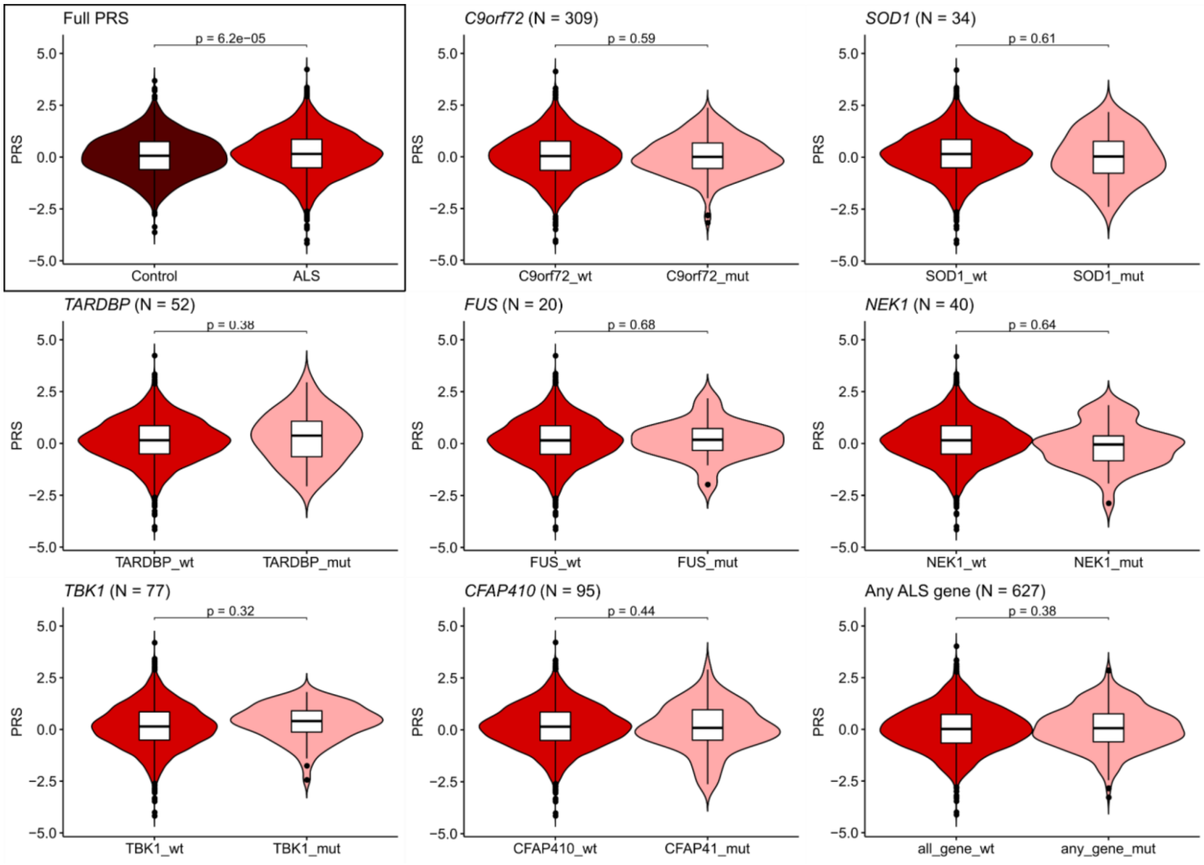

We therefore believe our manuscript provides genetic insights on ALS in general and the reader can appreciate its full spectrum shaped by risk allele frequencies and allelic effects. We hope our work will inspire future studies to further disentangle this spectrum. This warrants a large cohort of patients with extremely detailed family histories (including for ALS, but also dementia and potentially other neurodegenerative diseases to re-define "familial" disease) combined with dense genetic data on patients and their relatives. This might ultimately lead to better estimates of penetrance for specific mutations/genes that can improve genetic counselling.

It would also benefit from some brief details about the Asian ancestry studies (country of ascertainment, similarities or differences with the EUR studies).

The reviewer is correct and we have added a short description of the Asian ancestries GWAS.

Methods (page 34-35, line 742-750):

The ALS patients of Chinese ancestries were diagnosed with ALS according to the revised El-Escorial criteria[56] at Peking University Third Hospital (Beijing, China) by a neurologist specialized in ALS. Control subjects attended the Peking University Third Hospital, Peking University Sixth Hospital or Shanghai Changzheng Hospital (Shanghai) and had no medical or family history for neurological diseases. All patients and controls were of Chinese ancestry from Mainland China. The ALS patients of Japanese ancestries were ascertained by the Japanese Consortium for ALS research[57] throughout Japan. Patients were diagnosed with ALS according to the revised El-Escorial criteria[56] and had no family history for ALS. All patients were of Japanese ancestry. Control subjects were obtained from the Tohuku Medical Megabank Project[58].

I'm not sure why the Supplementary Tables are split between the pdf and excel file. It would be better to have them all together in the excel file. The tables in the excel file have no titles or legends. There's also a lot of Supplementary Figs and Tables which is a bit overwhelming. I don't think all of these are needed eg Supplementary Figs 2-17 and Supplementary Tables 4-18 present some of the same information. Could all the information be consolidated into figures? I think such figures and the Track plots would be better presented as Supplementary Data files and this would make the paper easier for the reader to navigate.

We agree with the reviewer that the supplementary data has become a lengthy document. We therefore removed the forest plots (as these data are already described in the supplementary tables). We also removed the manhattan plots and QQ-plots of the burden tests and summarized

the results in supplementary table 20. As a result, we greatly reduced the number of supplementary figures.

Some tables were too large to print on a single page and were therefore included as excel files, their titles and legends are described in the PDF (which then refers to the excel files).

We would be happy to provide the trackplots as supplementary Data files, whichever the editor prefers.

Was the WGS sample comprised of familial cases or a mixture of familial and sporadic?

The reviewer is correct that some of the whole-genome sequenced cases are known to have a positive family history for ALS. We now mention this in the methods section.

Methods (page 38, line 827-828):

"In total, 228 patients were known to have at least one first- or second-degree relative with ALS."

In the WGS QC section, I'm guessing non-Europeans were removed but this sentence seems unfinished "Outliers from the European population (HapMap3: > 10 SD on PC1-4, 1KG: > 4 SD on PC1-4)."

We thank the reviewer for pointing out this typo. It is correct that these individuals were excluded from downstream analyses.

Methods (page 40, line 878-879)

"Outliers from the European population (HapMap3: > 10 SD on PC1-4, 1KG: > 4 SD on PC1-4) were excluded from further analyses."

Where were the disruptive variant annotations obtained from?

Following the reviewer's remark, we added information about the classification of rare variant effects. These were classified by SnpEff similar to the definition described by Genoves et al, Nature Neuroscience, 2016. This line now states:

Methods (page 41, line 888-889):

"Disruptive" variants were those variants classified as frame-shift, splice-site, exon loss, stop gained, start loss and transcription ablation by SnpEff[71].

Please add the number of tests and the multiple testing correction threshold for all analyses (burden tests, QTL analyses, MAGMA enrichments, MR).

We regret that the precise number of tests for some analyses is not directly apparent. For the rare variant burden analysis, and the eQTL and mQTL analyses we applied Bonferroni correction for multiple testing correcting for the number of genes (independent tests) within the locus. This threshold is shown in the figures and (Supplementary Figures 33-47) and the overall procedure for determining the number of tests as described in the combined legend for Supplementary Figures 33-47. In addition, we now added the precise number of tests in each of the individual Figure legends of these Supplementary Figures. For example, the Supplementary Figure 33 now reads:

"Number of tests: SMR eQTL = 14 gene tissue pairs, SMR mQTL = 48 CpG tissue pairs, rare variant burden = 4 genes, repeat expansion = 69 repeat loci."

For the MAGMA enrichment analyses we used FDR correction for multiple testing because enrichment statistics do not reflect independent tests given the covariance in gene expression levels across tissues and cell-types. This was only described in the figure legend of the original manuscript, and we now also describe this in the methods section and provide the exact number of tissues and cell-types.

Methods (page 47, line 1034-1044):

Tissue and cell-type enrichment analyses were performed using the GWAS summary statistics of the European ancestries meta-analysis and FUMA[33] software v1.3.6a. FUMA performs a genic aggregation analysis of GWAS association signals to calculate gene-wise association signals using MAGMA v1.6 and subsequent tests whether tissues and cell-types are enriched for expression of these genes. For tissue enrichment analysis we used the GTEx v8 reference set. FDR-corrected p-values < 0.05 across all tissues (N = 54) were considered statistically significant. For cell-type enrichment analyses[34] we used human-derived single-cell RNA sequencing data on major brain cell-types (GSE67835 without fetal samples[111]), the Allen Brain Atlas Cell-type[112] for the human-derived major neuronal subtypes and the DropViz[113] dataset for mouse-derived brain cell-types across all brain regions. We applied FDR correction for multiple testing within each expression dataset and FDR-corrected p-values < 0.05 were considered statistically significant.

Figure 4

Figure 4. Tissue and cell-type enrichment analysis. (a) Enrichment of tissues and brain regions included in the GTEx v8 illustrates a brain-specific enrichment pattern in ALS, similar to Parkinson's disease but contrasting Alzheimer's disease. Tissues and brain regions displayed are those significantly enriched in ALS and Parkinson's disease, tissues of interest for ALS and those previously reported in Alzheimer's disease. (b) Cell-type enrichment analyses indicate neuron-specific enrichment for glutamatergic neurons. No enrichment was found for microglia or other non-neuronal cell-types, contrasting the pattern observed in Alzheimer's disease. Statistically significant enrichments after correction for multiple testing over all tissues (N = 54), cell-types (N = 7) and neurons (N = 3) with a false discovery rate (FDR) < 0.05 are marked with an asterisk. ALS = amyotrophic lateral sclerosis, PD = Parkinson's disease, AD = Alzheimer's disease, Cx = cortex, OPC = oligodendrocyte progenitor cells.

Reviewer #3:
Remarks to the Author:
In their manuscript entitled "Common and rare variant association analyses in Amyotrophic Lateral Sclerosis identify 15 risk loci with distinct genetic architectures and neuron-specific biology" by van Rheenen, van der Spek, Bakker et al, the authors describe a comprehensive cross-ancestry meta-analyses of GWAS data from 29,612 ALS patients and 122,656 controls, combined with 8,953 whole-genome sequenced individuals (6,538 ALS patients, 2,415 controls) and a large cortex-derived eQTL dataset. First, the authors identified 15 risk loci significantly associated with ALS; ALS associated signals indicate a critical role in vesicle mediated transport and autophagy. Second, the authors describe distinct enrichment patterns across brain regions and cell-types, with evidence supporting cell-autonomous disease initiation in glutamatergic neurons. Third, and perhaps most surprisingly, Mendelian randomization analyses indicated a causal role for high cholesterol levels. Revisions to independently test the predictions described in this manuscript, either through analysis of imputed and convergent expression patterns of ALS risk genes, validation in electronic health records, and/or functional validation of key molecular risk genes would improve the impact of this systematic and thorough analyses. With what I hope to be straightforward new analyses, I expect that this report will be suitable for Nature Genetics.

We thank the reviewer for the careful revision of our manuscript and are grateful for the positive feedback on our work which we also hope is suitable for Nature Genetics. The reviewer has suggested some very interesting additional analyses which we believe have improved our manuscript considerably.

Major concerns:

1. Genomic analyses. The authors identify 15 common risk loci for ALS (8 previously reported), independently identify NEK as both a common and rare variant, and associate coding variants with 3 of the 15 common risk loci. They further conduct multi-omic analyses to integrate GWAS and eQTL datasets, and also explore enrichments across tissues, cell types, and biological pathways. This is a rigorous analysis and I have only minor suggestions:
a. Do the authors specifically include spinal cord tissue in the eQTL analyses and motor neuron signatures in the cell type analyses?

We are happy the reviewer shows their appreciation for the eQTL analyses. The reviewer is right that spinal cord samples were included in the MetaBrain eQTL datasets. This eQTL dataset, however, only includes 108 individuals compared to 2,970 for the brain cortex eQTL dataset. As a result, only a small number of eQTL could be detected (811 replicated eQTL in spinal cord compared to 11,803

in brain cortex). Furthermore, virtually all eQTL detected in spinal cord were found in brain cortex (98.93%), so we do not expect to find spinal cord-specific SMR results. Indeed, due to the limited power in the spinal cord eQTL dataset we did not find any genome-wide significant SMR results (not even C9orf72) when using this dataset. Therefore, we decided to not include this in the manuscript and hope the reviewer supports this.

Spinal cord transcriptome data was used for the MAGMA enrichment analysis. This showed that there was no significant enrichment of spinal cord tissue after correction for multiple testing. We show this in figure 4 of the revised manuscript. The lack of (detectable) enrichment in spinal cord could be a matter of power, but this can also be explained by the relatively low number of motor neurons in full spinal cord samples. There are no spinal cord scRNA-seq datasets available for the FUMA single cell enrichment analysis and we could therefore not test cell-type enrichment in spinal cord.

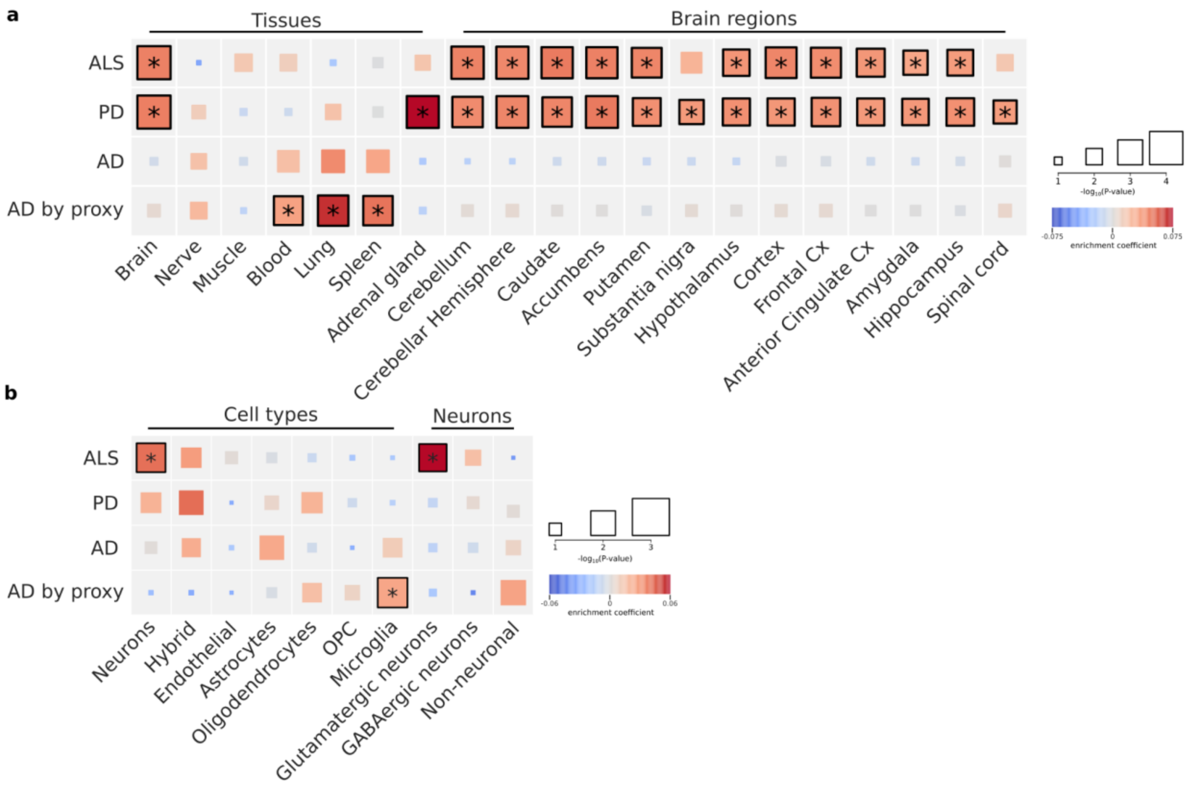

b. The analyses integrated eQTL information with GWAS by colocalization and SMR to estimate the association between intermediate gene expression levels and phenotypes. Are the results consistent with an alternative method (e.g. PrediXcan, TWAS)?

We thank the reviewer for this excellent question. As the reviewer suggested we applied PrediXcan and TWAS to compare these to SMR results. Results between these three methods are extremely consistent for whole-blood as well as in brain cortex eQTL datasets (see figure).

Important to note is that these analyses could only be performed in the smaller GTEx eQTL resource. Whereas S-PrediXcan and TWAS have the benefit of further reducing noise by elastic net regression, this warrants re-analysis of the eQTL datasets and access to individual level genotype and expression data. This means that these methods cannot be applied to eQTLGen and MetaBrain. Access to individual level data is restricted (and for eQTLGen no single consortium member has access to all datasets). Furthermore, from a statistical perspective both eQTLGen and MetaBrain are a result of meta-analyses across datasets. To our knowledge, the elastic net regression to calculate SNP weights for TWAS and PrediXcan have not been applied and validated yet for such a meta-analysis framework (with considerable batch effects).

Given that the results are highly consistent and SMR could be applied to the most powerful eQTL resources, we presented the SMR results as the main eQTL gene prioritization analysis. We now describe our reasoning with the comparison between SMR, PrediXcan and TWAS in the revised manuscript.

Methods (page 42, line 927-930):

We chose to apply SMR because this method yielded very similar results when compared to S-PrediXcan[83] and TWAS[84] (Supplementary Figure 28) when applied using GTEx v7 eQTL and it can be applied to the largest relevant eQTL datasets (MetaBrain and eQTLGen) without access to individual level genotype and gene expression data.

Supplementary figure 28 (page 51, supplementary material):

Supplementary figure 28. SMR, S-PrediXcan and TWAS comparison. Comparison between eQTL gene prioritization analyses using SMR, S-PrediXcan and TWAS with eQTL estimates from GTEx v7 whole blood and brain samples. eQTL-based gene statistics across all three methods were strongly correlated.

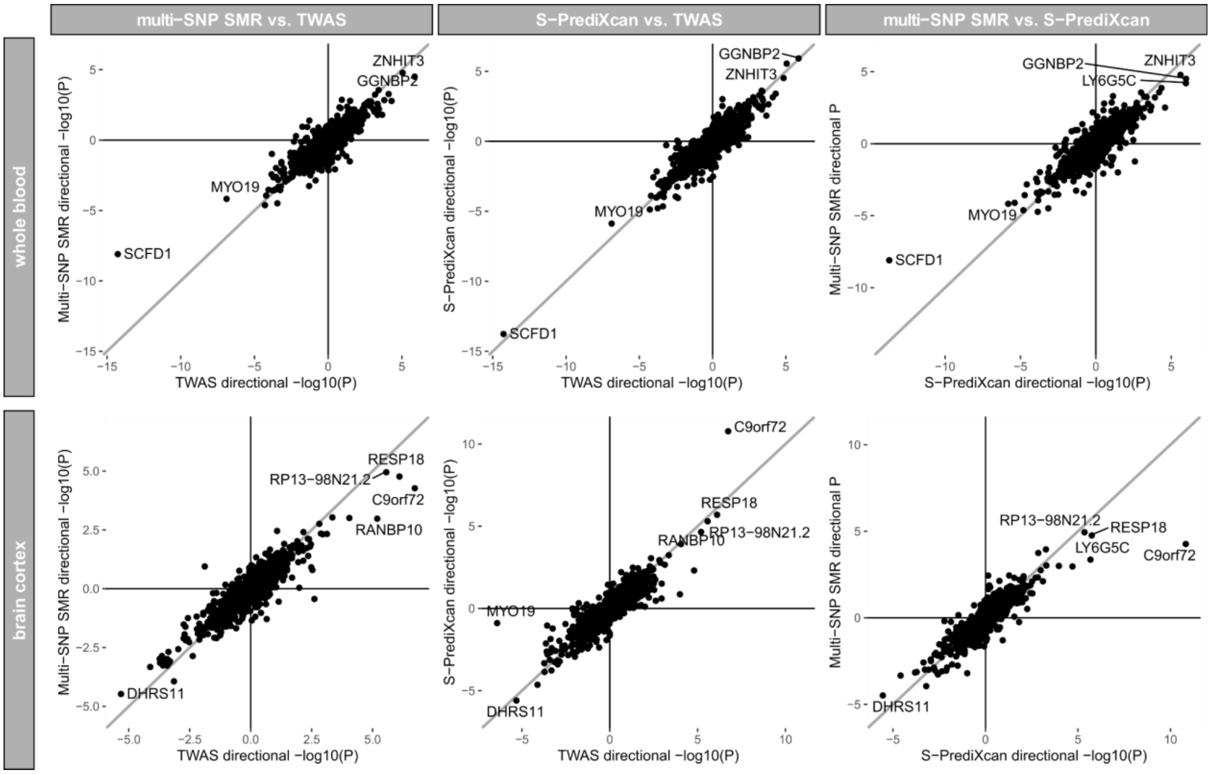

c. Are risk variants enriched for any regulatory motifs? To what extent are risk variants and genes in regions of evolutionary constraint?

We thank the reviewer for this question. Indeed we have performed enrichment analyses for regulatory motifs. This shows that conserved regions of the genome are enriched for ALS risk increasing genetic variation. These include both coding regions of the genome, but also 5' UTRs. We describe this on page 17, line 342-346 of the revised manuscript:

"To assess a general pattern of underlying architectures that link associated SNPs to causal genes, we first tested for annotation specific enrichment using stratified linkage disequilibrium score regression (LDSC). This revealed that 5' UTR regions as well as coding regions in the genome and those annotated as conserved were most enriched for ALS-associated SNPs (Supplementary Figure 2)."

Supplementary figure 2 (page 9 of supplementary information):

Supplementary figure 2: Annotation specific enrichment. Enrichment of SNP-based heritability was calculated with LD-score regression. Grey dashed line represents no enrichment

(enrichment = 1). Error bars denote standard error of enrichment estimate. Due to the regression framework in LDSC, enrichment estimates can be < 0 (with large standard errors).

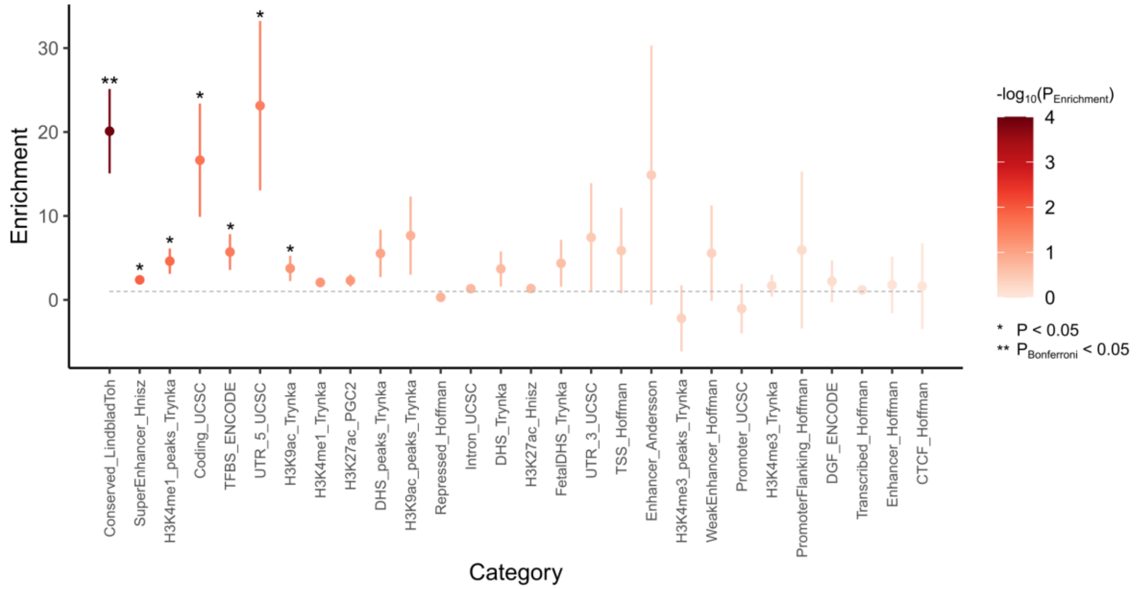

d. Is polygenic risk burden altered in rare variant carriers?

This is an intriguing question which we followed up. These analyses yielded interesting results. We constructed polygenic risk scores (PRS) for all individuals in our whole-genome sequencing cohort and compared controls to patients with rare variants in bonafide ALS genes and patients without these variants. To make sure the PRS did not capture rare variant signals in these genes we excluded the gene of interest and the 1MB flanking regions from the PRS. SNP weights were calculated using summary-BayesR which was shown to outperform p-value thresholding and clumping and was among the highest ranking PRS methods in terms of out-of-sample prediction accuracy in real-world GWAS data in psychiatric traits (Ni et al, MedRxiv, 2021, https://doi.org/10.1101/2020.09.10.20192310).

This showed that overall the PRS is higher in ALS patients compared to controls, but we observe no significant difference between ALS patients with and without rare variants in known ALS risk genes.

Methods (page 44, line 957-966):

Polygenic risk scores were constructed based on the 15 lead SNPs of the genome-wide significant loci (15-SNP PRS) or a full genome-wide model (full-genome PRS). For the 15 SNP PRS the SNP weights were defined as the meta-analysed effect estimates. We used the summary-BayesR framework from the Genome-wide Complex Trait Bayesian analysis (GCTB) toolkit[89,90] to obtain SNP weights for the full-genome PRS based on the European ancestries meta-analysis excluding stratum 6. We used the default model parameters and the pre-calculated sparse LD matrix of imputed HapMap3 SNPs in 50,000 random individuals included in the UK Biobank of European ancestries. Summary-BayesR SNP effects were plotted against marginal SNP effects to rule out potential biased estimates due to non-convergence of the MCMC algorithm. Finally, the polygenic risk scores for all individuals in stratum 6 were calculated using the --score function in PLINK and normalized to zero mean and unit variance.

Results (page 18, line 356-360):

"Polygenic risk score analyses did not illustrate a difference in polygenic risk scores in patients carrying rare variants in ALS risk genes (SOD1, C9orf72 repeat expansion, TARDBP, FUS, NEK1, TBK1, and CFAP410) compared to all ALS patients (Supplementary Figure 3). Although power is limited, this is compatible with a scenario where the genetic risk of ALS in these patients is a sum of rare variants in ALS genes and other (common) genetic variation."

Supplementary Figure 3 (page 12 of supplementary information):

Supplementary figure 3. Polygenic risk scores vs. rare variants. Distribution of polygenic risk scores in controls, and ALS patients with or without one or more rare variants in ALS risk genes. For SOD1, TARDBP, FUS, NEK1, TBK1, CFAP410 rare variants were included according to the model that yielded the strongest association in the rare variant burden association analyses. For C9orf72, patients with the pathogenic hexanucleotide repeat expansion were compared to those without the expansion. The "any ALS gene" combines all ALS patients together with a rare variant in any of the ALS risk genes. SNPs in ALS genes +/- 1MB were excluded from the PRS calculation to exclude any SNPs that tag rare variants. There is no statistically significant difference in PRS between ALS patients with and without rare variants in ALS risk genes.

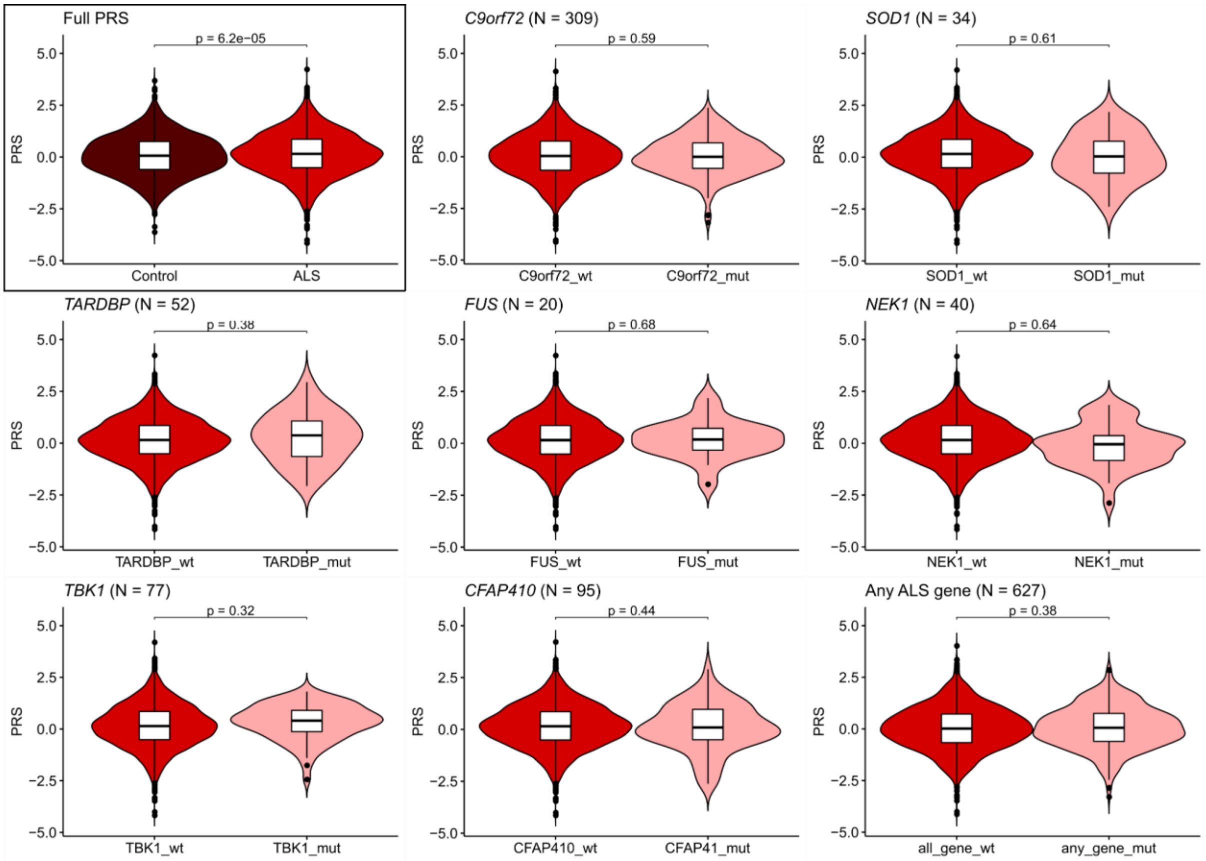

Do clinical characteristics (age of onset, rate of disease progression) differentiate rare variant carriers?

It is an interesting idea to expand our rare variant analyses with additional clinical characteristics. We provide this analysis together with the response to question 3a (see below).

2. Disease-specificity: The authors did a great job exploring the extent of genetic overlap with co-morbid disorders (FTD, AD, PD), particularly in applying existing bulk and scRNAseq datasets to discuss the expression patterns of ALS-specific and shared disease genes between neural cell types. Could this be expanded to consider expression across human development?

We are happy the reviewer appreciates our effort to disentangle similarities and differences between neurodegenerative diseases. As the reviewer suggested we have included more brain-related diseases and this indeed puts the genetic overlap between neurodegenerative diseases in a broader context.

To calculate genetic correlations we have included neurodegenerative diseases, psychiatric traits, cardiovasculair traits and MS as an autoimmune disease of the central nervous system. This shows that neurodegenerative diseases from a nicely correlated cluster.

We now present this at the beginning of the paragraph as it explains to the reader why we choose to zoom in on shared genetic risk factors between neurodegenerative diseases.

Results (page 20-21, line 429-433):

"To investigate the pleiotropic properties of ALS-associated variants and shared genetic risk with other brain diseases, we estimated genetic correlations between neurodegenerative diseases, psychiatric traits, cerebrovascular diseases and multiple sclerosis (Supplementary Figure 20). This showed strong genetic correlations among neurodegenerative diseases."

Supplementary figure 20 (page 43 of supplementary information)

Supplementary figure 20. Genetic correlations between brain diseases. Correlation matrix for genetic correlation estimates obtained from bivariate LD score regression. Strongest clusters appear between neurodegenerative diseases and within the psychiatric traits. ALS = amyotrophic lateral sclerosis, FTD = frontotemporal dementia, PSP progressive supranuclear palsy, PD = Parkinson's disease, CBD = corticobasal degeneration, AD = (clinically diagnosed) Alzheimer's disease, MS = multiple sclerosis, IS = ischemic stroke (any), ICH = intracerebral hemorrhage, IA = intracranial aneurysm (any), AN = anorexia nervosa, OCD = obsessive compulsive disorder, Anxiety = anxiety disorder (score), PTSD = post-traumatic stress disorder, MDD = major depressive disorder, BIP = bipolar disorder, SCZ = schizophrenia, TS = Tourette's syndrome, ASD = autism spectrum disorder, ADHD = attention-deficit hyperactivity disorder.

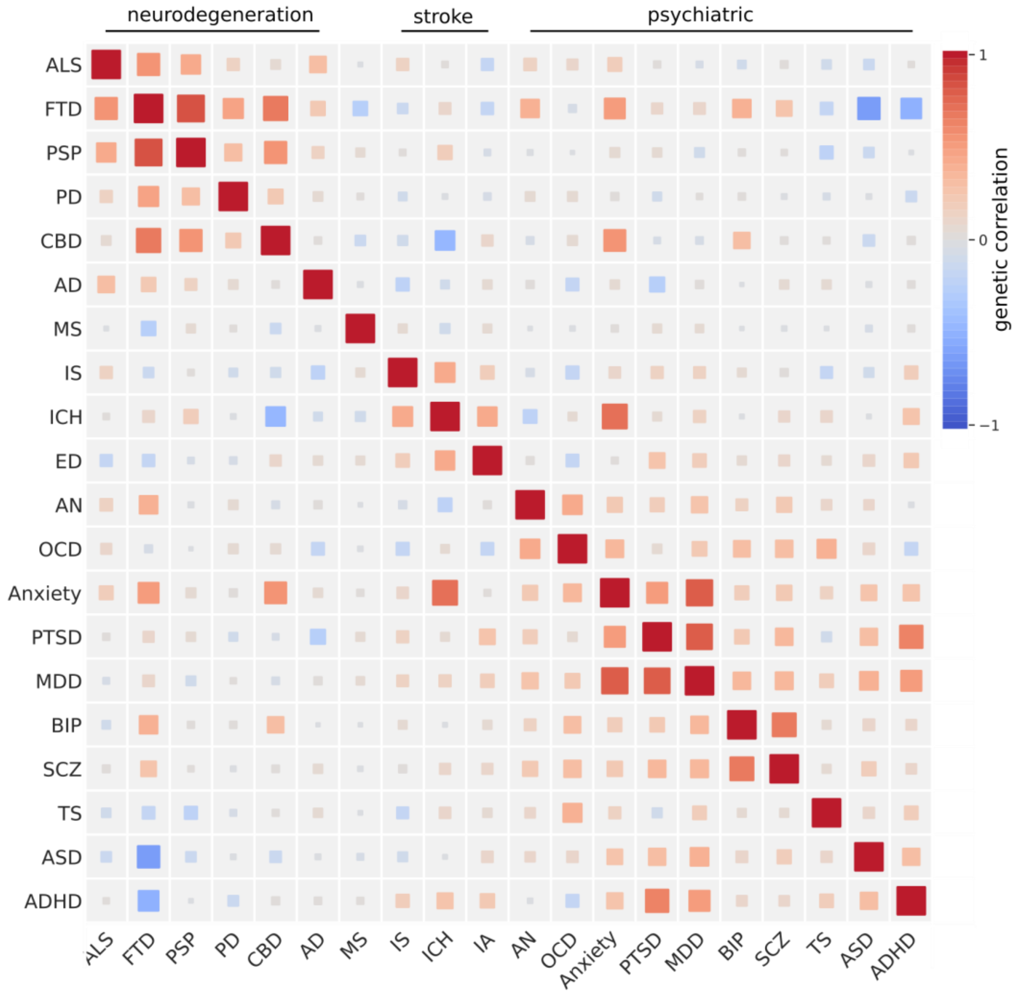

a. The association between GWAS target gene expression and disease could be explored using post-mortem datasets (psychiatric, neurodegenerative and non-brain related) to further explore and validate the specificity of the results.)

Similar to the previous question, this could again be a great suggestion to put our enrichment analyses in a broader context. To this end, we applied the tissue and cell-type enrichment analyses to neurodegenerative diseases (ALS, AD and PD), psychiatric diseases (with a wide range in age at onset), cerebrovascular diseases (ischemic stroke and intracranial aneurysms) and auto-immune disease of the CNS (multiple sclerosis).

Indeed, this shows that the brain enrichment pattern in ALS is not a random finding but specific for primary diseases of the central nervous system. We observe this pattern PD but also psychiatric traits. The enrichment pattern observed in AD (blood, spleen, lung and microglia) which we interpret as a pattern of immune-mediated tissues is also observed in a classic immune-related brain disease as multiple sclerosis. Furthermore, we observe an expected enrichment for arteries in intracranial aneurysms. We now describe these findings in the main text of the revised manuscript.

Results (page 22, line 462-465):

"We observed a significant enrichment in genes expressed in brain tissues across multiple brain regions, but not peripheral nervous tissue or muscle. Whereas this pattern roughly resembles the enrichments observed in PD and psychiatric traits, it is strikingly different from that reported[31] and observed in AD where blood, lung and spleen were mostly enriched resembling the pattern observed in multiple sclerosis which is a typical immune-mediated brain disease (Figure 4a, full results in Supplementary Figures 23 and 24a)."

Supplementary Figure 24a (page 48 of supplementary information)

Supplementary figure 24. Tissue and cell-type enrichment analyses for all brain diseases. Tissue (a) and cell-type (b) enrichment for all included brain diseases. Only brain diseases with significant MAGMA associations were suitable for tissue and cell-type enrichment analyses. Due to the large number of significant genes in the MAGMA analyses for schizophrenia, bipolar disorder and multiple sclerosis the enrichment p-values were truncated at $P < 1.0 \times 10^{-5}$. ALS = amyotrophic lateral sclerosis, PD = Parkinson's disease, AD = Alzheimer's disease, ADHD = attention-deficit hyperactivity disorder, ASD = autism spectrum disorder, TS = Tourette's syndrome, SCZ = schizophrenia, BIP = bipolar disorder, MDD = major depressive disorder, PTSD = post-traumatic stress syndrome, Anxiety = anxiety disorder (score), AN = anorexia nervosa, IA intracranial aneurysm (any), IS = ischemic stroke, MS = multiple sclerosis.

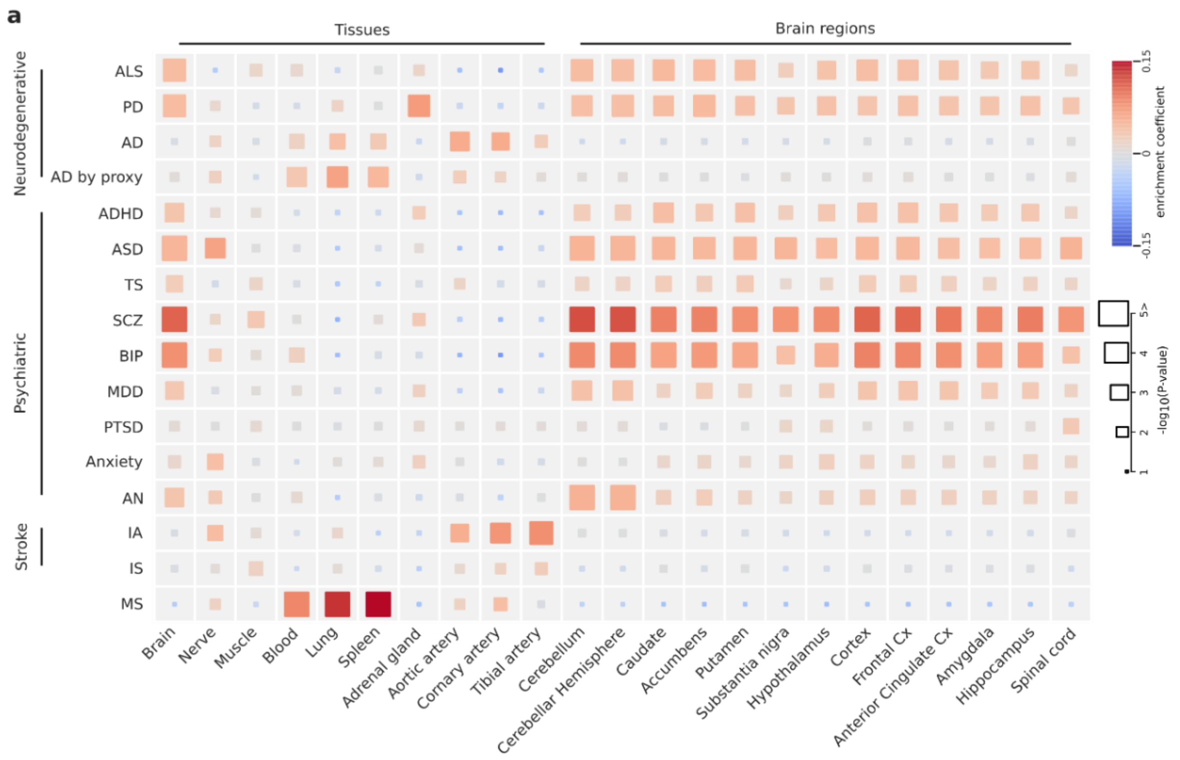

As for the cell-type enrichment, the glutamatergic neuron-specific enrichment seems specific to ALS. In psychiatric diseases most enrichment is seen in GABAergic neurons (major depressive disorder and bipolar disorder) and again non-neuronal cells in AD and MS.

Results (page 22, line 468-471):

We subsequently queried single-cell RNA sequencing datasets of human-derived brain samples to further specify brain-specific enriched cell-types using the cell-type analysis module in FUMA[34]. This showed significant enrichment for neurons but not microglia or astrocytes (Figure 4b). Further subtyping of these neurons illustrated that genes expressed in glutamatergic neurons were mostly enriched for genes within the ALS-associated risk loci. Again, this contrasted AD which showed specific enrichment of microglia similar to MS (Supplementary Figure 24b).

Supplementary Figure 24b (page 48 of supplementary information):

Supplementary figure 24. Tissue and cell-type enrichment analyses for all brain diseases. Tissue (a) and cell-type (b) enrichment for all included brain diseases. Only brain diseases with significant MAGMA associations were suitable for tissue and cell-type enrichment analyses. Due to the large number of significant genes in the MAGMA analyses for schizophrenia, bipolar disorder and multiple sclerosis the enrichment p-values were truncated at $P < 1.0 \times 10^{-5}$. ALS = amyotrophic lateral sclerosis, PD = Parkinson's disease, AD = Alzheimer's disease, ADHD = attention-deficit hyperactivity disorder, ASD = autism spectrum disorder, TS = Tourette's syndrome, SCZ = schizophrenia, BIP = bipolar disorder, MDD = major depressive disorder, PTSD = post-traumatic stress syndrome, Anxiety = anxiety disorder (score), AN = anorexia nervosa, IA intracranial aneurysm (any), IS = ischemic stroke, MS = multiple sclerosis.

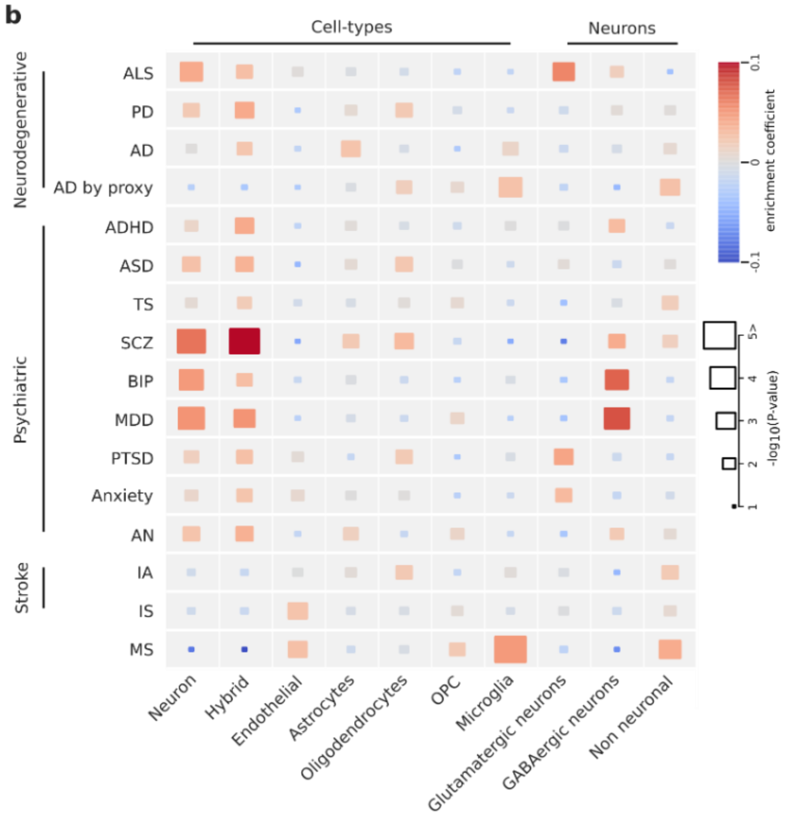

Together, this underlines the specificity of our claim that ALS is a primary brain disease with specific enrichment for glutamatergic neurons.

We thank the reviewer for these useful comments.

3. Replication: If the authors used all available ALS GWAS datasets in their meta-analysis, can the issue of replication be addressed using PRS leave-one-out analyses to demonstrate a higher burden of common risk variants associated with ALS cases, compared with controls, across all the cohorts.

The reviewer is correct that we have collected all ALS GWAS datasets (individual level genotype data for most and summary statistics for Asian ancestries ALS GWAS). As suggested, we have assessed the polygenic risk scores in a leave-one-out framework where we calculated the PRS based on the meta-analysis leaving the target stratum out and then assessed its association with case-control status in the SAIGE logistic regression framework (to accurately account for population stratification within the stratum).

The 15-SNP PRS that could be considered cross-validation of the genome-wide significant SNPs was strongly associated with case-control status in each stratum. This is in line with the forest plots in the supplement that indicate that association signals were consistent across strata and were not driven by single stratum outliers.

The full-genome PRS was also strongly associated with case-control status in each stratum, except for stratum 2. This can be explained by the fact that this is the smallest stratum. Combined with the fact that the SNPs in the full-genome PRS only explain a small proportion of the h2 in ALS (h^2 = 0.028 as calculated by LD score regression), this non-significant finding of the full-genome PRS in one of the strata can be expected.

	Full-genome PRS			***15-SNP PRS***		
Stratum	***BETA***	***SE***	***p.value***	***BETA***	***SE***	***p.value***
s1	*0.862*	*0.108*	*1.89E-15*	*0.619*	*0.102*	*1.33E-09*
s2	*0.189*	*0.122*	*0.121*	*0.712*	*0.159*	*7.05E-06*
s3	*0.978*	*0.144*	*1.15E-11*	*1.812*	*0.173*	*1.19E-25*
s4	*1.497*	*0.087*	*2.22E-66*	*1.559*	*0.157*	*2.94E-23*

s6	*0.860*	*0.048*	*5.14E-73*	*1.190*	*0.066*	*1.33E-72*
s7	*1.228*	*0.084*	*1.68E-48*	*1.071*	*0.125*	*8.24E-18*

Alternately, is it possible to conduct ALS-by-proxy in other existing datasets (eg. 23andme)?

We have contacted both the UK Biobank and 23andMe. In the UK Biobank ALS is recorded, but currently it includes only 423 cases mostly diagnosed during hospital admission and therefore already included in the UK MNDA biobank which is part of our study. In the UK biobank a family history for ALS (affected parents) is not collected and therefore the ALS-by-proxy approach is unfortunately not possible. The 23andMe team responded that ALS is neither recorded as a diagnosis, nor a family history for ALS. An unfortunate downside of studying a relatively rare disease is that we cannot profit from well-powered population based cohorts.

We acknowledge the value of replication of GWAS loci in an independent cohort. Therefore, we show that in our cross-ancestry design all the genome-wide significant SNPs all have consistent directions of effect in the independent Asian ancestries ALS GWAS. Collecting a new well-powered cohort to serve as independent replication for the genome-wide significant loci of the cross-ancestry meta-analysis would take a non-trivial amount of time for a disease as ALS which is relatively rare and which cannot be newly diagnosed from questionnaires used in population-based cohorts. For example, 10,000 ALS cases and 30,000 controls would be needed to achieve 80% power for replication of the PTPRN2 locus at alpha = 0.05 in a scenario where we do not account for multiple testing correction (and do not suffer from the winner's curse).

We hope that the reviewer and editors agree that the novel insights our GWAS analyses provide collectively on the biology of ALS, beyond single SNP associations, are indeed an important contribution to the field.

a. It would be interesting to evaluate the burden of ALS variants and mutations as a function of other clinical measures in HER.

We would like to thank the reviewer for another interesting suggestion. We collected detailed clinical data on age at onset, site of onset and longitudinal follow-up data on survival and respiratory status for ALS patients that were whole-genome sequenced (N = 6,095 out of a total of 6,538

sequenced cases). Within this cohort, we could study the effect of the 15 genome-wide significant SNPs, polygenic risk scores and rare variants on age at onset and disease progression (survival). We find disease modifying effects for the C9orf72, UNC13A, SOD1. Interestingly, the polygenic risk score for ALS did not have an effect on survival and most SNPs associated with ALS susceptibility did not affect age at onset nor survival. This is in line with the notion that disease susceptibility and disease modification are (partly) different processes.

Methods (page 44-45, line 967-980):

Modifier analyses

For 6,095 of the whole-genome sequenced ALS patients core clinical data was obtained including gender, site of onset (spinal or bulbar), age at onset (years), country of origin and survival defined as time from disease onset to death, 23-hour continuous non-invasive ventilation per day, or tracheostomy. Patients who were still alive were censored at the last date of follow-up.

The genetic risk factors included SNP genotypes, polygenic risk scores, C9orf72 repeat expansion status, and the number of rare coding mutations in ALS risk genes (SOD1, TARDBP, FUS, NEK1, TBK1, CFAP410) as obtained from whole-genome sequencing as described above.

For survival analyses the Cox proportional hazards mixed model from the `coxme` package in R was used modelling country of origin as a random effect. Fixed effect covariates included gender, age at onset, site of onset, GWAS stratum and PC1-5. Violation of the proportional hazards assumption for genotype on survival was assessed by inspecting Schoenfeld residuals. For the age at onset analyses, we applied linear regression of age at onset on genotype including gender, site of onset, country, GWAS stratum and PC1-5 as covariate.

Results (page 20-21, line 413-428):

Genetic modifiers of ALS disease progression. We investigated whether genetic risk factors for ALS also act as disease modifiers that affect disease onset and progression. Genotypes for the 15 genome-wide significant SNPs, polygenic risk scores and the rare variant burden for SOD1, C9orf72 (repeat expansion status), TARDBP, FUS, NEK1, TBK1 and CFAP410 were obtained for all whole-genome sequenced individuals for which the complete core clinical data (gender, age at onset, site of onset, survival, time to censoring) were available (N = 6,095). Association analyses with survival and age at onset showed that common variants had a limited effect on survival (Figure 2a) and age at onset (Figure 2b), but confirming the association between faster disease

progression for the UNC13A risk allele (rs12608932, Hazard ratio [HR] = 1.10, 95% confidence interval [CI] = 1.05 - 1.15, P = 1.2 × 10^{-4}) and slower disease progression in patients with the SOD1 p.D90A mutation (rs80265967, HR = 0.35, 95%-CI = 0.16 - 0.77, P = 8.4 × 10^{-4}). This limited effect of common genetic risk factors for ALS susceptibility on disease progression is reflected in the polygenic risk score analyses where we found no effect of the full-genome PRS on survival (HR = 1.02, 95%-CI = 0.98 - 1.06, P = 0.28) or age at onset (b = 0.10, SE = 0.21, P = 0.64). The analyses of rare variants confirmed the faster disease progression in patients with the C9orf72 repeat expansion (HR = 1.45, 95%-CI = 1.28 - 1.65, P = 1.2 × 10^{-8}) , with an earlier age at onset (b = -2.62, SE = 0.77, P = 6.4 × 10^{-4}).

Figure 2

Figure 2. Genetic modifier analyses. (a) Cox proportional hazards ratios for SNPs (brown), polygenic risk scores (red) and rare variant burden in ALS risk genes (pink) on survival (months). Higher hazard ratios correspond to shorter survival. (b) Effect estimates from linear regression model on age at onset (years). Lower effect estimates correspond to a younger age at onset. The risk increasing allele for ALS corresponds to the effect allele for both survival and age at onset analyses. Error bars correspond to 95% confidence intervals.

[figure on next page]

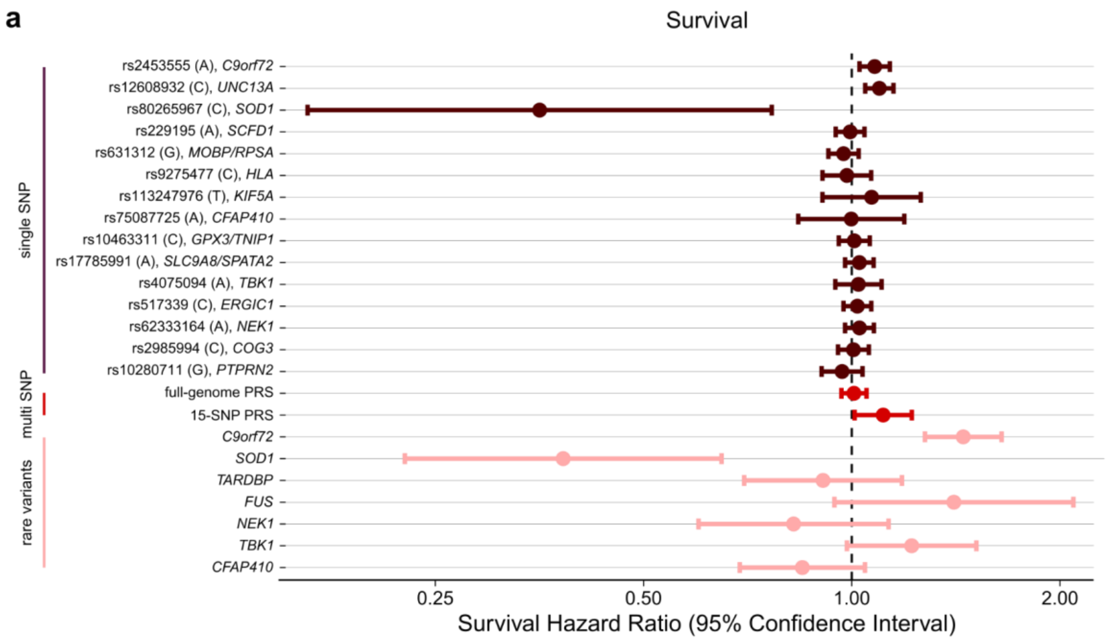
a
Survival
rs2453555 (A), C9orf72
rs12608932 (C), UNC13A
rs80265967 (C), SOD1
rs229195 (A), SCFD1
rs631312 (G), MOBP/RPSA
rs9275477 (C), HLA
rs113247976 (T), KIF5A
rs75087725 (A), CFAP410
rs10463311 (C), GPX3/TNIP1
rs17785991 (A), SLC9A8/SPATA2
rs4075094 (A), TBK1
rs517339 (C), ERGIC1
rs62333164 (A), NEK1
rs2985994 (C), COG3
rs10280711 (G), PTPRN2
full-genome PRS
15-SNP PRS
C9orf72
SOD1
TARDBP
FUS
NEK1
TBK1
CFAP410
single SNP
multi SNP
rare variants
0.25
0.50
1.00
2.00
Survival Hazard Ratio (95% Confidence Interval)

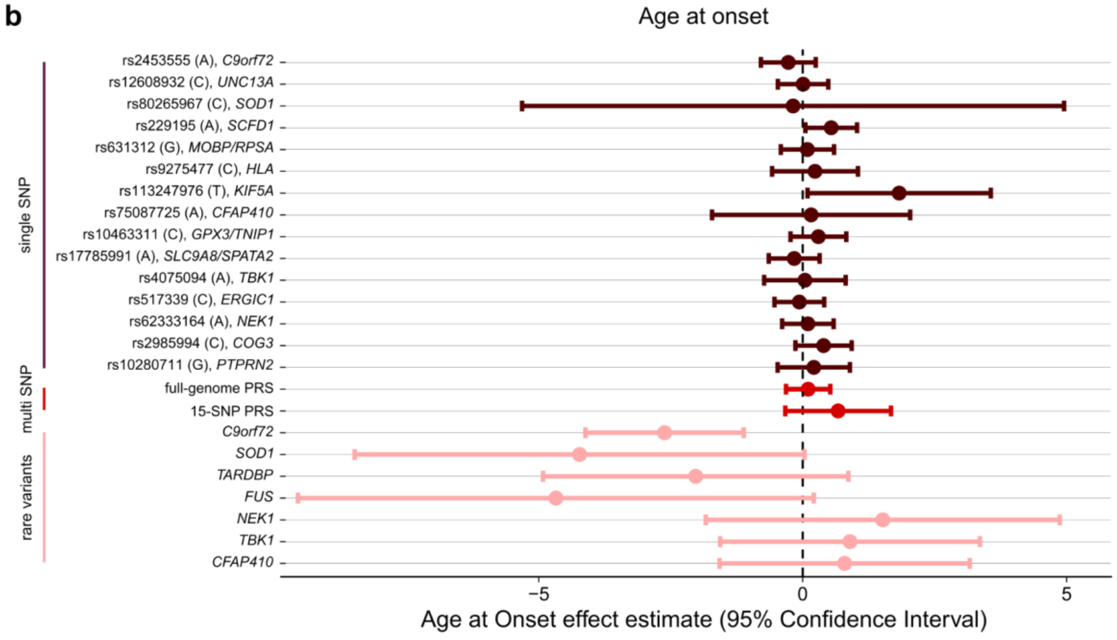
b
Age at onset
rs2453555 (A), C9orf72
rs12608932 (C), UNC13A
rs80265967 (C), SOD1
rs229195 (A), SCFD1
rs631312 (G), MOBP/RPSA
rs9275477 (C), HLA
rs113247976 (T), KIF5A
rs75087725 (A), CFAP410
rs10463311 (C), GPX3/TNIP1
rs17785991 (A), SLC9A8/SPATA2
rs4075094 (A), TBK1
rs517339 (C), ERGIC1
rs62333164 (A), NEK1
rs2985994 (C), COG3
rs10280711 (G), PTPRN2
full-genome PRS
15-SNP PRS
C9orf72
SOD1
TARDBP
FUS
NEK1
TBK1
CFAP410
single SNP
multi SNP
rare variants
−5
0
5
Age at Onset effect estimate (95% Confidence Interval)

4. Functional validation: The manuscript describes a wealth of analyses describing potential mechanisms by which common and rare ALS variants impact disease risk. Can any of these hypotheses be specifically evaluated? For example:

a. The authors posit that missense mutations explain the GWAS effect at four genes (SOD1, KIF5A, CFAP410, NEK1). Can they conduct in vitro experiments to explore whether these missense mutations lead to decreased protein function, activity or mutant gain of function?

b. Do disease relevant perturbations in gene expression (in vitro or in xenopus/zebrafish/mouse models) impact vesicle mediated transport, autophagy, and/or survival? Particularly in glutamatergic neurons?

c. Can the causal role of top colocalized risk variants be demonstrated through CRISPR-editing or reporter assays (up to and including MPRA)?

We thank the reviewer for these very interesting suggestions for functional characterization of the GWAS loci, this is exactly the kind of research we would like to inspire with our study. While follow-up experiments for GWAS loci have been challenging, very interesting results on ALS GWAS loci are present in literature. Here, we would like to highlight the UNC13A locus in which recent studies have shown that the GWAS SNPs act as splice-QTL only in the presence of TDP-43 dysfunction which results in retention of a cryptic exon.

We feel however, that the functional follow-up of these loci warrant rigorous multi-layered experiments which are currently beyond the scope of our study.

Decision Letter, first revision:

Our ref: NG-A57139R

19th Aug 2021

Dear Dr. van Rheenen,

Thank you for submitting your revised manuscript "Common and rare variant association analyses in Amyotrophic Lateral Sclerosis identify 15 risk loci with distinct genetic architectures and neuron-specific biology" (NG-A57139R). It has now been seen by the original referees and their comments are below. The reviewers find that the paper has improved in revision, and therefore we'll be happy in

principle to publish it in Nature Genetics, pending minor revisions to satisfy the referees' final requests and to comply with our editorial and formatting guidelines.

We are now performing detailed checks on your paper and will send you a checklist detailing our editorial and formatting requirements in about a week. Please do not upload the final materials and make any revisions until you receive this additional information from us.

Thank you again for your interest in Nature Genetics Please do not hesitate to contact me if you have any questions.

Sincerely,

Wei Li, PhD
Senior Editor
Nature Genetics
New York, NY 10004, USA
www.nature.com/ng

Reviewer #1 (Remarks to the Author):

the authors have responded thoroughly to reviewer suggestions and I have no further concerns or suggestions.

Reviewer #2 (Remarks to the Author):

The authors have done an excellent job of addressing my comments. With regard to the MR analysis, they correctly interpreted my suggestion to include the beta from the model.

I have one outstanding minor point. Has the SNP-heritability estimate from LDSC been converted to the liability scale, to adjust for the proportion of cases in the GWAS sample and the prevalence of ALS in the general population? Could the authors please add the prevalence estimate that was used for the conversion and the P value for the heritability.

Reviewer #3 (Remarks to the Author):

The authors have done an excellent job responding to my criticisms. I am happy to now recommend this manuscript for publication in Nature Genetics.

naturerearch

Final Decision Letter:

In reply please quote: NG-A57139R1 van Rheenen

18th Oct 2021

Dear Dr. van Rheenen,

I am delighted to say that your manuscript "Common and rare variant association analyses in Amyotrophic Lateral Sclerosis identify 15 risk loci with distinct genetic architectures and neuron-specific biology" has been accepted for publication in an upcoming issue of Nature Genetics.

Prior to setting your manuscript, we may make minor changes to enhance the lucidity of the text and with reference to our house style. We therefore ask that you examine the proofs most carefully to ensure that we have not inadvertently altered the sense of your text in any way.

Once your manuscript is typeset and you have completed the appropriate grant of rights, you will receive a link to your electronic proof via email with a request to make any corrections within 48 hours. If, when you receive your proof, you cannot meet this deadline, please inform us at rjsproduction@springernature.com immediately.

Your paper will be published online after we receive your corrections and will appear in print in the next available issue. You can find out your date of online publication by contacting the Nature Press Office (press@nature.com) after sending your e-proof corrections. Now is the time to inform your Public Relations or Press Office about your paper, as they might be interested in promoting its publication. This will allow them time to prepare an accurate and satisfactory press release. Include your manuscript tracking number (NG-A57139R1) and the name of the journal, which they will need when they contact our Press Office.

Before your paper is published online, we shall be distributing a press release to news organizations worldwide, which may very well include details of your work. We are happy for your institution or funding agency to prepare its own press release, but it must mention the embargo date and Nature Genetics. Our Press Office may contact you closer to the time of publication, but if you or your Press Office have any enquiries in the meantime, please contact press@nature.com.

Acceptance is conditional on the data in the manuscript not being published elsewhere, or announced in the print or electronic media, until the embargo/publication date. These restrictions are not intended to deter you from presenting your data at academic meetings and conferences, but any enquiries from the media about papers not yet scheduled for publication should be referred to us.

Please note that <i>Nature Genetics</i> is a Transformative Journal (TJ). Authors may publish their research with us through the traditional subscription access route or make their paper immediately open access through payment of an article-processing charge (APC). Authors will not be required to make a final decision about access to their article until it has been accepted. Find out more about Transformative Journals

<B>Authors may need to take specific actions to achieve compliance with funder and institutional open access mandates. For submissions from January 2021, if your research is supported by a funder that requires immediate open access (e.g. according to Plan S principles) then you should select the gold OA route, and we will direct you to the compliant route where possible. For authors selecting the subscription publication route our standard licensing terms will need to be accepted, including our self-archiving policies. Those standard licensing terms will supersede any other terms that the author or any third party may assert apply to any version of the manuscript.

Please note that Nature Research offers an immediate open access option only for papers that were first submitted after 1 January, 2021.

You will not receive your proofs until the publishing agreement has been received through our system.

If you have any questions about our publishing options, costs, Open Access requirements, or our legal forms, please contact ASJournals@springernature.com

If you have posted a preprint on any preprint server, please ensure that the preprint details are updated with a publication reference, including the DOI and a URL to the published version of the article on the journal website.

To assist our authors in disseminating their research to the broader community, our SharedIt initiative provides you with a unique shareable link that will allow anyone (with or without a subscription) to read the published article. Recipients of the link with a subscription will also be able to download and print the PDF.

As soon as your article is published, you will receive an automated email with your shareable link.

You can now use a single sign-on for all your accounts, view the status of all your manuscript submissions and reviews, access usage statistics for your published articles and download a record of your refereeing activity for the Nature journals.

An online order form for reprints of your paper is available at https://www.nature.com/reprints/author-reprints.html. Please let your coauthors and your institutions' public affairs office know that they are also welcome to order reprints by this method.

If you have not already done so, we invite you to upload the step-by-step protocols used in this manuscript to the Protocols Exchange, part of our on-line web resource, natureprotocols.com. If you complete the upload by the time you receive your manuscript proofs, we can insert links in your article that lead directly to the protocol details. Your protocol will be made freely available upon publication of your paper. By participating in natureprotocols.com, you are enabling researchers to more readily reproduce or adapt the methodology you use. Natureprotocols.com is fully searchable, providing your protocols and paper with increased utility and visibility. Please submit your protocol to

https://protocolexchange.researchsquare.com/. After entering your nature.com username and password you will need to enter your manuscript number (NG-A57139R1). Further information can be found at https://www.nature.com/nprot/.

Sincerely,

Wei Li, PhD
Senior Editor
Nature Genetics
New York, NY 10004, USA
www.nature.com/ng
